# Deaminative cross-coupling of amines by boryl radical β-scission

**DOI:** 10.1038/s41586-025-09725-1

**Published:** 2025-10-15

**Authors:** Zhenhua Zhang, Giovanni Lonardi, Thomas Sephton, Yusuf C. Guersoy, Chiara Stavagna, Giovanni V. A. Lenardon, Massimo Bietti, Daniele Leonori

**Affiliations:** 1 Institute of Organic Chemistry, RWTH Aachen University, Aachen, Germany; 2 Dipartimento di Scienze e Tecnologie Chimiche, Università “Tor Vergata”, Rome, Italy

**Keywords:** Synthetic chemistry methodology, Homogeneous catalysis, Synthetic chemistry methodology

## Abstract

Amines are among the most common functional groups in biologically active molecules and pharmaceuticals^1–3^, yet they are almost universally treated as synthetic end points^4^. Here we report a strategy that repositions native primary, secondary and tertiary amines as handles for cross-coupling. The platform relies on in situ activation through borane coordination and exploits a copper catalytic redox system that generates amine-ligated boryl radicals, which undergo β-scission across the C(*sp*^3^)–N bond to release alkyl radicals. These intermediates engage in copper-catalysed cross-couplings with a broad range of C-based, N-based, O-based and S-based nucleophiles. The method tolerates diverse amine classes, enables modular functionalization and supports late-stage diversification of complex drug scaffolds. Also, amides can be incorporated into the manifold through reductive funnelling. This work establishes a general approach to deaminative C–N bond functionalization and introduces a distinct approach for making and modifying drug-like molecules.

## Abstract (web summary)

A strategy is described making use of in situ activation of radical boron chemistry to convert amines to handles for cross-coupling, enabling a range of useful chemistries to make and modify drug-like molecules.

## Main

**Fig. 1:**
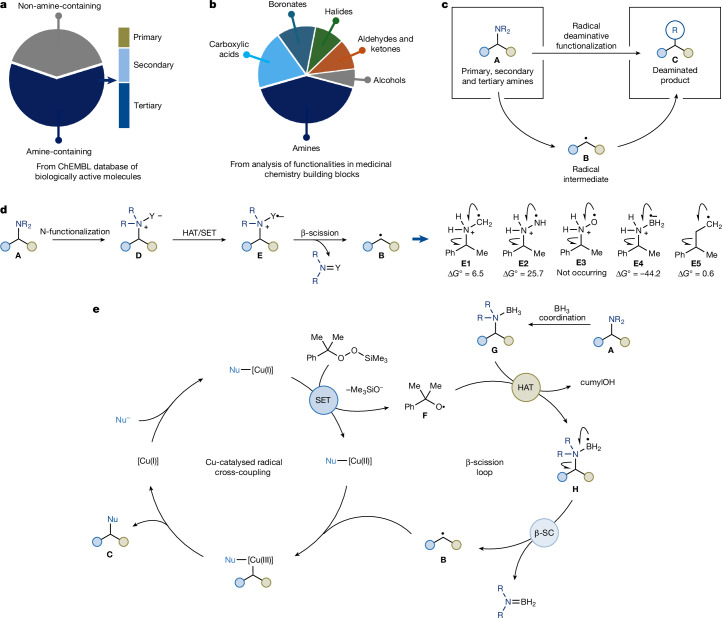
Relevance of amines and their functionalization through deaminative radical generation. **a**, Amines, and more specifically tertiary amines, are one of the most common functionalities in biologically active molecules. **b**, Amines are the most common functionality in the building blocks used in medicinal chemistry research. **c**, Strategy enabling the use of primary, secondary and tertiary amines in deaminative cross-coupling through radical intermediates. **d**, Proposed amine activation for C(*sp*^3^)–N β-scission and computational studies (ωB97X-D3/def2-TZVP//ωB97X-D3/def2-SVP). Δ*G*° values are in kcal mol^−1^. **e**, Proposed copper catalytic manifold for deaminative cross-coupling through β-scission of amine-ligated boryl radicals. β-SC, β-scission; Nu, nucleophile.

Amines are among the most widespread motifs in biologically active molecules^1^. They play critical roles in biological signalling (for example, neurotransmitters, hormones) and are embedded in the core of numerous pharmaceuticals and agrochemicals. A systematic analysis of the ChEMBL database^5^, comprising more than 420,000 unique biologically active small molecules, reveals that amines feature in more than 60% of entries. Tertiary amines rank as the third most common functional group, whereas secondary and primary amines are fifth and twelfth, respectively^2^ (Fig. 1a). Similarly, an AbbVie survey of building blocks used in medicinal chemistry campaigns found amines to be the most abundant functional group across synthetic intermediates for drug-discovery programmes^3^ (Fig. 1b). This prevalence as both targets and precursors, combined with their structural diversity and accessibility, makes amines prime candidates not only as end targets but also as starting points for molecular diversification^6^.

Yet, in synthesis, amines are almost exclusively treated as end points. Unlike alkyl halides or carboxylic acids, which serve as standard retrons for derivatization, amines are rarely used for direct functional group interconversion^7^. From a medicinal chemistry perspective, however, the ability to interconvert amines with other polar or non-polar functionalities would be of profound value^6,8^. Even a single change at the amine site can greatly alter pharmacological profiles, biological availability and metabolic stability. For example, introduction of a methylamine into penicillin G furnishes ampicillin, with enhanced Gram-negative activity^9^. Conversely, replacing the same amine with a hydroxyl or a carboxylic acid modifies both scope and potency. Similarly, conversion of the primary amine in the cyclopropyl anti-cancer agent ACC into an alcohol, nitrile or carboxylate (for example, NSC154619) yields distinct biological effects^10^. Even subtle changes, such as formation of alcohol metabolites from dapoxetine^11^ or sertraline^12^, can affect efficacy and require monitoring during manufacturing.

Despite this functional leverage, each deaminated analogue typically requires a new synthetic route, often involving multistep sequences with protection/deprotection or specialized precursors. This inefficiency arises from the poor leaving group character of amines, the high C(*sp*^3^)–N bond dissociation energies (>80 kcal mol^−1^)^13^ and their intrinsic basicity, all of which make direct deaminative functionalization largely elusive. Radical-based retrosynthetic logic has revolutionized C–C and C–Y (Y = heteroatom) bond construction but general radical deamination strategies across primary, secondary and tertiary amines remain undeveloped^4^. Present methods largely target primary amines through pre-functionalization or oxidation-state modulation (for example, diazonium salts, pyridinium, quaternary ammonium intermediates)^14–20^. However, these cannot provide a unified platform in which all amine classes undergo identical deaminative radical generation and downstream cross-coupling.

In this work, we report a strategy for divergent deaminative functionalization, enabled by in situ activation of amines through borane coordination (Fig. 1c). This process delivers amine–borane complexes that are generally stable species^21^. However, under a copper-based redox system, they undergo homolytic activation to yield amine-ligated boryl radicals. Instead of using these intermediates in standard radical settings such as borylation^22^ or halogen-atom transfer (XAT)^23^, we exploit their potential to undergo β-scission across the C(*sp*^3^)–N bond and convert amines **A** into alkyl radicals **B**. These species can be engaged in a series of cross-couplings with a wide range of carbon and heteroatom nucleophiles (N, O, S and F), enabling the direct replacement of the amino group with diverse functionalities (**C**). This approach applies to primary, secondary and tertiary amines, overcomes some of the long-standing barriers to C–N bond activation and offers new retrosynthetic logic for the diversification of complex molecules, including late-stage modifications of drugs.

Direct homolysis of C(*sp*^3^)–N bonds in amines remains an unsolved problem in radical chemistry. On oxidation or H-atom transfer (HAT)^24^, amines typically form α-aminoalkyl radicals that are too stable to undergo β-scission and the amine itself is a notoriously poor leaving group. This combination makes C–N bonds intrinsically resistant to fragmentation and has prevented the use of amines as radical precursors in cross-coupling chemistry.

We suggested that this barrier could be overcome by installing a polarizable substituent (Y) at nitrogen (**D**) to access α-ammonium radicals (**E**) predisposed to β-fragmentation (Fig. 1d). For this strategy to succeed, the substituent must be: (1) easily installable onto primary, secondary and tertiary amines leading to ammonium species (**D**); (2) capable of radical generation (**E**) through either single-electron transfer (SET) or HAT; and (3) able to destabilize the resulting radical intermediate sufficiently to make β-scission both thermodynamically favourable and kinetically viable. A strong driving force, such as formation of an N = Y *π*-bond, could further promote bond cleavage.

To explore this concept, we performed density functional theory calculations on a series of N-Bn α-ammonium radicals exploring their β-scission chemistry. The reference system (Y = CH_2_, **E1**) showed endothermic fragmentation, consistent with the inertness of these radicals. We next examined radicals bearing Y = NH• (**E2**) and O• (**E3**), mimicking hydrazine and hydroxylamine motifs. These two cases featured, respectively, highly endergonic fragmentation (**E2**) and facile carbocation rather than radical formation (**E3**) (see Supplementary Information). Combined with their limited synthetic accessibility, these motifs are poorly suited as general alkyl radical progenitors. By strong contrast, the Y = BH_2_•− system **E4** showed highly exergonic β-scission, driven by the formation of a strong B = N *π* system in the departing fragment. These results suggest the stable Lewis acid–base complex amine–borane^21^ as promising and modular precursors for C–N bond cleavage through radical pathways. Notably, the key amine-ligated boryl radical intermediate is formally isoelectronic with a conventional alkyl radical (**E5**), yet its reactivity diverges sharply. Although alkyl radicals are typically stable and do not fragment, our computational results indicate that boryl radical **E4** ought to undergo C(*sp*^3^)–N cleavage. This contrast arises from the polarized character of the B–N bond and the electrophilicity of boron, which destabilize the radical centre and bias the system towards bond scission. Important experimental support for our boryl-radical-based C(*sp*^3^)–N activation blueprint comes from electron paramagnetic resonance studies by Roberts and colleagues, who observed that (*i*-Pr)_2_EtN–BH_2_• generates *i*-Pr• through thermal β-scission^25,26^. However, this reactivity has never been synthetically exploited or even observed.

To translate this radical β-scission reactivity into a synthetically useful platform, we designed a copper-catalysed cross-coupling manifold for direct functionalization of amines with diverse nucleophiles (Fig. 1e). Mechanistically, we imagined a [Cu(I)/Cu(II)/Cu(III)] redox cycle initiated by nucleophile coordination to [Cu(I)] to give a Nu–[Cu(I)] species. At this point, SET with an oxidant such as cumylO_2_SiMe_3_ would generate a cumyloxy radical (**F**) and Nu–[Cu(II)]. The electrophilic cumylO• would serve as a polarity-matched HAT agent, selectively abstracting a H-atom from the amine–borane complex **G** (ref. ^27^), which can be conveniently accessed by in situ coordination of **A** with BH_3_. This step would form the amine-ligated boryl radical **H**. Following key β-scission, the liberated alkyl radical **B** would be captured by the Nu–[Cu(II)] complex, forming a putative alkyl, Nu–[Cu(III)] intermediate and forging the new C(*sp*^3^)–Nu bond in product **C** through reductive elimination. Alternatively, direct reaction between the alkyl radical **B** and the Nu–[Cu(II)] intermediate can also lead to product formation^28,29^.

**Fig. 2:**
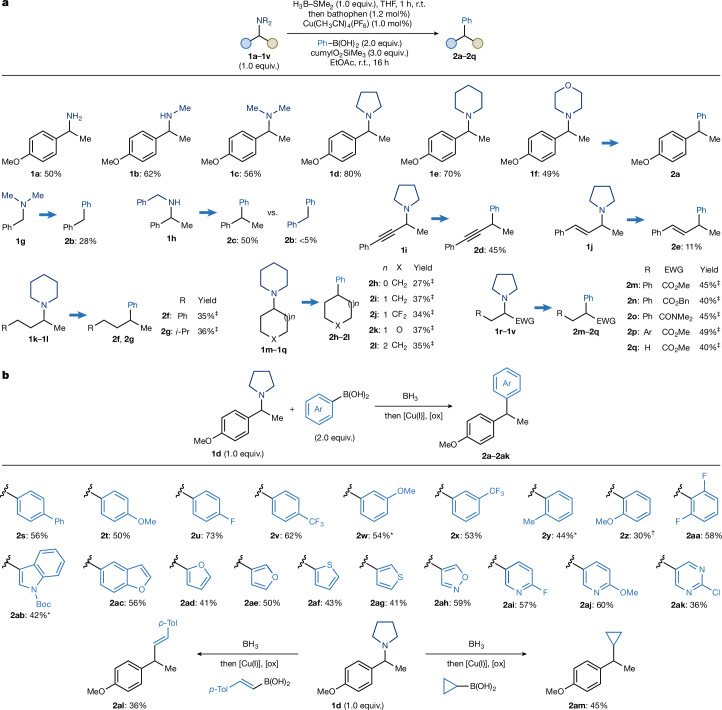
Scope for the deaminative arylation. **a**, Evaluation of amines **1a**–**1v** in the cross-coupling with Ph–B(OH)_2_. **b**, Scope of boronic acids in the deaminative cross-coupling with **1d**. ^‡^Reaction run with amine (2.0 equiv.), Ph–B(OH)_2_ (1.0 equiv.), 2.5 mol% [Cu] and 3 mol% bathophen. *Reaction run with 2.5 mol% [Cu] and 3 mol% bathophen. †Acetone was used as solvent. Ar, *p*-TBSO-C_6_H_4_; r.t., room temperature.

We began by testing the feasibility of this concept using a benzylic amine **1a** as a model radical precursor (Fig. 2a). Treatment of **1a** with H_3_B–SMe_2_ (1.0 equiv.) in THF provided the desired amine–borane that was directly engaged with Ph–B(OH)_2_ as the nucleophile under copper catalysis. Specifically, the use of Cu(CH_3_CN)_4_PF_6_ (1 mol%), bathophenanthroline (1.2 mol%), cumylO_2_SiMe_3_ (3.0 equiv.), in EtOAc at room temperature, gave the desired deamination-arylation product **2a** in 50% yield.

We then examined the influence of amine class and substitution. Both secondary (**1b**) and tertiary (**1c**) amines underwent smooth deaminative arylation, furnishing **2a** in 62% and 56% yield, respectively. Crucially, β-scission took place selectively at the benzylic C(*sp*^3^)–N bond, thus leading to the thermodynamic benzylic radical product^30^. We next evaluated medicinally relevant cyclic amines^31^. Substrates derived from pyrrolidine (**1d**), piperidine (**1e**) and morpholine (**1f**) underwent clean C(*sp*^3^)–N fragmentation and cross-coupling to **2a**, effectively replacing the nitrogen-containing ring with an aryl group in good to excellent yields. This transformation offers a powerful route to ‘molecular rigidification’ and ‘ring replacement’, two strategies often used to adjust physicochemical properties in lead optimization campaigns^32,33^.

By contrast, the benzylic amine **1g** afforded **2b** in lower yield, consistent with the expected decrease in the rate constant associated to the formation of a primary compared with a secondary radical. This divergence enabled a first look at chemoselectivity. Substrate **1h** featuring two potential benzylic C–N cleavage sites reacted exclusively at the position leading to a secondary radical giving **2c** with only traces of **2b**. This highlights the inherent response of the system to radical stabilization factors.

To experimentally investigate the kinetics of the C(*sp*^3^)–N bond fragmentation, we generated the amine-ligated boryl radical directly by means of nanosecond laser flash photolysis of the corresponding amine–borane complex **1c** under inert conditions (using HAT to cumylO•). The transient species exhibited a characteristic ultraviolet–visible absorption (*λ*_max_ = 360 nm) and decayed following first-order kinetics with a lifetime of *τ* = 5.7 μs at room temperature (*k*_obs_ ≈ 2 × 10^5 ^s^−^^1^) (see Supplementary Information for more details). This relatively long-lived behaviour suggests that β-scission is kinetically slow compared with most bimolecular boryl radical reactions (for example, XAT)^34^, which typically proceed on the sub-nanosecond timescale. The observed delay underscores the high kinetic barrier to C(*sp*^3^)–N cleavage, despite the favourable thermodynamics predicted by density functional theory (Fig. 1e). Notably, this slow unimolecular event is still successfully used in a catalytic manifold, suggesting that the surrounding redox and coordination environment, notably the rate of radical generation and copper-mediated capture, has been finely tuned to accommodate this constraint. These findings highlight the non-trivial kinetic balance required for this reaction to operate under mild conditions and distinguish this β-scission from faster, classical radical fragmentation processes (for example, radical probe processes).

The method was also compatible with other functionalized C(*sp*^3^) systems. A propargylic amine **1i** delivered the corresponding arylated product **2d** in 45% yield, with no detectable radical isomerization. By contrast, the allylic substrate **1j** showed extensive decomposition, delivering product **2e** in low yield, probably because of the competing side reactions of the allylic radical. Finally, we examined the strategy’s feasibility on substrates that generate unactivated alkyl radicals. Despite the inherent challenges associated with these β-fragmentations, we successfully used **1k**–**1q**, which afforded **2f**–**2l** in moderate to good yields. These substrates demonstrate deaminative cross-coupling over both acyclic and (hetero)cyclic systems of various size and also featuring HAT-activated positions (**1k** and **1p**). Furthermore, a series of amino acid derivatives **1r**–**1v** enabled direct modification at the N-terminus, a transformation orthogonal to conventional decarboxylative approaches. Although the resulting α-ester/amide radicals have moderate electrophilic character^35^, the desired products **2m**–**2q** were obtained in good yields. We propose that the stabilized nature of this radical intermediate facilitates β-fragmentation, thereby improving compatibility with the copper catalytic cycle and expanding the scope of this methodology. Amines leading to tertiary radicals were also examined. Although these derivatives could be activated, they did not furnish the arylated product but instead gave the hydro-deaminated derivative. We attribute this outcome to the lower propensity of tertiary radicals to engage in metal-catalysed cross-couplings, which increases the likelihood of competing HAT pathways (see Supplementary Information Section 7). Overall, although the present platform exhibits optimal reactivity with benzylic amines and substrates leading to stabilized radicals, these systems are particularly abundant in medicinal chemistry. Efforts to further control and push the β-fragmentation event to target further unactivated aliphatic systems are underway.

We then turned to the nucleophile scope (Fig. 2b). Using **1d** as the representative amine, we evaluated a broad range of aryl boronic acids. Both electron-rich (**2s** and **2t**) and electron-poor (**2u** and **2v**) *para*-substituted derivatives worked well, demonstrating tolerance with OMe, CF_3_ and F functionalities. Both *meta*-substituted (**2w** and **2x**) and *ortho*-substituted (**2y** and **2z**) arenes were installed effectively and 2,6-difluorophenylboronic acid gave product **2aa** despite its known tendency towards rapid protodeboronation under standard Suzuki conditions, an outcome we attribute to the anhydrous conditions of our reaction^36,37^.

We also explored heteroaryl boronic acids, which are challenging substrates under traditional coupling protocols. High-yielding couplings were achieved with indole (**2ab**), benzofuran (**2ac**), furans (**2ad** and **2ae**), thiophenes (**2af** and **2ag**), isoxazole (**2ah**) and 2,5-disubstituted pyridines (**2ai** and **2aj**), delivering products with handles amenable to S_N_Ar-based derivatization. 2-chloro-5-pyrimidine boronic acid also performed reliably (**2ak**), expanding the reach of the method into heterocycle-rich space.

Beyond arylation, we evaluated vinyl and cyclopropyl nucleophiles. Both species underwent productive coupling to assemble the corresponding C(*sp*^3^)–C(*sp*^2^) (**2al**) and C(*sp*^3^)–C(*sp*^3^) (**2am**) bonds in moderate to good yields. The formation of cyclopropylated product **2am** is particularly important, given the increasing use of this motif in drug discovery owing to its conformational and metabolic properties^38^.

A key conceptual feature of our platform is its potential for divergent reactivity. In principle, the same amine–borane precursor could undergo β-scission and radical capture with a wide variety of nucleophilic coupling partners, enabling modular interconversion of amines into many distinct functionalities. However, this poses a substantial practical challenge, as chemically diverse nucleophiles must behave similarly under a single catalytic regime, despite differences in polarity, transmetalation propensity, redox stability and ligand-exchange kinetics. To preserve the generality of the platform, we intentionally minimized individual reaction optimization and instead sought to establish proof of reactivity across key bond-forming classes (see Supplementary Information Sections 4.2 and 5 for optimization and specific reaction conditions). We focused on transformations of high relevance to medicinal chemistry, such as C–C, C–N, C–O and C–S bond construction.

**Fig. 3:**
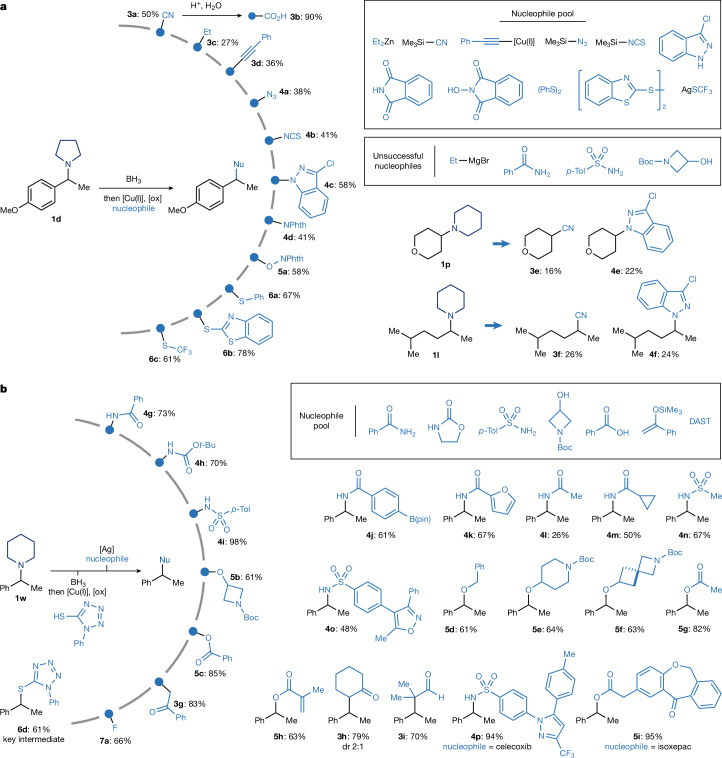
Nucleophile scope for deaminative functionalization. **a**, Divergent use of nucleophiles to enable direct deaminative cross-coupling on **1d**, leading to C(*sp*^3^)–C, C(*sp*^3^)–N, C(*sp*^3^)–O and C(*sp*^3^)–S bond formation. **b**, Two-step approach for deaminative diversification of **1w** through intermediate **6d**. Yields reported for products **3g**–**3i**, **4g**–**4p**, **5b**–**5i** and **7a** are from intermediate **6d**. Reaction conditions for the Ag-mediated nucleophile displacements: AgOTf (2.0 equiv.), nucleophiles (3.0 equiv.), CH_2_Cl_2_ (0.1 M), 3 Å molecular sieves, r.t., 16 h. See Supplementary Information for specific reaction conditions for the divergent functionalization. DAST, diethylaminosulfur trifluoride; dr, diastereomeric ratio.

We first explored carbon-based nucleophiles using **1d** as the representative amine (Fig. 3a). The use of Me_3_Si–CN enabled efficient deaminative cyanation, furnishing the corresponding nitrile **3a** in good yield. This represents a formal retro-Curtius transformation, converting a basic amine into a polar nitrile handle that was readily hydrolysed to the carboxylic acid **3b**. This conversion profoundly alters the physicochemical profile of the molecule, with implications for both lead diversification and metabolic pathway engineering^39^.

Encouraged by this, we evaluated preliminary alkyl cross-coupling reactions with organometallic reagents. Although the use of Grignard reagents resulted in no reactivity, treatment with Et_2_Zn afforded the corresponding ethylated product **3c** in 27% yield and the use of a copper acetylide delivered the alkynylated product **3d** in 36% yield. Although these yields are moderate, they serve as blueprints for reactivity, validating the feasibility of constructing C(*sp*^3^)–C(*sp*^3^) and C(*sp*^3^)–C(*sp*) bonds using this deaminative approach.

Unlike enzymatic transamination^39^, which requires carbonyl intermediates, there is no known biological or chemical system that directly replaces one amine with another N-functionality at a C(*sp*^3^) centre by means of radical intermediates. We demonstrate that our platform has some potential to enable this abiotic N-for-N exchange. Reaction of **1d** with Me_3_Si–N_3_ and Me_3_Si–NCS yielded the corresponding azide **4a** (38%) and isothiocyanate **4b** (41%) products, both valuable intermediates for further derivatization. Furthermore, indazole and phthalimide were engaged as nitrogen nucleophiles to give transaminated products **4c** and **4d** in 58% and 41% yield, respectively. Although common nucleophiles such as amides, carbamates and sulfonamides were unreactive, these results provide a proof of concept for a new disconnection logic for C(*sp*^3^)–N bonds directly from amines.

C–O bond formation is of particular interest for modulating polarity and metabolic profiles in drug leads. Although direct use of alcohols or phenols was unsuccessful under the standard conditions, we found that *N*-hydroxyphthalimide effectively replaced the amine moiety with an oxygen-based fragment, delivering the O-alkylated phthalimide product **5a** in good yield. This transformation serves as a formal retro-Mitsunobu reaction, in which subsequent SET reduction unveils the corresponding alcohol, offering a new route to amine-to-OH conversions relevant to fragment tuning and solubility optimization^40^.

Finally, we examined sulfur nucleophiles. Both diphenyl disulfide and 2,2′-dithiobis(benzothiazole) proved effective, delivering the corresponding thioether products (**6a** and **6b**) in high yields. Of note, the use of commercially available AgSCF_3_ enabled direct incorporation of the trifluoromethylthio (SCF_3_) group (**6c**), a highly valued motif in medicinal chemistry for modulating lipophilicity and metabolic resistance^41^. Notably, we have been able to extend the divergent functionalization platform to amines **1l** and **1p** that also underwent cyanation (**3e** and **3f**) and transamination with 2-Cl-indazole (**4e** and **4f**) in moderate yields.

We believe that the present study provides foundational evidence for a modular interconversion platform enabling construction of C–C, C–N, C–O and C–S bonds based on a single, native amine functional group. However, key limitations remain: several pharmaceutically relevant nucleophiles, such as alcohols, amides and carbamates, did not react under the standard conditions, probably because of mismatched polarities or [Cu(I/II)]-coordination profiles. Hence, we became interested by the possibility of decoupling the β-fragmentation event from nucleophile engagement and instead convert the amine into a stable, functionally versatile intermediate amenable to diversification. Inspired by reports showing that sulfides can be displaced by soft nucleophiles under Lewis-acidic conditions, we focused on developing an amine-to-sulfide transformation leading to a modular handle for further diversification^42,43^.

Using 1-phenyl-1H-tetrazole-5-thiol as a sulfur donor, we found that the corresponding alkyl sulfide **6d** could be formed in good yield under our standard copper catalytic conditions (Fig. 3b). This species served as a reactivity linchpin, undergoing a broad range of nucleophilic substitution reactions under silver Lewis-acid-promoted conditions. For instance, treatment with amides (**4g**), carbamates (**4h**) and sulfonamides (**4i**) lead to the replacement of the piperidine ring in **1w** with a series of diverse nitrogen-based functionalities, effectively accomplishing a two-step abiotic transamination.

This strategy also unlocked access to previously incompatible oxygen-based nucleophiles. Alcohols and carboxylic acids reacted smoothly with **6d** to furnish the corresponding ether (**5b**) and ester (**5c**), respectively, in good yields. Moreover, silyl enol ethers engaged efficiently, enabling direct installation of α-carbonyl groups in place of the original amine (**3g** and **3h**). This represents a powerful conversion, expanding the platform into polarity inversion chemistry. Finally, treatment with diethylaminosulfur trifluoride allowed for direct conversion of the amine into a fluorinated motif (**7a**) useful for pharmacokinetic tuning.

This reactivity could be easily extended to several other nucleophiles belonging to the classes discussed above (that is amides: **4j**–**4m**; sulfonamides: **4n** and **4o**; alcohols: **5d**–**5f**; carboxylates: **5g**–**5i**; and silyl enol ethers: **3h** and **3i**). The use of celecoxib (**4p**) and isoxepac (**5i**) illustrates the tolerance to complex and densely functionalized nucleophilic partners. Notably, some of these transformations could also be run in a fully telescoped fashion with no isolation of intermediate **6b** (see Supplementary Information Section 8).

**Fig. 4:**
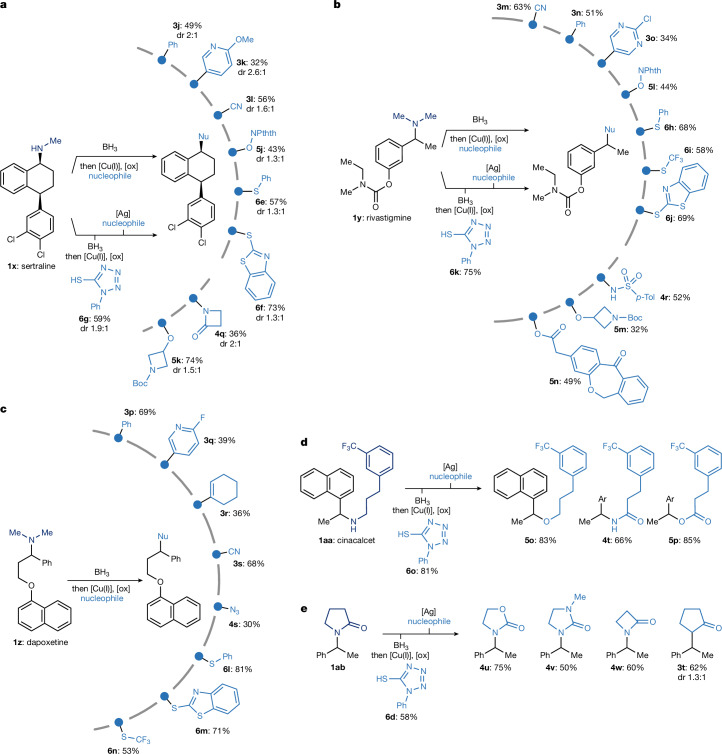
Divergent late-stage functionalizations via radical deamination. **a**, Late-stage functionalization of sertraline **1x**. Yields reported for products **4q** and **5k** are from intermediate **6g**. **b**, Late-stage functionalization of rivastigmine **1y**. Yields reported for products **4r**, **5m** and **5n** are from intermediate **6k**. **c**, Late-stage functionalization of dapoxetine **1z**. **d**, Diversification of the alkyl side chain of cinacalcet **1aa**. Yields reported for products **4t**, **5o** and **5p** are from intermediate **6o**. **e**, Use of deaminative couplings on lactam **1ab**. Yields reported for products **4u**–**4w** and **3t** are from intermediate **6d**. See Supplementary Information for specific reaction conditions for the divergent functionalization. Ar, 1-naphthyl.

Given the ubiquity of amines in pharmaceuticals, we were particularly interested in making use of our reaction manifold for divergent late-stage functionalization of complex drug molecules. As a case study, we selected three widely prescribed compounds and evaluated them under our standard conditions following in situ BH_3_ coordination. The antidepressant sertraline (**1x**) and Parkinson disease treatment rivastigmine (**1y**) feature benzylic secondary amines and other reactive motifs, including benzylic and α-N C(*sp*^3^)–H bonds (susceptible to HAT by electrophilic radicals), activated aryl chlorides (prone to XAT or metal-catalysed coupling) and a carbamate functionality. Under conventional late-stage editing strategies, these sites, particularly the benzylic or aryl halide positions, would typically be targeted for derivatization. By contrast, selective modification at the amine position remains out of reach. As a result, any analogue bearing structural changes at the amine site would generally require an independent de novo synthesis. Our platform overcomes this barrier: the NHMe moiety in sertraline was directly replaced with a range of electronically and structurally distinct fragments, including aryl (**3j** and **3k**), nitrile (**3l**), O-phthalimide (**5j**) and thioether (**6e** and **6f**) (Fig. 4a). Furthermore, the formation of intermediate **6g** enables further diversification with azetidinone (**4q**) and alcohol (**5k**) substituents. In the case of rivastigmine, we achieve ten different diversifications based on the replacement of the amine functionality with C-based (**3m**–**3o**), N-based (**4r**), O-based (**5l**–**5n**) and S-based substituents (**6h**–**6j**) (Fig. 4b). Each of these modifications would traditionally demand separate synthetic routes, yet here they are accessed from a common intermediate under a unified catalytic protocol.

Similarly, the selective serotonin reuptake inhibitor dapoxetine (**1z**) bearing a tertiary amine and other groups potentially incompatible under radical chemistry (for example, due to preferential oxidation of the naphthol unit) underwent smooth deamination functionalization, affording structurally diversified products (**3p**–**3s**, **4s** and **6l**–**6n**) in good to moderate yields (Fig. 4c). These transformations enable the rapid generation of analogues inaccessible with traditional methods, supporting applications in lead optimization and structure–activity exploration.

To further demonstrate the synthetic usefulness of our approach, we examined the calcium-sensing receptor agonist cinacalcet **1aa**, a drug featuring a benzylic amine adjacent to an alkyl chain (Fig. 4d). Following BH_3_ coordination and copper-catalysed thiolation, we obtained **6o**, serving as an entry point to downstream diversification. From this species, we prepared a set of derivatives in which the original NH group in the drug core was replaced by an O-atom (**5o**) or the non-benzylic α-amino methylene unit was oxidized to the corresponding amide (**4t**), a challenging transformation owing to the inherent tendency of oxidants to target the benzylic C(*sp*^3^)–H position. We further accessed ester analogue **5p** as a structural amide isostere. All three modifications were achieved in only two steps from the parent drug.

The use of this methodology extends beyond amines. We found that amides such as **1ab** can also be engaged through a tandem reduction–borane complexation sequence, enabling their incorporation into the deaminative platform (Fig. 4e). This enabled smooth conversion of **1ab** into **6d**, proving access to a range of structurally diverse heterocycles through precise skeletal modifications. Displacement with oxazolidin-2-one and 1-methylimidazolidin-2-one led to the formation of cyclic carbamate (**4u**) and urea (**4v**), effectively replacing the α-carbonyl methylene with an oxygen and a nitrogen atom, respectively. Substitution with an azetidinone led to a formal one-carbon-ring contraction, affording a four-membered heterocycle (**4w**). Finally, interception with a silyl enol ether delivered a product bearing an α-carbonyl methylene group in place of the original nitrogen, thus accomplishing a valuable N-to-C transmutation (**3t**). These transformations demonstrate that amide-derived substrates, once funnelled through the amine–borane manifold, can undergo precise and divergent functionalization.

## Conclusions

We have developed a strategy for radical-mediated deaminative cross-coupling that applies to all classes of amines (primary, secondary and tertiary) and is at present optimized for systems that proceed through benzylic and stabilized carbon radicals. This transformation exploits the unique ability of amine-ligated boryl radicals to undergo β-scission as blueprint for C(*sp*^3^)–N bond activation. This converts amines into alkyl radicals that engage in Cu-catalysed C(*sp*^3^)–C, C(*sp*^3^)–N, C(*sp*^3^)–O and C(*sp*^3^)–S bond formation. The methodology tolerates a wide range of amine classes, enables selective fragmentation even in complex environments and demonstrates high chemoselectivity. The approach is applicable to late-stage diversification of pharmaceuticals, providing divergent access to analogues that would otherwise require individual synthetic efforts. Although certain nucleophile classes remain challenging, the development of a modular amine-to-sulfide conversion offers a powerful workaround, enabling downstream diversification with otherwise incompatible nucleophiles under orthogonal conditions. We hope that the broad scope and mechanistic modularity will further stimulate the development of this concept positioning amine–boranes and their β-scission as a versatile tool for synthetic and medicinal chemistry.

## Online content

Any methods, additional references, Nature Portfolio reporting summaries, source data, extended data, supplementary information, acknowledgements, peer review information; details of author contributions and competing interests; and statements of data and code availability are available at 10.1038/s41586-025-09725-1.

## Supplementary information

Supplementary Information This file contains Supplementary Information.

Supplementary Data Cartesian coordinates.

## Data Availability

All data are available in the main text or the Supplementary Information and can also be obtained from the corresponding author on request.
