# Supplementary material for: Deaminative cross-coupling of amines by boryl radical β-scission
Source: Nature. 2025 Oct 15;647(8091):913–20. doi: 10.1038/s41586-025-09725-1 (PMC12657212; doi:10.1038/s41586-025-09725-1)
Supplement: Supplementary file 1 — This file contains Supplementary Information. [file 41586_2025_9725_MOESM1_ESM.pdf]

nature portfolio

https://doi.org/10.1038/s41586-025-09725-1

Supplementary information

Deaminative cross-coupling of amines by boryl radical β-scission

In the format provided by the authors and unedited

Deaminative cross-coupling of amines by boryl radical β-scission

Zhenhua Zhang,[1†] Giovanni Lonardi,[1†] Thomas Sephton,[1†] Yusuf C. Guersoy,[1] Chiara Stavagna,[1] Giovanni V. A. Lenardon,[1] Massimo Bietti[2] and Daniele Leonori[1]*

[1]*Institute of Organic Chemistry, RWTH Aachen University, Landoltweg 1, Aachen 52056, Germany*

[2]*Dipartimento di Scienze e Tecnologie Chimiche, Università "Tor Vergata", Via della Ricerca Scientifica, 1 I-00133 Rome, Italy*

daniele.leonori@rwth-aachen.de

† These authors contributed equally to this work. * Corresponding author.

Table of Contents

1 General Experimental Details ... 3

2 List of Amines ... 4

3 Starting Material Synthesis ... 5

4 Reaction Optimizations ... 8

4.1 Deaminative Arylation ... 8

4.2 Divergent Functionalizations ... 13

4.3 Nucleophilic Substitution ... 17

5 Deaminative Functionalizations ... 18

6 Substrate Scope ... 24

7 Tertiary Radicals Precursors ... 63

8 One-Pot Procedures ... 64

9 Laser Flash Photolysis Studies ... 65

10 UV/Vis Absorption Spectroscopy Studies ... 68

11 Mechanistic Experiments ... 70

12 Amine poisoning and stability of amine and amine–borane ... 71

13 Computational Studies ... 73

13.1 General Details ... 73

13.2 Level of Theory Selection ... 74

13.3 Nomenclature ... 76

13.4 Thermodynamics....77
14 NMR Spectra....86
15 Source Data Information....162
16 References....163

1 General Experimental Details

All required fine chemicals were used directly without purification unless stated otherwise. All air and moisture sensitive reactions were carried out under an argon atmosphere using standard Schlenk manifold technique. All solvents were bought from Acros as 99.8% purity. ^{1}H and ^{13}C Nuclear Magnetic Resonance (NMR) spectra were acquired at various field strengths as indicated and were referenced to $CDCl_3$ (7.26 and 77.16 ppm for ^{1}H and ^{13}C respectively). ^{1}H NMR coupling constants are reported in Hertz and refer to apparent multiplicities and not true coupling constants. Data are reported as follows: chemical shift, integration, multiplicity (s = singlet, br s = broad singlet, d = doublet, t = triplet, q = quartet, p = quintet, h = sextet, hept = septet, m = multiplet, dd = doublet of doublets, etc.), proton assignment (determined by 2D NMR experiments: NOESY, HSQC and HMBC) where possible. High-resolution mass spectra were obtained using a JEOL JMS-700 spectrometer or a Fissions VG Trio 2000 quadrupole mass spectrometer. Spectra were obtained using electron impact ionization (EI) and chemical ionization (CI) techniques, or positive electrospray (ES). Analytical TLC: aluminum backed plates pre-coated (0.25 mm) with Merck Silica Gel 60 F254. Compounds were visualized by exposure to UV-light or by dipping the plates in permanganate ($KMnO_4$) stain followed by heating. Flash column chromatography was performed using Merck Silica Gel 60 (40–63 µm). All mixed solvent eluents are reported as v/v solutions. UV/Vis spectroscopy studies were performed on a Duetta Fluorescence and Absorbance Spectrometer from Horiba Scientific, using quartz High Precision Cells from Hellma Analytics (10 mm × 10 mm). All yields are isolated unless specified otherwise.

2 List of Amines

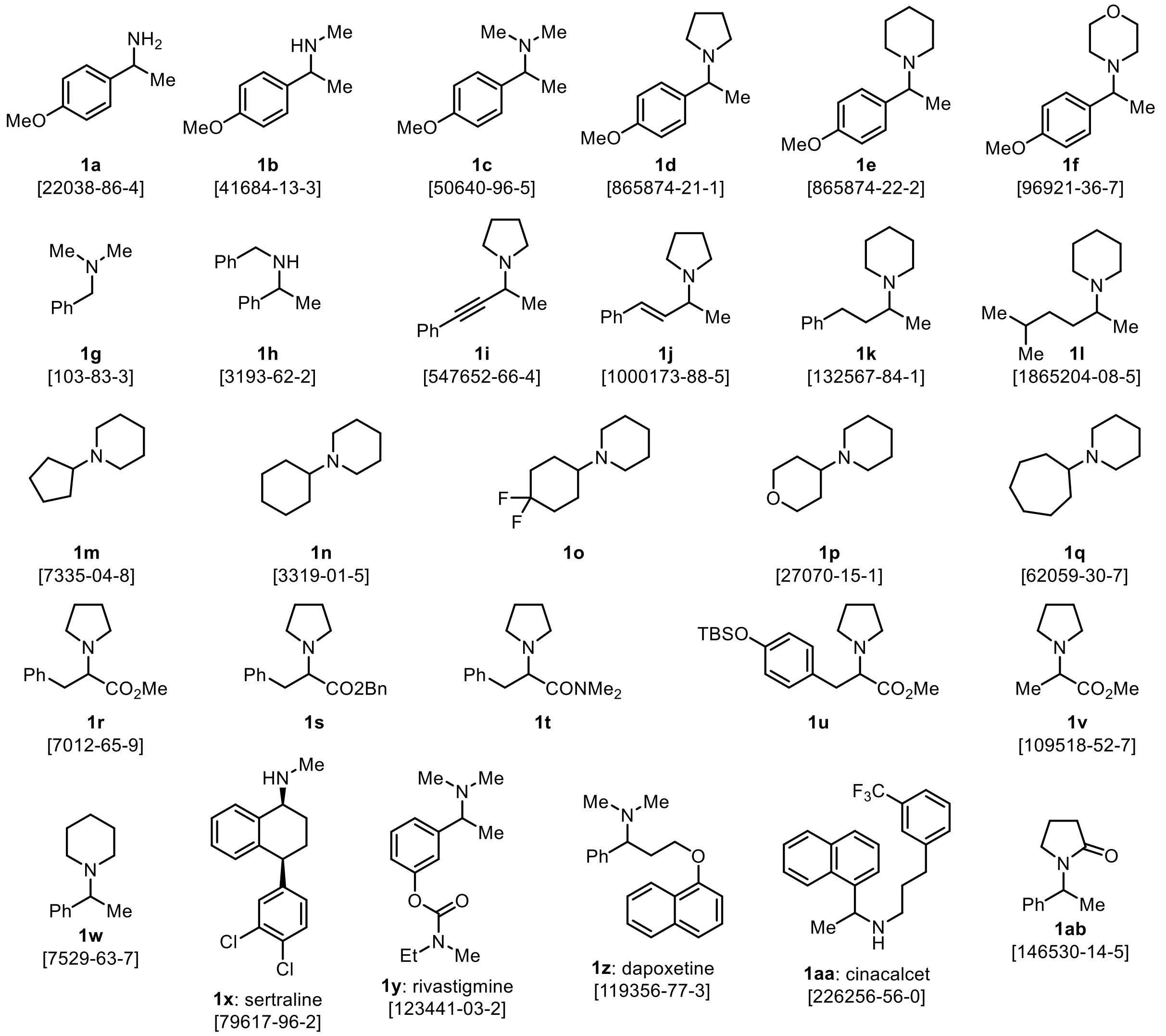

Figure S1.

3 Starting Material Synthesis

Trimethyl((2-phenylpropan-2-yl)peroxy)silane (CumylO$_2$SiMe$_3$)

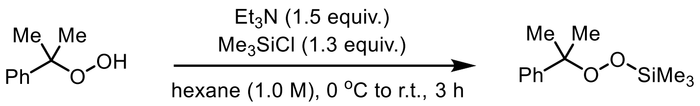

A round bottom flask equipped with a stirring bar was charged with Et_3N (10.5 mL, 75.0 mmol, 1.50 equiv.) and hexane (100 mL). cumylO$_2$H (9.3 mL, 80% technical solution, 50.0 mmol, 1.0 equiv.) was slowly added. The reaction media was then cooled in an ice-water bath and Me_3SiCl (8.25 mL, 65.0 mmol, 1.30 equiv.) was slowly added. The ice-water bath was removed, and the reaction was stirred at room temperature for 3 h. H_2O (100 mL) was added, the layers were separated and the organic phase was washed with H_2O (100 mL x 4) and brine (100 mL), dried ($MgSO_4$), filtered and evaporated to give cumylO$_2$SiMe$_3$ as an oil (10.97 g, 98%) with approx. density of 0.94 g/ml. ^{1}H NMR (400 MHz, $CDCl_3$) δ 7.45 (2H, d, *J* = 8.4 Hz), 7.33 (2H, t, *J* = 7.7 Hz), 7.24 (1H, t, *J* = 7.9 Hz), 1.58 (6H, s), 0.18 (9H, s); ^{13}C NMR (101 MHz, $CDCl_3$) δ 145.4, 127.9, 127.0, 125.6, 83.4, 26.5, –1.1. Data in accordance with literature.[1]

Safety Assessment of CumylO$_2$SiMe$_3$

We previously carried out a comprehensive safety assessment of cumylO$_2$SiMe$_3$, indicating that it can be stored in a conventional freezer (–20 °C) for several weeks without significant degradation. While not explosive, cumylO$_2$SiMe$_3$ does decompose exothermically at relatively low temperatures (>100 °C). For this reason, appropriate safety measures should be taken when handling this compound.[1]

1-(1-(4-Methoxyphenyl)ethyl)pyrrolidine Borane (S1)

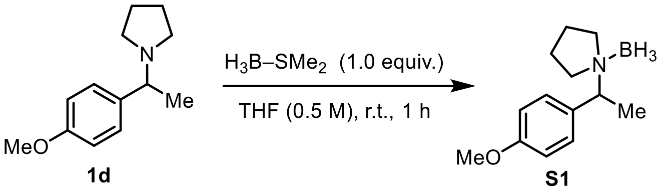

A round bottom flask equipped with a stirring bar was charged with **1d** (1.0 equiv.). The flask was evacuated and refilled with Ar (x 3) and dry THF (0.5 M) was added. $H_3B–SMe_2$ (1.0 equiv.) was added dropwise and the mixture was stirred for 1 h at r.t. The solvent was evaporated and the residue filtered through a silica plug eluting with CH_2Cl_2 to give **S1** as a solid (99%). R_f 0.95 [CH_2Cl_2]; ^{1}H NMR (600 MHz, $CDCl_3$) δ 7.36 (2H, d, *J* = 8.7 Hz), 6.87 (2H, d, *J* = 8.9 Hz), 3.92 (1H, q, *J* = 7.0 Hz), 3.81 (3H, s), 3.25–3.17 (1H, m), 2.94–2.87 (1H, m), 2.81 (1H, app q, *J* = 9.4 Hz), 2.71 (1H, app q, *J* = 8.9 Hz), 2.24–2.09 (2H, m), 1.74 (3H, d, *J* = 7.0 Hz), 1.72–1.67 (2H, m), 1.85–1.37 (3H, br m); ^{13}C NMR (151 MHz, $CDCl_3$) δ 159.8, 131.9, 130.0, 113.4, 70.3, 60.6, 57.9, 55.4, 22.8, 22.7, 18.3; ^{11}B NMR (192 MHz, $CDCl_3$) δ –13.9; HRMS (ESI): Found MNa^+ 242.16929, $C_{13}H_{22}ONBNa$ requires 242.16867.

General Procedure for the Amine Synthesis – GP0

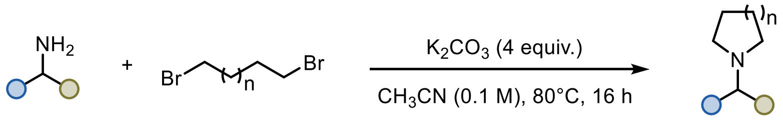

A round bottom flask equipped with a stirring bar was charged with the amine (if solid; 1.0 equiv.) and K_2CO_3 (4.0 equiv.). The flask was evacuated and refilled with Ar (x 3). Anhydrous CH_3CN (0.1 M) followed by the amine (if liquid; 1.0 equiv.) and the corresponding dibromoalkane (1.2 equiv.) were added. The mixture was stirred for 16 h at 80°C. The mixture was filtered through cotton, the solvent was evaporated and the residue was purified by column chromatography on silica gel to give the desired product.

1-(4,4-Difluorocyclohexyl)piperidine (1o)

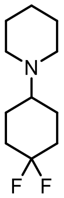

Following **GP0,** 4,4-Difluorocyclohexanamine (676 mg, 5 mmol, 1.0 equiv.) gave **1o** (72%) as an oil. R_f 0.14 [pentane:EtOAc (50:50)]; ^{1}H NMR (400 MHz, $CDCl_3$) δ 2.51 (4H, t, J = 5.4 Hz), 2.38 (1H, t, J = 11.2 Hz), 2.18–2.10 (2H, m), 1.85 (2H, d, J = 12.4 Hz), 1.77–1.56 (8H, m), 1.46–1.41 (2H, m); ^{13}C NMR (151 MHz, $CDCl_3$) δ 123.3 (t, J = 240.2 Hz), 62.1, 50.4, 33.0 (t, J = 25.5 Hz), 26.5, 24.8, 24.3 (d, J = 9.8 Hz), ^{19}F NMR (564 MHz, $CDCl_3$) δ –93.57 (d, J = 233.9 Hz), –101.29 (d, J = 235.4 Hz); HRMS (ESI): Found MH$^+$ 204.15540, $C_{11}H_{20}NF_2$ requires 240.15583.

Benzyl 3-phenyl-2-(pyrrolidin-1-yl)propanoate (1s)

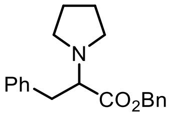

Following **GP0,** benzyl phenylalaninate (2.0 g, 6.9 mmol, 1.0 equiv.) gave **1s** (89%) as an oil. R_f 0.66 [pentane:EtOAc (50:50)]; ^{1}H NMR (600 MHz, $CDCl_3$) δ 7.30–7.26 (1H, m), 7.26–7.16 (1H, m), 7.19–7.13 (1H, m), 7.12–7.08 (1H, m), 5.01 (1H, d, J = 12.3 Hz), 4.96 (1H, d, J=12.2 Hz), 3.53 (1H, dd, J = 9.5, 6.0 Hz), 3.08 (1H, dd, J = 7.8, 5.1 Hz), 2.79 (1H, d, J = 7.7 Hz), 2.70–2.64 (1H, m), 1.82–1.74 (2H, m); ^{13}C NMR (101 MHz, $CDCl_3$) δ 172.0, 137.6, 135.6, 129.1, 128.5, 128.5, 128.3, 128.1 126.7, 68.4, 66.2, 50.6, 38.1, 23.6; HRMS (ESI): Found MNa$^+$ 332.16066, $C_{20}H_{23}NO_2$ requires 332.16210.

N,N-Dimethyl-3-phenyl-2-(pyrrolidin-1-yl)propanamide (1t)

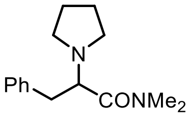

Following **GP0,** 2-amino-N,N-dimethyl-3-phenylpropanamide (577 mg, 3 mmol, 1.0 equiv.) gave **1t** (67%) as a solid. R_f 0.14 [EtOAc]; ^{1}H NMR (600 MHz, $CDCl_3$) δ 7.24 (2H, t, *J* = 7.8 Hz), 7.22–7.15 (3H, m), 3.72 (1H, dd, *J* = 10.6, 4.0 Hz), 3.17 (1H, t, *J* = 11.6 Hz), 2.98 (1H, dd, *J* = 12.7, 4.0 Hz), 2.84–2.77 (5H, m), 2.70–2.63 (2H, m), 2.60 (3H, s), 1.85–1.74 (4H, m); ^{13}C NMR (151 MHz, $CDCl_3$) δ 172.1, 138.7, 129.5, 128.4, 126. 5, 64.3, 50.5, 37.1, 36. 8, 35.8, 23.6; HRMS (ESI): Found MNa^+ 269.16159, $C_{15}H_{22}ON_2Na$ requires 269.16243.

Methyl 3-(4-((*tert*-Butyldimethylsilyl)oxy)phenyl)-2-(pyrrolidin-1-yl)propanoate (1u)

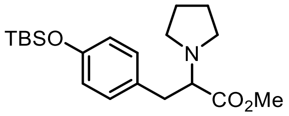

Following **GP0,** methyl 2-amino-3-(4-((*tert*-butyldimethylsilyl)oxy)phenyl)propanoate (3.71 g, 12 mmol, 1.0 equiv.) gave **1u** (78%) as an oil. R_f 0.63 [pentane:EtOAc (50:50)]; ^{1}H NMR (400 MHz, $CDCl_3$) δ 7.02 (2H, d, *J* = 8.0 Hz), 6.73 (2H, d, *J* = 8.4 Hz), 3.53 (3H, s), 3.36 (1H, dd, *J* = 10.0, 5.7 Hz), 3.02–2.92 (2H, m), 2.78–2.70 (2H, m), 2.65–2.56 (2H, m), 1.86–1.76 (4H, m), 0.96 (9H, s), 0.17 (6H, s); ^{13}C NMR (101 MHz, $CDCl_3$) δ 172.9, 154.4, 130.5, 130.1, 120.1, 69.1, 51.3, 50.9, 37.4, 25.8, 23.5, 18.4, -4.3; HRMS (ESI): Found MNa^+ 386.21121, $C_{20}H_{33}O_3NNaSi$ requires 386.21219.

4 Reaction Optimizations

To aid reaction development we have initially optimised the individual deaminative transformations on the isolate amine-borane complex **S1**.

4.1 Deaminative Arylation

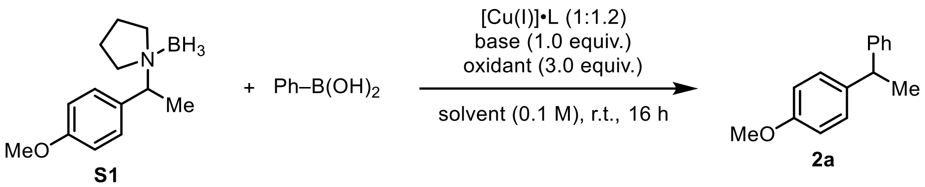

An oven-dried 5 mL microwave vial equipped with a stirring bar was charged with the [Cu] catalyst and ligand (1:1.2 ratio), **S1** (22 mg, 0.10 mmol, 1.0 equiv.), Ph–B$(OH)_2$ (18 mg, 0.15 mmol, 1.5 equiv.) and the base (0.1 mmol, 1 equiv.). The vial was capped with a Supelco aluminium crimp seal with septum (PTFE/butyl), evacuated and refilled with Ar (x 3). Dry and degassed solvent (1 mL) was then added. The mixture was stirred for 5 minutes at room temperature and then the oxidant (0.3 mmol, 3 equiv.) was slowly added. The mixture was then stirred at room temperature for 16 h. Upon completion, 1,3,5-trimethoxybenzene (17 mg, 0.1 mmol, 1 equiv.) was added and the volatiles were evaporated. The residue was dissolved in $CDCl_3$ (0.7 mL) and analysed by ^1H NMR spectroscopy to obtain the NMR yield.

Table S1.

Entry	Solvent	[Cu(I)] (mol%)	Ligand	Base	Oxidant	Yield (%)
solvent screening						
**1**	EtOAc	CuI (5)	**L1**	TMP	cumylO_2SiMe_3	50
**2**	DCE					11
**3**	DME					13
**4**	DMSO					0
**5**	benzene					3
**6**	CH_3CN					0
**7**	$PhCF_3$					6
**8**	acetone					18
[Cu(I)] screening						
**9**	EtOAc	CuCl (5)	**L1**	TMP	cumylO_2SiMe_3	32
**10**		CuBr (5)				52
**11**		$CuCl_2$ (5)				48
**12**		CuTC (5)				52
**13**		CuOTf (5)				52
**14**		CuOAc (5)				52
**15**		[Cu(CH_3CN)]PF_6 (5)				52
[Cu(I)] loading screening						
	EtOAc					
**16**		(2.5)				56
**17**		(7.5)				45

**18**		(10)	**L1**	TMP	cumylO_2SiMe_3	53
**19**		(15)				48
ligand screening						
**20**			**L2**			49
**21**			**L3**			62
**22**			**L4**			14
**23**			**L5**			35
**24**			**L6**			48
**25**			**L7**			15
**26**	EtOAc	$[Cu(CH_3CN)]PF_6$ (2.5)	**L8**	TMP	cumylO_2SiMe_3	67
**27**			**L9**			52
**28**			**L10**			46
**29**			**L11**			48
**30**			**L12**			20
**31**			**L13**			50
**32**			**L14**			0
base screening						
**33**				pyridine		61
**34**				2,6-lutidine		58
**35**				K_3PO_4		37
**36**				K_2CO_3	cumylO_2SiMe_3	46
**37**				KOAc		39
**38**				KF		48

**39**	EtOAc			LiOH•H_2O		48
**40**		[Cu(CH_3CN)]PF_6	**L8**	LiO*t*Bu		35
**41**		(2.5)		LiOMe		0
**42**				LiF		67
**43**				Na_2CO_3		45
**44**				Cs_2CO_3		38
**45**				–		67
loading screening						
**46**						70
**47**	EtOAc	[Cu(CH_3CN)]PF_6 (1)	**L8**	–	cumylO_2SiMe_3	70
**48**[a]						80
oxidant screening						
**49**					cumylO_2SiEt_3	70
**50**	EtOAc	[Cu(CH_3CN)]PF_6 (1)	**L8**	–	cumylO_2Si(*i*-Pr)$_3$	0
**51**					cumylO_2Si*t*-BuPh$_2$	0

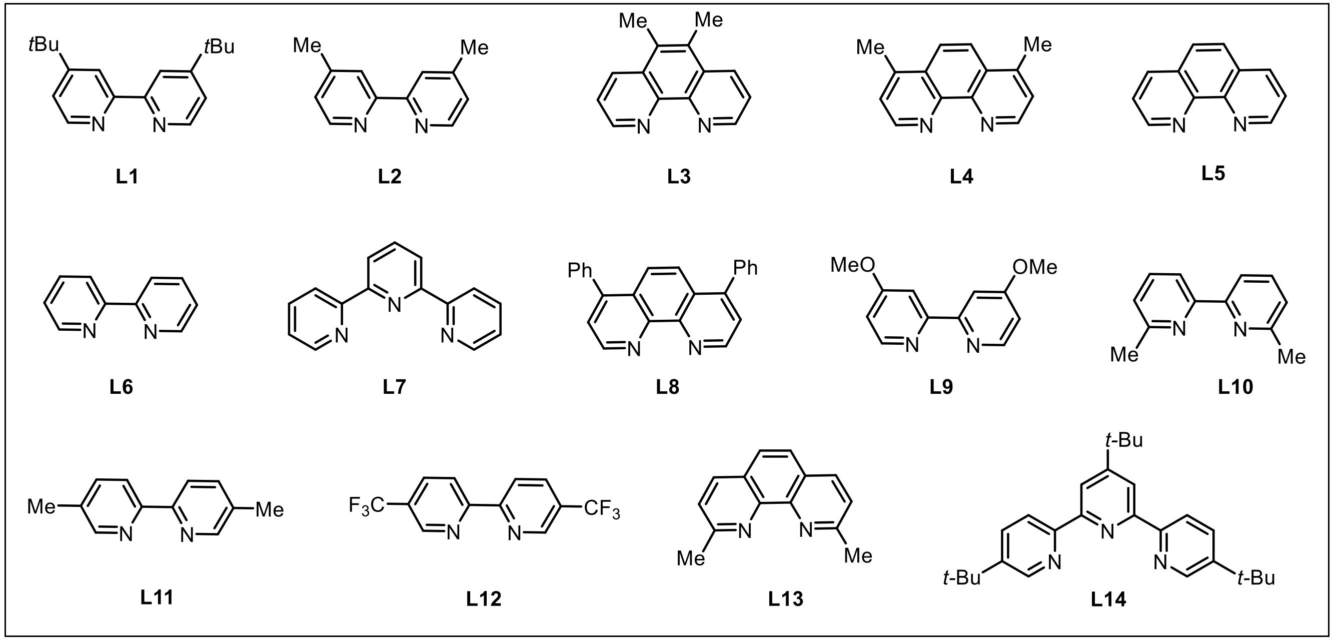

[a] 2 equiv. Ph–$B(OH)_2$

4.2 Divergent Functionalizations

In order to determine the divergency potential for functionalization of the Cu-strategy optimised in Section 4.1, we screened several nucleophiles and performed minimum optimization (Scheme S1). All reactions discussed in the Table **S2** were run using the same procedure discussed in section 4.1.

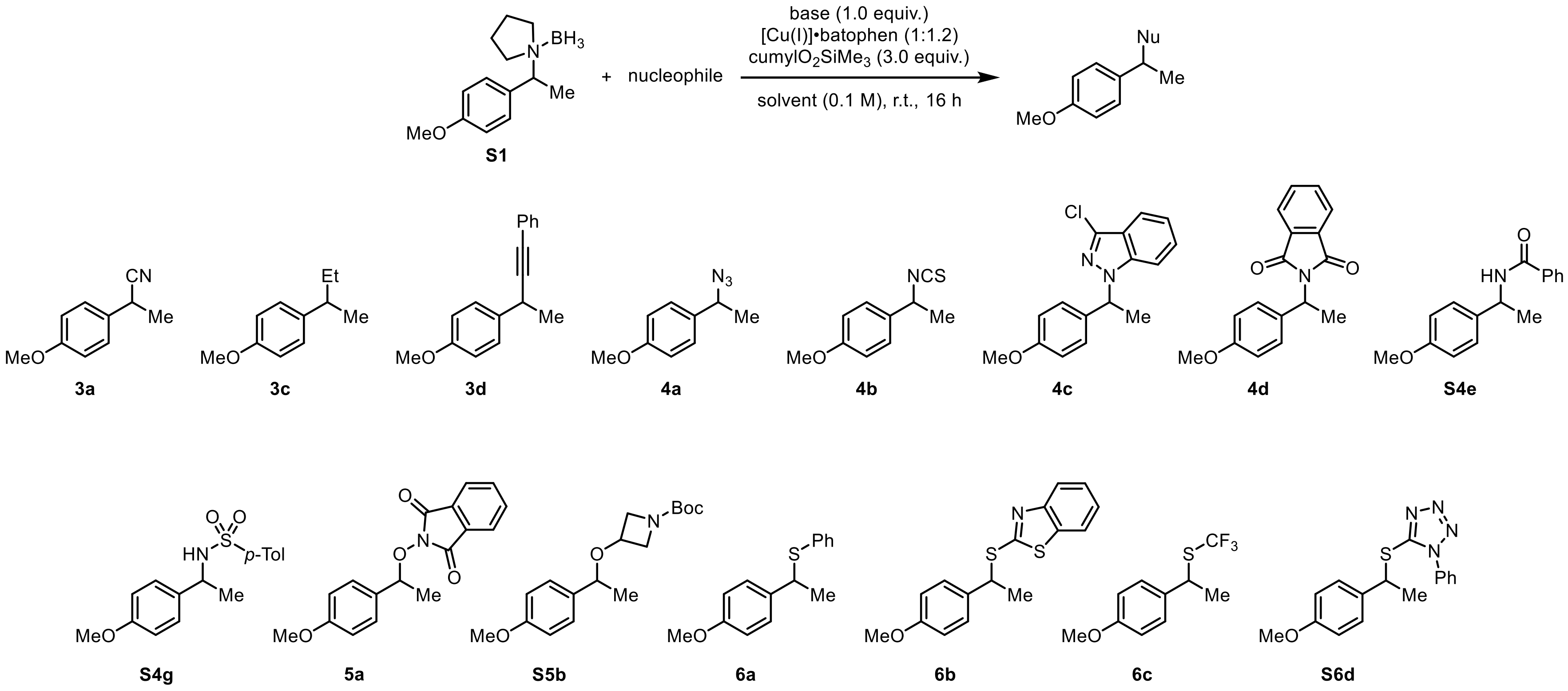

Scheme S1.

Table S2.

Entry	Nucleophile (equiv.)	Base (equiv.)	[Cu(I)] (mol%)	Solvent	Product	Yield (%)
**1**	Me_3SiCN (2.0)	–	$[Cu(CH_3CN)]PF_6$ (1)	EtOAc	**3a**	40
**2**		–	$[Cu(CH_3CN)]PF_6$ (5)	EtOAc		50
**3**	EtMgBr (2.0)	–	$[Cu(CH_3CN)]PF_6$ (5)	EtOAc	**3c**	–
**2**	Et_2Zn (2.0)	–	$[Cu(CH_3CN)]PF_6$ (2.5)	EtOAc		27
**3**		–	$[Cu(CH_3CN)]PF_6$ (10)	EtOAc		18
**4**		–	CuTC (10)	EtOAc		15
**5**	Phenylacetylene (2.0)	TMP (2.0)	$[Cu(CH_3CN)]PF_6$ (5)	EtOAc	**3d**	13
**6**		2,6-Lutidine (2.0)	$[Cu(CH_3CN)]PF_6$ (5)	PhF		22
**7**		TMP (2.0)	$[Cu(CH_3CN)]PF_6$ (5)	PhF		36
**8**	Me_3SiN_3 (2.0)	TMP (2.0)	$[Cu(CH_3CN)]PF_6$ (5)	EtOAc	**4a**	25
**9**		TMG (2.0)	$[Cu(CH_3CN)]PF_6$ (5)	EtOAc		17
**10**		TMG (2.0)	$[Cu(CH_3CN)]PF_6$ (5)	PhCl		38
**11**	Me_3SiNCS (2.0)	TMP (2.0)	$[Cu(CH_3CN)]PF_6$ (5)	EtOAc	**4b**	31
**12**		TMP (1.0)	$[Cu(CH_3CN)]PF_6$ (5)	EtOAc		17
**13**		TMP (2.0)	$[Cu(CH_3CN)]PF_6$ (5)	Acetone		41
**14**	3-Chloroindazole (1.5)	–	$[Cu(CH_3CN)]PF_6$ (2.5)	EtOAc	**4c**	10
**15**		TMP (2.0)	$[Cu(CH_3CN)]PF_6$ (10)	EtOAc		16
**16**		TMP (2.0)	CuI (10)	EtOAc		20
**17**		TMP (2.0)	CuTC (10)	EtOAc		26

Entry	Nucleophile (equiv.)	Base (equiv.)	[Cu(I)] (mol%)	Solvent	Product	Yield (%)
**18**		TMP (2.0)	CuTC (10)	$PhCF_3$		44
**19**		TMP (2.0)	CuTC (10)	PhF		58
**20**		TMP (2.0)	CuTC (2.5)	PhF		20
**21**		LiOtBu (2.0)	CuTC (10)	PhF		36
**22**		TMP (2.0)	CuTC (10)	PhF		15
**23**	Phthalimide (1.5)	TMP (2.0)	CuTC (10)	$PhCF_3$	**4d**	35
**24**		TMP (2.0)	CuTC (10)	PhF		41
**25**	Benzamide (1.5)	TMP (2.0)	CuTC (10)	PhF	**4e**	–
**26**	4-Toluenesulfonamide (1.5)	TMP (2.0)	CuTC (10)	PhF	**4g**	–
**27**	NHPI (2.0)	TMP (2.0)	$[Cu(CH_3CN)]PF_6$ (5)	EtOAc	**5a**	58
**28**		–	$[Cu(CH_3CN)]PF_6$ (5)	EtOAc		–
**29**		TMP (2.0)	$[Cu(CH_3CN)]PF_6$ (5)	PhCl		50
**30**	N-Boc azetidinone OH (2.0)	TMP (2.0)	$[Cu(CH_3CN)]PF_6$ (5)	EtOAc	**5b**	–
**31**	Ph_2S_2 (2.0)	TMP (2.0)	$[Cu(CH_3CN)]PF_6$ (5)	EtOAc	**6a**	31
**32**[a]	Ph_2S_2 (1.0)	TMP (1.0)	$[Cu(CH_3CN)]PF_6$ (5)	EtOAc		48
**33**[a]		TMP (1.0)	$[Cu(CH_3CN)]PF_6$ (5)	PhCl		67
**34**[a]	Benzothiazole disulfide (1.0)	TMP (1.0)	$[Cu(CH_3CN)]PF_6$ (5)	PhCl	**6b**	78
**35**[a]	$AgSCF_3$ (1.0)	TMP (1.0)	$[Cu(CH_3CN)]PF_6$ (5)	PhCl	**6c**	51
**36**[a]		TMP (1.0)	$[Cu(CH_3CN)]PF_6$ (5)	EtOAc		61
**37**[a]	SPTH (1.0)	TMP (1.0)	$[Cu(CH_3CN)]PF_6$ (5)	EtOAc	**6d**	70

Entry	Nucleophile (equiv.)	Base (equiv.)	[Cu(I)] (mol%)	Solvent	Product	Yield (%)
**38**[a]		TMP (1.0)	$[Cu(CH_3CN)]PF_6$ (5)	MeCN		49
**39**[a]		TMP (1.0)	$[Cu(CH_3CN)]PF_6$ (5)	PhCl		66

[a] 2.0 equiv. **S1**

4.3 Nucleophilic Substitution

This reactiivty was explored uisnf **6d** that features a Ph group as the one with a PMP unit was found unstable due to facile carbocation formation.

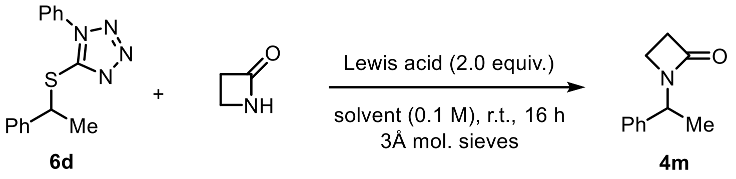

An oven-dried microwave vial equipped with a stirring bar was charged with **6d** (28 mg, 0.1 mmol, 1.0 equiv.), azetidinone (21 mg, 0.3 mmol, 3.0 equiv.), the Lewis acid (0.2 mmol, 2.0 equiv.) and 3Å molecular sieves (≈ 10 mg, activated by heating at 200 °C overnight). The vial was capped with a Supelco aluminium crimp seal with septum (PTFE/butyl), evacuated and refilled with Ar (x 3). Dry solvent (1 mL) was added, and the mixture was stirred for 16 h at r.t. The reaction was diluted with CH_2Cl_2 and filtered over silica, washing with CH_2Cl_2. 1,3,5-trimethoxybenzene (17 mg, 0.1 mmol, 1 equiv.) was added and the volatiles were evaporated. The residue was dissolved in $CDCl_3$ (0.7 mL) and analysed by ^{1}H NMR spectroscopy to obtain the NMR yield.

Table S3.

Entry	Solvent	Lewis acid	Yield (%)
		solvent screening	
**1**	$PhCF_3$	Ag(OTf)	50
**2**	DCE		5
**3**	CH_2Cl_2		60
		Lewis acid screening	
**4**	CH_2Cl_2	$AlCl_3$	0
**5**		$FeCl_3$	0
**6**		$Cu(OTf)_2$	0
**7**		$Zn(OTf)_2$	0
**8**		$Sc(OTf)_3$	0
**9**		$Ce(OTf)_3$	0
**10**		$Al(OTf)_3$	0
**11**		$InBr_3$	0
**12**		$Et_2O–BF_3$	0

5 Deaminative Functionalizations

General Procedure for the Deamination-Arylation Reaction – GP1

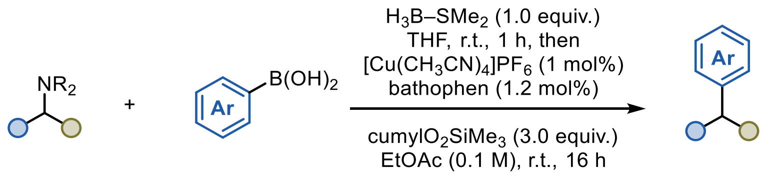

An oven-dried microwave vial equipped with a stirring bar was charged with the amine (1.0 equiv.), capped with a Supelco aluminium crimp seal with septum (PTFE/butyl), evacuated and refilled with Ar (x 3). Dry THF (0.1 M) was added, followed by $H_3B–SMe_2$ (1.0 equiv.). The mixture was stirred for 1 h at r.t. The volatiles were evaporated, and the vial was charged with $[Cu(CH_3CN)_4]PF_6$ (1 mol%), **L8** (1.2 mol%) and the boronic acid (2.0 equiv.). The vial was capped with a Supelco aluminium crimp seal with septum (PTFE/butyl), evacuated and refilled with Ar (x 3). Dry and degassed EtOAc (0.1 M) was added. The mixture was stirred for 5 min and then $cumylO_2SiMe_3$ (3.0 equiv.) was slowly added. The reaction was stirred for 16 h at r.t. The volatiles were evaporated, and the crude was purified by flash column chromatography on silica gel to give the desired product.

General Procedure for the Deamination-Cyanation Reaction – GP2

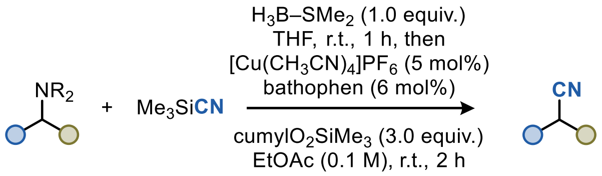

An oven-dried microwave vial equipped with a stirring bar was charged with the amine (1.0 equiv.), capped with a Supelco aluminium crimp seal with septum (PTFE/butyl), evacuated and refilled with Ar (x 3). Dry THF (0.1 M) was added, followed by $H_3B–SMe_2$ (1.0 equiv.). The mixture was stirred for 1 h at r.t. The volatiles were evaporated, and the vial was charged with $[Cu(CH_3CN)_4]PF_6$ (5.0 mol%) and **L8** (6.0 mol%). The vial was capped with a Supelco aluminium crimp seal with septum (PTFE/butyl), evacuated and refilled with Ar (x 3). Dry and degassed EtOAc (0.1 M) and Me_3SiCN (2.0 equiv.) were added. The mixture was stirred for 5 min and then $cumylO_2SiMe_3$ (3.0 equiv.) was slowly added. The reaction was stirred for 2 h at r.t.. The volatiles were evaporated, and the crude was purified by flash column chromatography on silica gel to give the desired product.

General Procedure for the Deamination-Alkylation Reaction – GP3

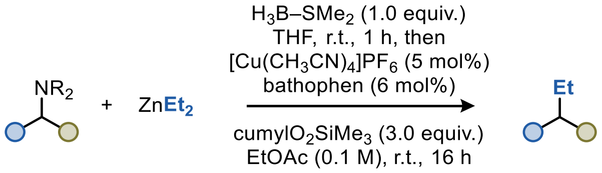

An oven-dried microwave vial equipped with a stirring bar was charged with the amine (1.0 equiv.), capped with a Supelco aluminium crimp seal with septum (PTFE/butyl), evacuated and refilled with Ar (x 3). Dry THF (0.1 M) was added, followed by $H_3B–SMe_2$ (1.0 equiv.). The mixture was stirred for 1 h at r.t. The volatiles were evaporated, and the vial was charged with $[Cu(CH_3CN)_4]PF_6$ (5.0 mol%) and **L8** (6.0 mol%). The vial was capped with a Supelco aluminium crimp seal with septum (PTFE/butyl), evacuated and refilled with Ar (x 3). Dry and degassed EtOAc (0.1 M) and Et_2Zn (1.0 M in hexane, 1.5 equiv.) were added. The mixture was stirred for 5 min and then cumylO_2SiMe_3 (3.0 equiv.) was slowly added. The reaction was stirred for 16 h at r.t. The volatiles were evaporated, and the crude was purified by flash column chromatography on silica gel to give the desired product.

General Procedure for the Deamination-Alkynylation Reaction – GP4

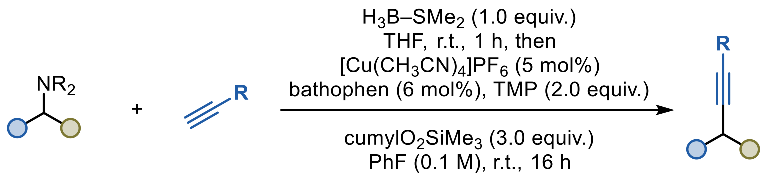

An oven-dried microwave vial equipped with a stirring bar was charged with the amine (1.0 equiv.), capped with a Supelco aluminium crimp seal with septum (PTFE/butyl), evacuated and refilled with Ar (x 3). Dry THF (0.1 M) was added, followed by $H_3B–SMe_2$ (1.0 equiv.). The mixture was stirred for 1 h at r.t. The volatiles were evaporated, and the vial was charged with $[Cu(CH_3CN)_4]PF_6$ (5.0 mol%), and **L8** (6.0 mol%). The vial was capped with a Supelco aluminium crimp seal with septum (PTFE/butyl), evacuated and refilled with Ar (x 3). Dry and degassed PhF (0.1 M), TMP (2.0 equiv.) and the corresponding alkyne (2.0 equiv.) were added. The mixture was stirred for 5 min and then cumylO_2SiMe_3 (3.0 equiv.) was slowly added. The reaction was stirred for 16 h at r.t. The volatiles were evaporated, and the crude was purified by flash column chromatography on silica gel to give the desired product.

General Procedure for the Ag-Mediated Substitution – GP5

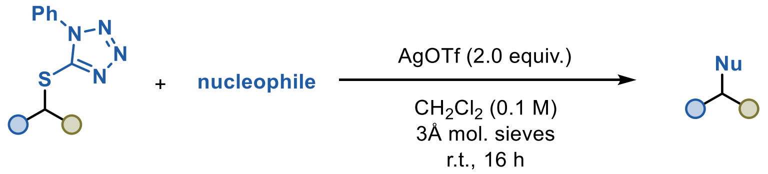

An oven-dried microwave vial equipped with a stirring bar was charged with the SPT sulfide (1.0 equiv.), the nucleophile (3.0 equiv.), AgOTf (2.0 equiv.) and 3Å molecular sieves (≈ 10 mg, activated by heating at 200 °C overnight). The vial was capped with a Supelco aluminium crimp seal with septum (PTFE/butyl), evacuated and refilled with Ar (x 3). Dry CH_2Cl_2 (0.1 M) was added, and the mixture was stirred for 16 h at r.t. The reaction was diluted with CH_2Cl_2 and filtered over silica, washing with

CH_2Cl_2. The volatiles were evaporated, and the crude was purified by flash column chromatography on silica gel to give the desired product.

General Procedure for the Deamination-Azidation Reaction – GP6

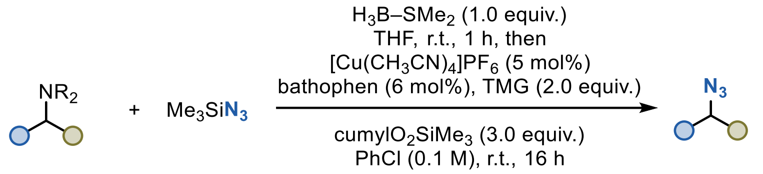

An oven-dried microwave vial equipped with a stirring bar was charged with the amine (1.0 equiv.), capped with a Supelco aluminium crimp seal with septum (PTFE/butyl), evacuated and refilled with Ar (x 3). Dry THF (0.1 M) was added, followed by $H_3B–SMe_2$ (1.0 equiv.). The mixture was stirred for 1 h at r.t. The volatiles were evaporated, and the vial was charged with $[Cu(CH_3CN)_4]PF_6$ (5.0 mol%) and **L8** (6.0 mol%). The vial was capped with a Supelco aluminium crimp seal with septum (PTFE/butyl), evacuated and refilled with Ar (x 3). Dry and degassed PhCl (0.1 M), TMG (2.0 equiv.) and Me_3SiN_3 (2.0 equiv.) were added. The mixture was stirred for 5 min and then cumylO_2SiMe_3 (3.0 equiv.) was slowly added. The reaction was stirred for 16 h at r.t. The volatiles were evaporated, and the crude was purified by flash column chromatography on silica gel to give the desired product.

General Procedure for the Deamination-Isothiocyanation Reaction – GP7

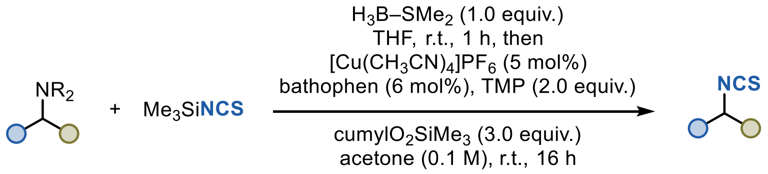

An oven-dried microwave vial equipped with a stirring bar was charged with the amine (1.0 equiv.), capped with a Supelco aluminium crimp seal with septum (PTFE/butyl), evacuated and refilled with Ar (x 3). Dry THF (0.1 M) was added, followed by $H_3B–SMe_2$ (1.0 equiv.). The mixture was stirred for 1 h at r.t. The volatiles were evaporated, and the vial was charged with $[Cu(CH_3CN)_4]PF_6$ (5.0 mol%) and **L8** (6.0 mol%). The vial was capped with a Supelco aluminium crimp seal with septum (PTFE/butyl), evacuated and refilled with Ar (x 3). Dry and degassed acetone (0.1 M), TMP (2.0 equiv.) and Me_3SiNCS (2.0 equiv.) were added. The mixture was stirred for 5 min and then cumylO_2SiMe_3 (3.0 equiv.) was slowly added. The reaction was stirred for 16 h at r.t. The volatiles were evaporated, and the crude was purified by flash column chromatography on silica gel to give the desired product.

General Procedure for the Transamination Reaction – GP8

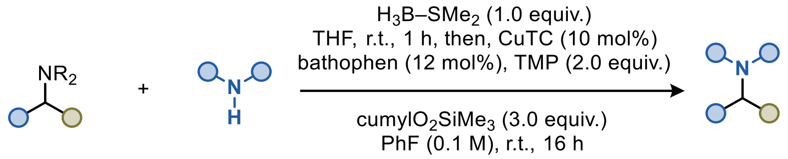

An oven-dried microwave vial equipped with a stirring bar was charged with the amine (1.0 equiv.), capped with a Supelco aluminium crimp seal with septum (PTFE/butyl), evacuated and refilled with Ar (x 3). Dry THF (0.1 M) was added, followed by $H_3B–SMe_2$ (1.0 equiv.). The mixture was stirred for 1 h at r.t. The volatiles were evaporated, and the vial was charged with CuTC (10 mol%), **L8** (12 mol%) and the N-nucleophile (1.5 equiv.). The vial was capped with a Supelco aluminium crimp seal with septum (PTFE/butyl), evacuated and refilled with Ar (x 3). Dry and degassed PhF (0.1 M) and TMP (2.0 equiv.) was added. The mixture was stirred for 5 min and then cumylO_2SiMe_3 (3.0 equiv.) was slowly added. The reaction was stirred for 2 h at r.t. The volatiles were evaporated, and the crude was purified by flash column chromatography on silica gel to give the desired product.

General Procedure for the Deamination-Hydroxylation Reaction – GP9

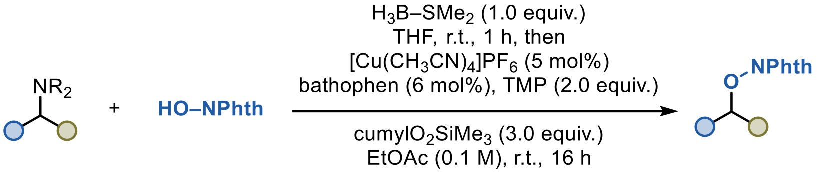

An oven-dried microwave vial equipped with a stirring bar was charged with the amine (1.0 equiv.), capped with a Supelco aluminium crimp seal with septum (PTFE/butyl), evacuated and refilled with Ar (x 3). Dry THF (0.1 M) was added, followed by $H_3B–SMe_2$ (1.0 equiv.). The mixture was stirred for 1 h at r.t. The volatiles were, evaporated and the vial was charged with $[Cu(CH_3CN)_4]PF_6$ (5.0 mol%), **L8** (6.0 mol%) and NHPI (2.0 equiv.). The vial was capped with a Supelco aluminium crimp seal with septum (PTFE/butyl), evacuated and refilled with Ar (x 3). Dry and degassed EtOAc (0.1 M) and TMP (2.0 equiv.) were added. The mixture was stirred for 5 min and then cumylO_2SiMe_3 (3.0 equiv.) was slowly added. The reaction was stirred for 16 h at r.t. The volatiles were evaporated and the crude was purified by flash column chromatography on silica gel to give the desired product.

General Procedure for the Deamination-Thiolation Reaction – GP10

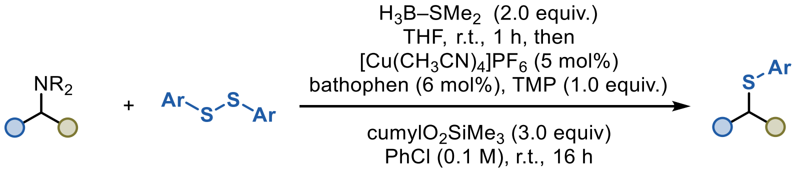

An oven-dried microwave vial equipped with a stirring bar was charged with the amine (2.0 equiv.), capped with a Supelco aluminium crimp seal with septum (PTFE/butyl), evacuated and refilled with Ar

(x 3). Dry THF (0.1 M) was added, followed by $H_3B–SMe_2$ (2.0 equiv.). The mixture was stirred for 1 h at r.t. The volatiles were evaporated, and the vial was charged with $[Cu(CH_3CN)_4]PF_6$ (5.0 mol%), **L8** (6.0 mol%) and the corresponding disulfide (1.0 equiv.). The vial was capped with a Supelco aluminium crimp seal with septum (PTFE/butyl), evacuated and refilled with Ar (x 3). Dry and degassed PhCl (0.1 M) and TMP (1.0 equiv.) were added. The mixture was stirred for 5 min and then cumylO_2SiMe_3 (3.0 equiv.) was slowly added. The reaction was stirred for 16 h at r.t. The volatiles were evaporated, and the crude was purified by flash column chromatography on silica gel to give the desired product.

General Procedure for the Deamination-Trifluoromethylthiolation Reaction – GP11

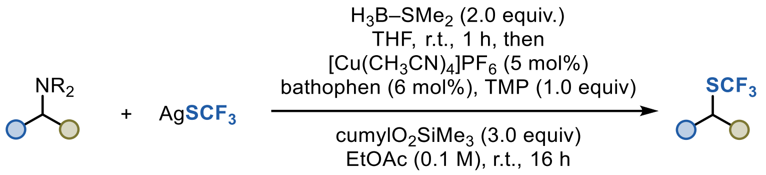

An oven-dried microwave vial equipped with a stirring bar was charged with the amine (2.0 equiv.), capped with a Supelco aluminium crimp seal with septum (PTFE/butyl), evacuated and refilled with Ar (x 3). Dry THF (0.1 M) was added, followed by $H_3B–SMe_2$ (2.0 equiv.). The mixture was stirred for 1 h at r.t. The volatiles were evaporated, and the vial was charged with $[Cu(CH_3CN)_4]PF_6$ (5.0 mol%), **L8** (6.0 mol%) and $AgSCF_3$ (1.0 equiv.). The vial was capped with a Supelco aluminium crimp seal with septum (PTFE/butyl), evacuated and refilled with Ar (x 3). Dry and degassed PhCl (0.1 M) and TMP (1.0 equiv.) were added. The mixture was stirred for 5 min and then cumylO_2SiMe_3 (3.0 equiv.) was slowly added. The reaction was stirred for 16 h at r.t. The volatiles were evaporated, and the crude was purified by flash column chromatography on silica gel to give the desired product.

General Procedure for the Deamination-Thiolation Reaction for SPT Sulfides – GP12

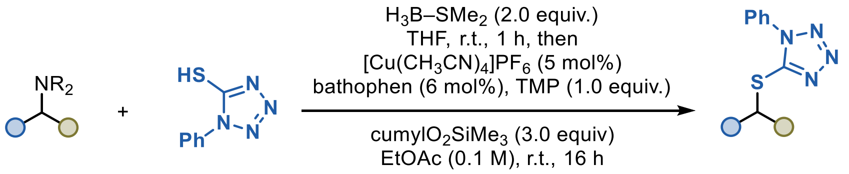

An oven-dried microwave vial equipped with a stirring bar was charged with the amine (2.0 equiv.), capped with a Supelco aluminium crimp seal with septum (PTFE/butyl), evacuated and refilled with Ar (x 3). Dry THF (0.1 M) was added, followed by $H_3B–SMe_2$ (2.0 equiv.). The mixture was stirred for 1 h at r.t. The volatiles were evaporated, and the vial was charged with $[Cu(CH_3CN)_4]PF_6$ (5.0 mol%), **L8** (6.0 mol%) and 1-phenyl-1H-tetrazole-5-thiol (1.0 equiv.). The vial was capped with a Supelco aluminium crimp seal with septum (PTFE/butyl), evacuated and refilled with Ar (x 3). Dry and degassed EtOAc (0.1 M) and TMP (1.0 equiv.) were added. The mixture was stirred for 5 min and then cumylO_2SiMe_3 (3.0 equiv.) was slowly added. The reaction was stirred for 16 h at r.t. The volatiles

were evaporated, and the crude was purified by flash column chromatography on silica gel to give the desired product.

General Procedure for the Deamidation-Thiolation Reaction – GP13

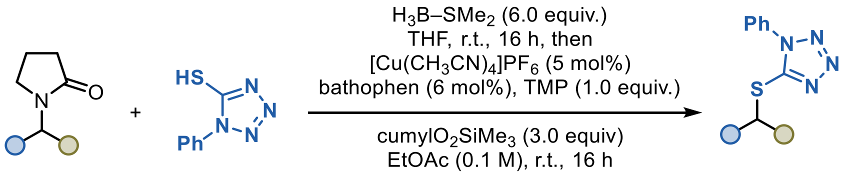

An oven-dried microwave vial equipped with a stirring bar was charged with the amide (2.0 equiv.), capped with a Supelco aluminium crimp seal with septum (PTFE/butyl), evacuated and refilled with Ar (x 3). Dry THF (0.1 M) was added, followed by $H_3B–SMe_2$ (6.0 equiv.). The mixture was stirred for 16 h at r.t. The volatiles were evaporated, and the vial was charged with $[Cu(CH_3CN)_4]PF_6$ (5.0 mol%), **L8** (6.0 mol%) and 1-phenyl-1H-tetrazole-5-thiol (1.0 equiv.). The vial was capped with a Supelco aluminium crimp seal with septum (PTFE/butyl), evacuated and refilled with Ar (x 3). Dry and degassed EtOAc (0.1 M) and TMP (1.0 equiv.) were added. The mixture was stirred for 5 min and then cumylO_2SiMe_3 (3.0 equiv.) was slowly added. The reaction was stirred for 16 h at r.t. The volatiles were evaporated, and the crude was purified by flash column chromatography on silica gel to give the desired product.

6 Substrate Scope

For every product in brackets are reported yield and borylated residual starting material (rsm).

1-Methoxy-4-(1-phenylethyl)benzene (2a)

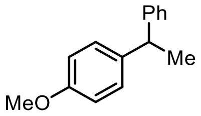

Following **GP1,** using $NaBH_4$ (8 mg, 2.0 equiv. 0.2 mmol), and aq. $NaHCO_3$ (1 M, 0.4 mL, 4.0 equiv.) instead of $H_3B–SMe_2$ and stirring for 16 h, **1a** (15 mg, 0.10 mmol, 1.0 equiv.) and Ph–$B(OH)_2$ (24 mg, 0.20 mmol, 2.0 equiv.) gave **2a** (50%) as an oil. ^{1}H NMR (400 MHz, $CDCl_3$) δ 7.29 (2H, t, *J* = 7.8 Hz), 7.24–7.16 (3H, m), 7.14 (2H, d, *J* = 8.7 Hz), 6.83 (2H, d, *J* = 8.7 Hz), 4.11 (1H, q, *J* = 7.3 Hz), 3.78 (3H, s), 1.62 (3H, d, *J* = 7.2 Hz); ^{13}C NMR (101 MHz, $CDCl_3$) δ 157.8, 146.8, 138.5, 128.5, 128.4, 127.4, 125.9, 113.6, 55.2, 43.9, 22.0. Data in accordance with literature.[2]

Following **GP1, 1b** (17 mg, 0.10 mmol, 1.0 equiv.) and Ph–$B(OH)_2$ (24 mg, 0.20 mmol, 2.0 equiv.) gave **2a** (62%) as an oil.

Following **GP1, 1c** (18 mg, 0.10 mmol, 1.0 equiv.) and Ph–$B(OH)_2$ (24 mg, 0.20 mmol, 2.0 equiv.) gave **2a** (56%) as an oil.

Following **GP1, 1d** (21 mg, 0.10 mmol, 1.0 equiv.) and Ph–$B(OH)_2$ (24 mg, 0.20 mmol, 2.0 equiv.) gave **2a** (80%) as an oil.

Following **GP1, 1e** (22 mg, 0.10 mmol, 1.0 equiv.) and Ph–$B(OH)_2$ (24 mg, 0.20 mmol, 2.0 equiv.) gave **2a** (70%) as an oil.

Following **GP1, 1f** (21 mg, 0.10 mmol, 1.0 equiv.) and Ph–$B(OH)_2$ (24 mg, 0.20 mmol, 2.0 equiv.) gave **2a** (49%) as an oil.

Diphenylmethane (2b)

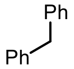

Following **GP2, 1g** (14 mg, 0.10 mmol, 1.0 equiv.) and PhB–$(OH)_2$ (24 mg, 0.20 mmol, 2.0 equiv.) gave **2b** (28%) as an oil. ^{1}H NMR (600 MHz, $CDCl_3$) 7.30–7.27 (4H, m), 7.22–7.17 (6H, m), 3.99 (2H, s); ^{13}C NMR (151 MHz, $CDCl_3$) δ 141.6, 129.1, 128.6, 126.2, 41.9. Data in accordance with literature.[3]

Ethane-1,1-diyldibenzene (2c)

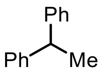

Following **GP1, 1h** (21 mg, 0.10 mmol, 1.0 equiv.) and Ph–$B(OH)_2$ (24 mg, 0.20 mmol, 2.0 equiv.) gave **2c** (50%) as an oil. ^{1}H NMR (600 MHz, $CDCl_3$) δ 7.30–7.27 (4H, m), 7.22 (4H, d, *J* = 7.3 Hz), 7.19–7.16 (2H, m), 4.16 (1H, q, *J* = 7.2 Hz), 1.64 (3H, d, *J* = 7.2 Hz,); ^{13}C NMR (151 MHz, $CDCl_3$) δ 146.5, 128.5, 127.8, 126.2, 44.9, 22.0. Data in accordance with literature.[4]

But-1-yne-1,3-diyldibenzene (2d)

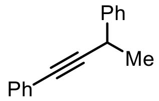

Following **GP1, 1i** (20 mg, 0.10 mmol, 1.0 equiv.) and Ph–B(OH)$_2$ (24 mg, 0.20 mmol, 2.0 equiv.) gave **2d** (45%) as an oil. ^{1}H NMR (400 MHz, CDCl$_3$) δ 7.51−7.44 (4H, m), 7.38 (2H, t, *J* = 7.6 Hz), 7.35−7.25 (4H, m), 4.01 (1H, q, *J* = 7.1 Hz), 1.62 (3H, d, *J* = 7.2 Hz); ^{13}C NMR (101 MHz, CDCl$_3$) δ 143.3, 131.6, 128.6, 128.2, 127.7, 126.9, 126.6, 123.8 92.7, 82.4, 32.5 24.5. Data in accordance with literature.[5]

But-1-yne-1,3-diyldibenzene (2e)

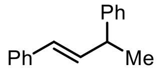

Following **GP2, 1j** (20 mg, 0.10 mmol, 1.0 equiv.) and Ph–B(OH)$_2$ (24 mg, 0.20 mmol, 2.0 equiv.) gave **2e** (11%) as an oil. ^{1}H NMR (400 MHz, CDCl$_3$) δ 7.38–7.28 (8H, m), 7.24–7.16 (2H, m), 6.40 (2H, d, *J* = 5.3 Hz), 3.75–3.55 (1H, m), 1.47 (3H, d, *J* = 7.0); ^{13}C NMR (101 MHz, CDCl$_3$) δ 145.8, 137.8, 135.4, 128.8, 128.7, 127.4, 127.2, 126.4, 126.4, 42.7, 21.3. Data in accordance with literature.[6]

Butane-1,3-diyldibenzene (2f)

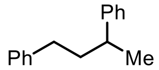

Following **GP1, 1k** (43 mg, 0.20 mmol, 2.0 equiv.) and Ph–B(OH)$_2$ (12 mg, 0.10 mmol, 1.0 equiv.) gave **2f** (35%, 96% rsm) as an oil. ^{1}H NMR (400 MHz, CDCl$_3$) δ 7.37–7.09 (10H, m), 2.73 (1H, h, *J* = 7.0 Hz), 2.60–2.44 (2H, m), 1.98–1.87 (2H, m), 1.28 (3H, d, *J* = 6.9 Hz);^{13}C NMR (101 MHz, CDCl$_3$) δ 147.5, 142.7, 128.5, 128.5, 128.4, 127.2, 126.1, 125.8, 40.1, 39.7, 34.1, 22.6. Data in accordance with literature.[7]

(5-Methylhexan-2-yl)benzene (2g)

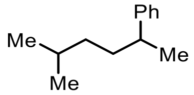

Following **GP1, 1l** (37 mg, 0.20 mmol, 2.0 equiv.) and Ph–B(OH)$_2$ (12 mg, 0.10 mmol, 1.0 equiv.) gave **2g** (36%, 90% rsm) as an oil. R_f 0.54 [pentane]; ^{1}H NMR (400 MHz, CDCl$_3$) δ 7.33–7.25 (2H, m), 7.21 – 7.13 (3H, m), 2.63 (1H, h, *J* = 7.1 Hz), 1.66–1.42 (4H, m), 1.23 (3H, d, *J* = 7.0 Hz), 1.21–1.13 (1H, m), 1.13 – 0.97 (1H, m), 0.84 (6H, dd, *J* = 6.6, 3.0 Hz);^{13}C NMR (101 MHz, CDCl$_3$) δ 148.2, 128.4, 127.1, 125.9, 40.4, 37.2, 36.3, 28.3, 22.8, 22.7, 22.5. HRMS (EI): Found M$^+$ 176,15782, $C_{13}H_{20}$ requires 176,15650.

Cyclopentylbenzene (2h)

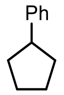

Following **GP1, 1m** (31 mg, 0.20 mmol, 2.0 equiv.) and Ph–B(OH)$_2$ (12 mg, 0.10 mmol, 1.0 equiv.) gave **2h** (27%, 90% rsm) as an oil. ^{1}H NMR (400 MHz, CDCl$_3$) δ 7.32–7.20 (4H, m), 7.16 (1H, t, *J* = 7.2 Hz), 2.98 (1H, p, *J* = 8.7 Hz), 2.12–1.98 (2H, m), 1.84–1.75 (2H, m), 1.71–1.52 (4H, m); ^{13}C NMR (101 MHz, CDCl$_3$) δ 146.6, 128.4, 127.2, 125.8, 46.1, 34.7, 25.7. Data in accordance with literature.[8]

Cyclohexylbenzene (2i)

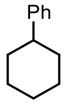

Following **GP1, 1n** (33 mg, 0.20 mmol, 2.0 equiv.) and Ph–B(OH)$_2$ (12 mg, 0.10 mmol, 1.0 equiv.) gave **2i** (37%, 80% rsm) as an oil. ^{1}H NMR (400 MHz, CDCl$_3$) δ 7.29 (2H, t, *J* = 7.5 Hz), 7.24–7.1 4 (3H, m), 2.55–2.45 (1H, m), 1.94–1.80 (4H, m), 1.80–1.70 (1H, m), 1.49–1.33 (4H, m), 1.33–1.19 (1H, m); ^{13}C NMR (101 MHz, CDCl$_3$) δ 148.3, 128.4, 127.0, 125.9, 44.8, 34.62, 27.1, 26.33. Data in accordance with literature.[9]

(4,4-Difluorocyclohexyl)benzene (2j)

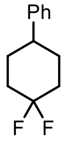

Following **GP1, 1o** (41 mg, 0.20 mmol, 2.0 equiv.) and Ph–B(OH)$_2$ (12 mg, 0.10 mmol, 1.0 equiv.) gave **2j** (34%, 40% rsm) as an oil. ^{1}H NMR (600 MHz, CDCl$_3$) δ 7.33–7.29 (2H, m), 7.24–7.20 (3H, m), 2.65–2.57 (1H, m), 2.24–2.17 (2H, m), 1.96–1.78 (6H, m); ^{13}C NMR (151 MHz, CDCl$_3$) δ 145.3, 128.7, 126.9, 126.6, 123.3 (t, *J* = 221.4 Hz), 42.7, 34.2 (t, *J* = 23.1 Hz), 30.4 (d, *J* = 9.8 Hz), ^{19}F NMR (564 MHz, CDCl$_3$) δ –91.59 (d, *J* = 236.4 Hz), –102.34 (dt, *J* = 236.4, 33.9 Hz). Data in accordance with literature.[10]

4-Phenyltetrahydro-2H-pyran (2k)

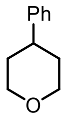

Following **GP1, 1p** (34 mg, 0.20 mmol, 2.0 equiv.) and Ph–B(OH)$_2$ (12 mg, 0.10 mmol, 1.0 equiv.) gave **2k** (37%, 39% rsm) as an oil. ^{1}H NMR (400 MHz, CDCl$_3$) δ 7.32 (2H, t, *J*=7.5 Hz), 7.25–7.19 (3H, m), 4.09 (2H, dd, *J* = 10.5, 3.3 Hz), 3.53 (2H, dd, *J* = 11.4, 2.1 Hz), 2.80–2.71 (1H, m), 1.87–1.74

(4H, m); ^{13}C NMR (101 MHz, $CDCl_3$) δ 146.0, 128.7, 126.9, 126.4, 68.6, 41.7, 34.1. Data in accordance with literature.[8]

Phenylcycloheptane (2l)

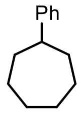

Following **GP1, 1q** (36 mg, 0.20 mmol, 2.0 equiv.) and Ph–$B(OH)_2$ (12 mg, 0.10 mmol, 1.0 equiv.) gave **2l** (35%, 56% rsm) as an oil. ^{1}H NMR (400 MHz, $CDCl_3$) δ 7.30–7.23 (2H, m), 7.21–7.11 (3H, m), 2.66 (1H, tt, J = 10.5, 3.6 Hz), 1.97–1.84 (2H, m), 1.84–1.75 (2H, m), 1.75–1.49 (8H, m); ^{13}C NMR (101 MHz, $CDCl_3$) δ 150.2, 128.4, 126.8, 125.6, 47.2, 37.0, 28.1, 27.4. Data in accordance with literature.[8]

Methyl 2,3-diphenylpropanoate (2m)

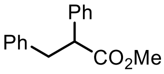

Following **GP1, 1r** (46 mg, 0.20 mmol, 2.0 equiv.) and Ph–$B(OH)_2$ (12 mg, 0.1 mmol, 1.0 equiv.) gave **2m** (45%) as an oil. ^{1}H NMR (400 MHz, $CDCl_3$) δ 7.32–7.20 (7H, m), 7.20–7.15 (1H, m), 7.12 (2H, d, J = 7.1 Hz), 3.85 (1H, dd, J = 8.8, 6.7 Hz), 3.60 (3H, s), 3.42 (1H, dd, J = 13.8, 8.8 Hz), 3.02 (1H, dd, J = 13.7, 6.7 Hz); ^{13}C NMR (101 MHz, $CDCl_3$) δ 174.0, 139.2, 138.8, 129.1, 128.8, 128.5, 128.1, 127.5, 126.5, 53.7, 52.1, 39.9. Data in accordance with literature.[11]

Benzyl 2,3-Diphenylpropanoate (2n)

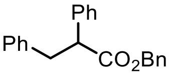

Following **GP1, 1s** (62 mg, 0.20 mmol, 2.0 equiv.) and Ph–$B(OH)_2$ (12 mg, 0.10 mmol, 1.0 equiv.) gave **2n** (40%, 17% rsm) as an oil. ^{1}H NMR (600 MHz, $CDCl_3$) δ 7.35–7.28 (4H, m), 7.28–7.24 (4H, m), 7.25–7.16 (3H, m), 7.19–7.07 (4H, m), 5.09 (1H, d, J = 12.5 Hz), 4.99 (1H, d, J = 12.5 Hz), 3.92 (1H, dd, J = 9.2, 6.5 Hz), 3.43 (1H, dd, J = 13.7, 9.2 Hz), 3.05 (1H, dd, J = 13.7, 6.5 Hz); ^{13}C NMR (151 MHz, $CDCl_3$) δ 173.3, 139.1, 138.7, 135.9, 129.1, 128.8, 128.6, 128.5, 128.1, 128.0, 127.6, 126.5, 66.6, 53.8, 39.9. Data in accordance with literature.[12]

N,N-Dimethyl-2,3-diphenylpropanamide (2o)

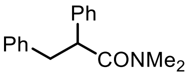

Following **GP1, 1t** (49 mg, 0.20 mmol, 2.0 equiv.) and Ph–$B(OH)_2$ (12 mg, 0.10 mmol, 1.0 equiv.) gave **2o** (45%) as a solid. ^{1}H NMR (600 MHz, $CDCl_3$) δ 7.31–7.20 (5H, m), 7.23–7.18 (2H, m), 7.15

(1H, t, J = 7.3 Hz), 7.07 (2H, d, J = 6.7 Hz), 3.97 (1H, dd, J = 7.9, 6.7 Hz), 3.47 (1H, dd, J = 13.6, 7.9 Hz), 2.94 (1H, dd, J = 13.6, 6.8 Hz), 2.90 (3H, s), 2.81 (3H, s); ^{13}C NMR (151 MHz, $CDCl_3$) δ 172.7, 140.3, 139.7, 129.3, 128.8, 128.3, 128.2, 127.1, 126.2, 51.4, 41.4, 37.3, 36.1. Data in accordance with literature.[13]

Methyl 3-(4-((*tert*-Butyldimethylsilyl)oxy)phenyl)-2-phenylpropanoate (2p)

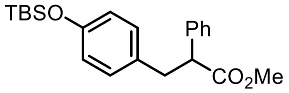

Following **GP1, 1u** (73 mg, 0.20 mmol, 2.0 equiv.) and Ph–B(OH)$_2$ (12 mg, 0.10 mmol, 1.0 equiv.) gave **2p** (49%) as an oil. R_f 0.35 [pentane:EtOAc (95:5)]; ^{1}H NMR (400 MHz, $CDCl_3$) δ 7.16–7.05 (5H, m), 6.78 (2H, d, J = 7.9 Hz), 6.54 (2H, d, J = 7.7 Hz), 3.63 (1H, dd, J = 8.9, 6.9 Hz), 3.43 (3H, s), 3.17 (1H, dd, J = 13.8, 8.7 Hz), 2.78 (1H, dd, J = 13.9, 6.7 Hz), 0.80 (9H, s), 0.00 (6H, s); ^{13}C NMR (101 MHz, $CDCl_3$) δ 174.1, 154.2, 138.8, 131.9, 130.0, 128.7, 128.1, 127.5, 120.0, 54.0, 52.1, 39.3, 25.8, 18.3, -4.3; HRMS (ESI): Found MNa$^+$ 393.18480, $C_{22}H_{30}O_3NaSi$ requires 393.18564.

Methyl 2-Phenylpropanoate (2q)

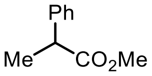

Following **GP1, 1v** (31 mg, 0.20 mmol, 2.0 equiv.) and Ph–B(OH)$_2$ (12 mg, 0.10 mmol, 1.0 equiv.) gave **2q** (40%, 22% rsm) as an oil. ^{1}H NMR (400 MHz, $CDCl_3$) δ; 7.38–7.26 (5H, m), 3.75 (1H, q, J = 7.3 Hz), 3.68 (3H, s), 1.52 (3H, d, J = 7.3 Hz); ^{13}C NMR (101 MHz, $CDCl_3$) δ 175.2, 140.7, 128.8, 127.6, 127.3, 52.2, 45.6, 18.7. Data in accordance with literature.[14]

4-(1-(4-Methoxyphenyl)ethyl)-1,1'-biphenyl (2s)

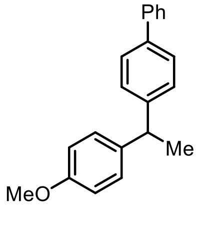

Following **GP1, 1d** (42 mg, 0.20 mmol, 1.0 equiv.) and *p*-biphenyl boronic acid (79 mg, 0.4 mmol, 2.0 equiv.) gave **2s** (56%, 7% rsm) as an oil. ^{1}H NMR (600 MHz, CDCl3) δ 7.67–7.15 (11H, m), 6.96–6.83 (2H, m), 4.19 (1H, q, J = 7.0 Hz), 3.81 (3H, s), 1.69 (3H, d, J = 7.0 Hz); ^{13}C NMR (151 MHz, $CDCl_3$) δ 158.0, 146.0, 141.1, 139.0, 138.5, 128.8, 128.6, 128.0, 127.2, 127.2, 127.1, 113.9, 55.4, 43.8, 22.2. Data in accordance with literature[15]

4,4'-(Ethane-1,1-diyl)bis(methoxybenzene) (2t)

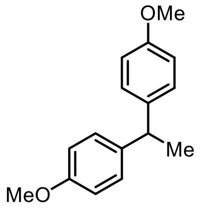

Following **GP2, 1d** (21 mg, 0.10 mmol, 1.0 equiv.) and *p*-anisyl boronic acid (30 mg, 0.20 mmol, 2.0 equiv.) gave **2t** (50%, 19% rsm) as an oil. ^{1}H NMR (400 MHz, $CDCl_3$) δ 7.15 (4H, d, *J* = 8.6 Hz, 4H), 6.85 (4H, d, *J* = 8.6 Hz), 4.06 (1H, q, *J* = 8.0 Hz), 3.77 (6H, s), 1.61 (3H, d, *J* = 8.0 Hz); ^{13}C NMR (101 MHz, $CDCl_3$) δ 157.8, 138.9, 128.3, 113.6, 55.1, 43.0, 22.1. Data in accordance with literature.[16]

1-fluoro-4-(1-(4-methoxyphenyl)ethyl)benzene (2u)

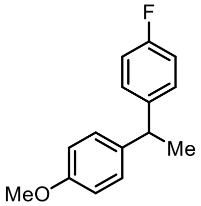

Following **GP1, 1d** (21 mg, 0.10 mmol, 1.0 equiv.) and *p*-fluorophenyl boronic acid (28 mg, 0.20 mmol, 2.0 equiv.) gave **2u** (73%, 12% rsm) as an oil. ^{1}H NMR (400 MHz, $CDCl_3$) δ 7.15–7.05 (4H, m), 6.91 (2H, t, *J* = 8.5 Hz), 6.80 (2H, d, *J* = 8.5 Hz), 4.05 (1H, q, *J* = 7.2 Hz), 3.75 (s, 3H), 1.56 (3H, d, *J* = 7.2 Hz); ^{13}C NMR (101 MHz, $CDCl_3$) δ 161.2 (d, *J* = 243.7 Hz), 157.9, 142.4 (d, *J* = 3.1 Hz), 138.3, 128.9 (d, *J* = 7.8 Hz), 128.4, 115.0 (d, *J* = 21.0 Hz), 113.8, 55.2, 43.2, 22.2. Data in accordance with literature.[17]

1-Methoxy-4-(1-(4-(trifluoromethyl)phenyl)ethyl)benzene (2v)

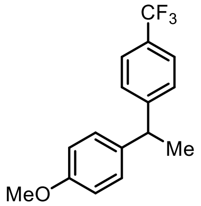

Following **GP1, 1d** (21 mg, 0.10 mmol, 1.0 equiv.) and *p*-trifluoromethylphenyl boronic acid (38 mg, 0.20 mmol, 2.0 equiv.) gave **2v** (62%) as an oil. ^{1}H NMR (400 MHz, $CDCl_3$) δ 7.55 (2H, d, *J* = 8.1 Hz), 7.33 (2H, d, *J* = 8.5 Hz), 7.15 (2H, d, *J* = 8.4 Hz), 6.87 (2H, d, *J* = 8.7 Hz), 4.18 (1H, d, *J* = 7.2 Hz), 3.80 (3H, s), 1.65 (3H, d, *J* = 7.3 Hz); ^{13}C NMR (101 MHz, $CDCl_3$) δ 158.1, 150.9, 137.4, 128.5, 128.3 (d, *J* = 32.3 Hz), 127.8, 125.3 (q, *J* = 3.8 Hz), 124.3 (d, *J* = 272.7 Hz), 113.9, 55.2,43.8, 21.8. Data in accordance with literature.[18]

1-Methoxy-3-(1-(4-methoxyphenyl)ethyl)benzene (2w)

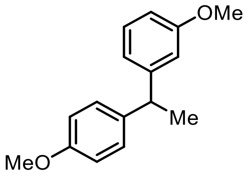

Following **GP1, 1d** (21 mg, 0.10 mmol, 1.0 equiv.) and *m*-anisyl boronic acid (30 mg, 0.20 mmol, 2.0 equiv.) gave **2w** (54%, 11% rsm) as an oil. ^{1}H NMR (400 MHz, $CDCl_3$) δ 7.23 (1H, t, *J* = 7.9 Hz), 7.18 (2H, d, *J* = 8.7 Hz), 6.87−6.84 (3H, m), 6.81−6.80 (1H, m), 6.76 (1H, dd, *J* = 8.1, 2.6 Hz), 4.11 (1H, q, *J* = 7.2 Hz), 3.82−3.78(m, 6H), 1.64 (3H, d, *J* = 7.2 Hz); ^{13}C NMR (101 MHz, $CDCl_3$) δ 159.6, 157.9, 148.6, 138.3, 129.3, 128.5, 120.1, 113.7, 113.7, 110.9, 55.3, 55.2, 44.0, 22.0. Data in accordance with literature.[18]

1-(1-(4-Methoxyphenyl)ethyl)-3-(trifluoromethyl)benzene (2x)

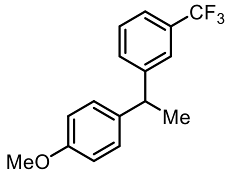

Following **GP1, 1d** (21 mg, 0.10 mmol, 1.0 equiv.) and *m*-trifluoromethylphenyl boronic acid (38 mg, 0.20 mmol, 2.0 equiv.) gave **2x** (53%, 17% rsm) as an oil. R_f 0.50 [pentane:EtOAc (99:1)]; ^{1}H NMR (600 MHz, $CDCl_3$) δ 7.47 (1H, s), 7.46–7.41 (1H, m), 7.41–7.34 (2H, m), 7.12 (2H, d, *J* = 8.6 Hz), 6.84 (2H, d, *J* = 8.7 Hz), 4.16 (1H, q, *J* = 7.2 Hz), 3.79 (3H, s), 1.63 (3H, d, *J* = 7.2 Hz); ^{13}C NMR (151 MHz, $CDCl_3$) f 158.2, 147.9, 137.6, 131.2, 130.7 (q, *J* = 31.9 Hz), 128.9, 128.6, 125.3 (q, *J* = 274.1 Hz), 124.3 (q, *J* = 3.8 Hz), 123.0 (q, *J* = 3.8 Hz), 114.1, 55.4, 44.0, 22.1; ^{19}F NMR (565 MHz, $CDCl_3$) δ –62.50; HRMS (EI): Found M^+ 280.1066, $C_{16}H_{15}F_3O$ requires 280.1075.

1-(1-(4-Methoxyphenyl)ethyl)-2-methylbenzene (2y)

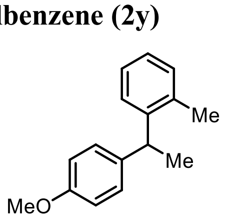

Following **GP1, 1d** (21 mg, 0.10 mmol, 1.0 equiv.) and *o*-tolyl boronic acid (27 mg, 0.20 mmol, 2.0 equiv.) gave **2y** (44%, 16% rsm) as an oil. ^{1}H NMR (400 MHz, $CDCl_3$) δ 7.25 (1H, s), 7.22–7.14 (1H, m), 7.14 (2H, d, *J* = 4.0 Hz), 7.07 (2H, d, *J* = 8.6 Hz), 6.80 (2H, d, *J* = 8.6 Hz), 4.28 (1H, q, *J* = 7.2 Hz), 3.77 (3H, s), 2.25 (3H, s), 1.59 (3H, d, *J* = 7.2 Hz); ^{13}C NMR (101 MHz, $CDCl_3$) δ 157.7, 144.3, 138.3, 136.0, 130.4, 128.6, 126.5, 126.0, 126.0, 113.6, 55.2, 40.1, 22.2, 19.7. Data in accordance with literature.[18]

1-Methoxy-2-(1-(4-methoxyphenyl)ethyl)benzene (2z)

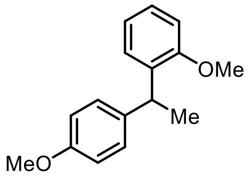

Following **GP1,** using acetone as solvent, **1d** (21 mg, 0.10 mmol, 1.0 equiv.) and *o*-anisyl boronic acid (30 mg, 0.20 mmol, 2.0 equiv.) gave **2z** (30%, 26% rsm) as an oil. ^{1}H NMR (400 MHz, $CDCl_3$) δ 7.23–7.11 (4H, m), 6.92 (1H, dd, *J* = 10.7, 4.2 Hz), 6.89–6.80 (3H, m), 4.56 (1H, q, *J* = 7.2 Hz), 3.81 (3H, s), 3.79 (3H, s), 1.58 (3H, d, *J* = 7.2 Hz); ^{13}C NMR (101 MHz, $CDCl_3$) δ 157.5, 156.7, 138.5, 135.3, 128.6, 127.6, 127.0, 120.5, 113.5, 110.6, 55.5, 55.2, 36.5, 21.1. Data in accordance with literature.[19]

1,3-Difluoro-2-(1-(4-methoxyphenyl)ethyl)benzene (2aa)

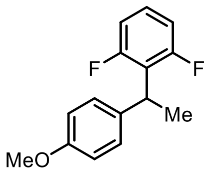

Following **GP1, 1d** (21 mg, 0.10 mmol, 1.0 equiv.) and *o*-difluorophenyl boronic acid (31 mg, 0.20 mmol, 2.0 equiv.) gave **2aa** (58%, 12% rsm) as an oil. R_f 0.50 [pentane:EtOAc (99:1)]; ^{1}H NMR (600 MHz, $CDCl_3$) δ 7.25 (2H, d, *J* = 8.5 Hz), 7.12 (1H, tt, *J* = 8.3, 6.3 Hz), 6.83 (2H, d, *J* = 8.7 Hz), 6.86–6.80 (2H, m), 4.56 (1H, q, *J* = 7.4 Hz), 3.78 (3H, s), 1.72 (3H, d, *J* = 7.4 Hz); ^{13}C NMR (151 MHz, $CDCl_3$) δ 161.4 (dd, *J* = 247.1, 9.0 Hz), 158.1, 136.1, 128.3, 127.7 (t, *J* = 10.6 Hz), 122.2 (t, *J* = 17.7 Hz), 113.8, 112.7–110.5 (m), 55.3, 33.4 (t, *J* = 2.4 Hz), 18.9 (t, *J* = 3.0 Hz); ^{19}F NMR (565 MHz, $CDCl_3$) δ –113.50 (t, *J* = 6.9 Hz); HRMS (EI): Found M^+ 248.1008, $C_{15}H_{14}F_2O$ requires 248.1013.

***tert*-Butyl 3-(1-(4-methoxyphenyl)ethyl)-1H-indole-1-carboxylate (2ab)**

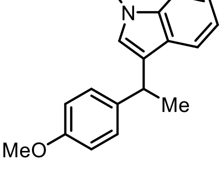

Following **GP1, 1d** (21 mg, 0.10 mmol, 1.0 equiv.) and 1-boc-3-indoleboronic acid (52 mg, 0.20 mmol, 2.0 equiv.) gave **2ab** (42%, 14% rsm) as an oil. R_f 0.30 [pentane:EtOAc (99:1)]. ^{1}H NMR (400 MHz, $CDCl_3$) δ 8.07 (1H, s), 7.44 (1H, s), 7.24 (2H, m), 7.18 (2H, d, *J* = 8.7 Hz), 7.09 (1H, t, *J* = 7.5 Hz), 6.81 (2H, d, *J* = 8.7 Hz), 4.22 (1H, q, *J* = 7.0 Hz), 3.77 (3H, s), 1.68 (9H, s), 1.66 (3H, d, *J* = 7.0 Hz); ^{13}C NMR (151 MHz, $CDCl_3$) δ 158.1, 137.8, 130.3, 128.9, 128.4, 125.9, 124.3, 122.4 (2C), 120.1, 115.3, 114.0, 113.3, 83.6, 55.4, 36.1, 28.4, 22.3; HRMS (ESI): Found MNa^+ 359.15773, $C_{17}H_{24}O_5N_2Na$ requires 359.15774.

5-(1-(4-Methoxyphenyl)ethyl)benzofuran (2ac)

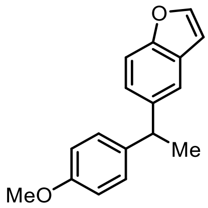

Following **GP1, 1d** (42 mg, 0.20 mmol, 1.0 equiv.) and 5-benzofuranboronic acid (72 mg, 0.4 mmol, 2.0 equiv.) gave **2ac** (56%, 19% rsm) as an oil. R_f 0.63 [hexane:EtOAc (90:10)]; ^{1}H NMR (600 MHz, $CDCl_3$) δ 7.58 (1H, d, *J* = 2.2 Hz), 7.42 (1H, d, *J* = 1.9 Hz), 7.39 (1H, d, *J* = 8.5 Hz), 7.15 (2H, d, *J* = 8.7 Hz), 7.13 (1H, dd, *J* = 8.6, 1.9 Hz), 6.82 (2H, d, *J* = 8.8 Hz), 6.70 (1H, dd, *J* = 2.2, 1.0 Hz), 4.20 (1H, t, *J* = 7.2 Hz), 3.77 (3H, s), 1.65 (3H, d, *J* = 7.2 Hz); ^{13}C NMR (151 MHz, $CDCl_3$) δ 157.9, 153.7, 145.3, 141.6, 139.1, 128.6, 127.5, 124.5, 119.6, 113.9, 111.2, 106.7, 55.4, 43.9, 22.7; HRMS (EI): Found M^+ 252.11453, $C_{17}H_{16}O_2$ requires 252.11448.

2-(1-(4-Methoxyphenyl)ethyl)furan (2ad)

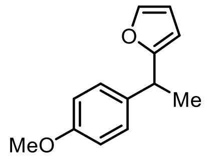

Following **GP1, 1d** (21 mg, 0.10 mmol, 1.0 equiv.) and 2-furanboronic acid (22 mg, 0.20 mmol, 2.0 equiv.) gave **2ad** (41%) as an oil. R_f 0.48 [pentane:EtOAc (80:20)]; ^{1}H NMR (600 MHz, $CDCl_3$) δ 7.30 (1H, dd, *J* = 1.9, 0.9 Hz), 7.13 (2H, d, *J* = 8.6 Hz), 6.85 (2H, d, *J* = 8.7 Hz), 6.29 (1H, dd, *J* = 3.2, 1.9 Hz), 6.02 (1H, dt, *J* = 3.3, 1.0 Hz), 4.08 (1H, q, *J* = 7.2 Hz), 3.79 (3H, s), 1.57 (3H, d, *J* = 7.2 Hz); ^{13}C NMR (151 MHz, $CDCl_3$) δ 159.5, 158.4, 141.4, 136.5, 128.4, 114.0, 110.1, 104.8, 55.4, 38.6, 20.8; HRMS (EI): Found M^+ 202.0989, $C_{13}H_{14}O_2$ requires 202.0994.

3-(1-(4-Methoxyphenyl)ethyl)furan (2ae)

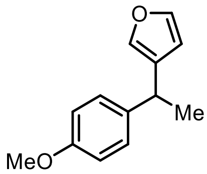

Following **GP1, 1d** (42 mg, 0.20 mmol, 1.0 equiv.) and 3-furanboronic acid (44 mg, 0.4 mmol, 2.0 equiv.) gave **2ae** (50%, 8% rsm) as an oil. R_f 0.50 [hexane:EtOAc (95:5)]; ^{1}H NMR (600 MHz, $CDCl_3$) δ 7.34 (1H, t, *J* = 1.6 Hz), 7.20–7.17 (1H, m), 7.14 (2H, d, *J* = 8.6 Hz), 6.84 (2H, d, *J* = 8.6 Hz), 6.21–6.18 (1H, m), 3.91 (1H, q, *J* = 7.2 Hz), 3.79 (3H, s), 1.52 (3H, d, *J* = 7.1 Hz); ^{13}C NMR (151 MHz, $CDCl_3$) δ 158.0, 142.9, 138.6, 138.2, 130.7, 128.2, 113.7, 110.5, 55.2, 35.6, 22.2; HRMS (EI): Found M^+ 202.09883, $C_{13}H_{14}O_2$ requires 202.09883.

2-(1-(4-Methoxyphenyl)ethyl)thiophene (2af)

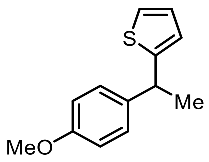

Following **GP1, 1d** (42 mg, 0.20 mmol, 1.0 equiv.) and 2-thiophenylboronic acid (51 mg, 0.4 mmol, 2.0 equiv.) gave **2af** (43%, 11% rsm) as an oil. ^{1}H NMR (600 MHz, $CDCl_3$) δ 7.18 (2H, d, *J* = 8.7 Hz), 7.13 (1H, dd, *J* = 5.2, 1.1 Hz), 6.91 (1H, dd, *J* = 5.1, 3.5 Hz,), 6.85 (2H, d, *J* = 8.7 Hz,), 6.81–6.76 (1H, m), 4.30 (1H, q, *J* = 7.2 Hz), 3.79 (3H, s), 1.68 (3H, d, *J* = 7.2 Hz); ^{13}C NMR (151 MHz, $CDCl_3$) δ 158.3, 151.5, 138.4, 128.4, 126.6, 123.6, 123.5, 114.0, 55.4, 40.1, 23.6. Data in accordance with literature.[20]

3-(1-(4-Methoxyphenyl)ethyl)thiophene (2ag)

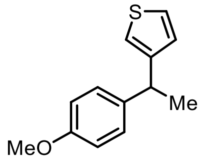

Following **GP1, 1d** (21 mg, 0.10 mmol, 1.0 equiv.) and 3-thiophenylboronic acid (25 mg, 0.20 mmol, 2.0 equiv.) gave **2ag** (41%, 8% rsm) as an oil. R_f 0.38 [pentane:EtOAc (90:10)]; ^{1}H NMR (400 MHz, $CDCl_3$) δ 7.22 (1H, dd, *J* = 4.9, 2.9 Hz), 7.12 (2H, d, *J* = 8.7 Hz), 6.97–6.91 (1H, m), 6.87 (1H, dd, *J* = 5.0, 1.3 Hz), 6.83 (2H, d, *J* = 8.8 Hz), 4.11 (1H, q, *J* = 7.2 Hz), 3.78 (3H, s), 1.60 (4H, d, *J* = 7.2 Hz); ^{13}C NMR (151 MHz, $CDCl_3$) δ 158.1, 147.8, 138.6, 128.5, 128.0, 125.5, 119.8, 113.9, 55.4, 40.1, 22.5; HRMS (EI): Found M^+ 218.0760, $C_{13}H_{12}OS$ requires 218.0765.

4-(1-(4-Methoxyphenyl)ethyl)isoxazole (2ah)

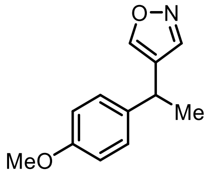

Following **GP1, 1d** (21 mg, 0.10 mmol, 1.0 equiv.) and isoxazol-4-ylboronic acid (23 mg, 0.20 mmol, 2.0 equiv.) gave **2ah** (59%, 10% rsm) as an oil. R_f 0.33 [pentane:EtOAc (95:5)]; ^{1}H NMR (400 MHz, $CDCl_3$) δ 8.14 (1H, s), 8.05 (1H, s), 7.11 (2H, d, *J* = 8.6 Hz), 6.86 (2H, d, *J* = 8.6 Hz), 3.96 (1H, q, *J* = 7.3 Hz), 3.80 (3H, s), 1.57 (3H, d, *J* = 7.2 Hz); ^{13}C NMR (101 MHz, $CDCl_3$) δ 158.4, 154.0, 149.7, 136.7, 128.1, 125.3, 114.1, 55.3, 33.5, 22.4; HRMS (EI): Found M^+ 203.0941, $C_{12}H_{13}O_2N$ requires 203.0946.

2-Fluoro-5-(1-(4-methoxyphenyl)ethyl)pyridine (2ai)

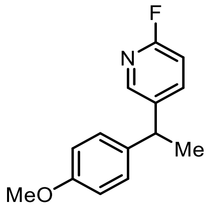

Following **GP1, 1d** (21 mg, 0.10 mmol, 1.0 equiv.) and 2-fluoropyridylboronic acid (24 mg, 0.20 mmol, 2.0 equiv.) gave **2ai** (57%, 10% rsm) as an oil. R_f 0.27 [pentane:EtOAc (98:2)]; ^{1}H NMR (400 MHz, $CDCl_3$) δ 8.09 (1H, d, *J* = 2.6 Hz), 7.55 (1H, td, *J* = 8.1, 2.6 Hz), 7.10 (2H, d, *J* = 8.7 Hz), 6.89–6.79 (1H, m), 6.85 (2H, d, *J* = 8.6 Hz), 4.13 (1H, q, *J* = 7.2 Hz), 3.79 (3H, s), 1.62 (3H, d, *J* = 7.4 Hz); ^{13}C NMR (101 MHz, $CDCl_3$) δ 162.4 (d, *J* = 237.8 Hz), 158.4, 146.4 (d, *J* = 14.4 Hz), 140.4 (d, *J* = 7.8 Hz), 139.8 (d, *J* = 4.5 Hz), 137.0, 128.5, 114.2, 109.3 (d, *J* = 37.4 Hz), 55.4, 40.9, 22.0; ^{19}F NMR (126 MHz, $CDCl_3$) δ –71.97; HRMS (ESI): Found MNa^+ 254.09547, $C_{14}H_{14}FNO$ requires 254.09516.

2-Methoxy-5-(1-(4-methoxyphenyl)ethyl)pyridine (2aj)

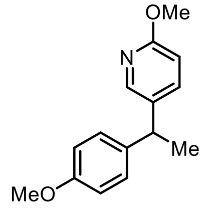

Following **GP1, 1d** (42 mg, 0.20 mmol, 1.0 equiv.) and 2-methoxypyridylboronic acid (61 mg, 0.4 mmol, 2.0 equiv.) gave **2aj** (60%, 12% rsm) as an oil. R_f 0.33 [hexane:EtOAc (85:15)]; ^{1}H NMR (600 MHz, $CDCl_3$) δ 8.04 (1H, d, *J* = 2.6 Hz), 7.36 (1H, dd, *J* = 8.5, 2.5 Hz), 7.11 (2H, d, *J* = 8.3 Hz), 6.83 (2H, d, *J* = 8.7 Hz), 6.65 (1H, d, *J* = 8.5 Hz), 4.05 (1H, q, *J* = 7.4 Hz), 3.91 (3H, s), 3.78 (3H, s), 1.59 (3H, d, *J* = 7.1 Hz); ^{13}C NMR (151 MHz, $CDCl_3$) δ 162.8, 158.1, 145.3, 138.3, 138.0, 134.9, 128.5, 114.0, 110.7, 55.4, 53.5, 40.9, 22.1; HRMS (ESI): Found MH^+ 244.13337, $C_{15}H_{18}O_2N$ requires 244.13321.

2-Chloro-5-(1-(4-methoxyphenyl)ethyl)pyrimidine (2ak)

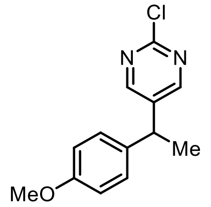

Following **GP1, 1d** (21 mg, 0.10 mmol, 1.0 equiv.) and (2-chloropyrimidin-5-yl)boronic acid (32 mg, 0.20 mmol, 2.0 equiv.) gave **2ak** (36%) as an oil. R_f 0.43 [pentane:EtOAc (95:5)]; ^{1}H NMR (400 MHz, $CDCl_3$) δ 8.45 (2H, s), 7.09 (2H, d, *J* = 8.6 Hz), 6.87 (2H, d, *J* = 8.8 Hz), 4.11 (1H, q, *J* = 7.3 Hz), 3.79 (3H, s), 1.66 (3H, d, *J* = 7.3 Hz); ^{13}C NMR (101 MHz, $CDCl_3$) δ 159.4, 158.9, 158.8, 138.3, 135.3,

128.5, 114.5, 55.5, 39.2, 21.5; HRMS (ESI): Found MNa^+ 271.0609, $C_{13}H_{13}ON_2ClNa$ requires 271.0609.

(E)-1-Methoxy-4-(4-(p-tolyl)but-3-en-2-yl)benzene (2al)

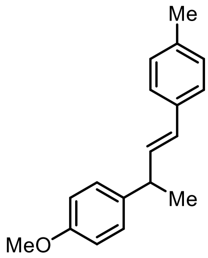

Following **GP1, 1d** (21 mg, 0.10 mmol, 1.0 equiv.) and (E)-(4-methylstyryl)boronic acid (32 mg, 0.20 mmol, 2.0 equiv.) gave **2al** (36%) as an oil. R_f 0.43 [pentane:EtOAc (99:1)]; ^{1}H NMR (600 MHz, $CDCl_3$) δ 7.24 (2H, d, *J* = 8.1 Hz), 7.18 (2H, d, *J* = 8.6 Hz), 7.09 (2H, d, *J* = 7.8 Hz), 6.86 (2H, d, *J* = 8.7 Hz), 6.36 (1H, d, *J* = 16.0 Hz), 6.30 (1H, dd, *J* = 15.9, 6.3 Hz), 3.79 (3H, s), 3.58 (1H, p, *J* = 6.8 Hz), 2.32 (3H, s), 1.43 (3H, d, *J* = 7.0 Hz); ^{13}C NMR (151 MHz, $CDCl_3$) δ 158.1, 138.1, 136.9, 135.0, 134.7, 129.3, 128.4, 128.2, 126.2, 114.0, 55.4, 41.8, 21.5, 21.3; HRMS (EI): Found M^+ 252.1510, $C_{18}H_{20}O$ requires 252.1514.

1-(1-Cyclopropylethyl)-4-methoxybenzene (2am)

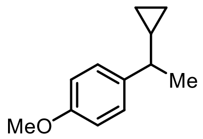

Following **GP1, 1d** (21 mg, 0.10 mmol, 1.0 equiv.) and cyclopropylboronic acid (17 mg, 0.20 mmol, 2.0 equiv.) gave **2am** (45%) as an oil. ^{1}H NMR (400 MHz, $CDCl_3$) δ 7.18 (2H, d, *J* = 8.6 Hz), 6.85 (2H, d, *J* = 8.7 Hz), 3.80 (3H, s), 2.01–1.88 (1H, m), 1.31 (3H, d, *J* = 7.0 Hz), 0.90 (1H, qt, *J* = 9.0, 4.9 Hz), 0.58–0.49 (1H, m), 0.46–0.36 (1H, m), 0.23–0.09 (2H, m); ^{13}C NMR (101 MHz, $CDCl_3$) δ 157.9, 139.7, 128.0, 113.8, 55.4, 43.8, 21.8, 18.9, 4.7, 4.4. Data in accordance with literature.[21]

2-(4-Methoxyphenyl)propanenitrile (3a)

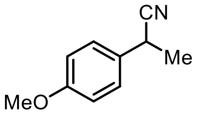

Following **GP2, 1d** (21 mg, 0.10 mmol, 1.0 equiv.) and Me_3SiCN (27 µL, 0.20 mmol, 2.0 equiv.) gave **3a** (50%) as an oil. ^{1}H NMR (600 MHz, $CDCl_3$) δ 7.27 (2H, d, *J* = 7.7 Hz), 6.91 (2H, d, *J* = 8.7 Hz), 3.85 (1H, q, *J* = 7.3 Hz), 3.81 (3H, s), 1.62 (3H, d, *J* = 7.3 Hz); ^{13}C NMR (151 MHz, $CDCl_3$) δ 159.3, 129.2, 128.0, 122.0, 114.6, 55.5, 30.5, 21.6. Data in accordance with literature.[22]

2-(4-Methoxyphenyl)propanenitrile (3b)

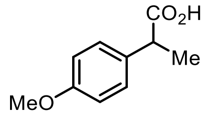

A microwave vial equipped with a stirring bar was charged with **3a** (0.1 mmol). H_2O (0.5 mL) and AcOH (0.5 mL) were added and then H_2SO_4 (100 μL) was added dropwise. The reaction was stirred for 12 h at 80 °C. Upon reaction completion (TLC analysis), the mixture was diluted with EtOAc and H_2O. The layers were separated, and the aqueous layer was extracted with EtOAc (x 2). The combined organic layers were washed with brine (x 2), dried ($MgSO_4$) and filtered. The solvent was evaporated, and the residue was purified by column chromatography on silica gel to give **3b** (90%) as a solid. ^1H NMR (400 MHz, $CDCl_3$) δ 7.23 (2H, d, *J* = 8.6 Hz), 6.85 (2H, d, *J* = 8.7 Hz), 3.78 (3H, s), 3.69 (1H, q, *J* = 7.2 Hz), 1.48 (3H, d, *J* = 7.2 Hz); ^{13}C NMR (101 MHz, $CDCl_3$) δ 181.0, 159.1, 132.1, 128.8, 114.1, 55.5, 44.6, 18.3. Data in accordance with literature.[23]

1-(*sec*-Butyl)-4-methoxybenzene (3c)

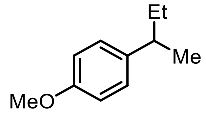

Following **GP3, 1d** (21 mg, 0.10 mmol, 1.0 equiv.) and Et_2Zn (0.15 mL, 1.0 M in hexane, 0.15 mmol, 1.5 equiv.) gave **3c** (27%, 38% rsm) as a liquid. ^1H NMR (400 MHz, $CDCl_3$) δ 7.10 (2H, d, *J* = 8.5 Hz), 6.84 (2H, d, *J* = 8.6 Hz), 3.79 (3H, s), 2.60–2.50 (1H, m), 1.21 (2H, d, *J* = 6.9 Hz), 0.81 (2H, t, *J* = 7.4 Hz); ^{13}C NMR (151 MHz, $CDCl_3$) δ 157.6, 139.7, 127.8, 113.6, 55.3, 40.8, 31.2, 22.0, 12.2. Data in accordance with literature.[24]

1-Methoxy-4-(4-phenylbut-3-yn-2-yl)benzene (3d)

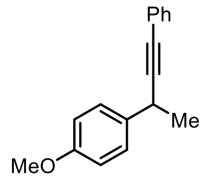

Following **GP4, 1d** (21 mg, 0.10 mmol, 1.0 equiv.) and phenylacetylene (22 μL, 0.20 mmol, 2.0 equiv.) gave **3d** (36%, 48% rsm) as a liquid. ^1H NMR (400 MHz, $CDCl_3$) δ 7.47–7.40 (m, 2H), 7.37 (d, *J* = 8.7 Hz, 2H), 7.31–7.27 (m, 3H), 6.89 (d, *J* = 8.8 Hz, 2H), 3.94 (q, *J* = 7.1 Hz, 1H), 3.81 (s, 3H), 1.55 (d, *J* = 7.1 Hz, 3H); ^{13}C NMR (101 MHz, $CDCl_3$) δ 158.5, 135.7, 131.8, 128.3, 128.0, 127.9, 123.9, 114.1, 93.1, 82.4, 55.5, 31.8, 24.7. Data in accordance with literature.[25]

Tetrahydro-2H-pyran-4-carbonitrile (3e)

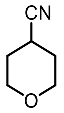

Following **GP2, 1p** (34 mg, 0.20 mmol, 2.0 equiv.) and Me_3SiCN (14 µL, 0.10 mmol, 1.0 equiv.) gave **3e** (16%, 98% rsm) as an oil. ^{1}H NMR (400 MHz, $CDCl_3$) δ 3.89 (2H, ddd, *J* = 12.1, 6.4, 3.6 Hz), 3.59 (2H, ddd, *J* = 11.6, 7.7, 3.3 Hz), 2.86 (1H, tt, *J* = 8.2, 4.3 Hz), 1.98 – 1.90 (3H, m), 1.90 – 1.81 (2H, m); ^{13}C NMR (101 MHz, $CDCl_3$) δ 121.3, 65.8, 29.1, 25.5. Data in accordance with literature.[26]

2,5-Dimethylhexanenitrile (3f)

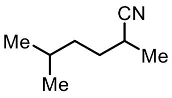

Following **GP2, 1l** (37 mg, 0.20 mmol, 2.0 equiv.) and Me_3SiCN (14 µL, 0.10 mmol, 1.0 equiv.) gave **3f** (26%, 58% rsm) as an oil. R_f 0.78 [pentane:EtOAc (95:5)]; ^{1}H NMR (400 MHz, $CDCl_3$) δ 2.63–2.50 (1H, m), 1.68–1.48 (3H, m), 1.47–1.28 (2H, m), 1.32 (3H, d, *J* = 7.1 Hz), 0.91 (6H, dd, *J* = 6.6, 2.4 Hz); ^{13}C NMR (101 MHz, $CDCl_3$) δ 123.3, 77.5, 76.8, 36.2, 32.1, 27.9, 25.9, 22.7, 22.4, 18.21. HRMS (ESI): Found MNa^+ 148.10895, $C_8H_{15}NNa$ requires 148.10967. Due to volatility of the product NMR spectra contain residual EtOAc.

1,3-Diphenylbutan-1-one (3g)

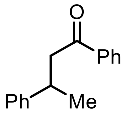

Following **GP5**, **6d** (28 mg, 0.10 mmol, 1.0 equiv.) and 1-phenyl-1-trimethylsilyloxyethylene (62 µL 0.30 mmol, 3.0 equiv.) gave **3g** (83%) as an oil. ^{1}H NMR (600 MHz, $CDCl_3$) δ 7.93 (2H, d, *J* = 7.3 Hz), 7.57–7.53 (1H, m), 7.44 (2H, t, *J* = 7.8 Hz), 7.32–7.26 (4H, m), 7.20 (1H, tt, *J* = 7.0, 1.7 Hz), 3.50 (1H, dt, *J* = 13.9, 6.9 Hz), 3.30 (1H, dd, *J* = 16.4, 5.7 Hz), 3.19 (1H, dd, *J* = 16.4, 8.3 Hz), 1.34 (3H, d, *J* = 6.9 Hz); ^{13}C NMR (151 MHz, $CDCl_3$) δ 199.2, 146.7, 137.4, 133.1, 128.7, 128.7, 128.2, 127.0, 126.4, 47.2, 35.7, 22.0. Data in accordance with literature.[27]

2-(1-Phenylethyl)cyclohexan-1-one (3h)

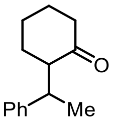

Following **GP5**, **6d** (28 mg, 0.10 mmol, 1.0 equiv.) and 1-(trimethylsiloxy)cyclohexene (58 µL 0.30 mmol, 3.0 equiv.) gave **3h** (79%) as a mixture of diastereomers, as an oil. dr = 2:1. ^{1}H NMR (400 MHz, $CDCl_{3,}$ diastereomers) δ 7.32–7.27 (2H, m), 7.22–7.14 (3H, m), 3.47-3.40 & 3.19–3.05 (1H, m), 2.57–

2.18 (3H, m), 2.05–1.84 (2H, m), 1.84–1.68 (2H, m), 1.66–1.51 (2H, m), 1.25 & 1.22 (3H, d, *J* = 6.6 Hz); ^{13}C NMR (101 MHz, $CDCl_3$, diastereomers) δ 213.8, 212.2, 146.2, 145.1, 128.6, 128.4, 127.9, 127.7, 126.4, 126.1, 57.8, 57.2, 42.4, 42.3, 39.4, 37.3, 32.7, 28.9, 28.6, 27.9, 24.8, 24.1, 21.2, 16.3. Data in accordance with literature.[28]

2,2-Dimethyl-3-phenylbutanal (3i)

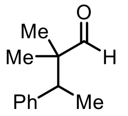

Following **GP5**, **6d** (28 mg, 0.10 mmol, 1.0 equiv.) and 2-methyl-1-(trimethylsilyloxy)-1-propene (53 µL 0.30 mmol, 3.0 equiv.) gave **3i** (70%) as an oil. ^{1}H NMR (400 MHz, $CDCl_3$) δ 9.55 (1H, s), 7.38–7.13 (5H, m), 3.02 (1H, q, *J* = 7.2 Hz), 1.27 (3H, d, *J* = 7.2 Hz), 1.04 (3H, s), 0.93 (3H, s); ^{13}C NMR (101 MHz, $CDCl_3$) δ 206.6, 142.0, 129.1, 128.2, 126.9, 49.5, 44.8, 20.9, 17.9, 16.0. Data in accordance with literature.[29]

(1*S*,4*R*)-1-(3,4-Dichlorophenyl)-4-phenyl-1,2,3,4-tetrahydronaphthalene (3ja) & (1*S*,4*S*)-1-(3,4-Dichlorophenyl)-4-phenyl-1,2,3,4-tetrahydronaphthalene (3jb)

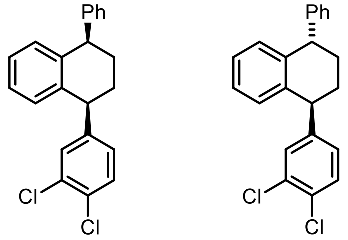

Following **GP1, 1x** (31 mg, 0.10 mmol, 1.0 equiv.) and Ph–$B(OH)_2$ (24 mg, 0.20 mmol, 2.0 equiv.) gave **3j** (49%) as an unseparable mixture of diastereomers, as an oil. dr = 2:1. R_f 0.31 [pentane:EtOAc (98:2)]. Pure samples of each diastereomer for characterization purposes was obtained by prep-HPLC.
Data for the major diastereomer: ^{1}H NMR (400 MHz, $CDCl_3$) δ 7.36 (1H, d, *J* = 8.3 Hz), 7.31 (2H, t, *J* = 8.1 Hz), 7.25–7.20 (2H, m), 7.12 (2H, d, *J* = 6.1 Hz), 7.10–7.04 (2H, m), 6.97 (1H, dd, *J* = 8.2, 2.1 Hz), 6.93–6.79 (2H, m), 4.23 (1H, br t, *J* = 6.5 Hz), 4.22 (1H, br t, *J* = 6.6 Hz), 2.29–2.15 (2H, m), 1.95–1.77 (2H, m); ^{13}C NMR (101 MHz, $CDCl_3$) δ 147.9, 147.2, 140.2, 138.8, 132.5, 130.8, 130.4, 130.3, 130.2, 129.9, 128.9, 128.5, 128.4, 126.6, 126.5, 126.4, 46.0, 45.5, 31.4, 31.3; HRMS (EI): Found M^+ 352.07805, $C_{22}H_{18}Cl_2$ requires 352.07801
Data for the minor diastereomer: ^{1}H NMR (400 MHz, $CDCl_3$) δ 7.40–7.20 (5H, m), 7.15–7.09 (4H, m), 7.00–6.96 (1H, m), 6.94–6.87 (1H, m), 4.24–4.14 (2H, m), 2.15–2.02 (2H, m), 1.98–1.84 (2H, m); ^{13}C NMR (101 MHz, $CDCl_3$) δ 147.8, 147.1, 139.8, 138.5, 132.4, 130.9, 130.7, 130.4, 130.2, 129.9, 128.9, 128.5, 128.3, 126.9, 126.6, 126.3, 45.5, 45.0, 29.9, 29.9; HRMS (EI): Found M^+ 352.07801, $C_{22}H_{18}Cl_2$ requires 352.07801.

5-((1S,4S)-4-(3,4-Dichlorophenyl)-1,2,3,4-tetrahydronaphthalen-1-yl)-2-methoxypyridine (3ka) & 5-((1R,4S)-4-(3,4-Dichlorophenyl)-1,2,3,4-tetrahydronaphthalen-1-yl)-2-methoxypyridine (3kb)

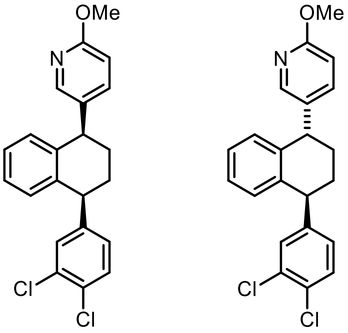

Following **GP1, 1x** (31 mg, 0.10 mmol, 1.0 equiv.) and 2-methoxypyridylboronic acid (30 mg, 0.2 mmol, 2.0 equiv.) gave **3k** (32%) as a mixture of diastereomers, as an oil. dr = 2.6:1. Data for the major diastereomer: R_f 0.40 [pentane:EtOAc (85:15)]; ^{1}H NMR (600 MHz, $CDCl_3$) δ 7.97 (1H, d, *J* = 2.5 Hz), 7.37 (1H, d, *J* = 8.2 Hz), 7.28–7.26 (1H, m), 7.22 (1H, d, *J* = 2.1 Hz), 7.09–7.07 (2H, m), 6.96–6.95 (1H, m), 6.88–6.86 (1H, m), 6.85–6.82 (1H, m), 6.69 (1H, d, *J* = 8.5 Hz), 4.23–4.18 (2H, m), 3.94 (3H, s), 2.25–2.15 (2H, m), 1.86–1.80 (2H, m); ^{13}C NMR (101 MHz, $CDCl_3$) δ 163.1, 147.7, 146.8, 138.9, 138.8, 135.0, 132.5, 130.8, 130.5, 130.3, 130.3, 130.0, 130.0, 128.3, 126.8, 126.7, 111.0, 53.5, 45.4, 42.5, 31.4, 31.2; HRMS (ESI): Found MH^+ 384.09211, $C_{22}H_{20}NCl_2$ requires 384.09165.

(1S,4S)-4-(3,4-Dichlorophenyl)-1,2,3,4-tetrahydronaphthalene-1-carbonitrile (3la) & (1R,4S)-4-(3,4-Dichlorophenyl)-1,2,3,4-tetrahydronaphthalene-1-carbonitrile (3lb)

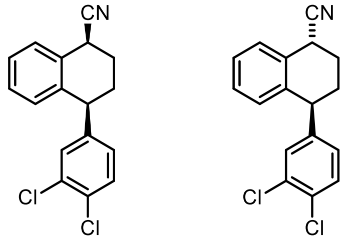

Following **GP2, 1n** (31 mg, 0.10 mmol, 1.0 equiv.) and Me_3SiCN (27 μL, 0.20 mmol, 2.0 equiv.) gave **3l** (56%) as a mixture of diastereomers, as an oil. dr = 1.6:1. R_f 0.44 [pentane:EtOAc (90:10)].

Data for the major diastereomer: ^{1}H NMR (400 MHz, $CDCl_3$) δ 7.48 (1H, d, *J* = 7.7 Hz), 7.36 (1H, d, *J* = 8.3 Hz), 7.29 (1H, t, *J* = 7.3 Hz), 7.23 (1H, t, *J* = 7.4 Hz), 7.12 (1H, d, *J* = 2.1 Hz), 6.88 (2H, dd, *J* = 8.3, 2.1 Hz), 4.17 (1H, t, *J* = 6.7 Hz), 4.13 (1H, t, *J* = 6.6 Hz), 2.38 (1H, dddd, *J* = 13.9, 8.5, 5.8, 2.7 Hz), 2.26 (1H, dddd, *J* = 13.8, 8.4, 5.4, 2.7 Hz), 2.10 (1H, dddd, *J* = 13.5, 9.4, 7.7, 2.7 Hz), 1.86 (1H, dddd, *J* = 13.6, 9.5, 7.6, 2.6 Hz); ^{13}C NMR (101 MHz, $CDCl_3$) δ 146.3, 137.6, 132.8, 130.8, 130.8, 130.7, 130.6, 130.5, 129.0, 128.7, 128.2, 127.8, 121.6, 44.0, 31.3, 30.8, 25.2; HRMS (ESI): Found MNa^+ 324.03210, $C_{17}H_{13}NCl_2Na$ requires 324.03173.

Data for the minor diastereomer: R_f 0.41 [pentane:EtOAc (90:10)]; ^{1}H NMR (400 MHz, $CDCl_3$) δ 7.42 (1H, d, *J* = 7.7 Hz), 7.37 (1H, d, *J* = 8.3 Hz), 7.32–7.25 (1H, m), 7.25–7.17 (2H, m), 6.94–6.85

(2H, m), 4.16 – 3.98 (2H, m), 2.33–2.15 (2H, m), 2.17–2.03 (2H, m); ^{13}C NMR (101 MHz, $CDCl_3$) δ 146.0, 137.7, 132.6, 130.7, 130.6 (2C), 130.6, 130.5, 129.1, 128.6, 128.1, 127.6, 121.4, 44.1, 30.9, 30.2, 24.9; HRMS (ESI): Found MNa^+ 324.03212, $C_{17}H_{13}NCl_2Na$ requires 324.03173.

3-(1-Cyanoethyl)phenyl Ethyl(methyl)carbamate (3m)

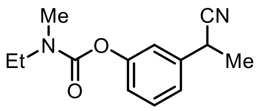

Following **GP2, 1y** (50 mg, 0.20 mmol, 2.0 equiv.) and Me_3SiCN (27 μL, 0.2 mmol, 2.0 equiv.) gave **3m** (63%) as an oil. R_f 0.28 [pentane:EtOAc 80:20)]; ^{1}H NMR (400 MHz, $CDCl_3$, rotamers) δ 7.39 (1H, t, J = 7.9 Hz), 7.21 (1H, d, J = 7.8 Hz), 7.19–7.09 (2H, m), 3.92 (1H, q, J = 7.3 Hz), 3.50 & 3.44 (2H, q, J = 7.0 Hz), 3.09 & 3.02 (3H, s), 1.67 (3H, d, J = 7.3 Hz), 1.26 & 1.21 (3H, t, J = 6.4 Hz); ^{13}C NMR (101 MHz, $CDCl_3$, rotamers) δ 152.0, 138.2, 130.0, 123.4, 123.4, 121.6, 121.3, 120.3, 44.1, 34.3, 33.9, 31.1, 21.3, 13.2, 12.5; HRMS (ESI): Found MNa^+ 255.11080, $C_{13}H_{16}O_2N_2Na$ requires 255.11040.

3-(1-Phenylethyl)phenyl Ethyl(methyl)carbamate (3n)

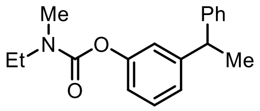

Following **GP1, 1y** (25 mg, 0.10 mmol, 1.0 equiv.) and Ph–$B(OH)_2$ (24 mg, 0.20 mmol, 2.0 equiv.) gave **3n** (51%) as an oil. ^{1}H NMR (400 MHz, $CDCl_3$, rotamers) δ 7.31–7.16 (6H, m), 7.03 (1H, d, J = 7.7 Hz), 7.00–6.91 (2H, m), 4.15 (1H, q, J = 7.2 Hz), 3.45 & 3.39 (2H, q, J = 7.1 Hz), 3.04 & 2.99 (3H, s), 1.63 (3H, d, J = 7.2 Hz), 1.23 & 1.18 (3H, t, J = 6.9 Hz); ^{13}C NMR (101 MHz, $CDCl_3$, rotamers) δ 154.7, 151.7, 147.9, 146.2, 129.2, 128.5, 127.8, 126.2, 124.6, 121.1, 119.6, 44.7, 44.2, 34.3, 33.9, 21.9, 13.4, 12.6. Data in accordance with literature.[30]

3-(1-(2-Chloropyrimidin-5-yl)ethyl)phenyl Ethyl(methyl)carbamate (3o)

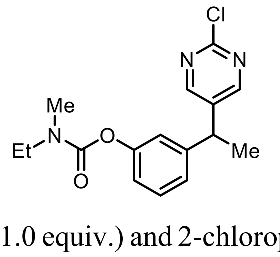

Following **GP1, 1y** (25 mg, 0.10 mmol, 1.0 equiv.) and 2-chloropyrimidine-5-boronic acid (32 mg, 0.20 mmol, 2.0 equiv.) gave **3o** (34%, 5% rsm) as an oil. R_f 0.18 [pentane:EtOAc (85:15)]; ^{1}H NMR (400 MHz, $CDCl_3$, rotamers) δ 8.47 (2H, s), 7.32 (1H, t, J = 7.9 Hz), 7.04–6.98 (2H, m), 6.95 (1H, s), 4.16 (1H, q, J = 7.3 Hz), 3.46 & 3.40 (2H, q, J = 7.2 Hz), 3.06 & 2.98 (3H, s), 1.68 (3H, d, J = 7.2 Hz), 1.23 & 1.19 (3H, t, J = 7.1 Hz); ^{13}C NMR (101 MHz, $CDCl_3$, rotamers) δ 159.6, 159.0, 154.5, 154.3, 152.1,

144.5, 137.6, 129.9, 124.2, 121.0, 120.8, 44.3, 39.8, 34.4, 34.0, 21.3, 13.4, 12.6; HRMS (ESI): Found MNa^+ 342.09874, $C_{16}H_{18}O_2N_3ClNa$ requires 342.09798.

1-(3,3-Diphenylpropoxy)naphthalene (3p)

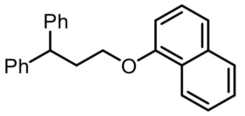

Following **GP1, 1z** (31 mg, 0.10 mmol, 1.0 equiv.) and Ph–B(OH)$_2$ (24 mg, 0.20 mmol, 2.0 equiv.) gave **3p** (69%) as an oil. R_f 0.78 [pentane:EtOAc (90:10)]; ^{1}H NMR (600 MHz, $CDCl_3$) δ 8.31–8.25 (1H, m), 7.83–7.77 (1H, m), 7.54–7.45 (2H, m), 7.40 (1H, d, J = 8.2 Hz), 7.35–7.27 (9H, m), 7.23–7.16 (2H, m), 6.67 (1H, d, J = 7.6 Hz), 4.40 (1H, t, J = 7.8 Hz), 4.08 (2H, t, J = 6.2 Hz), 2.69 (2H, dt, J = 7.8, 6.2 Hz); ^{13}C NMR (151 MHz, $CDCl_3$) δ 154.7, 144.5 (2C), 134.6, 128.7, 128.1, 127.6, 126.5, 126.0, 125.8, 125.3, 122.2, 120.2, 104.7, 66.1, 47.7, 35.2; HRMS (ESI): Found M^+ 338.16647, $C_{25}H_{22}O$ requires 338.16652.

2-Fluoro-5-(3-(naphthalen-1-yloxy)-1-phenylpropyl)pyridine (3q)

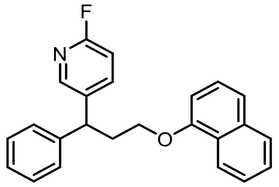

Following **GP1, 1z** (31 mg, 0.10 mmol, 1.0 equiv.) and 2-fluoropyridine-5-boronic acid (28 mg, 0.2 mmol, 2.0 equiv.) gave **3q** (39%) as an oil. R_f 0.30 [pentane:EtOAc (85:15)]; ^{1}H NMR (600 MHz, $CDCl_3$) δ 8.24 (1H, d, J = 7.1 Hz), 8.19 (1H, br s), 7.81 (1H, dd, J = 6.8, 2.2 Hz), 7.67 (1H, td, J = 8.1, 2.6 Hz), 7.52–7.48 (2H, m), 7.42 (1H, d, J = 8.2 Hz), 7.34–7.30 (3H, m), 7.29–7.23 (3H, m), 6.86 (1H, dd, J = 8.5, 3.0 Hz), 6.68 (1H, d, J = 7.6 Hz), 4.46 (1H, t, J = 7.9 Hz), 4.09 (2H, t, J = 6.0 Hz), 2.74–2.61 (2H, m); ^{13}C NMR (151 MHz, $CDCl_3$) δ 162.6 (d, J = 237.9 Hz), 154.5, 146.9 (d, J = 14.5 Hz), 142.9, 140.8 (d, J = 7.9 Hz), 137.7 (d, J = 4.5 Hz), 134.7, 129.1, 128.0, 127.75, 127.1, 126.65, 126.0, 125.7, 125.4, 121.9, 120.6, 109.6 (d, J = 37.5 Hz), 104.8, 65.5, 44.5, 35.0; ^{19}F NMR (565 MHz, $CDCl_3$) δ –71.20; HRMS (ESI): Found MNa^+ 380.14293, $C_{27}H_{20}ONFNa$ requires 380.14211.

1-(3-(Cyclohex-1-en-1-yl)-3-phenylpropoxy)naphthalene (3r)

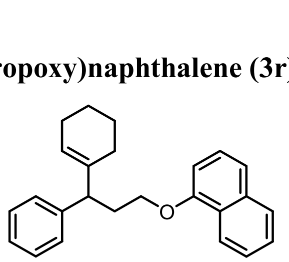

Following **GP1, 1z** (31 mg, 0.10 mmol, 1.0 equiv.) and 1-cyclohexenylboronic acid (25 mg, 0.2 mmol, 2.0 equiv.) gave **3r** (36%, 21% rsm) as an oil. R_f 0.70 [pentane:EtOAc (98:2)]; ^{1}H NMR (400 MHz,

$CDCl_3$) δ 8.33–8.25 (1H, m), 7.83–7.75 (1H, m), 7.52–7.43 (2H, m), 7.40 (1H, d, *J* = 8.2 Hz), 7.36–7.18 (6H, m), 6.72 (1H, d, *J* = 7.5 Hz), 5.72 (1H, br s), 4.10 (1H, dt, *J* = 9.4, 6.2 Hz), 4.02 (1H, dt, *J* = 9.3, 6.6 Hz), 3.55 (1H, t, *J* = 7.8 Hz), 2.53–2.43 (1H, m), 2.35–2.25 (1H, m), 2.11–2.04 (2H, m), 1.88–1.77 (2H, m), 1.57–1.51 (4H, m); ^{13}C NMR (101 MHz, $CDCl_3$) δ 154.9, 143.8, 139.6, 134.6, 128.4, 128.1, 127.6, 126.5, 126.3, 126.0, 125.9, 125.2, 122.2, 121.9, 120.1, 104.7, 66.4, 49.3, 32.5, 27.1, 25.5, 23.2, 22.7; HRMS (EI): Found M^+ 342.19777, $C_{25}H_{26}O$ requires 342.19782.

4-(Naphthalen-1-yloxy)-2-phenylbutanenitrile (3s)

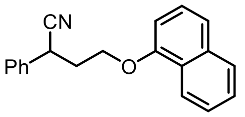

Following **GP1, 1z** (31 mg, 0.10 mmol, 1.0 equiv.) and Me_3SiCN (27 μL, 0.2 mmol, 2.0 equiv.) gave **3s** (68%) as an oil. R_f 0.29 [pentane:EtOAc (98:2)]; ^{1}H NMR (400 MHz, $CDCl_3$) δ 8.33–8.24 (1H, m), 7.88–7.79 (1H, m), 7.58–7.51 (2H, m), 7.48 (1H, d, *J* = 8.3 Hz), 7.45–7.32 (6H, m), 6.77 (1H, d, *J* = 7.6 Hz), 4.36–4.26 (2H, m), 4.14 (1H, ddd, *J* = 9.9, 6.7, 4.4 Hz), 2.61 (1H, dddd, *J* = 14.6, 8.2, 6.7, 4.4 Hz), 2.47 (1H, dtd, *J* = 14.2, 7.2, 4.4 Hz); ^{13}C NMR (101 MHz, $CDCl_3$) δ 154.1, 135.1, 134.7, 129.4, 128.5, 127.7, 127.6, 126.7, 125.9, 125.65, 125.6, 121.9, 121.0, 120.78, 104.9, 64.2, 35.7, 34.3; HRMS (ESI): Found MNa^+ 310.1212, $C_{20}H_{17}ONNa$ requires 310.1202.

2-(1-Phenylethyl)cyclopentan-1-one (3t)

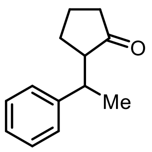

Following **GP5**, **6d** (28 mg, 0.10 mmol, 1.0 equiv.) and 1-(trimethylsiloxy)cyclopentene (53 μL 0.30 mmol, 3.0 equiv.) gave **3t** (62%) as a mixture of diastereomers, as an oil. dr = 1.3:1. ^{1}H NMR (400 MHz, $CDCl_3$, diastereomers) δ 7.35–7.15 (5H, m), 3.48–3.40 & 3.28–3.20 (1H, m), 2.43–2.20 (2H, m), 2.12–1.63 (5H, m), 1.44 & 1.22 (3H, d, *J* = 7.1 Hz); ^{13}C NMR (101 MHz, $CDCl_3$, diastereomers) δ 220.4, 220.1, 145.4, 144.1, 128.4, 128.2, 128.0, 127.3, 126.3, 126.2, 55.9, 55.3, 39.1 (2C), 38.9, 37.9, 26.6, 24.6, 20.5, 20.4, 20.0, 15.5. Data in accordance with literature.[29]

1-(1-Azidoethyl)-4-methoxybenzene (4a)

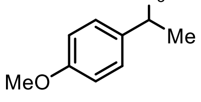

Following **GP6, 1d** (21 mg, 0.10 mmol, 1.0 equiv.) and Me_3SiN_3 (26 μL, 0.20 mmol, 2.0 equiv.) gave **4a** (38%, 30% rsm) as a liquid. ^{1}H NMR (600 MHz, $CDCl_3$) δ 7.25 (d, *J* = 8.7 Hz, 2H), 6.91 (d, *J* = 8.5

Hz, 2H), 4.57 (q, J = 6.8 Hz, 1H), 3.82 (s, 3H), 1.51 (d, J = 6.8 Hz, 3H); ^{13}C NMR (151 MHz, $CDCl_3$) δ 159.6, 133.0, 127.8, 114.2, 60.9, 55.5, 21.6. Data in accordance with literature.[31]

1-(1-Isothiocyanatoethyl)-4-methoxybenzene (4b)

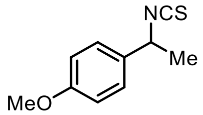

Following **GP7, 1d** (21 mg, 0.10 mmol, 1.0 equiv.) and Me_3SiNCS (28 μL, 0.20 mmol, 2.0 equiv.) gave **4a** (41%, 29% rsm) as an oil. ^{1}H NMR (400 MHz, $CDCl_3$) δ 7.24 (2H, d, J = 9.1 Hz), 6.90 (2H, d, J = 8.5 Hz), 4.86 (1H, q, J = 6.8 Hz), 3.81 (3H, s), 1.65 (3H, d, J = 6.8 Hz); ^{13}C NMR (101 MHz, $CDCl_3$) δ 159.6, 132.5, 132.1, 126.9, 114.4, 56.7, 55.5, 25.0. Data in accordance with literature.[32]

3-Chloro-1-(1-(4-methoxyphenyl)ethyl)-1H-indazole (4c)

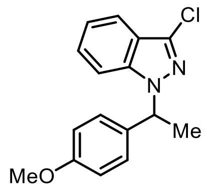

Following **GP8, 1d** (21 mg, 0.10 mmol, 1.0 equiv.) and 3-chloro indazole (23 mg, 0.15 mmol, 1.5 equiv.) gave **4c** (58%) as an oil. R_f 0.72 [pentane:EtOAc (90:10)]; ^{1}H NMR (600 MHz, $CDCl_3$) δ 7.65 (1H, d, J = 8.2 Hz), 7.34–7.29 (1H, m), 7.25 (1H, d, J = 8.6 Hz), 7.21 (2H, d, J = 8.6 Hz), 7.15 (1H, t, J = 7.5 Hz), 6.82 (2H, d, J = 8.6 Hz), 5.72 (1H, q, J = 7.0 Hz), 3.76 (3H, s), 1.99 (3H, d, J = 7.1 Hz); ^{13}C NMR (101 MHz, $CDCl_3$) δ 159.1, 140.3, 133.7 (2C), 127.5, 127.2, 121.5, 121.2, 119.8, 114.0, 110.0, 58.2, 55.3, 21.2; HRMS (ESI): Found MNa^+ 309.07567, $C_{16}H_{15}ON_2ClNa$ requires 309.07651.

2-(1-(4-Methoxyphenyl)ethyl)isoindoline-1,3-dione (4d)

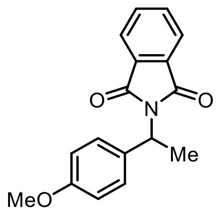

Following **GP8, 1d** (21 mg, 0.10 mmol, 1.0 equiv.) and phthalimide (22 mg, 0.15 mmol, 1.5 equiv.) gave **4d** (41%) as an oil. ^{1}H NMR (400 MHz, $CDCl_3$) δ 7.79–7.77 (2H, m), 7.66–7.65 (2H, m), 7.45 (2H, J = 8.3 Hz), 6.85 (2H, d, J = 8.4 Hz), 5.52 (1H, q, J = 7.4 Hz), 3.76 (3H, s), 1.90 (3H, d, J = 7.3 Hz); ^{13}C NMR (101 MHz, $CDCl_3$) δ 168.1, 159.0, 133.8, 132.4, 132.0, 128.8, 123.0, 113.7, 55.2, 49.1, 17.6. Data in accordance with literature.[33]

3-Chloro-1-(tetrahydro-2H-pyran-4-yl)-1H-indazole (4e)

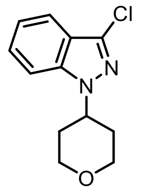

Following **GP8, 1p** (34mg, 0.20 mmol, 2.0 equiv.) and 3-chloro indazole (15 mg, 0.1 mmol, 1.0 equiv.) gave **4e** (22%) as an oil. ^{1}H NMR (600 MHz, $CDCl_3$) δ 7.68 (1H, d, J = 8.2 Hz), 7.47 – 7.37 (2H, m), 7.21 (1H, ddd, J = 8.0, 5.8, 1.9 Hz), 4.59 (1H, td, J = 11.6, 5.8 Hz), 4.17 (4H, dd, J = 11.8, 4.5 Hz), 3.60 (3H, t, J = 11.7 Hz), 2.43 (2H, dd, J = 12.7, 4.4 Hz), 2.37 (2H, dd, J = 12.1, 4.6 Hz), 1.97 (3H, d, J = 12.1 Hz); ^{13}C NMR (101 MHz, $CDCl_3$) δ 140.1, 133.0 127.5, 121.5, 121.4, 120.2, 109.3, 67.3, 55.8, 32.5. Data in accordance with literature.[34]

3-Chloro-1-(5-methylhexan-2-yl)-1H-indazole (4f)

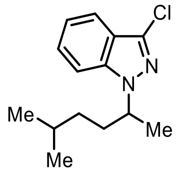

Following **GP8, 1l** (21 mg, 0.20 mmol, 2.0 equiv.) and 3-chloro indazole (15 mg, 0.1 mmol, 1.0 equiv.) gave **4f** (24%, 30% rsm) as an oil. R_f 0.58 [pentane:EtOAc (95:5)]; ^{1}H NMR (400 MHz, $CDCl_3$) δ) 7.67 (1H, d, J = 8.2 Hz), 7.45 – 7.35 (2H, m), 7.18 (1H, ddd, J=7.9, 4.7, 2.9 Hz), 4.54 (1H, dq, J = 8.7, 6.6 Hz), 2.07 (1H, dddd, J = 13.6, 11.1, 8.7, 4.9 Hz), 1.84 (1H, ddt, J = 13.7, 11.0, 5.4 Hz), 1.55 (3H, d, J = 6.7 Hz), 1.53 – 1.45 (1H, m), 1.13 (1H, dddd, J = 13.4, 11.6, 6.7, 5.3 Hz), 0.96 (1H, dddd, J = 13.2, 11.4, 6.8, 4.9 Hz), 0.82 (6H, dd, J = 6.6, 2.5 Hz); ^{13}C NMR (101 MHz, $CDCl_3$) δ 140.7, 132.5, 127.2, 121.1, 121.0, 119.9, 109.5, 55.8, 36.8, 34.4, 28.0, 22.7, 22.6, 21.0. HRMS (EI): Found M^+ 250.12313, $C_{14}H_{19}ClN_2$ requires 250.12368.

N-(1-Phenylethyl)benzamide (4g)

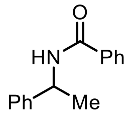

Following **GP5**, **6d** (28 mg, 0.10 mmol, 1.0 equiv.) and benzamide (36 mg, 0.30 mmol, 3.0 equiv.) gave **4g** (73%) as an oil. ^{1}H NMR (400 MHz, $CDCl_3$) δ 7.85–7.79 (2H, m), 7.63–7.31 (8H, m), 6.47 (1H, brs), 5.33 (1H, p, J = 6.6 Hz), 1.60 (3H, d, J = 6.9 Hz); ^{13}C NMR (101 MHz, $CDCl_3$) δ 168.4, 143.0, 133.8, 132.1, 129.0, 128.5, 127.5, 126.4, 125.7, 50.3, 21.9. Data in accordance with literature.[35]

tert-Butyl (1-phenylethyl)carbamate (4h)

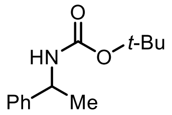

Following **GP5**, **6d** (28 mg, 0.10 mmol, 1.0 equiv.) and *tert*-butylcarbamate (35 mg, 0.30 mmol, 3.0 equiv.) gave **4h** (70%) as an oil. ^{1}H NMR (400 MHz, $CDCl_3$) δ 7.39–7.26 (5H, m), 4.80 (1H, brs), 1.53 –1.40 (12H, m); ^{13}C NMR (101 MHz, $CDCl_3$) δ 155.2, 144.1, 128.5, 127.0, 125.9, 79.5, 50.2, 28.4, 22.6. Data in accordance with the literature.[36]

4-Methyl-N-(1-phenylethyl)benzenesulfonamide (4i)

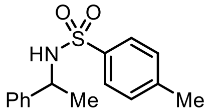

Following **GP5**, **6d** (28 mg, 0.10 mmol, 1.0 equiv.) and tosylamine (51 mg, 0.30 mmol, 3.0 equiv.) gave **4i** (98%) as an oil. ^{1}H NMR (400 MHz, $CDCl_3$) δ 7.65–7.58 (2H, m), 7.19 (5H, m), 7.10 (2H, m), 4.78 (1H, d, *J* = 6.9 Hz), 4.46 (1H, p, *J* = 6.9 Hz), 2.39 (3H, s), 1.43 (3H, d, *J* = 6.9 Hz); ^{13}C NMR (101 MHz, $CDCl_3$) δ 143.1, 142.0, 137.7, 129.5, 128.6, 127.5, 127.1, 126.1, 53.6, 23.5, 21.5. Data in accordance with literature.[37]

N-(1-Phenylethyl)-4-(4,4,5,5-tetramethyl-1,3,2-dioxaborolan-2-yl)benzamide (4j)

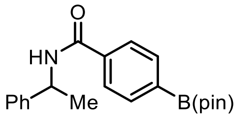

Following **GP5**, **6d** (28 mg, 0.10 mmol, 1.0 equiv.) and 4-(4,4,5,5-tetramethyl-1,3,2-dioxaborolan-2-yl)benzamide (74 mg, 0.30 mmol, 3.0 equiv.) gave **4j** (61%) as an oil. ^{1}H NMR (400 MHz, $CDCl_3$) δ 7.85 (2H, d, *J* = 7.9 Hz), 7.75 (2H, d, *J* = 8.0 Hz), 7.44–7.33 (4H, m), 7.31–7.25 (1H, m), 6.32 (1H, d, *J* = 7.7 Hz), 5.35 (1H, p, *J* = 7.0 Hz), 1.62 (3H, d, *J* = 6.9 Hz), 1.35 (12H, s); ^{13}C NMR (101 MHz, $CDCl_3$) δ 166.5, 143.0, 136.8 135.0, 128.8, 127.5, 126.3, 126.0, 84.1, 49.3, 30.4, 24.9, 21.9. Data in accordance with literature.[38]

N-(1-Phenylethyl)furan-2-carboxamide (4k)

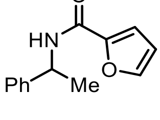

Following **GP5**, **6d** (28 mg, 0.10 mmol, 1.0 equiv.) and furan-2-carboxamide (33 mg, 0.30 mmol, 3.0 equiv.) gave **4k** (67%) as an oil. ^{1}H NMR (400 MHz, $CDCl_3$) δ 7.43–7.31 (5H, m), 7.31–7.25 (1H, m), 7.11 (1H, dd, *J* = 3.5, 0.9 Hz), 6.55 (1H, br s), 6.49 (1H, dd, *J* = 3.5, 1.8 Hz), 5.31 (1H, p, *J* = 7.1 Hz),

1.60 (3H, d, J = 7.0 Hz); ^{13}C NMR (151 MHz, $CDCl_3$) δ 157.6, 148.2, 143.9, 143.1, 128.9, 127.6, 126.4, 114.4, 112.3, 48.6, 21.9. Data in accordance with literature.[36]

N-(1-Phenylethyl)acetamide (4l)

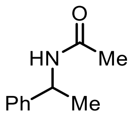

Following **GP5**, **6d** (28 mg, 0.10 mmol, 1.0 equiv.) and acetamide (18 mg, 0.30 mmol, 3.0 equiv.) gave **4l** (26%) as an oil. ^{1}H NMR (600 MHz, $CDCl_3$) δ 7.34–7.28 (4H, m), 7.25–7.21 (1H, m), 5.66 (1H, br s), 5.13 (1H, p, J = 7.2 Hz), 1.99 (1H, s), 1.50 (1H, d, J = 6.9 Hz); ^{13}C NMR (101 MHz, $CDCl_3$) δ 169.2, 143.2, 128.9, 127.6, 126.4, 49.0, 29.9, 21.8. Data in accordance with the literature.[36]

N-(1-Phenylethyl)cyclopropanecarboxamide (4m)

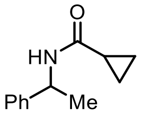

Following **GP5**, **6d** (28 mg, 0.10 mmol, 1.0 equiv.) and cyclopropanecarboxamide (26 mg, 0.30 mmol, 3.0 equiv.) gave **4m** (50%) as an oil. ^{1}H NMR (400 MHz, $CDCl_3$) δ 7.40–7.30 (4H, m), 7.30–7.23 (1H, m), 5.83 (1H, br s), 5.15 (1H, p, J = 7.1 Hz), 1.50 (3H, d, J = 6.9 Hz), 1.35–1.27 (1H, m), 1.05–0.91 (2H, m), 0.78–0.62 (2H, m); ^{13}C NMR (101 MHz, $CDCl_3$) δ 172.7, 143.5, 128.8, 127.5, 126.4, 49.0, 22.0, 15.0, 7.3. Data in accordance with the literature.[39]

N-(1-Phenylethyl)methanesulfonamide (4n)

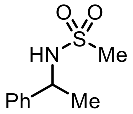

Following **GP5**, **6d** (28 mg, 0.10 mmol, 1.0 equiv.) and methanesulfonamide (29 mg, 0.30 mmol, 3.0 equiv.) gave **4n** (67%) as an oil. ^{1}H NMR (400 MHz, $CDCl_3$) δ 7.39–7.25 (5H, m), 4.97 (1H, d, J = 7.1 Hz), 4.63 (1H, p, J = 6.9 Hz), 2.60 (3H, s), 1.52 (3H, d, J = 6.9 Hz); ^{13}C NMR (101 MHz, $CDCl_3$) δ 142.6, 129.1, 128.1, 126.4, 53.9, 41.9, 24.1. Data in accordance with literature.[40]

4-(5-Methyl-3-phenylisoxazol-4-yl)-N-(1-phenylethyl)benzenesulfonamide (4o)

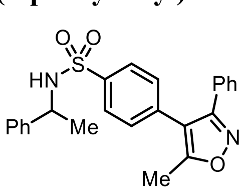

Following **GP5**, **6d** (28 mg, 0.10 mmol, 1.0 equiv.) and 4-(5-methyl-3-phenylisoxazol-4-yl)benzenesulfonamide (94 mg, 0.30 mmol, 3.0 equiv.) gave **4o** (48%) as an oil. R_f 0.03 [pentane:EtOAc

(90:10)]; ^{1}H NMR (400 MHz, $CDCl_3$) δ 7.68 (2H, d, *J* = 8.4 Hz), 7.43–7.29 (5H, m), 7.16 (5H, m), 7.09 (2H, m), 4.84 (1H, d, *J* = 7.1 Hz), 4.57 (1H, p, *J* = 6.9 Hz), 2.46 (3H, s), 1.48 (3H, d, *J* = 6.9 Hz); ^{13}C NMR (101 MHz, $CDCl_3$) δ 167.2, 141.7, 139.9, 134.9, 130.0, 129.7, 128.7, 128.6, 128.5, 128.4, 127.7, 127.4, 126.1, 114.5, 53.8, 31.3, 23.7, 11.7. HRMS (ESI): Found MNa^+ 441.12418, $C_{24}H_{22}N_2O_3SNa$ requires 441.12433.

N-(1-Phenylethyl)-4-(5-(p-tolyl)-3-(trifluoromethyl)-1H-pyrazol-1yl)benzenesulfonamide (4p)

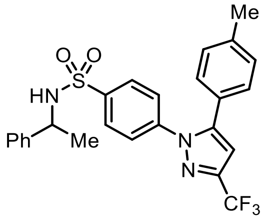

Following **GP5**, **6d** (28 mg, 0.10 mmol, 1.0 equiv.) and celecoxib (114 mg, 0.30 mmol, 3.0 equiv.) gave **4p** (94 %) as an oil. ^{1}H NMR (400 MHz, $CDCl_3$) δ 7.69 (2H, d, *J* = 8.6 Hz), 7.33 (2H, d, *J* = 8.7 Hz), 7.27–7.17 (3H, m), 7.16 (2H, d, *J* = 8.0 Hz), 7.14–7.07 (2H, m), 7.07 (2H, d, *J* = 8.1 Hz), 6.73 (1H, s), 5.00 (1H, d, *J* = 6.9 Hz), 4.51 (1H, p, *J* = 6.9 Hz), 2.38 (3H, s), 1.44 (3H, d, *J* = 6.9 Hz); ^{13}C NMR (101 MHz, $CDCl_3$) δ 145.2, 144.0 (q, *J* = 38.5 Hz), 142.2, 141.6, 140.2, 139.7, 129.7, 128.7, 128.7, 128.0, 127.8, 126.1, 125.8, 125.2, 121.1 (q, *J* = 270.1 Hz), 106.3 (d, *J* = 2.2 Hz), 53.9, 23.5, 21.3. Data in accordance with literature.[37]

1-((1S,4S)-4-(3,4-Dichlorophenyl)-1,2,3,4-tetrahydronaphthalen-1-yl)azetidin-2-one (4qa) & 1-((1R,4S)-4-(3,4-Dichlorophenyl)-1,2,3,4-tetrahydronaphthalen-1-yl)azetidin-2-one (4qb)

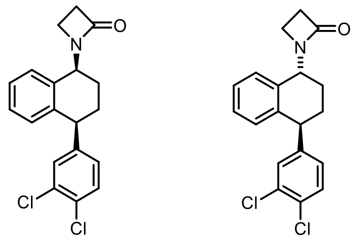

Following **GP5**, **6g** (45 mg, 0.10 mmol, 1.0 equiv.) and azetidinone (21 mg, 0.30 mmol, 3.0 equiv.) gave **4q** (36%) as a separable mixture of diastereomers, as an oil. dr = 2:1.

Data for the major diastereomer: R_f 0.09 [pentane:EtOAc (90:10)]; ^{1}H NMR (400 MHz, $CDCl_3$) δ 7.39–7.24 (3H, m), 7.19 (1H, t, *J* = 7.6 Hz), 7.10 (1H, d, *J* = 2.1 Hz), 6.93–6.85 (2H, m), 5.09 (1H, t, *J* = 6.4 Hz), 4.11 (1H, t, *J* = 5.4 Hz), 3.22–3.15 (1H, m), 3.17–3.09 (1H, m), 2.97 (2H, t, *J* = 4.1 Hz), 2.27–2.12 (1H, m), 1.99–1.87 (3H, m); ^{13}C NMR (101 MHz, $CDCl_3$) δ 167.9, 146.7, 138.6, 134.6, 132.5, 130.8, 130.55, 130.5, 130.4, 129.0, 128.2, 128.1, 127.6, 49.7, 44.0, 37.0, 36.3, 30.0, 24.5; HRMS (ESI): Found MNa^+ 368.05799, $C_{19}H_{17}ONCl_2Na$ requires 368.05794.

Data for the minor diastereomer: R_f 0.08 [pentane:EtOAc (90:10)]; ^{1}H NMR (400 MHz, $CDCl_3$) δ 7.29 (1H, d, *J* = 8.2 Hz), 7.23–7.13 (2H, m), 7.13–7.03 (2H, m), 6.82 (1H, dd, *J* = 8.2, 2.1 Hz), 6.74 (1H, d,

J = 7.8 Hz), 5.12 (1H, t, J = 7.0 Hz), 4.01 (1H, dd, J = 8.7, 5.3 Hz), 3.11 (1H, dt, J = 5.6, 4.5 Hz), 3.00 (1H, dt, J = 5.2, 4.3 Hz), 2.91 (2H, t, J = 4.1 Hz), 2.22–2.11 (1H, m), 2.10–1.99 (1H, m), 1.90–1.73 (2H, m); ^{13}C NMR (101 MHz, $CDCl_3$) δ 167.9, 146.5, 139.1, 134.6, 132.5, 130.6, 130.55, 130.5, 130.1, 128.0, 127.9, 127.7, 127.2, 49.8, 45.0, 36.4, 36.2, 31.2, 26.7; HRMS (ESI): Found MNa^+ 368.05806, $C_{19}H_{17}ONCl_2Na$ requires 368.05794.

3-(1-((4-Methylphenyl)sulfonamido)ethyl)phenyl Ethyl(methyl)carbamate (4r)

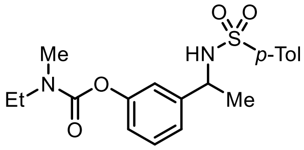

Following **GP5**, **6k** (38 mg, 0.10 mmol, 1.0 equiv.) and tosylamine (51 mg 0.30 mmol, 3.0 equiv.) gave **4r** (52%) as an oil. R_f 0.14 [pentane:EtOAc (70:30)]; ^{1}H NMR (600 MHz, $CDCl_3$, rotamers) δ 7.63 (2H, d, J = 7.9 Hz), 7.20 (2H, d, J = 8.0 Hz), 7.17 (1H, t, J = 8.0 Hz), 6.95 (1H, t, J = 7.6 Hz), 6.92–6.85 (2H, m), 4.73 (1H, d, J = 6.9 Hz), 4.46 (1H, p, J = 6.9 Hz), 3.46 & 3.40 (2H, q, J = 7.1 Hz), 3.05 & 2.98 (3H, s), 2.39 (3H, s), 1.41 (3H, d, J = 6.8 Hz), 1.24 & 1.19 (3H, t, J = 7.1 Hz); ^{13}C NMR (151 MHz, $CDCl_3$, rotamers) δ 151.8, 143.5, 143.3, 137.7, 129.7, 129.5, 127.3, 123.0, 121.1, 119.8, 53.4, 44.2, 34.4, 34.0, 23.5, 21.6, 13.4, 12.6; HRMS (ESI): Found MNa^+ 399.13419, $C_{19}H_{24}O_4N_2SNa$ requires 399.13490.

1-(3-Azido-3-phenylpropoxy)naphthalene (4s)

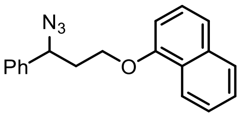

Following **GP6, 1z** (31 mg, 0.10 mmol, 1.0 equiv.) and Me_3SiN_3 (26 µL, 0.2 mmol, 2.0 equiv.) gave **4s** (30%) as an oil. R_f 0.84 [pentane:EtOAc (95:5)]; ^{1}H NMR (600 MHz, $CDCl_3$) δ 8.27–8.23 (1H, m), 7.83–7.79 (1H, m), 7.54–7.46 (2H, m), 7.45–7.33 (7H, m), 6.77 (1H, d, J = 7.6 Hz), 4.93 (1H, dd, J = 8.3, 6.4 Hz), 4.28 (1H, ddd, J = 9.3, 7.3, 4.8 Hz), 4.10 (1H, dt, J = 9.5, 5.8 Hz), 2.48–2.39 (1H, m), 2.38–2.29 (1H, m); ^{13}C NMR (151 MHz, $CDCl_3$) δ 154.5, 139.3, 134.7, 129.1, 128.6, 127.7, 127.2, 126.6, 126.0, 125.7, 125.4, 122.0, 120.6, 104.8, 64.5, 63.4, 36.2; HRMS (ESI): Found MNa^+ 326.12705, $C_{19}H_{17}ON_3Na$ requires 326.12638.

N-(1-(Naphthalen-1-yl)ethyl)-3-(3-(trifluoromethyl)phenyl)propanamide (4t)

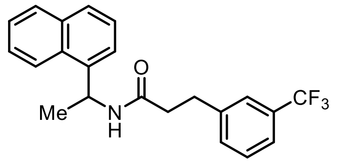

Following **GP5**, **6o** (33 mg, 0.10 mmol, 1.0 equiv.) and 3-(3-(trifluoromethyl)phenyl)propanamide (65 mg, 0.30 mmol, 3.0 equiv.) gave **4t** (66%) as an oil. ^{1}H NMR (600 MHz, $CDCl_3$) δ 8.06–8.01 (1H, m), 7.89–7.83 (1H, m), 7.79 (1H, dd, *J* = 6.2, 3.3 Hz), 7.54–7.46 (2H, m), 7.46–7.39 (4H, m), 7.37–7.29 (2H, m), 5.95–5.87 (1H, p, *J* = 7.7 Hz), 5.61 (1H, d, *J* = 8.2 Hz), 3.06 (1H, dt, *J* = 14.3, 7.5 Hz), 3.01 (1H, dt, *J* = 14.3, 7.5 Hz), 2.46 (1H, dt, *J*=14.7, 7.5 Hz), 1.60 (3H, d, *J* = 6.8 Hz); ^{13}C NMR (101 MHz, $CDCl_3$) δ 170.3, 141.7, 138.0, 133.9, 132.0 (d, *J* = 1.3 Hz), 131.1, 130.8 (p, *J* = 32.0 Hz), 128.9, 128.8, 128.4, 126.6, 125.9, 125.2, 125.0 (q, *J* = 3.8 Hz), 123.3, 123.1 (d, *J* = 3.7 Hz), 122.8 (q, *J* = 272.9 Hz), 122.5, 44.7, 38.0, 31.3, 20.6; ^{19}F NMR (565 MHz, $CDCl_3$) δ –62.57; HRMS (ESI): Found MNa^+ 395.12505, $C_{24}H_{20}O_4Na$ requires 395.12538.

3-(1-Phenylethyl)oxazolidin-2-one (4u)

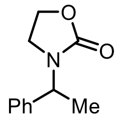

Following **GP5**, **6d** (28 mg, 0.10 mmol, 1.0 equiv.) and oxazolidinone (26 mg, 0.30 mmol, 3.0 equiv.) gave **4u** (75%) as an oil. ^{1}H NMR (400 MHz, $CDCl_3$) δ 7.46–7.27 (5H, m), 5.21 (1H, q, *J* = 7.3 Hz), 4.33 (1H, td, *J* = 9.0, 6.8 Hz), 4.25 (1H, td, *J* = 8.9, 6.8 Hz), 3.52 (1H, td, *J* = 8.9, 6.9 Hz), 3.18 (1H, td, *J* = 8.9, 6.8 Hz), 1.59 (3H, d, *J* = 7.1 Hz); ^{13}C NMR (101 MHz, $CDCl_3$) δ 158.5, 139.5, 128.8, 128.1, 127.1, 62.4, 51.7, 40.3, 16.5. Data in accordance with literature.[41]

1-Methyl-3-(1-phenylethyl)imidazolidin-2-one (4v)

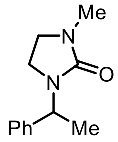

Following **GP5**, **6d** (28 mg, 0.10 mmol, 1.0 equiv.) and 1-methylimidazolidin-2-one (30 mg, 0.30 mmol, 3.0 equiv.) gave **4v** (50%) as an oil. ^{1}H NMR (400 MHz, $CDCl_3$) δ 7.34–7.31 (4H, m), 7.28–7.22 (1H, m), 5.27 (1H, q, *J* = 7.1 Hz), 3.30–3.11 (3H, m), 2.93–2.88 (1H, m), 2.79 (3H, s), 1.51 (3H, d, *J* = 7.1 Hz); ^{13}C NMR (101 MHz, $CDCl_3$) δ 161.2, 140.9, 128.5, 127.4, 67.1, 50.5, 45.4, 37.7, 31.6, 16.3. HRMS data could not be obtained for this compound.

1-(1-Phenylethyl)azetidin-2-one (4w)

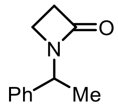

Following **GP5**, **6d** (28 mg, 0.10 mmol, 1.0 equiv.) and azetidinone (21 mg, 0.30 mmol, 3.0 equiv.) gave **4w** (60%) as an oil. ^{1}H NMR (400 MHz, $CDCl_3$) δ 7.40–7.33 (2H, m), 7.33–7.24 (3H, m), 4.92 (1H, q, *J* = 7.0 Hz), 3.21 (1H, td, *J* = 5.2, 3.2 Hz), 3.02 (1H, td, *J* = 5.3, 3.1 Hz), 2.94–2.79 (2H, m), 1.59 (3H, d, *J* = 7.1 Hz); ^{13}C NMR (101 MHz, $CDCl_3$) δ 167.1, 140.6, 128.7, 127.6, 126.7, 51.5, 36.5, 35.7, 18.4. Data in accordance with literature.[42]

2-(1-(4-Methoxyphenyl)ethoxy)isoindoline-1,3-dione (5a)

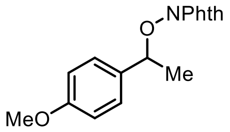

Following **GP9, 1d** (21 mg, 0.10 mmol, 1.0 equiv.) and NHPI (33 mg, 0.20 mmol, 2.0 equiv.) gave **5a** (58%, 23% rsm) as an oil. ^{1}H NMR (400 MHz, $CDCl_3$) δ 7.77–7.67 (4H, m), 7.42 (2H, d, *J* = 8.7 Hz), 6.85 (2H, d, *J* = 8.7 Hz), 5.46 (1H, q, *J* = 6.6 Hz), 3.78 (3H, s), 1.70 (3H, d, *J* = 6.6 Hz); ^{13}C NMR (101 MHz, $CDCl_3$) δ 164.0, 160.3, 134.4, 131.1, 129.3, 129.0, 123.5, 113.8, 84.9, 55.4, 20.4. Data in accordance with literature.[43]

***tert*-Butyl 3-(1-Phenylethoxy)azetidine-1-carboxylate (5b)**

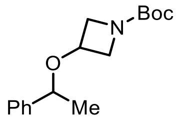

Following **GP5**, using 2,6-di-*tert*-butyl-4-methylpyridine (21 mg, 0.10 mmol, 1.0 equiv.) as a base, **6d** (28 mg, 0.10 mmol, 1.0 equiv.) and 1-Boc-3-hydroxyazetidine (52 mg, 0.30 mmol, 3.0 equiv.) gave **5b** (61%) as an oil. R_f 0.75 [pentane:EtOAc (80:20)]; ^{1}H NMR (400 MHz, $CDCl_3$) δ 7.36–7.26 (5H, m), 4.37 (1H, q, *J* = 6.5 Hz), 4.19–4.10 (1H, m), 4.02 (1H, dd, *J* = 9.1, 6.4 Hz), 3.90–3.76 (2H, m), 3.70 (1H, dd, *J* = 9.4, 4.7 Hz), 1.46 (3H, d, *J* = 6.5 Hz), 1.41 (9H, s); ^{13}C NMR (101 MHz, $CDCl_3$, rotamers) δ 156.5, 142.8, 128.7, 128.1, 126.5, 79.6, 77.2, 65.9, 57.1 (2C), 28.5, 23.8; HRMS (ESI): Found MNa^+ 300.15665, $C_{16}H_{23}O_3NNa$ requires 300.15701.

1-Phenylethyl Benzoate (5c)

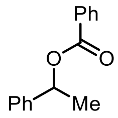

Following **GP5**, using 2,6-di-*tert*-butyl-4-methylpyridine (21 mg, 0.10 mmol, 1.0 equiv.) as a base, **6d** (28 mg, 0.10 mmol, 1.0 equiv.) and benzoic acid (37 mg, 0.30 mmol, 3.0 equiv.) gave **5c** (85%) as an

oil. ^{1}H NMR (400 MHz, $CDCl_3$) δ 8.09 (2H, d, *J* = 6.9 Hz), 7.56 (1H, t, *J* = 7.4 Hz), 7.47–7.41 (4H, m), 7.40–7.35 (2H, m), 7.34–7.28 (1H, m), 6.14 (1H, q, *J* = 6.6 Hz), 1.68 (3H, d, *J* = 6.6 Hz); ^{13}C NMR (101 MHz, $CDCl_3$) δ 166.0, 141.9, 133.0, 130.7, 129.8, 128.7, 128.5, 128.0, 126.2, 73.1, 22.6. Data in accordance with literature.[44]

(1-(Benzyloxy)ethyl)benzene (5d)

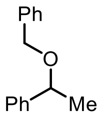

Following **GP5**, using 2,6-di-*tert*-butyl-4-methylpyridine (21 mg, 0.10 mmol, 1.0 equiv.) as a base, **6d** (28 mg, 0.10 mmol, 1.0 equiv.) and BnOH (31 μL, 0.30 mmol, 3.0 equiv.) gave **5d** (61%) as an oil. ^{1}H NMR (400 MHz, $CDCl_3$) δ 7.38–7.27 (10H, m), 4.50 (1H, q, *J* = 6.5 Hz), 4.45 (1H, d, *J* = 11.8 Hz), 4.30 (1H, d, *J* = 11.8 Hz), 1.49 (3H, d, *J* = 6.4 Hz); ^{13}C NMR (101 MHz, $CDCl_3$) δ 138.8, 128.6, 128.5, 127.8, 127.6, 127.6, 126.5, 77.4, 70.5, 24.4. Data in accordance with literature.[45]

***tert*-Butyl 4-(1-Phenylethoxy)piperidine-1-carboxylate (5e)**

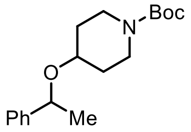

Following **GP5**, using 2,6-di-*tert*-butyl-4-methylpyridine (21 mg, 0.10 mmol, 1.0 equiv.) as a base, **6d** (28 mg, 0.10 mmol, 1.0 equiv.) and 1-Boc-4-hydroxypiperidine (60 mg, 0.30 mmol, 3.0 equiv.) gave **5e** (64%) as an oil. R_f 0.86 [pentane:EtOAc (80:20)]; ^{1}H NMR (400 MHz, $CDCl_3$) δ 7.36–7.24 (5H, m), 4.57 (1H, q, *J* = 6.5 Hz), 3.84–3.68 (2H, m), 3.31 (1H, tt, *J* = 8.2, 3.7 Hz), 3.05–2.90 (2H, m), 1.88–1.79 (1H, m), 1.69–1.60 (1H, m), 1.55–1.46 (2H, m), 1.44 (9H, s), 1.41 (3H, d, *J* = 6.5 Hz); ^{13}C NMR (101 MHz, $CDCl_3$, rotamers) δ 155.0, 144.6, 128.5, 127.5, 126.2, 79.5, 74.8, 72.0, 41.5 (2C), 32.3, 30.7, 28.6, 24.9; HRMS (ESI): Found MNa^+ 328.18788, $C_{18}H_{27}O_3NNa$ requires 328.18831.

***tert*-Butyl 6-(1-Phenylethoxy)-2-azaspiro[3.3]heptane-2-carboxylate (5f)**

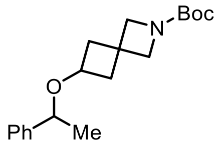

Following **GP5**, using 2,6-di-*tert*-butyl-4-methylpyridine (21 mg, 0.10 mmol, 1.0 equiv.) as a base, **6d** (28 mg, 0.10 mmol, 1.0 equiv.) and *tert*-butyl 6-hydroxy-2-azaspiro[3.3]heptane-2-carboxylate (64 mg, 0.30 mmol, 3.0 equiv.) gave **5f** (63%) as an oil. R_f 0.57 [pentane:EtOAc (80:20)]; ^{1}H NMR (400 MHz, $CDCl_3$) δ 7.38–7.26 (5H, m), 4.35 (1H, q, *J* = 6.5 Hz), 3.84 (2H, s), 3.81 (2H, s), 3.76 (1H, p, *J* = 7.3 Hz), 2.49–2.37 (1H, m), 2.27–2.17 (1H, m), 2.13 (1H, dd, *J* = 7.8, 3.9 Hz), 2.05 (1H, dd, *J* = 11.9, 7.6 Hz), 1.42 (3H, d, *J* = 2.0 Hz), 1.40 (9H, s); ^{13}C NMR (101 MHz, $CDCl_3$, rotamers) δ 156.3, 143.8, 128.5,

127.7, 126.4, 79.4, 75.9, 66.3, 61.3 (2C), 41.7, 41.6, 30.2, 28.5, 24.2; HRMS (ESI): Found MNa^+ 340.18831, $C_{19}H_{27}O_3NNa$ requires 340.18831.

1-Phenylethyl Acetate (5g)

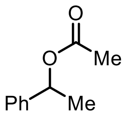

Following **GP5**, using 2,6-di-*tert*-butyl-4-methylpyridine (21 mg, 0.10 mmol, 1.0 equiv.) as a base, **6d** (28 mg, 0.10 mmol, 1.0 equiv.) and AcOH (17 μL, 0.30 mmol, 3.0 equiv.) gave **5g** (82%) as an oil. ^{1}H NMR (400 MHz, $CDCl_3$) δ 7.35–7.31 (5H, m), 5.87 (1H, q, J = 6.6 Hz), 2.06 (3H, s), 1.52 (3H, d, J = 6.7 Hz); ^{13}C NMR (101 MHz, $CDCl_3$) δ 170.5, 141.8, 128.6, 128.0, 126.2, 72.5, 22.3, 21.5. Data in accordance with literature.[46]

1-Phenylethyl Methacrylate (5h)

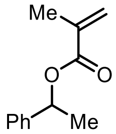

Following **GP5**, using 2,6-di-*tert*-butyl-4-methylpyridine (21 mg, 0.10 mmol, 1.0 equiv.) as a base, **6d** (28 mg, 0.10 mmol, 1.0 equiv.) and methacrylic acid (25 μL, 0.30 mmol, 3.0 equiv.) gave **5h** (63%) as an oil. ^{1}H NMR (400 MHz, $CDCl_3$) δ 7.40–7.32 (4H, m), 7.31–7.26 (1H, m), 6.16 (1H, s), 5.95 (1H, q, J = 6.6 Hz), 5.60–5.54 (1H, m), 1.58 (3H, d, J = 6.6 Hz); ^{13}C NMR (101 MHz, $CDCl_3$) δ 166.8, 142.0, 136.8, 128.6, 127.9, 126.1, 125.6, 72.7, 22.5, 18.5. Data in accordance with literature.[47]

1-Phenylethyl 2-(11-Oxo-6,11-dihydrodibenzo[b,e]oxepin-2-yl)acetate (5i)

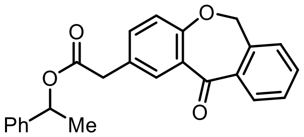

Following **GP5**, using 2,6-di-*tert*-butyl-4-methylpyridine (21 mg, 0.10 mmol, 1.0 equiv.) as a base, **6d** (28 mg, 0.10 mmol, 1.0 equiv.) and isoxepac (81 mg, 0.30 mmol, 3.0 equiv.) gave **5e** (95%) as an oil. R_f 0.14 [pentane:EtOAc (90:10)]; ^{1}H NMR (400 MHz, $CDCl_3$) δ 8.13 (1H, d, J = 2.4 Hz), 7.90 (1H, d, J = 7.6 Hz), 7.56 (1H, td, J = 7.5, 1.5 Hz), 7.47 (1H, td, J = 7.6, 1.3 Hz), 7.40 (1H, dd, J = 8.4, 2.4 Hz), 7.36 (1H, d, J = 7.6 Hz), 7.34–7.25 (5H, m), 7.01 (1H, d, J = 8.4 Hz), 5.90 (1H, q, J = 6.6 Hz), 5.19 (2H, s), 3.69 (1H, d, J = 15.8 Hz), 3.65 (1H, d, J = 15.8 Hz), 1.54 (3H, d, J = 6.6 Hz); ^{13}C NMR (101 MHz, $CDCl_3$) δ 190.9, 170.8, 160.6, 141.6, 140.6, 136.5, 135.7, 132.9, 132.6, 129.6, 129.4, 128.6, 128.0, 127.95, 127.9, 126.2, 125.3, 121.1, 73.8, 73.1, 40.6, 22.3; HRMS (ESI): Found MNa^+ 395.12505, $C_{24}H_{20}O_4Na$ requires 395.12538.

2-(((1S,4S)-4-(3,4-Dichlorophenyl)-1,2,3,4-tetrahydronaphthalen-1-yl)oxy)isoindoline-1,3-dione (5ja) & 2-(((1R,4S)-4-(3,4-Dichlorophenyl)-1,2,3,4-tetrahydronaphthalen-1-yl)oxy)isoindoline-1,3-dione (5jb)

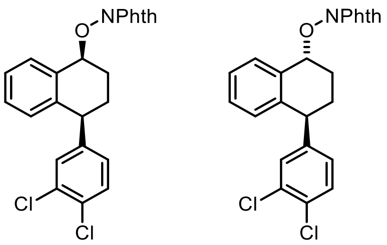

Following **GP9, 1x** (31 mg, 0.10 mmol, 1.0 equiv.) and NHPI (33 mg, 0.20 mmol, 2.0 equiv.) gave **5f** (43%, 5% rsm) as a mixture of diastereomers, as an oil. dr = 1.3:1. R_f 0.72 [pentane:EtOAc (85:15)]; ^{1}H NMR (600 MHz, $CDCl_3$, diastereomers) δ 7.86–7.83 (2H, m), 7.77–7.74 (2H, m), 7.56 (0.54H, d, *J* = 7.5 Hz), 7.50 (0.46H, d, *J* = 7.4 Hz), 7.45–7.40 (1H, m), 7.37–7.16 (3H, m), 7.06 (0.47H, d, *J* = 2.1 Hz), 6.96 (0.47H, d, *J* = 1.8 Hz), 6.85 (0.48H, d, *J* = 7.6 Hz), 6.81 (0.48H, dd, *J* = 8.3, 2.1 Hz), 5.40 (0.47H, t, *J* = 3.9 Hz), 5.36 (0.54H, t, *J* = 3.0 Hz), 4.33 (0.46H, t, *J* = 5.0 Hz), 3.99 (0.53H, dd, *J* = 11.7, 5.8 Hz), 2.84–2.76 (0.47H, m), 2.56 (0.52H, tdd, *J* = 13.8, 11.6, 2.8 Hz), 2.46 (0.53H, dq, *J* = 14.4, 3.2 Hz), 2.17–2.12 (0.46H, m), 2.11–2.05 (0.54H, m), 2.02–1.89 (1H, m), 1.84–1.79 (0.46H, m); ^{13}C NMR (151 MHz, $CDCl_3$, diastereomers) δ 164.4, 164.3 146.9, 146.5, 140.9, 139.2, 134.6, 134.6, 134.0, 132.5, 132.5, 132.2, 131.7, 131.4, 131.1, 130.8, 130.6, 130.5, 130.3, 129.8, 129.7, 129.2, 129.1, 128.6, 128.4, 128.2, 127.3, 126.7, 124.5, 123.7, 123.6, 82.9, 82.7, 45.8, 43.3, 31.9, 27.8, 27.1, 26.9; HRMS (ESI): Found MNa^+ 460.04973, $C_{24}H_{17}O_3NCl_2Na$ requires 460.04777.

***tert*-Butyl 3-(((1S,4S)-4-(3,4-dichlorophenyl)-1,2,3,4-tetrahydronaphthalen-1-yl)oxy)azetidine-1-carboxylate (5ka) & *tert*-Butyl 3-(((1R,4S)-4-(3,4-dichlorophenyl)-1,2,3,4-tetrahydronaphthalen-1-yl)oxy)azetidine-1-carboxylate (5kb)**

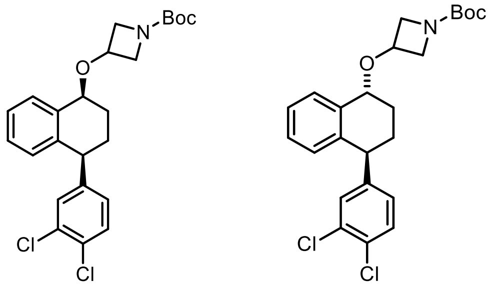

Following **GP5**, using 2,6-di-*tert*-butyl-4-methylpyridine (21 mg, 0.10 mmol, 1.0 equiv.) as a base, **6g** (45 mg, 0.10 mmol, 1.0 equiv.) and 1-Boc-3-hydroxyazetidine (52 µL 0.30 mmol, 3.0 equiv.) gave **5g** (74%) as a separable mixture of diastereomers, as an oil. dr 1.5:1.

Data for the major diastereomer: R_f 0.29 [pentane:EtOAc (90:10)]; ^{1}H NMR (400 MHz, $CDCl_3$) δ 7.42 (1H, d, *J* = 7.6 Hz), 7.33 (1H, d, *J* = 8.3 Hz), 7.26–7.14 (1H, m), 7.18 (1H, t, *J* = 7.3 Hz), 7.10 (1H, d, *J* = 2.1 Hz), 6.88–6.80 (2H, m), 4.53 (1H, t, *J* = 5.1 Hz), 4.47 (1H, td, *J* = 6.6, 3.3 Hz), 4.19–4.06 (3H,

m), 3.91 (1H, dd, J = 9.2, 4.6 Hz), 3.86 (1H, dd, J = 9.3, 4.6 Hz), 2.44–2.33 (1H, m), 2.06–1.94 (1H, m), 1.86–1.72 (2H, m), 1.44 (9H, s); ^{13}C NMR (101 MHz, $CDCl_3$) δ 156.6, 147.0, 138.4, 136.7, 132.5, 130.7, 130.4, 130.2, 128.8, 128.4, 128.2, 127.1, 79.7, 75.2, 66.2, 57.6 (2C), 44.2, 28.8, 28.5, 26.3; HRMS (ESI): Found MNa^+ 470.12601, $C_{24}H_{27}O_3NCl_2Na$ requires 470.12602.

Data for the minor diastereomer: R_f 0.26 [pentane:EtOAc (90:10)]; ^{1}H NMR (400 MHz, $CDCl_3$) δ 7.32–7.22 (2H, m), 7.21–7.07 (3H, m), 6.89 (1H, dd, J = 8.3, 2.1 Hz), 6.76 (1H, d, J = 7.6 Hz), 4.42 (1H, tt, J = 6.5, 4.6 Hz), 4.36 (1H, d, J = 4.2 Hz), 4.12–4.04 (1H, m), 4.04–3.98 (1H, m), 3.92 (1H, t, J = 7.3 Hz), 3.85 (1H, dd, J = 9.2, 4.6 Hz), 3.77 (1H, dd, J = 9.3, 4.6 Hz), 2.11–1.92 (3H, m), 1.79 (1H, td, J = 11.1, 4.4 Hz), 1.37 (9H, s); ^{13}C NMR (101 MHz, $CDCl_3$) δ 156.4, 147.0, 138.9, 136.0, 132.4, 130.7, 130.4, 130.3, 130.0, 129.5, 128.5, 128.2, 126.7, 79.6, 74.7, 66.0, 57.3, 45.0, 28.4, 28.2, 27.1; HRMS (ESI): Found MNa^+ 395.12505, $C_{24}H_{20}O_4Na$ requires 395.12538.

3-(1-((1,3-Dioxoisoindolin-2-yl)oxy)ethyl)phenyl Ethyl(methyl)carbamate (5l)

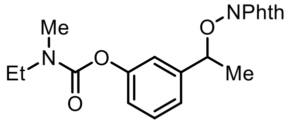

Following **GP9, 1y** (25 mg, 0.10 mmol, 1.0 equiv.) and NHPI (33 mg, 0.20 mmol, 2.0 equiv.) gave **5l** (44%, 19% rsm) as an oil. R_f 0.24 [pentane:EtOAc (85:15)]; ^{1}H NMR (400 MHz, $CDCl_3$, rotamers) δ 7.80–7.74 (2H, m), 7.73–7.68 (2H, m), 7.39–7.30 (2H, m), 7.27–7.25 (1H, m), 7.09 (1H, d, J = 7.9 Hz), 5.48 (1H, q, J = 6.5 Hz), 3.45 & 3.39 (2H, q, J = 6.9 Hz), 3.05 & 2.98 (3H, s), 1.70 (3H, d, J = 6.6 Hz), 1.23 & 1.18 (3H, t, J = 7.3 Hz); ^{13}C NMR (101 MHz, $CDCl_3$, rotamers) δ 164.0, 154.5, 154.3, 151.6, 140.7, 134.5, 129.4, 129.1, 124.3, 123.6, 122.5, 121.0, 84.9, 44.2, 34.4, 34.0, 20.8, 13.4, 12.6; Found MNa^+ 391.12644, $C_{20}H_{20}O_5N_2Na$ requires 391.12644.

***tert*-Butyl 3-(1-(3-((ethyl(methyl)carbamoyl)oxy)phenyl)ethoxy)azetidine-1-carboxylate (5m)**

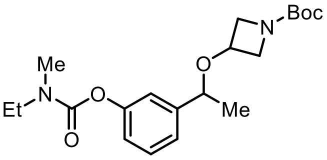

Following **GP5**, using 2,6-di-*tert*-butyl-4-methylpyridine (21 mg, 0.10 mmol, 1.0 equiv.) as a base, **6k** (38 mg, 0.10 mmol, 1.0 equiv.) and tert-butyl 3-hydroxyazetidine-1-carboxylate (52 mg 0.30 mmol, 3.0 equiv.) gave **5m** (32%) as an oil. R_f 0.43 [pentane:EtOAc (70:30)]; ^{1}H NMR (400 MHz, $CDCl_3$, rotamers) δ 7.32 (1H, t, J = 8.2 Hz), 7.10 (1H, d, J = 7.6 Hz), 7.04 (2H, br s), 4.36 (1H, q, J = 6.5 Hz), 4.16 (1H, tt, J = 6.5, 4.6 Hz), 4.02 (1H, dd, J = 9.2, 6.4 Hz), 3.92–3.82 (2H, m), 3.73 (1H, dd, J = 9.3, 4.6 Hz), 3.47 & 3.42 (2H, q, J = 7.0 Hz), 3.07 & 2.99 (3H, s), 1.45 (3H, d, J = 6.5 Hz), 1.41 (9H, s), 1.24 & 1.19 (3H, t, J = 6.4 Hz); ^{13}C NMR (101 MHz, $CDCl_3$, rotamers) δ 156.5, 151.9, 144.4, 130.4,

129.5, 123.1, 121.4, 119.8, 79.6, 76.7, 65.9, 44.2, 34.4, 34.0, 28.5, 23.8, 13.4, 12.6; HRMS (ESI): Found MNa^+ 401.20422, $C_{20}H_{30}O_5N_2Na$ requires 401.20469.

1-(3-((Ethyl(methyl)carbamoyl)oxy)phenyl)ethyl 2-(11-Oxo-6,11-dihydrodibenzo[b,e]oxepin-2-yl)acetate (5n)

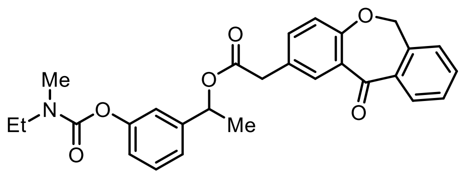

Following **GP5**, using 2,6-di-*tert*-butyl-4-methylpyridine (21 mg, 0.10 mmol, 1.0 equiv.) as a base, **6k** (38 mg, 0.10 mmol, 1.0 equiv.) and isoxepac (52 mg 0.30 mmol, 3.0 equiv.) gave **5n** (49%) as an oil. R_f 0.48 [pentane:EtOAc (70:30)]; ^{1}H NMR (400 MHz, $CDCl_3$, rotamers) δ 8.12 (1H, d, J = 2.4 Hz), 7.89 (1H, dd, J = 7.8, 1.4 Hz), 7.56 (1H, td, J = 7.5, 1.5 Hz), 7.47 (1H, td, J = 7.6, 1.4 Hz), 7.41 (1H, dd, J = 8.4, 2.4 Hz), 7.36 (1H, d, J = 7.4 Hz), 7.30 (1H, t, J = 7.9 Hz), 7.14 (1H, d, J = 7.8 Hz), 7.10–7.03 (2H, m), 7.01 (1H, d, J = 8.5 Hz), 5.89 (1H, q, J = 6.6 Hz), 5.19 (2H, s), 3.69 (1H, d, J = 15.8 Hz), 3.65 (1H, d, J = 15.6 Hz), 3.45 & 3.40 (2H, q, J = 7.2 Hz), 3.05 & 3.01 (3H, s), 1.53 (3H, d, J = 6.6 Hz), 1.26 & 1.21 (3H, q, J = 6.1 Hz); ^{13}C NMR (101 MHz, $CDCl_3$, rotamers) δ 190.9, 170.7, 160.6, 151.8, 142.9, 140.6, 136.5, 135.8, 132.9, 132.7, 129.7, 129.4, 127.9, 125.3, 123.0, 121.5, 121.2, 119.7, 73.8, 72.7, 64.6, 44.2, 40.6, 34.4, 33.9, 25.5, 22.3, 13.4, 12.6; HRMS (ESI): Found MNa^+ 496.17190, $C_{28}H_{27}O_6NNa$ requires 496.17306.

1-(1-(3-(3-(Trifluoromethyl)phenyl)propoxy)ethyl)naphthalene (5o)

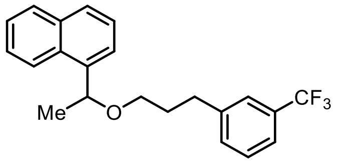

Following **GP5**, using 2,6-di-*tert*-butyl-4-methylpyridine (21 mg, 0.10 mmol, 1.0 equiv.) as a base, **6o** (33 mg, 0.10 mmol, 1.0 equiv.) and 3-(3-(trifluoromethyl)phenyl)propan-1-ol (61 mg, 0.30 mmol, 3.0 equiv.) gave **5o** (83%) as an oil. R_f 0.95 [pentane:EtOAc (50:50)]; ^{1}H NMR (600 MHz, $CDCl_3$) δ 8.21 (1H, d, J = 8.2 Hz), 7.92–7.86 (1H, m), 7.78 (1H, d, J = 8.1 Hz), 7.57 (1H, d, J = 7.1 Hz), 7.54–7.45 (3H, m), 7.45–7.41 (2H, m), 7.39–7.30 (2H, m), 5.13 (1H, q, J = 6.6 Hz), 3.46–3.37 (2H, m), 2.82 (1H, dt, J = 15.3, 7.8 Hz), 2.73 (1H, dt, J = 14.5, 7.7 Hz), 1.94 (2H, p, J = 7.1 Hz), 1.64 (3H, d, J = 6.6 Hz); ^{13}C NMR (151 MHz, $CDCl_3$) δ 143.1, 139.7, 134.1, 132.0, 130.9, 130.7 (q, J = 31.9 Hz), 129.1, 128.8, 128.0, 126.0, 125.7, 126.6, 125.3 (q, J = 3.8 Hz), 123.1 (p, J = 271.9 Hz), 123.6, 123.5, 122.8 (q, J = 3.8 Hz), 76.1, 67.9, 32.5, 31.6, 23.6; ^{19}F NMR (565 MHz, $CDCl_3$) δ –62.53; HRMS (EI): Found M^+ 358.15368, $C_{22}H_{21}OF_3$ requires 358.15390.

1-(Naphthalen-1-yl)ethyl 3-(3-(Trifluoromethyl)phenyl)propanoate (5p)

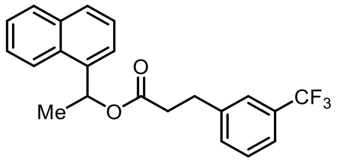

Following GP5, using 2,6-di-*tert*-butyl-4-methylpyridine (21 mg, 0.10 mmol, 1.0 equiv.) as a base, **6o** (33 mg, 0.10 mmol, 1.0 equiv.) and 3-(3-(trifluoromethyl)phenyl)propan-1-ol (61 mg, 0.30 mmol, 3.0 equiv.) gave **5p** (85%) as an oil. R_f 0.97 [pentane:EtOAc (50:50)] ^{1}H NMR (600 MHz, $CDCl_3$) δ 8.05 (1H, d, *J* = 8.1 Hz), 7.87 (1H, d, *J* = 8.1 Hz), 7.80 (1H, d, *J* = 8.1 Hz), 7.53–7.44 (6H, m), 7.38–7.31 (2H, m), 6.65 (1H, q, *J* = 6.6 Hz), 3.07–3.00 (2H, m), 2.80–2.67 (2H, m), 1.67 (3H, d, *J* = 6.6 Hz); ^{13}C NMR (151 MHz, $CDCl_3$) δ 171.9, 141.4, 137.3, 134.0, 131.9, 130.9 (q, *J* = 32.0 Hz), 130.4, 129.2, 129.1, 129.0, 128.7, 126.5, 125.8, 125.5, 125.1 (q, *J* = 3.7 Hz), 124.3 (q, *J* = 272.1 Hz), 123.4 (q, *J* = 3.8 Hz), 123.4, 70.0, 35.9, 30.8, 21.7; ^{19}F NMR (565 MHz, $CDCl_3$) δ –62.60; HRMS (ESI): Found MNa^+ 395.12263; $C_{22}H_{19}O_2F_3Na$ requires 395.12294.

1-(4-Methoxyphenyl)ethyl)(phenyl)sulfane (6a)

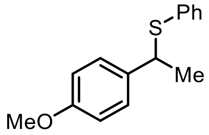

Following **GP10, 1d** (42 mg, 0.20 mmol, 2.0 equiv.) and Ph_2S_2 (22 mg, 0.10 mmol, 1.0 equiv.) gave **6a** (67%, 60% rsm) as an oil. ^{1}H NMR (600 MHz, $CDCl_3$) δ 7.29 (2H, d, *J* = 6.8 Hz), 7.24–7.19 (5H, m), 6.82 (2H, d, *J* = 8.5 Hz), 4.32 (1H, q, *J* = 7.0 Hz), 3.79 (3H, s), 1.61 (3H, d, *J* = 7.0 Hz); ^{13}C NMR (151 MHz, $CDCl_3$) δ 158.8, 135.5, 135.4, 132.6, 128.8, 128.5, 127.2, 113.9, 55.4, 47.5, 22.6. Data in accordance with literature.[48]

2-((1-(4-Methoxyphenyl)ethyl)thio)benzo[d]thiazole (6b)

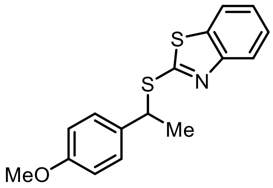

Following **GP10, 1d** (42 mg, 0.20 mmol, 2.0 equiv.) and benzothiazole disulfide (33 mg, 0.10 mmol, 1.0 equiv.) gave **6b** (78%, 71% rsm) as a solid. ^{1}H NMR (600 MHz, $CDCl_3$) δ 7.91 (1H, d, *J* = 8.1 Hz), 7.73 (1H, d, *J* = 8.0 Hz), 7.46–7.38 (3H, m), 7.29 (1H, ddd, *J* = 8.2, 7.3, 1.2 Hz), 6.87 (2H, d, *J* = 8.7 Hz), 5.13 (1H, q, *J* = 7.0 Hz), 3.79 (3H, s), 1.84 (3H, d, *J* = 7.0 Hz); ^{13}C NMR (151 MHz, $CDCl_3$) δ 166.2, 159.3, 153.4, 135.6, 133.9, 128.7, 126.1, 124.4, 121.8, 121.1, 114.2, 55.4, 47.3, 22.8. Data in accordance with literature.[49]

(1-(4-Methoxyphenyl)ethyl)(trifluoromethyl)sulfane (6c)

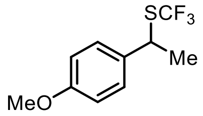

Following **GP11, 1d** (42 mg, 0.20 mmol, 2.0 equiv.) and $AgSCF_3$ (21 mg, 0.10 mmol, 1.0 equiv.) gave **6c** (51%, 69% rsm) as an oil. R_f 0.70 [pentane:EtOAc (98:2)]; ^{1}H NMR (600 MHz, $CDCl_3$) δ 7.28 (2H, d, J = 8.9 Hz), 6.87 (2H, d, J = 8.5 Hz), 4.52 (1H, q, J = 7.0 Hz), 3.81 (3H, s), 1.72 (3H, d, J = 7.1 Hz); ^{13}C NMR (151 MHz, $CDCl_3$) δ 159.4, 133.2, 130.7 (q, J = 307.6 Hz), 128.4, 114.3, 55.4, 44.3, 23.3; ^{19}F NMR (565 MHz, $CDCl_3$) δ –40.15. HRMS data could not be obtained for this compound.

1-Phenyl-5-((1-phenylethyl)thio)-1H-tetrazole (6d)

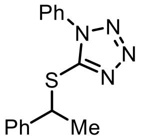

Following **GP12**, **1w** (38 mg, 0.20 mmol, 2.0 equiv.) and 1-Phenyl-1H-tetrazol-5-thiol (18 mg, 0.10 mmol, 1.0 equiv.) gave **6d** (61%, 96% rsm) as a solid. ^{1}H NMR (400 MHz, $CDCl_3$) 7.55–7.45 (5H, m), 7.43–7.39 (2H, m), 7.35–7.26 (3H, m), 5.21 (1H, q, J = 7.0 Hz), 1.88 (3H, d, J = 7.0 Hz); ^{13}C NMR (101 MHz, $CDCl_3$) δ 153.8, 141.0, 133.8, 130.2, 129.8, 129.0, 128.4, 127.4, 124.2, 48.1, 22.4. Data in accordance with literature.[50]

Following **GP13**, amide **1q** (38 mg, 0.20 mmol, 2.0 equiv.) and 1-Phenyl-1H-tetrazol-5-thiol (18 mg, 0.10 mmol, 1.0 equiv.) gave **6d** (58%) as a solid.

((1S,4S)-4-(3,4-Dichlorophenyl)-1,2,3,4-tetrahydronaphthalen-1-yl)(phenyl)sulfane (6ea) & ((1R,4S)-4-(3,4-Dichlorophenyl)-1,2,3,4-tetrahydronaphthalen-1-yl)(phenyl)sulfane (6eb)

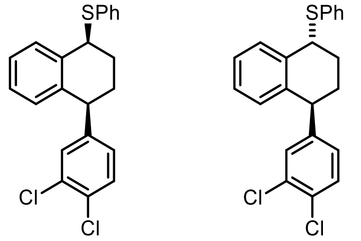

Following **GP10, 1x** (62 mg, 0.20 mmol, 2.0 equiv.) and Ph_2S_2 (22 mg, 0.1 mmol, 1.0 equiv.) gave **6e** (57%, 63% rsm) as a mixture of diastereomers, as an oil. dr = 1.3:1. R_f 0.60 [pentane:EtOAc (98:2)]; ^{1}H NMR (600 MHz, $CDCl_3$, diastereomers) δ 7.56–7.44 (3H, m), 7.37–7.27 (5H, m), 7.26–7.07 (2H, m), 6.96 (0.48H, d, J = 8.3 Hz), 6.88 (0.52H, d, J = 7.7 Hz), 6.80 (0.48H, dd, J = 8.3, 2.1 Hz), 6.77 (0.47H, d, J = 7.8 Hz), 4.65 (0.52H, t, J = 4.7 Hz), 4.61 (0.48H, t, J = 3.7 Hz), 4.19 (0.52H, t, J = 5.0 Hz), 3.98 (0.48H, dd, J = 10.8, 6.1 Hz), 2.69–2.60 (0.52H, m), 2.41–2.30 (0.48H, m), 2.09–2.05 (1H, m), 2.04–1.97 (1H, m), 1.88–1.81 (0.52H, m), 1.78–1.72 (0.52H, m); ^{13}C NMR (101 MHz, $CDCl_3$, diastereomers) 147.5, 147.3, 139.3, 138.0, 136.3, 136.1, 135.8, 135.5, 132.7, 132.5, 132.36, 132.1,

130.9, 130.8, 130.7, 130.7, 130.6 (2C), 130.4, 130.2, 130.2, 130.0, 129.2, 129.2, 128.4, 128.2, 127.8, 127.7, 127.6, 127.3, 127.0, 126.6, 48.0, 47.5, 45.5, 43.5, 28.8, 27.8, 27.5, 24.4. HRMS data could not be obtained for this compound.

2-(((1S,4S)-4-(3,4-Dichlorophenyl)-1,2,3,4-tetrahydronaphthalen-1-yl)thio)benzo[d]thiazole (6fa) & 2-(((1R,4S)-4-(3,4-Dichlorophenyl)-1,2,3,4-tetrahydronaphthalen-1-yl)thio)benzo[d]thiazole (6fb)

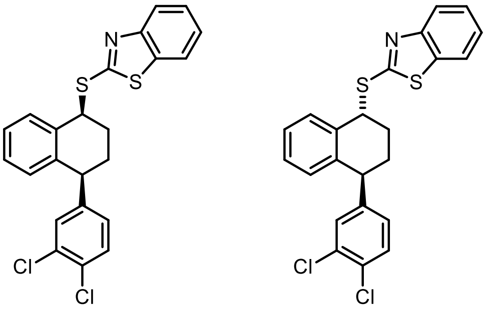

Following **GP10, 1x** (62 mg, 0.20 mmol, 2.0 equiv.) and benzothiazole disulfide (33 mg, 0.1 mmol, 1.0 equiv.) gave **6f** (73%, 77% rsm) as a separable mixture of diastereomers, as an oil. dr = 1.3:1.

Data for the major diastereomer: R_f 0.68 [pentane:EtOAc (95:5)]; ^{1}H NMR (600 MHz, $CDCl_3$) δ 7.90 (1H, d, J = 8.1 Hz), 7.78 (1H, d, J = 8.0 Hz), 7.61 (1H, d, J = 7.8 Hz), 7.43 (1H, t, J = 8.3 Hz), 7.32 (2H, t, J = 8.3 Hz), 7.27–7.24 (1H, m), 7.20 (1H, t, J = 7.6 Hz), 7.07 (1H, s), 6.92 (1H, d, J = 7.7 Hz), 6.82 (1H, d, J = 8.3 Hz), 5.58 (1H, t, J = 4.3 Hz), 4.25 (1H, t, J = 4.8 Hz), 2.60–2.52 (1H, m), 2.36–2.27 (1H, m), 2.25–2.17 (1H, m), 1.93–1.85 (1H, m); ^{13}C NMR (151 MHz, $CDCl_3$) δ 166.3, 153.4, 147.1, 138.4, 135.5, 135.1, 132.5, 131.0, 130.8 (2C), 130.3, 130.3, 128.3, 128.3, 127.5, 126.3, 124.6, 121.8, 121.2, 47.5, 43.4, 28.5, 25.7; HRMS (ESI): Found MNa^+ 464.00807, $C_{23}H_{17}NCl_2NaS_2$ requires 464.00717.

Data for the minor diasteromer: R_f 0.65 [pentane:EtOAc (95:5)]; ^{1}H NMR (400 MHz, $CDCl_3$) δ 7.93 (1H, d, J = 8.1 Hz), 7.79 (1H, d, J = 8.2 Hz), 7.53 (1H, d, J = 7.7 Hz), 7.48–7.42 (1H, m), 7.39 (1H, d, J = 8.2 Hz), 7.35–7.31 (1H, m), 7.30 (1H, d, J = 2.1 Hz), 7.21 (1H, t, J = 7.3 Hz), 7.14 (1H, t, J = 7.5 Hz), 7.02 (1H, dd, J = 8.3, 2.1 Hz), 6.80 (1H, d, J = 7.8 Hz), 5.55 (1H, t, J = 3.6 Hz), 4.08–3.99 (1H, m), 2.53–2.45 (1H, m), 2.39–2.22 (2H, m), 2.17–2.10 (1H, m); ^{13}C NMR (101 MHz, $CDCl_3$) δ 165.9, 153.4, 146.9, 139.7, 135.5, 134.9, 132.7, 130.9, 130.9, 130.7, 130.6, 130.1, 128.4, 128.3, 127.1, 126.3, 124.7, 121.9, 121.2, 48.0, 45.5, 29.6, 28.8; HRMS (ESI): Found MNa^+ 464.00780, $C_{23}H_{17}NCl_2NaS_2$ requires 464.00717.

5-(((1S,4S)-4-(3,4-Dichlorophenyl)-1,2,3,4-tetrahydronaphthalen-1-yl)thio)-1-phenyl-1H-tetrazole (6ga) & 5-(((1R,4S)-4-(3,4-Dichlorophenyl)-1,2,3,4-tetrahydronaphthalen-1-yl)thio)-1-phenyl-1H-tetrazole (6gb)

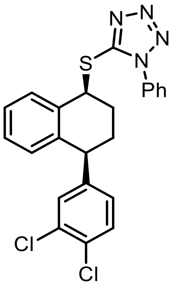

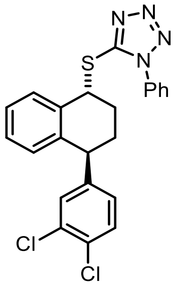

Following **GP12**, **1x** (62 mg, 0.20 mmol, 2.0 equiv.) and 1-phenyl-1*H*-tetrazol-5-thiol (18 mg, 0.10 mmol, 1.0 equiv.) gave **6g** (59%, 5% rsm) as a separable mixture of diastereomers, as a solid. dr = 1.9:1. Data for the major diastereomer: R_f 0.34 [pentane:EtOAc (10:90)]; ^{1}H NMR (400 MHz, $CDCl_3$) δ 7.62–7.48 (6H, m), 7.32 (1H, d, *J* = 8.3 Hz), 7.30–7.16 (2H, m), 7.05 (1H, d, *J* = 2.1 Hz), 6.91 (1H, d, *J* = 7.6 Hz), 6.76 (1H, dd, *J* = 8.3, 2.2 Hz), 5.58 (1H, t, *J* = 3.9 Hz), 4.22 (1H, dd, *J* = 5.9, 3.4 Hz), 2.51 (1H, tdd, *J* = 13.8, 6.0, 2.9 Hz), 2.33 (1H, tdd, *J* = 12.1, 4.6, 2.7 Hz), 2.22 (1H, ddt, *J* = 14.7, 5.9, 3.2 Hz), 1.87 (1H, ddt, *J* = 12.5, 6.0, 3.1 Hz); ^{13}C NMR (101 MHz, $CDCl_3$) δ 153.9, 146.6, 138.4, 134.0, 133.7, 132.4, 130.8, 130.8, 130.5, 130.4, 130.2, 130.1, 129.8, 128.7, 128.0, 127.5, 123.9, 48.0, 43.0, 27.9, 25.0; HRMS (ESI): Found MNa^+ 475.05084, $C_{23}H_{18}ON_4Cl_2NaS$ requires 475.05214.

Data for the minor diastereomer: R_f 0.31 [pentane:EtOAc (10:90)]; ^{1}H NMR (400 MHz, $CDCl_3$) δ 7.64–7.50 (5H, m), 7.47 (1H, dd, *J* = 7.6, 1.6 Hz), 7.39 (1H, d, *J* = 8.2 Hz), 7.30–7.12 (4H, m), 7.00 (1H, dd, *J* = 8.3, 2.1 Hz), 6.81 (1H, d, *J* = 7.7 Hz), 5.59 (1H, t, *J* = 3.5 Hz), 4.05 (1H, t, *J* = 8.6 Hz), 2.56 (1H, dq, *J* = 14.7, 3.5 Hz), 2.40 (1H, ddq, *J* = 12.9, 7.9, 4.2, 3.5 Hz), 2.16 (2H, td, *J* = 9.9, 3.6 Hz); ^{13}C NMR (101 MHz, $CDCl_3$) δ 153.8, 146.4, 139.7, 133.8, 133.6, 132.5, 130.8, 130.6, 130.6, 130.5, 130.2, 130.0, 129.8, 128.6, 128.2, 127.1, 123.9, 48.4, 45.3, 29.3, 28.6. HRMS (ESI): Found MNa^+ 475.05062, $C_{23}H_{18}ON_4Cl_2NaS$ requires 475.05214.

3-(1-(Phenylthio)ethyl)phenyl Ethyl(methyl)carbamate (6h)

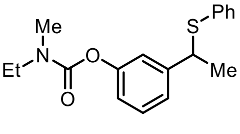

Following **GP10, 1y** (50 mg, 0.20 mmol, 2.0 equiv.) and Ph_2S_2 (22 mg, 0.1 mmol, 1.0 equiv.) gave **6h** (68%, 46% rsm) as an oil. R_f 0.78 [pentane:EtOAc (85:15)]; ^{1}H NMR (400 MHz, $CDCl_3$, rotamers) δ 7.33–7.28 (2H, m), 7.25–7.18 (4H, m), 7.13–7.04 (2H, m), 7.02–6.95 (1H, m), 4.32 (1H, q, *J* = 7.0 Hz), 3.46 & 3.41 (2H, q, *J* = 7.2 Hz), 3.06 & 2.99 (3H, s), 1.62 (3H, d, *J* = 7.0 Hz), 1.24 & 1.19 (3H, t, *J* = 7.2 Hz); ^{13}C NMR (101 MHz, $CDCl_3$, rotamers) δ 154.7, 154.5, 151.7, 144.8, 135.0, 132.8, 129.2, 128.9, 127.4, 124.2, 120.8, 120.7, 47.9, 44.2, 34.4, 34.0, 22.4, 13.4, 12.6; HRMS (ESI): Found MNa^+ 338.11917, $C_{18}H_{21}O_2NNaS$ requires 338.11852.

3-(1-((Trifluoromethyl)thio)ethyl)phenyl Ethyl(methyl)carbamate (6i)

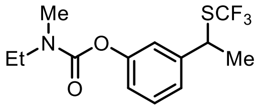

Following **GP11, 1y** (50 mg, 0.20 mmol, 2.0 equiv.) and $AgSCF_3$ (21 mg, 0.1 mmol, 1.0 equiv.) gave **6i** (58%, 22% rsm) as an oil. R_f 0.43 [pentane:EtOAc (95:5)]; ^{1}H NMR (400 MHz, $CDCl_3$, rotamers) δ 7.33 (1H, t, J = 7.9 Hz), 7.18 (1H, d, J = 7.8 Hz), 7.13 (1H, s), 7.06 (1H, d, J = 8.2 Hz), 4.51 (1H, q, J = 7.1 Hz), 3.47 & 3.42 (2H, q, J = 7.2 Hz), 3.07 & 3.00 (3H, s), 1.73 (3H, d, J = 7.1 Hz), 1.25 & 1.20 (3H, t, J = 7.1 Hz); ^{13}C NMR (101 MHz, $CDCl_3$, rotamers) δ 154.5, 154.3, 151.9, 142.74, 130.5 (q, J = 314.8 Hz), 129.7, 123.9, 121.6, 120.6, 44.3, 34.4, 34.0, 23.1, 13.4, 12.6; ^{19}F NMR (565 MHz, $CDCl_3$) δ –40.16; HRMS (EI): Found M^+ 307.08570, $C_{13}H_{16}O_2NF_3S$ requires 307.0848.

3-(1-(Benzo[d]thiazol-2-ylthio)ethyl)phenyl Ethyl(methyl)carbamate (6j)

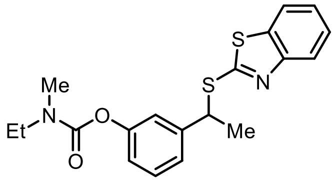

Following **GP10, 1y** (100 mg, 0.40 mmol, 2.0 equiv.) and benzothiazole disulfide (66 mg, 0.2 mmol, 1.0 equiv.) gave **6j** (69%, 42% rsm) as an oil. R_f 0.56 [pentane:EtOAc (85:15)]; ^{1}H NMR (400 MHz, $CDCl_3$, rotamers) δ 7.90 (1H, d, J = 8.2 Hz), 7.73 (1H, d, J = 8.0 Hz), 7.41 (1H, dd, J = 8.3, 7.2 Hz), 7.34–7.23 (4H, m), 7.04 (1H, s), 5.16 (1H, q, J = 7.0 Hz), 3.46 & 3.41 (2H, q, J = 7.2 Hz), 3.06 & 2.99 (3H, s), 1.84 (3H, d, J = 7.0 Hz), 1.24 & 1.20 (3H, t, J = 7.1 Hz); ^{13}C NMR (101 MHz, $CDCl_3$, rotamers) δ 165.8, 154.6, 154.4, 153.4, 151.9, 135.6, 129.5, 126.1, 124.5, 124.4, 121.9, 121.4, 121.1, 120.9, 47.3, 44.2, 39.5, 34.4, 33.9, 22.7, 13.4, 12.6; HRMS (ESI): Found MNa^+ 395.08668, $C_{19}H_{20}O_2N_2NaS_2$ requires 395.08584.

3-(1-((1-Phenyl-1H-tetrazol-5-yl)thio)ethyl)phenyl Ethyl(methyl)carbamate (6k)

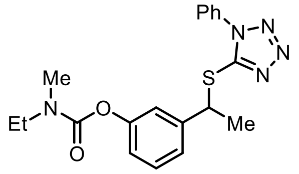

Following **GP12**, **1y** (50 mg, 0.20 mmol, 2.0 equiv.) and 1-phenyl-1H-tetrazol-5-thiol (18 mg, 0.10 mmol, 1.0 equiv.) gave **6k** (79%, 96% rsm) as an oil. R_f 0.38 [pentane:EtOAc (70:30)]; ^{1}H NMR (400 MHz, $CDCl_3$, rotamers) δ 7.54–7.46 (5H, m), 7.32–7.21 (2H, m), 7.16 (1H, s), 7.04 (1H, d, J = 8.0 Hz), 5.21 (1H, q, J = 7.0 Hz), 3.46 & 3.40 (2H, q, J = 6.9 Hz), 3.06 & 2.99 (3H, s), 1.87 (3H, d, J = 7.0 Hz), 1.24 & 1.19 (3H, t, J = 6.8 Hz); ^{13}C NMR (101 MHz, $CDCl_3$, rotamers) δ 154.4, 153.7, 151.9, 142.3,

133.8, 130.2, 129.8, 129.7, 124.2, 124.2, 121.8, 120.8, 47.7, 44.2, 34.4, 34.0, 22.3, 13.4, 12.6; HRMS (ESI): Found MNa$^+$ 406.13029, $C_{19}H_{21}O_2N_5NaS$ requires 406.13204.

(3-(Naphthalen-1-yloxy)-1-phenylpropyl)(phenyl)sulfane (6l)

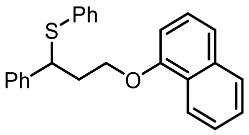

Following **GP10, 1z** (62 mg, 0.20 mmol, 2.0 equiv.) and Ph_2S_2 (22 mg, 0.1 mmol, 1.0 equiv.) gave **6l** (81%, 40% rsm) as an oil. R_f 0.75 [pentane:EtOAc (95:5)]; ^{1}H NMR (600 MHz, $CDCl_3$) δ 8.20 (1H, d, *J* = 7.7 Hz), 7.79 (1H, d, *J* = 7.7 Hz), 7.51–7.45 (2H, m), 7.40 (1H, d, *J* = 8.2 Hz), 7.32–7.16 (11H, m), 6.68 (1H, d, *J* = 7.6 Hz), 4.60 (1H, dd, *J* = 8.9, 6.2 Hz), 4.15 (1H, dt, *J* = 9.3, 5.6 Hz), 3.96 (1H, dt, *J* = 9.2, 4.9 Hz), 2.68–2.59 (1H, m), 2.52–2.43 (1H, m); ^{13}C NMR (151 MHz, $CDCl_3$) δ 154.5, 141.5, 134.9, 134.6, 132.4, 128.9, 128.7, 128.0, 127.6, 127.5, 127.3, 126.5, 126.0, 125.8, 125.3, 122.1, 120.4, 104.8, 65.5, 50.3, 36.1; HRMS (EI): Found M$^+$ 370.13848, $C_{25}H_{22}OS$ requires 370.13859.

2-((3-(Naphthalen-1-yloxy)-1-phenylpropyl)thio)benzo[d]thiazole (6m)

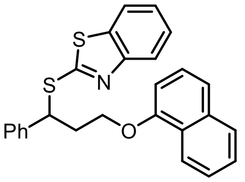

Following **GP10, 1z** (62 mg, 0.20 mmol, 2.0 equiv.) and benzothiazole disulfide (33 mg, 0.1 mmol, 1.0 equiv.) gave **6m** (71 %, 29% rsm) as an oil. R_f 0.54 [pentane:EtOAc (95:5)]; ^{1}H NMR (600 MHz, $CDCl_3$) δ 8.38–8.32 (1H, m), 7.87 (1H, d, *J* = 8.1 Hz), 7.83–7.78 (1H, m), 7.72 (1H, d, *J* = 8.0 Hz), 7.52–7.49 (4H, m), 7.42–7.39 (2H, m), 7.35–7.31 (2H, m), 7.31–7.27 (3H, m), 6.69 (1H, d, *J* = 7.6 Hz), 5.45 (1H, dd, *J* = 9.3, 5.8 Hz), 4.25 (1H, dt, *J* = 9.4, 5.5 Hz), 4.09 (1H, dt, *J* = 9.3, 4.9 Hz), 2.98–2.90 (1H, m), 2.69–2.60 (1H, m); ^{13}C NMR (151 MHz, $CDCl_3$) δ 165.5, 154.5, 153.3, 140.1, 135.6, 134.6, 129.0, 128.1, 128.0, 127.6, 126.5, 126.1, 125.9, 125.8, 125.3, 124.5, 122.3, 122.0, 121.1, 120.5, 104.7, 65.3, 49.8, 36.6; HRMS (ESI): Found MNa$^+$ 450.09573, $C_{26}H_{21}ONNaS_2$ requires 450.09568.

(3-(Naphthalen-1-yloxy)-1-phenylpropyl)(trifluoromethyl)sulfane (6n)

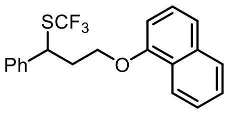

Following **GP11, 1z** (62 mg, 0.20 mmol, 2.0 equiv.) and $AgSCF_3$ (21 mg, 0.1 mmol, 1.0 equiv.) gave **6n** (53%, 5% rsm) as an oil. R_f 0.58 [pentane:EtOAc (98:2)]; ^{1}H NMR (600 MHz, $CDCl_3$) δ 8.27–8.21 (1H, m), 7.83–7.79 (1H, m), 7.54–7.51 (2H, m), 7.43 (1H, d, *J* = 8.3 Hz), 7.37–7.29 (6H, m), 6.67 (1H, d, *J* = 7.6 Hz), 4.80 (1H, dd, *J* = 9.3, 6.1 Hz), 4.18 (1H, dt, *J* = 9.5, 5.3 Hz), 3.97 (1H, dd, *J* = 9.4, 4.6

Hz), 2.75–2.67 (1H, m), 2.56–2.45 (1H, m); ^{13}C NMR (151 MHz, $CDCl_3$) δ 154.3, 139.8, 134.7, 130.6 (q, J = 308.4 Hz), 129.1, 128.3, 127.7, 127.6, 126.6, 125.9, 125.7, 125.5, 121.9, 120.7, 104.8, 64.7, 46.6, 36.6; ^{19}F NMR (565 MHz, $CDCl_3$) δ –39.86; HRMS (EI): Found M^+ 362.09459, $C_{20}H_{17}OF_3S$ requires 362.09467.

5-((1-(Naphthalen-1-yl)ethyl)thio)-1-phenyl-1H-tetrazole (6o)

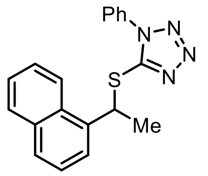

Following **GP12**, **1aa** (72 mg, 0.20 mmol, 2.0 equiv.) and 1-phenyl-1H-tetrazol-5-thiol (18 mg, 0.10 mmol, 1.0 equiv.) gave **6o** (81%, 86% rsm) as a solid. ^{1}H NMR (400 MHz, $CDCl_3$) δ 8.21 (1H, d, J = 8.4 Hz), 7.87 (1H, d, J = 8.0 Hz), 7.82 (1H, d, J = 8.1 Hz), 7.63 (1H, d, J = 7.0 Hz), 7.59–7.42 (8H, m), 6.08 (1H, q, J = 6.8 Hz), 2.10 (3H, d, J = 6.8 Hz); ^{13}C NMR (101 MHz, $CDCl_3$) δ 154.1, 135.6, 134.0, 133.7, 130.7, 130.1, 129.7, 129.3, 129.2, 126.9, 126.2, 125.4, 124.7, 123.9, 123.1, 43.7, 22.0. Data in accordance with literature.[50]

(1-Fluoroethyl)benzene (7a)

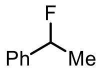

Following GP5, **6d** (28 mg, 0.10 mmol, 1.0 equiv.) and DAST (40 μL 0.30 mmol, 3.0 equiv.) gave **7a** (66%) as an oil. ^{1}H NMR (600 MHz, $CDCl_3$) δ 7.35–7.29 (5H, m), 5.61 (1H, dq, J = 47.8, 6.4 Hz), 1.62 (3H, dd, J = 23.9, 6.4 Hz); ^{13}C NMR (151 MHz, $CDCl_3$) δ 141.4 (d, J = 19.4 Hz), 128.4, 128.2 (d, J = 1.8 Hz), 125.2 (d, J = 6.7 Hz), 90.9 (d, J = 167.1 Hz), 22.7 (d, J = 24.8 Hz); ^{19}F NMR (565 MHz, $CDCl_3$) δ –166.73 (dq, J = 47.8, 23.9 Hz). Data in accordance with literature.[51]

7 Tertiary Radicals Precursors

The amine–borane substrate **S3**, which would lead to a tertiary radical intermediate, was evaluated under our standard conditions. While this species appears to undergo activation via our strategy, no arylation product **S4** was detected (Scheme S2). Instead, we isolated the hydro-deamination product **S5**, indicating that the tertiary radical undergoes premature hydrogen atom transfer (HAT), likely as part of a radical chain propagation involving the amine–borane. Importantly, no formation of **S6** was observed, thereby ruling out oxidation to a tertiary carbocation as the issue.

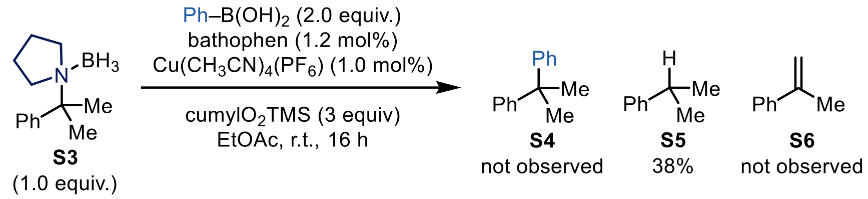

Scheme S2.

8 One-Pot Procedures

We have performed a one-pot reaction (borylation then cross coupling then nucleophilic substitution) using $TsNH_2$, 2-OH-N-Boc-azetidine and benzoic acid as nucleophiles (Scheme S3). In all cases, the desired products **4b**, **5b** and **5c** were formed in yields comparable to the expected overall yield of the stepwise approach with isolation of the intermediate sulfide **6d**.

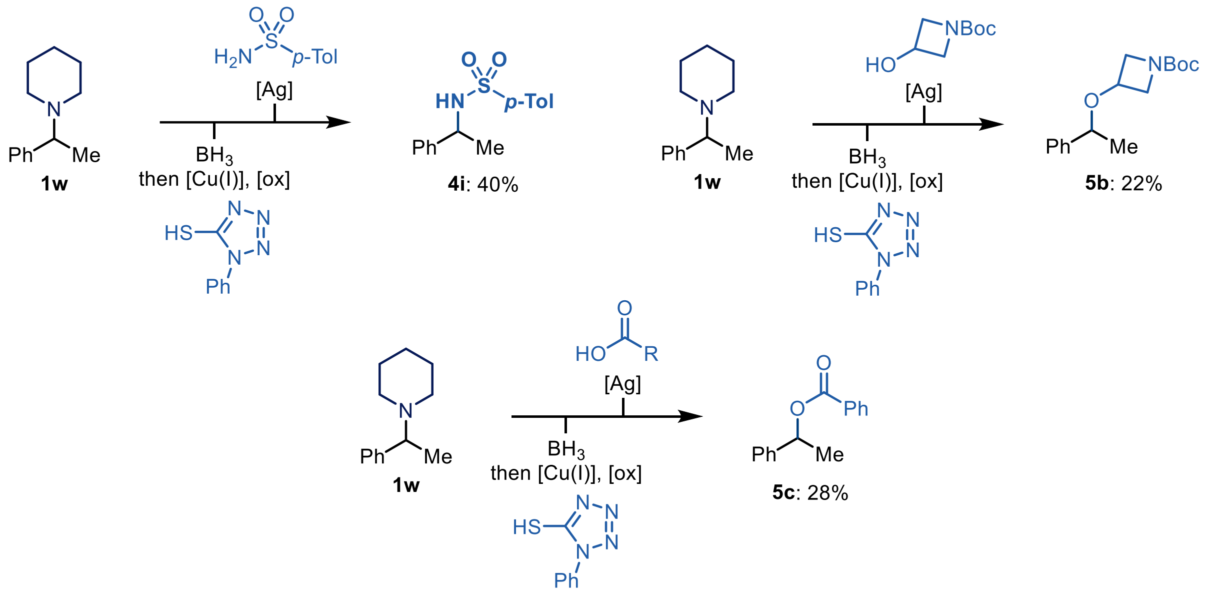

Scheme S3.

9 Laser Flash Photolysis Studies

General Procedure

Laser Flash Photolysis (LFP) experiments were carried out with a laser spectrometer using the third harmonic (355 nm) of a Q-switched Nd:YAG laser, delivering 8 ns pulses. The laser energy was adjusted to 10 mJ/pulse. A 3.5 mL Suprasil quartz cell (10 mm × 10 mm) was used in all experiments. All the experiments were carried out at $T = 25 \pm 0.5$ °C. Argon saturated DTBP:benzene solutions were employed. The observed rate constants (k) were obtained by averaging 3 individual values and were reproducible within 5%. Boryl-radicals have been generated by hydrogen atom transfer (HAT) from the pertinent amine borane precursor with tert-butoxy radical. The observed decay constant ($k_{\beta SC}$) is an average of the measurement at 5 different concentrations (10 mM, 25 mM, 40 mM, 50 mM and 65 mM).

Transient Absorption Spectroscopy Studies

To provide direct evidence of the generation and decay of the boryl radical we performed transient absorption spectroscopy (TAS) studies. The whole process can be described according to Scheme S4.

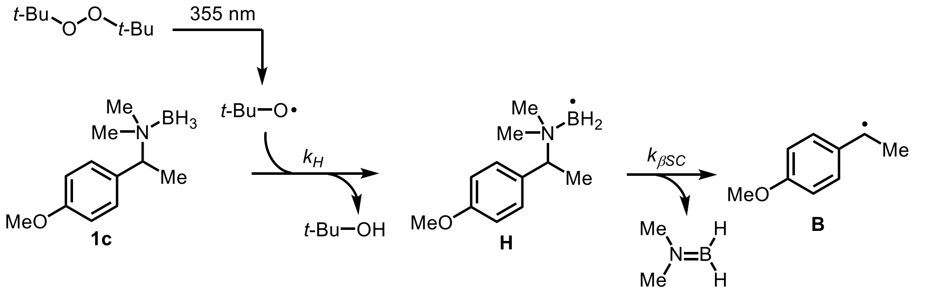

Scheme S4.

In order to measure the rate of β-scission ($k_{\beta SC}$) values, the formation of the boryl radical by HAT (k_H) must be significantly faster (pseudo-first order) than its decay ($k_{\beta SC}$). Since the k_H value measured for the reaction of **1c** with t-BuO$^\bullet$ is 2×10^8 $M^{-1}s^{-1}$ in a range of concentrations between 10–65 mM, a concentration of 25 mM is optimal for determining $k_{\beta SC}$. The reactivity was studies using benzene as the solvent to minimize any other potential reactivity of the boryl radical (e.g. HAT or addition) (Figure **S2**). Hence, the decay trace of the amine-ligated boryl radical **H** (unimolecular process) was used to determine its lifetime that should correspond to its β-scission rate (Figure S3). Under these conditions, $k_{\beta SC} = 2 \times 10^5$ s^{-1}.

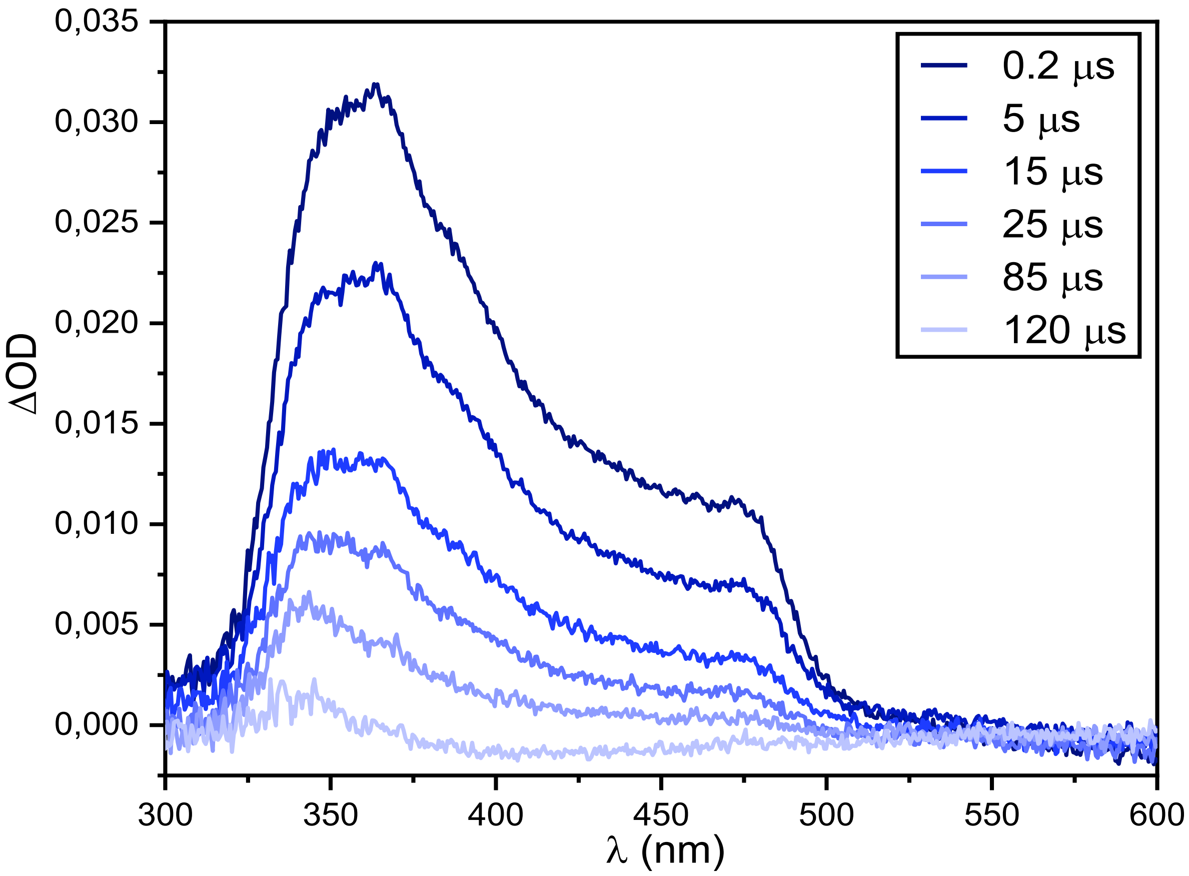

Figure S2. Transient absorbtion spectra of **H** (25 mM) in a 1:1 mixture of DTBP:benzene as solvents at different time intervals under Ar.

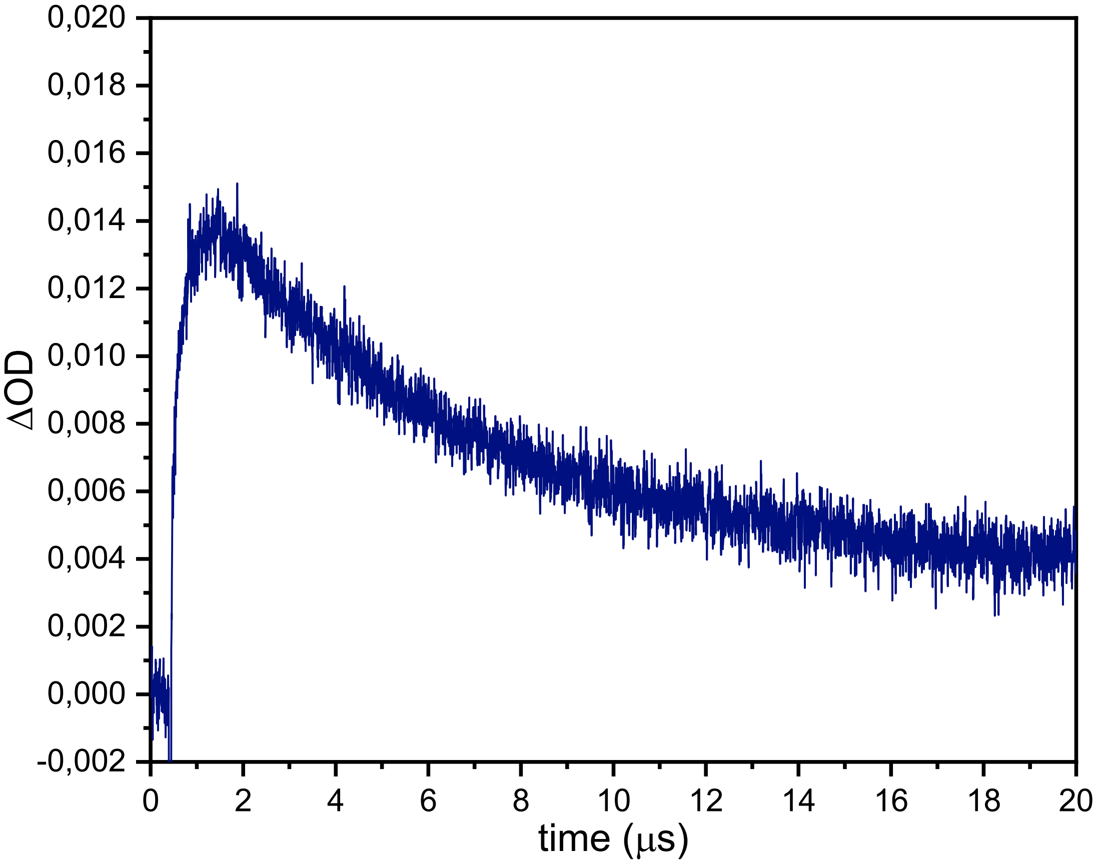

Figure S3. Corresponding decay trace of **H** monitored at 475 nm.

10 UV/Vis Absorption Spectroscopy Studies

The oxidation of [Cu(I)] to [Cu(II)] by cumylO_2SiMe_3 is supported by UV/Vis absorption spectroscopy studies (Figures **S4** & **S5**). The experiments were carried out with $[Cu(CH_3CN)_4]PF_6$ (1.0 equiv.), cumylO_2SiMe_3 (2.0 equiv.), Ph–$B(OH)_2$ (1.0 equiv.) and $(PhS)_2$ (1.0 equiv.) in EtOAc (5 mM). The formation of a new band (λ = 700 nm) upon mixing the [Cu(I)] with the oxidant indicates the generation of a [Cu(II)] species.[52,53]

While these experiments clearly demonstrates that cumylO_2SiMe_3 can oxidize [Cu(I)] to [Cu(II)], they do not give any indication regarding the nucleophile coordination. In our proposed mechanism, Ph–$B(OH)_2$ is proposed to coordinate [Cu(I)] but we cannot exclude a related catalytic cycle where [Cu(I)] oxidation takes place before nucleophile coordination.

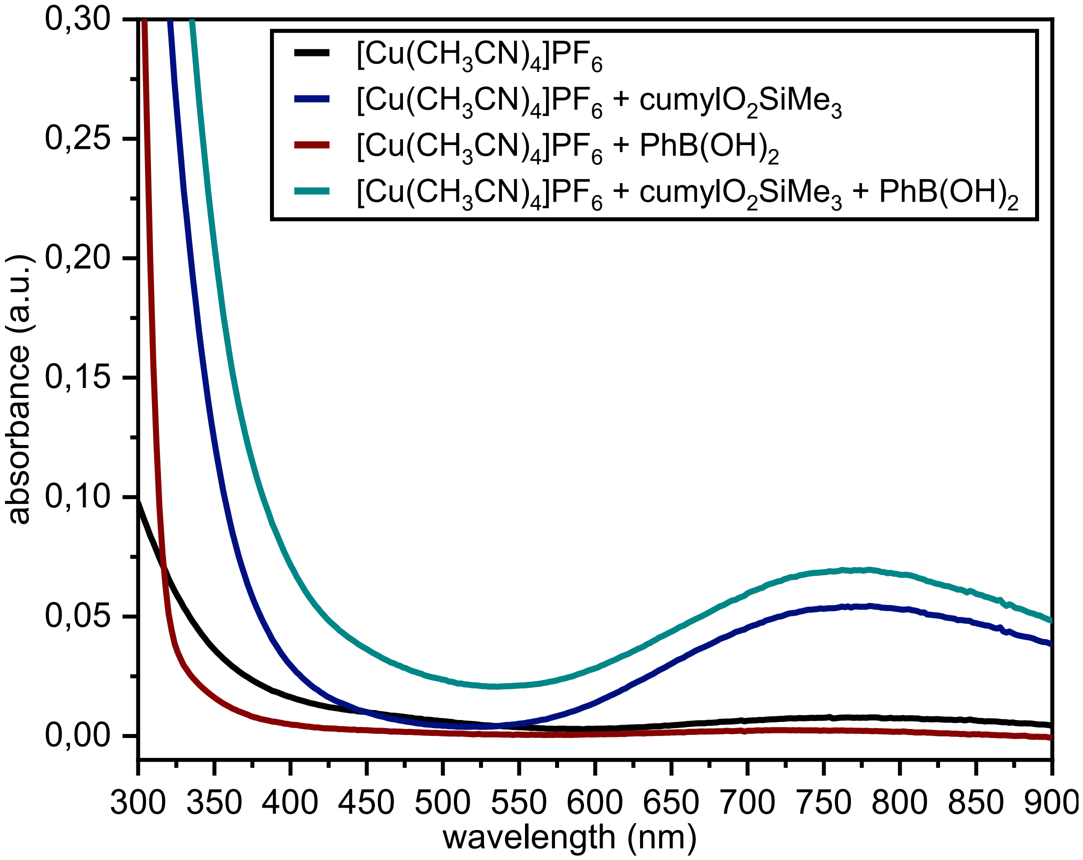

Figure S4.

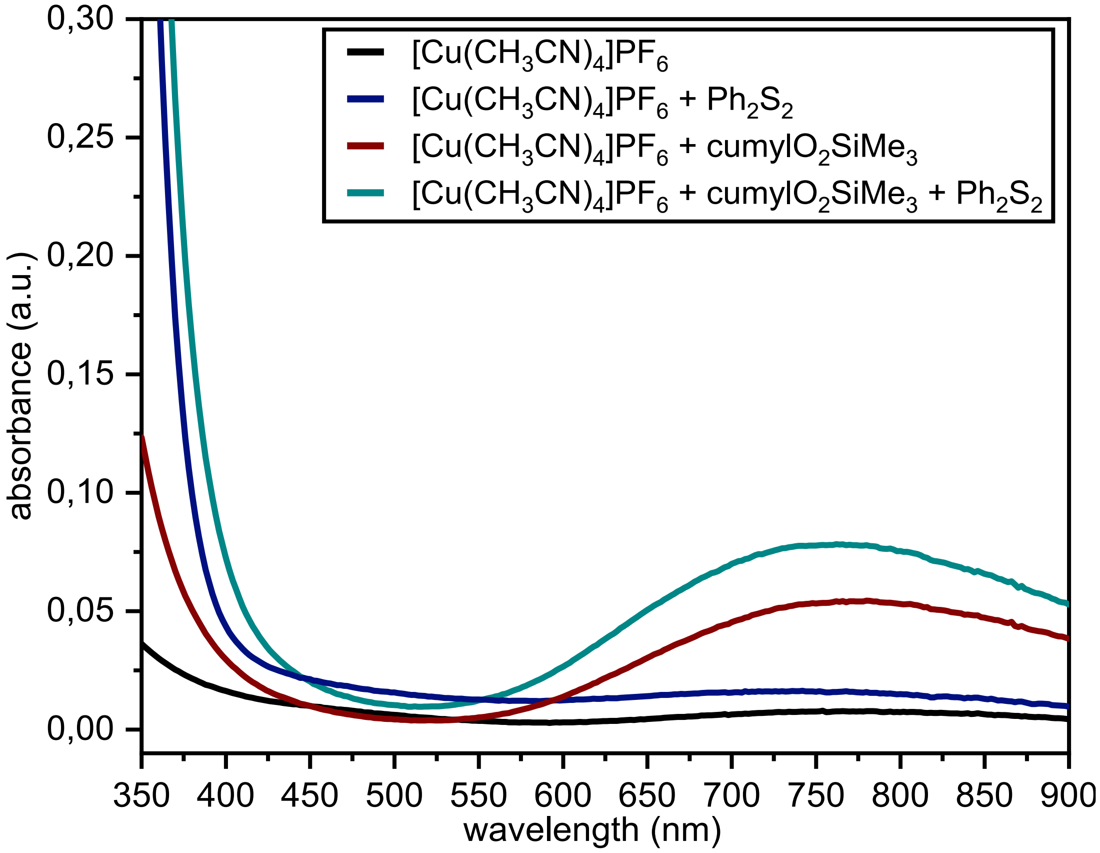

0,30
0,25
0,20
0,15
0,10
0,05
0,00
absorbance (a.u.)
350 400 450 500 550 600 650 700 750 800 850 900
wavelength (nm)
[Cu(CH3CN)4]PF6
[Cu(CH3CN)4]PF6 + Ph2S2
[Cu(CH3CN)4]PF6 + cumylO2SiMe3
[Cu(CH3CN)4]PF6 + cumylO2SiMe3 + Ph2S2

Figure S5.

11 Mechanistic Experiments

To prove the radical nature of the reaction we subjected enantiopure amine borane (*R*)–**S1** to our standard conditions for cyanation, which afforded nitrile **3a** as a racemic mixture (Scheme S6).

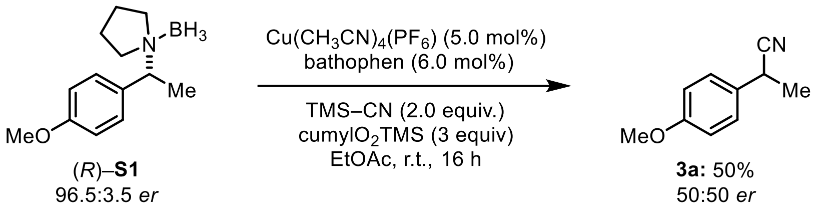

Scheme S6.

HPLC trace for (*R*)–**S1**: Chiralpak® OJ-H, hexane: *i*–PrOH 90:10, flow 1.0 mL/min, oven temperature 30 °C, t_r (minor) =25.33 min and t_r (major) = 33.91 min;

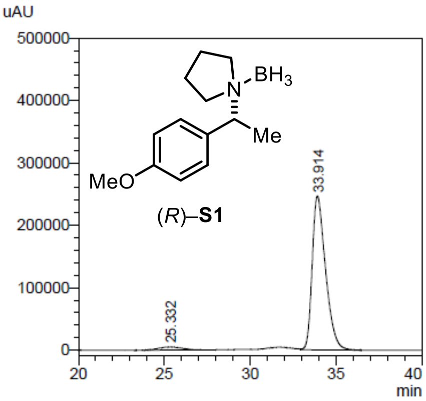

PDA Ch1 225nm

Peak#	Ret. Time	Area	Area%
1	25.332	480123	3.479
2	33.914	13322213	96.521
Total		13802336	100.000

HPLC trace for **3a:** Chiralpak® OJ-H, hexane: *i*–PrOH 99:1, flow 1.0 mL/min, oven temperature 30 °C, t_r = 23.63 min and t_r = 25.99 min;

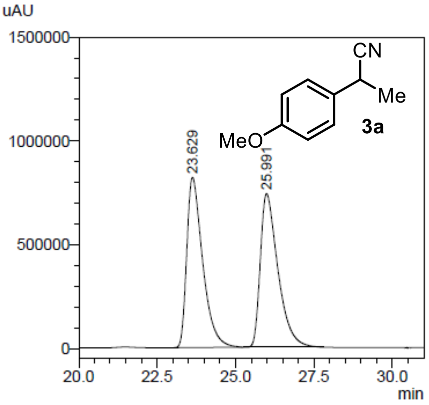

PDA Ch1 225nm

Peak#	Ret. Time	Area	Area%
1	23.629	28890874	49.984
2	25.991	28908880	50.016
Total		57799754	100.000

12 Amine poisoning and stability of amine and amine–borane

To have a better understanding on reaction efficiency we tested the possible catalyst deactivation upon amine coordination by adding different amounts of amine **S2** (Table S4).

Table S4.

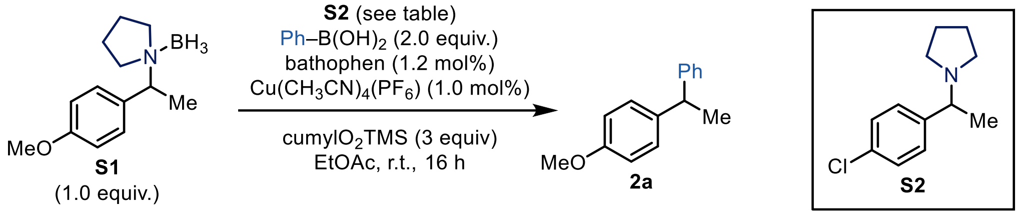

entry	S2 (equiv.)	2a (%)	S1 (%)	1d (%)	S2 recovered (%)	mass balance (%)
**1**	0	80	12	–	–	92
**2**	0.1	65	30	–	–	95
**3**	0.25	51	39	–	–	90
**4**	0.5	32	59	–	–	91
**5**	1.0	16	77	–	–	93

We have further evaluated the stability of **S2** under various conditions to understand the most likely decomposition pathway (Table S5).

Table S5.

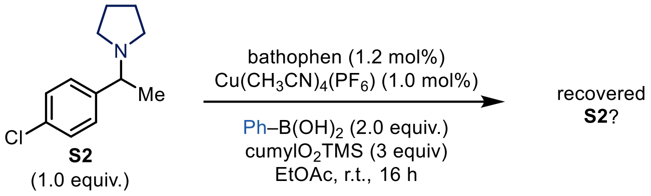

entry	Variations	S2 (%)
**1**	none	–
**2**	no [Cu(I)]	75
**3**	no bathophen	–
**4**	no Ph–B(OH)$_2$	–
**5**	no CumO$_2$SiMe$_3$	80
**6**	no [Cu(I)] and no bathophen	91
**7**	No Cu and no CumO$_2$SiMe$_3$	>95
**8**	Only CumO$_2$SiMe$_3$	70

Regarding the possibility of the amine to coordinate the [Cu(I)], UV/Vis studies confirm that this can happen (Figure S6).

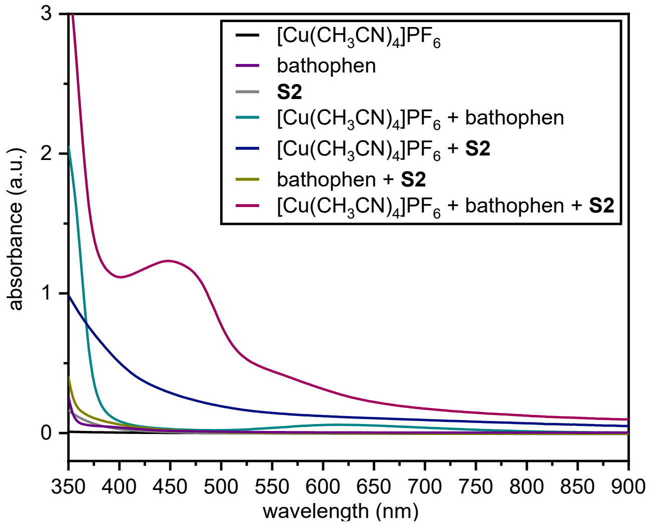

Figure S6.

We also tested the stability of amine boranes toward possible BH_3-decomplexation followed by amine decomposition mediated by [Cu(I)] and the oxidant. However, the amine-boranes are stable under the reaction conditions (without Ph–$B(OH)_2$) and also in the presence of large excess of the potential by-products generated during the SET (TMSO$^-$; we used LiOH in the experiments below) and HAT (cumyl–OH) steps (Table S6).

Table S6.

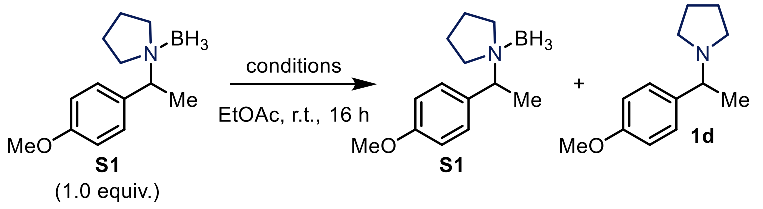

Entry	Conditions	S1 (%)	1d (%)
**1**	cumylOH (10 equiv.)	90	10
**2**	LiOH (10 equiv.)	91	9
**3**	cumylO_2TMS	100	0

13 Computational Studies

13.1 General Details

A conformational analysis was completed with *autodE* due to the large number of rotatable bonds in the transition state.[54] From the SMILES strings of both reactants and products, the program identifies all possible conformers to ouput the transition state geometry at the PBE0/def2-SVP level of theory in gas phase.[55,56] This geometry was then used for the initial guess of the geometry optimization at the chosen level of theory. When *autodE* did not converge to the desired initial guess, a scan of the relevant fragmented bond was completed instead.

Density Functional theory (DFT)[57,58] calculations carried out employed the computational chemistry package ORCA (version 5.0.4).[59] The geometry optimization and vibrational frequency calculations were run, unless otherwise stated, using the Head-Gordon ωB97X functional,[60] combined with an atom-pairwise dispersion correction with the zero-damping scheme (D3)[61] and the Karlsruhe def2-SVP basis set in gas phase. The self-consistent field (SCF) convergence was set by the 'TightSCF' keyword, while the integration grids were set by the keyword 'defgrid3' to increase numerical precision. Moreover, the default settings of this version of ORCA included the def2/J auxiliary basis set[62] and the RIJCOSX approximation in all calculations. The potential energy minima were confirmed to show all-real vibrational frequencies and the transition states to display one imaginary frequency, corresponding to the reaction normal mode. Intrinsic reaction coordinate (IRC) calculations[63] were performed to confirm the connection between reactants and products to their respective saddle point.

More accurate single point energy calculations (SPC) diverged from the conditions above in the larger def2-TZVP basis set[56,62] and including, when stated, solvent effects (EtOAc) with the implicit universal solvent model (SMD).[64] The selection of the level of theory applied in the studies is explained in Section 13.2. The thermodynamic data was extracted from the output files using the python script OTHERM.py[65] where the thermodynamic contributions from the vibrational analysis were combined with the SPC calculations. The Gibbs free energies, corrected for the standard state of 1 M, were obtained at 298.15 K.

Non-covalent interactions (NCIs) analysis[66] was conducted for selected examples using Multifwn[67,68] (version 3.7), with the corresponding isosurfaces images and electron density plots done with VMD[69] (version 1.9.4) and gnuplot (version 5.2)[70].

Cartesian coordinates of all structures discussed, including their thermodynamic data, can be found in the Supplementary Material attached.

13.2 Level of Theory Selection

The level of theory was selected by comparing the energy barrier ($\Delta G^{\ddagger}$) magnitude derived computationally to the experimentally measured one for **H** (k_{obs} = 2 10^5 s^{-1}, see Section 8).

The rate constant was subjected to the Eyring equation (Eq. 1) to quantify the Gibbs free energy of activation ($\Delta G^{\ddagger}$) in kcal mol^{-1}.

$$\Delta G^{\ddagger} = -\mathrm{RT}\ln\left(\frac{kh}{k_B T}\right) \qquad \text{(Eq. 1)}$$

Where R is the gas constant (8.31 J K^{-1} mol^{-1}), T is the temperature (298.15 K), k is the rate constant measured by transient spectroscopy (s^{-1}), and k_B is the Boltzmann constant (1.38×10^{-23} J K^{-1}). The transmission coefficient (k) was assumed to be 1.[71]

Using equation 1, the experimental $\Delta G^{\ddagger}$ value was determined to be approximately 10.22 kcal mol^{-1}. To find an appropriate level of theory, the energy barrier of the same process was benchmarked by employing functionals[72–76] used to describe reactions involving boryl radicals, including functionals known to effectively describe radical processes.[77–83] The calculated barriers were then compared to the experimentally measured value (Table S7). The def2-SVP basis set was chosen with its larger option (def2-TZVP) for the SPC as it is often found in literature in conjunction with the functionals tested.

Table S7. Computational benchmark testing different functionals. Geometry optimization was attempted in gas phase, followed by a single point energy calculation including solvent effects in benzene and/or EtOAc (*). The calculated activation barrier ($\Delta G^{\ddagger}$) and the Gibbs Free Energy change (ΔG^{0}) of the β-scission process are displayed. The final column shows the difference ($\Delta G^{\ddagger}_{exp}–\Delta G^{\ddagger}_{comp}$) between the computational and experimental values calculated by transient absorption spectroscopy (in benzene).

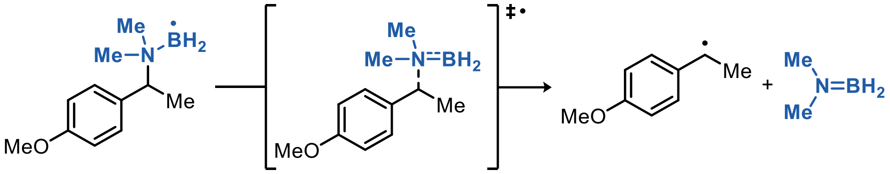

Functional	$\Delta G^{\ddagger}$ (kcal mol^{-1})	ΔG^{0} (kcal mol^{-1})	$\Delta G^{\ddagger}_{exp}–\Delta G^{\ddagger}_{comp}$ (kcal mol^{-1})
ωB97X-D3	7.7	-41.9	2.5
ωB97X-D3*	8.6	-39.3	
ωB97M-V	7.3	-38.9	2.9
ωB97M-V*	8.1	-36.4	
B3LYP	1.8	-54.2	8.4
B3LYP*	2.4	-51.8	
CAM-B3LYP	6.0	-49.3	4.2
CAM-B3LYP*	6.8	-46.7	
B3LYP-D3BJ	2.4	-43.9	7.9
B3LYP-D3BJ*	2.9	-41.5	
M06-2X	7.5	-35.6	2.7
M06-2X*	8.5	-33.2	

Across the table, we see that the B3LYP-based functionals significantly underestimate the barrier, thereby increasing the error, whereas the M06-2X and the ωB97X functionals are the best performing. To streamline the computational efforts, the functional requiring numerical frequency (ωB97XM-V) was discarded. The M06-2X and ωB97X-D3 functional were identified as the most appropriate to describe the fragmentation studied, and both can be employed. The ωB97X-D3 functional was then decided to move forward with the computational studies due to having the lowest $\Delta G^{\ddagger}_{exp}–\Delta G^{\ddagger}_{comp}$ value.

13.3 Nomenclature

The labelling of the geometries is based on the substrate and the point along the reaction coordinate (see Figure S7). The naming can be either the reagent radical after HAT (R), the saddle point indicating the C–Y bond is cleaved with the simultaneous Y=X double bond formation (TS), and the products resulting from the fragmentation (P-1 and P-2) treated at an infinite distance.

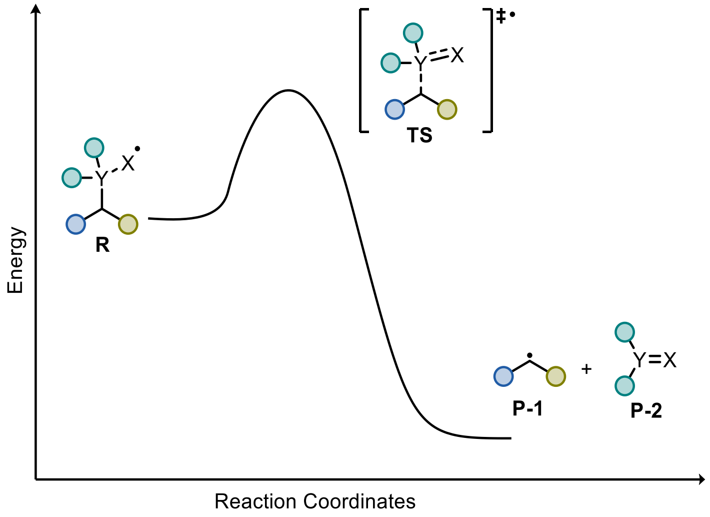

Figure S7. Potential energy surface example to indicate the nomenclature of the geometries.

The second part of the nomenclature uses the labelling illustrated below to describe the nature of the compound investigated (Figure S8). The compounds upon radical generation are portrayed to facilitate the graphical illustration of the numbering system.

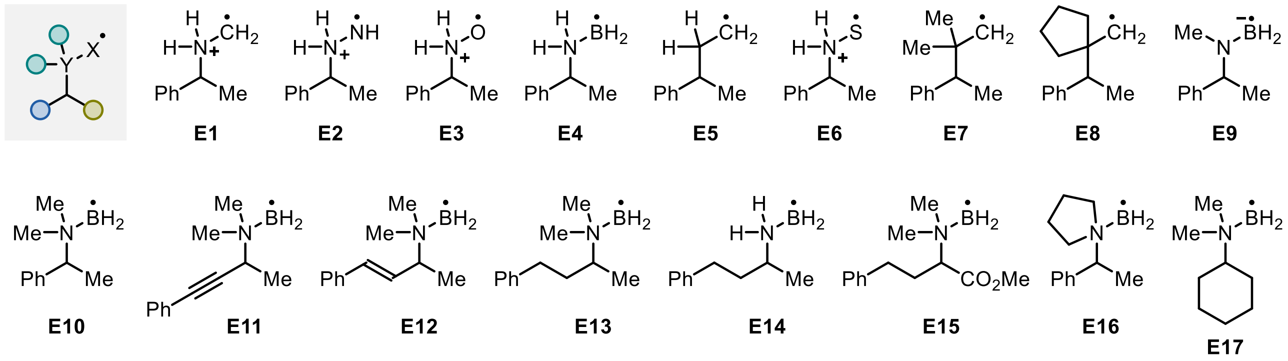

Figure S8. Numerical labelling of compound analyzed.

13.4 Thermodynamics

β-Scission involving heteroatomic radicals

To showcase the unique properties of amine-ligated boryl radicals and the effects of the B=N double bond formation on the thermodynamic parameters, different systems were selected (**E1-3**, **E5-9**) and compared to model species **E4** and **E10**. As shown in Table S8, amine-ligated boryl radicals **E4** and **E10** features the lower activation barrier ($\Delta G^{\ddagger}$) and the most exergonic (ΔG^0) fragmentation. Both $\Delta G^{\ddagger}$ and ΔG^0 increase significantly in the other systems. This comparison is conducted in gas phase to highlight the advantages of the amine-ligated boryl radical in the specific β-scission step of the mechanism. As the thermodynamic data does not include the effects of solvation and other external factors, the values do not reflect the *absolute* experimental kinetic and thermodynamics of the process but they describe the *relative* reactivity of the series.

Table S8. Thermodynamic data in the gas phase to compare the leaving group's impact on the feasibility of the β-scission. The calculated activation barrier ($\Delta G^{\ddagger}$) and the Gibbs Free Energy change (ΔG^0) of the fragmentation process are displayed.

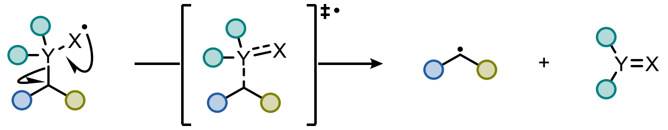

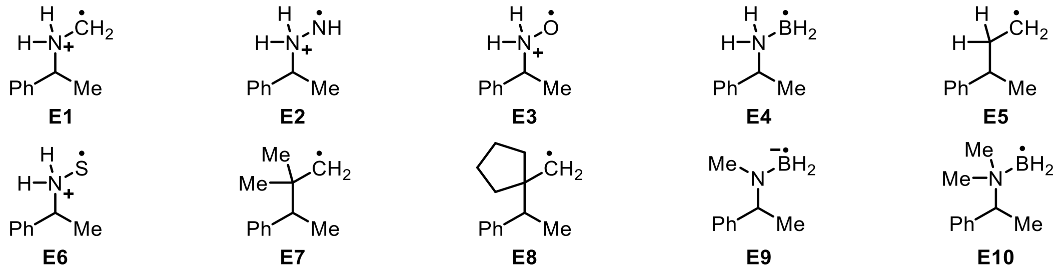

Compound	$\Delta G^{\ddagger}$ (kcal mol^{-1})	ΔG^0 (kcal mol^{-1})
**E1**	8.2	6.5
**E2**	-	25.7
**E4**	4.2	-44.2
**E5**	22.4	0.6
**E7**	19.9	-7.2
**E8**	23.2	-5.8
**E9**	7.6	-0.7
**E10**	5.6	-44.9

For system **E2**, a transition state guess could not be determined as while scanning the C–N bond until cleavage (> 3 Å), a saddle point was not observed therefore suggesting a barrierless process. For species **E3** and **E6,** the ΔG^0 indicates a significant endergonic value (Figure S9). When investigating the Mulliken atomic spin density at the transition state initial guess geometry (O = 0.76 and S = 0.78), the density is primarily localized at the heteroatom rather than spread across the forming benzyl radical. Hence, it is suggested that species **E3** and **E6** do not undergo a radical fragmentation, but an ionic rearrangement leading to the unpaired electron likely located in the π* orbital of the N=Y double bond. When recalculating the thermodynamics with the appropriate fragmented products, the ΔG^0 indicates a feasible process. The variation in reactivity in species **E3** and **E6** highlights the advantages of amine-borane complexes (**E4** and **E10)**.

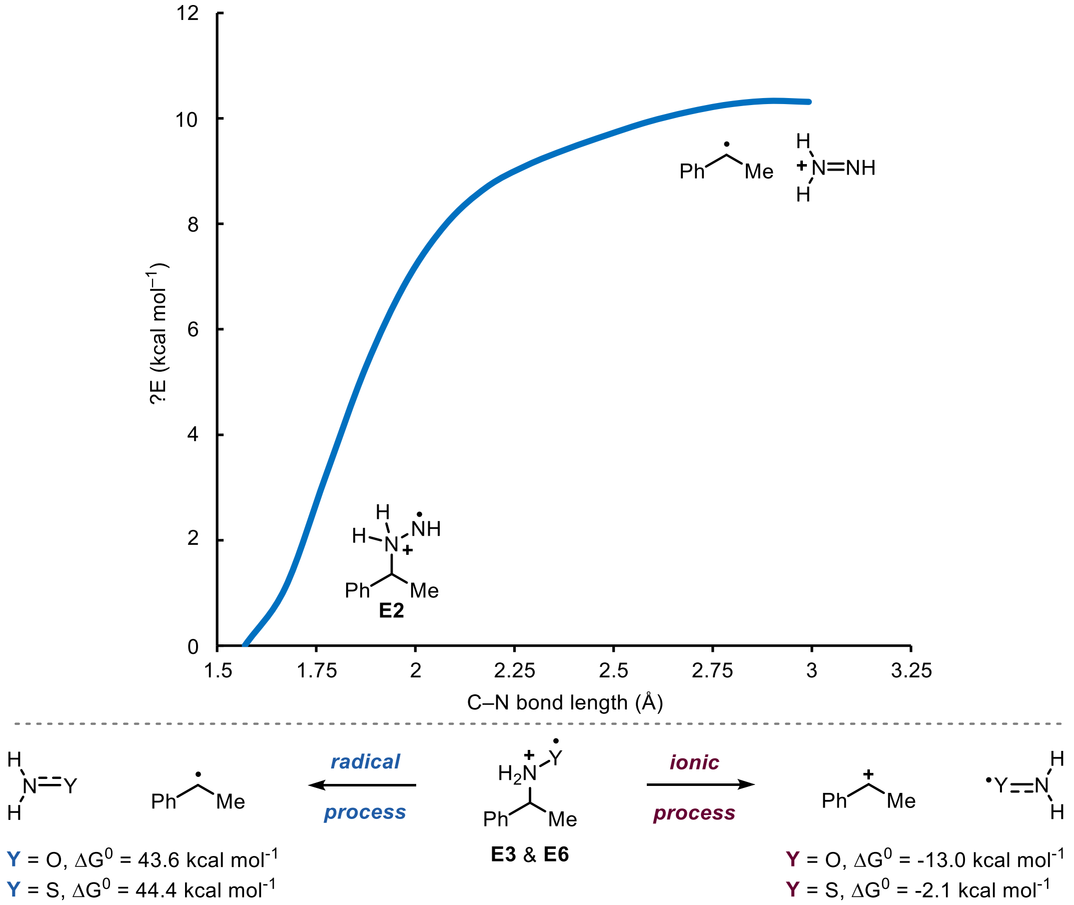

Figure S9. Outliers found in comparison studies to species **E4** and **E10**.

β-Scission of amino boryl radicals: substituent effects

The thermodynamics of the β-scission process were analyzed through the activation barrier ($\Delta G^{\ddagger}$) and the Gibbs free energy change (ΔG^0). They were determined for different examples to investigate the effects of the substitution pattern on the carbon and nitrogen (Table S9). The β-scission process for all compounds is exergonic ($\Delta G^0 < 0$ kcal mol^{-1}). Activated species (**E10–E12**) favor the β-scission process (approx. $\Delta G^0 \leq$ -40 kcal mol^{-1}) with a barrier of less than 10 kcal mol^{-1}. In fragmentations leading to less stable carbon radicals (**E13, E14** and **E17**), although with a negative ΔG^0 indicating feasibility, the value is largely more positive than other species ($\Delta\Delta G^0 \approx$ 12–22 kcal mol^{-1}). Moreover, the $\Delta G^{\ddagger}$ values for these substrates increase by 4–5 kcal mol^{-1}, resulting in a more challenging β-scission due to a higher energy transition state. The substitution pattern at the nitrogen does not contribute to lowering the barrier (**E13** and **E14**). Nevertheless, adding an electron-withdrawing group (**E13** and **E15**) seems to stabilize the newly formed C radical, therefore improving the energetics. Model substrate **E16** presents a lower barrier than **E10** ($\Delta\Delta G^{\ddagger} \approx$ 1 kcal mol^{-1}), with a similar negative ΔG^0 value.

Table S9. Thermodynamic data in EtOAc to compare the substituent effects, both on the fragmented radical and on the nitrogen, on the feasibility of the β-scission step. The calculated activation barrier ($\Delta G^{\ddagger}$) and the Gibbs Free Energy change (ΔG^0) of the fragmentation process are displayed.

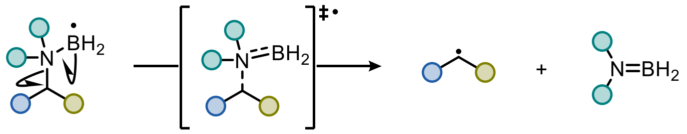

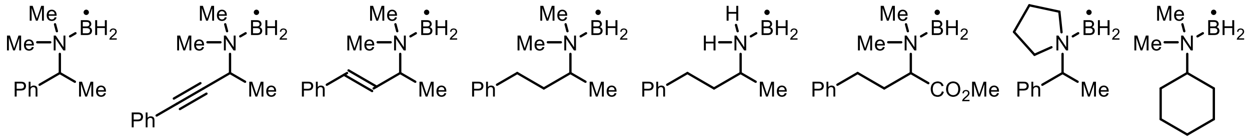

E10 **E11** **E12** **E13** **E14** **E15** **E16** **E17**

Compound	$\Delta G^{\ddagger}$ (kcal mol^{-1})	ΔG^0 (kcal mol^{-1})
**E10**	8.4	-39.7
**E11**	7.6	-41.7
**E12**	7.1	-45.7
**E13**	13.0	-27.4
**E14**	13.0	-22.9
**E15**	8.4	-40.0
**E16**	7.4	-38.6
**E17**	14.4	-26.5

The advantage of cyclic over non-cyclic amines in the β-scission step

As aforementioned, the pyrrolidine-substituted amino-boryl radical (**E16**) presents a lower energy barrier over the acyclic dimethylamino borane species (**E10**) in the reaction solvent ($\Delta\Delta G^{\ddagger} \approx 1$ kcal mol^{-1}). To explain this observation, an analysis of the non-covalent interactions (NCIs) was conducted: NCIs describes a wide range of bonding and nonbonding interactions through the electron density, from weaker forces like van der Waals, to stronger ones like H-bonding or steric repulsion.[84–87] In the case of substrates **E10** and **E16**, we aimed to analyse the key differences in weak interactions, whether attractive or repulsive forces.[88,89]

The NCIs analysis is displayed in two formats. Firstly, a scatter plot of the reduced density gradient (RDG) *versus* the electron density (ρ), multiplied by the sign of the second Hessian eigenvalue (λ_2), to quantify and determine the type of interaction present within the species.[66] The low RDG spike regions (RDG < 0.48 a.u.) indicate significant NCIs present within the species. Depending on the sign (whether positive or negative) and magnitude of the sign(λ_2)ρ value, the type of interaction can be charaterized (Figure S10): low density values describe weak van der Waals interactions, while higher densities identify either attractive forces (e.g. H-bonding) or steric repulsion. The scatter plot can be converted visually to a NCI map in the system's real space, to display the gradient isosurfaces, matching the colour code of the sign(λ_2)ρ value, revealing their location.

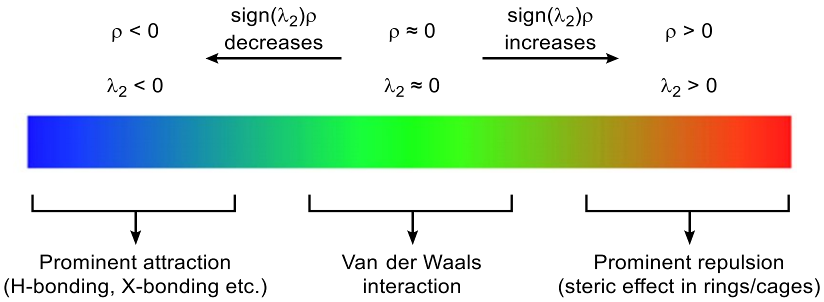

Figure S10. Colour-coded bar to identify the type of weak interaction according to the sign(λ_2)ρ value.

The NCIs study was conducted across the PES of the β-scission reactions, especially at the boryl radical stage and transition state level as the early stage of the fragmentation dictates the thermodynamics. When comparing the β-scission process between **E10** and **E16**, a significant difference is oberserved in the orientation of the amino boryl motif. In species **E10**, the $-CH_3$ groups bonded to *N* are located above the phenyl ring, whereas the pyrrolidine is approximately rotated 90° compared to the arene. The rotated conformation of **E16** is likely adopted to alleviate the steric clash between the two rings, as the conformation with the same orientation as **E10** is higher in energy ($\Delta\Delta G \approx 2.9$ kcal mol^{-1}).

Firstly, the NCIs were evaluated for the amino boryl radical species through the scatter plots (Figure **S11**). When looking at the lower RDG region (RDG < 0.48 a.u.), spike regions are observed indicating the presence of weak interactions. In both **E10** (Figure **S11a**) and **E16** (Figure **S11b**), similar patterns are seen in the Van der Waals bonding ($sign(\lambda_2)\rho \approx -0.01$) and nonbonding ($0.005 < sign(\lambda_2)\rho < 0.015$) regions; the same observation can be done for the steric repulsion ($sign(\lambda_2)\rho \approx 0.02$). The scatter plot of **E16** presents a new spike region at approximately 0.04 a.u. that is assigned to the steric cage of the pyrrolidine, where the atoms interact due to close proximity, but are not directly bonded. This steric crowding phenomenon is found in all scatter graphs for the **E16** series.

When comparing the NCI maps of the radical system, two major differences in the isosurfaces between **E10** and **E16** can be distinguished. Although present in both cases, species **E16** displays stronger interactions, a combination between van der Waals and steric repulsion, below the $–BH_2$ group with the $–CH_3$ and phenyl ring. Secondly, an isosurface above the $–BH_2$ group indicates an interaction with the pyrrolidine that is not observed in **E10**. This is reflected in the atomic distances and angles (Table S10). While the B–C distances and corresponding angles are analogous in both **E10** and **E16** for the C1 and C2, in **E16** the atomic distances are significantly shorter than expected for C3 and C4 (≈ 3 Å), further evidenced by the almost 90° bond angle ($\theta_{B–N–C3/4}$).[90] Together, these findings indicate a steric repulsion between the $–BH_2$ and pyrrolidine caused by the envelope conformation of the latter.

Table S10. Atomic distances (Å) and degree angles (θ) for the optimized geometry of the amino boryl radical **E10** and **E16**.

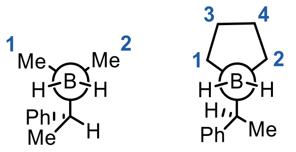

E10 **E16**

Species	B–C1 / Å	B–C2 / Å	B–C3 / Å	B–C4 / Å	$\theta_{B–N–C1}$ /°	$\theta_{B–N–C2}$/°	$\theta_{B–N–C3}$ /°	$\theta_{B–N–C4}$ /°
**E10**	2.52	2.49	-	-	108.6	106.8	-	-
**E16**	2.53	2.52	3.01	3.04	109.0	108.2	95.5	96.3

a.

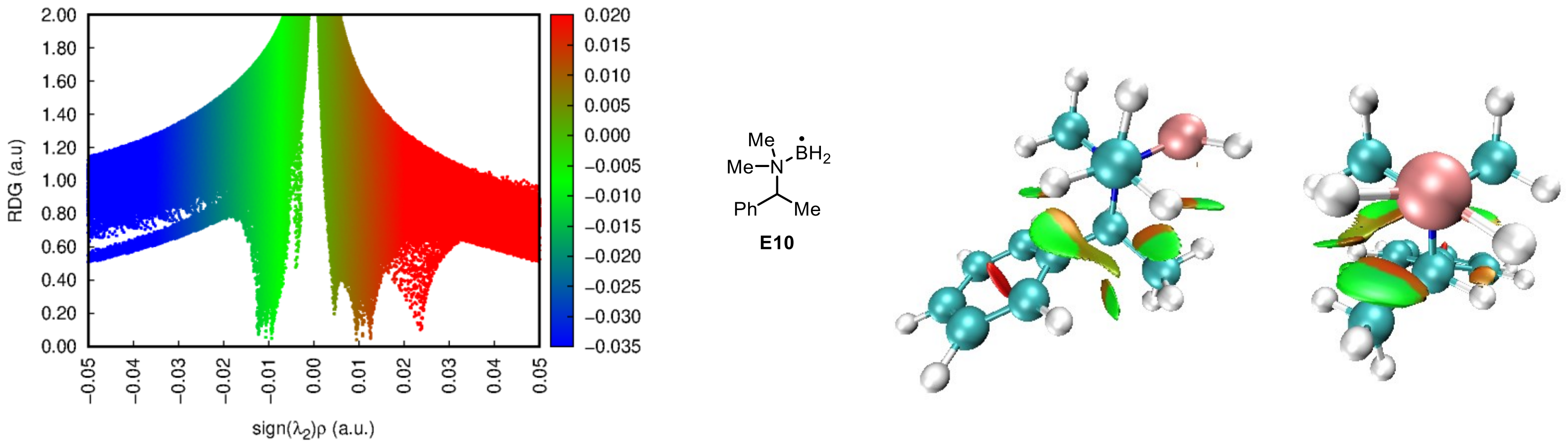

b.

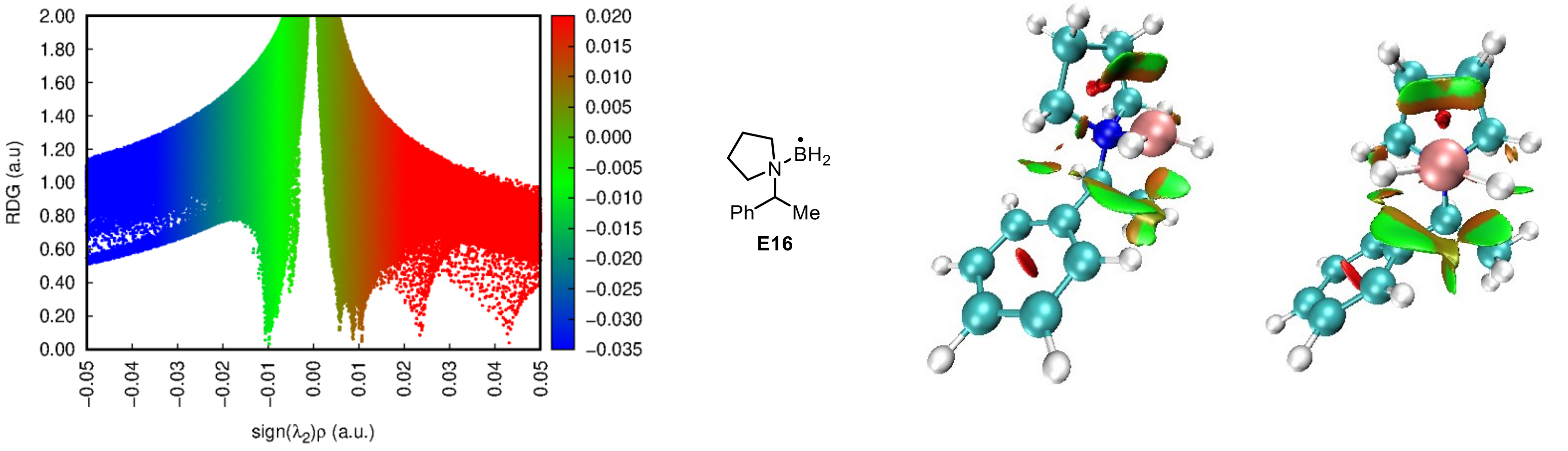

Figure S11. NCI scatter plot and map of amino boryl radical species **E10** (part a) and **E16** (part b) in EtOAc. Isosurface value = 0.48. carbon = light blue; hydrogen = white; boron = pink; nitrogen = dark blue.

The NCIs analysis was then extended to the transition state of the β-scission step (Figure S12). When comparing the scatter plot, an extra spike region is observed when $\mathrm{sign}(\lambda_2)\rho \approx 0.015$ in species **E10** compared to **E16**. Moreover, the NCIs isosurface map in the transition state of **E16** shows the loss of the steric repulsion between the $–BH_2$ and the pyrrolidine due to the gradual flattening of the amino boryl group to form the by-product after the C–N bond cleave. This is as well reflected in the increase in bond angle from boryl radical species **E16** to its transition state ($\bar{x}_\theta =$ 95.9 → $\bar{x}_\theta =$ 103.3).

Table S11. Atomic distances (Å) and degree angles (θ) for the optimized geometry of the transition state for **E10** and **E16**.

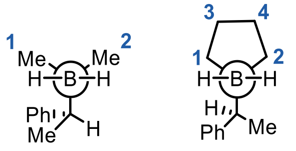

TS10 **TS16**

Species	$B–C_1$ / Å	$B–C_2$ / Å	$B–C_3$ / Å	$B–C_4$ / Å	$\theta_{B–N–C1}$ /°	$\theta_{B–N–C2}$/°	$\theta_{B–N–C3}$ /°	$\theta_{B–N–C4}$ /°
**E10**	2.48	2.47	-	-	112.9	112.5	-	-
**E16**	2.49	2.49	3.09	3.11	113.1	113.2	102.8	103.8

Hence, it is proposed that **E16** presents a lower energy barrier of the β-scission thanks to the strain release that occurs during the fragmentation. The initial rotated conformation of **E16** compared to **E10** avoids stronger repulsion with the phenyl ring, with the envelope conformation of the pyrrolidine resulting in steric clash with the $–BH_2$ group. As the β-scission progresses, the flattening of the pyrrolidine to produce the aminoborane by-product results in the decrease in steric repulsion, effectively lowering the energy barrier. In the preferred conformation of **E10**, no steric clash between the methyl groups on the nitrogen and the $–BH_2$ is seen. Furthermore, at the transition state the flattening of the amino boryl group may result in the slight increase in steric repulsion between the methyl groups and the phenyl ring, as higher steric repulsion interactions were visible in the RDG plot. These differences are suggested to cause the $\Delta\Delta G^{‡} \approx 1$ kcal mol^{-1} gap between acyclic dimethylamine and pyrrolidine borylated species.

a.

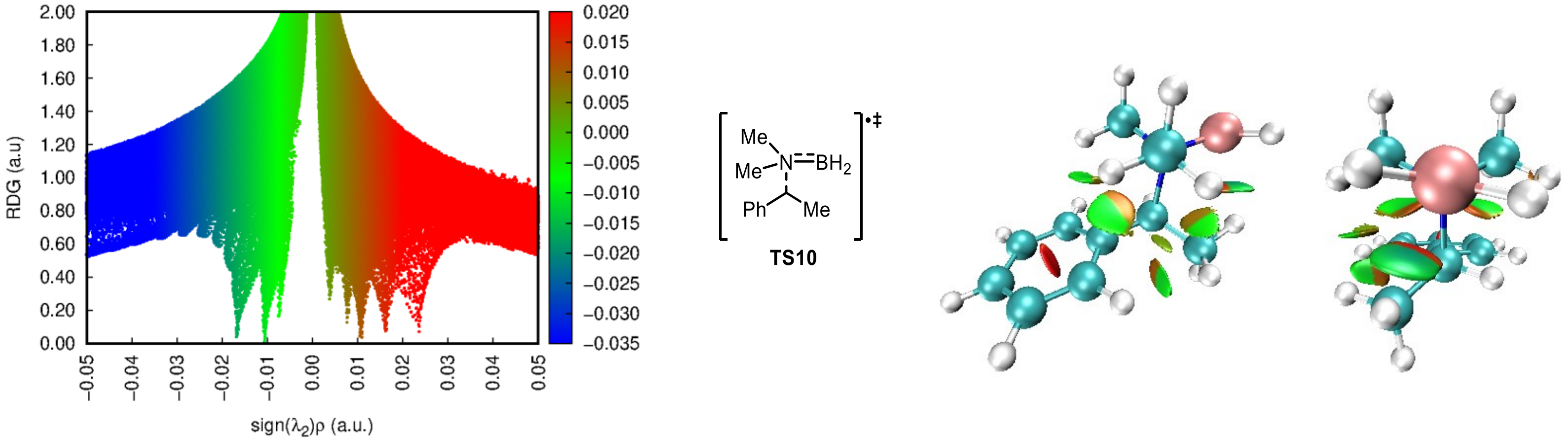

b.

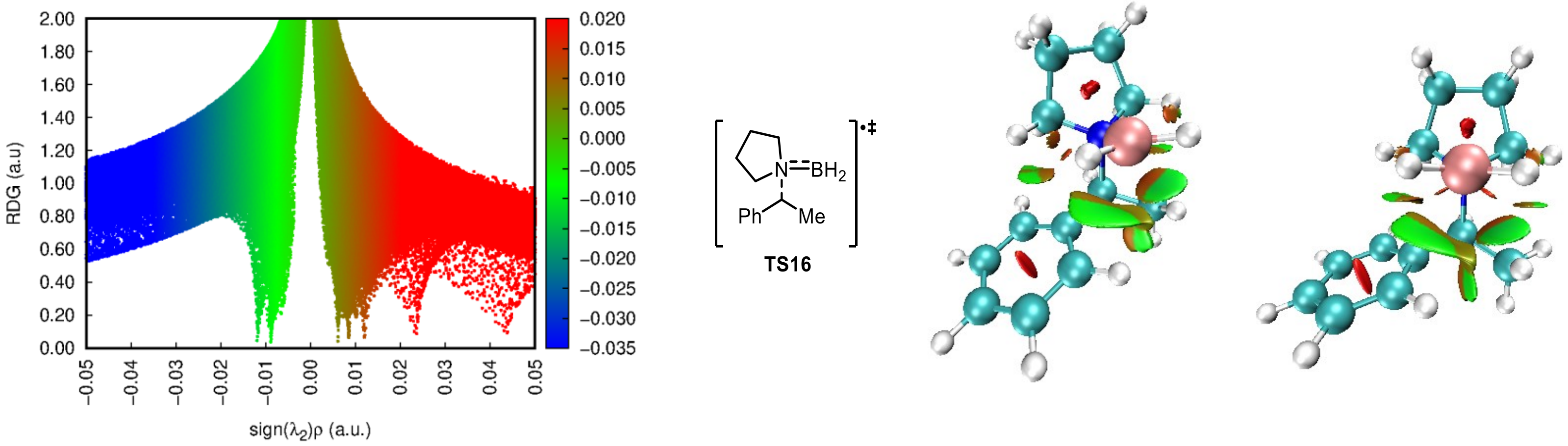

Figure S12. NCI scatter plot and map of the transition state of amino boryl radical species **E10** (part a) and **E16** (part b) in EtOAc. Isosurface value = 0.48. carbon = light blue; hydrogen = white; boron = pink; nitrogen = dark blue.

14 NMR Spectra

S1 – ^{1}H NMR (600 MHz, $CDCl_3$)

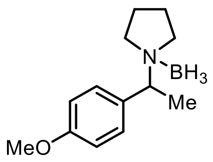

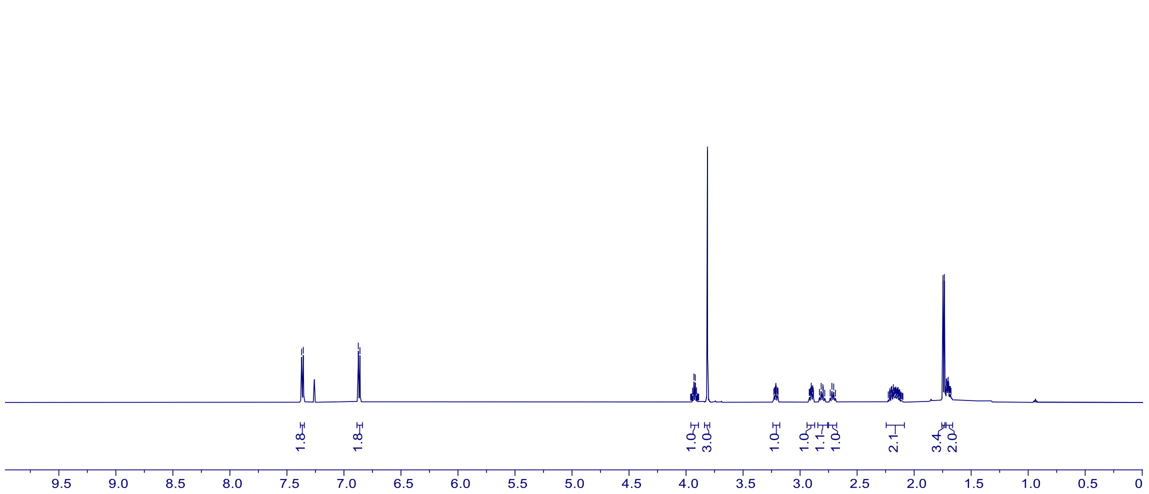

S1 – ^{13}C NMR (151 MHz, $CDCl_3$)

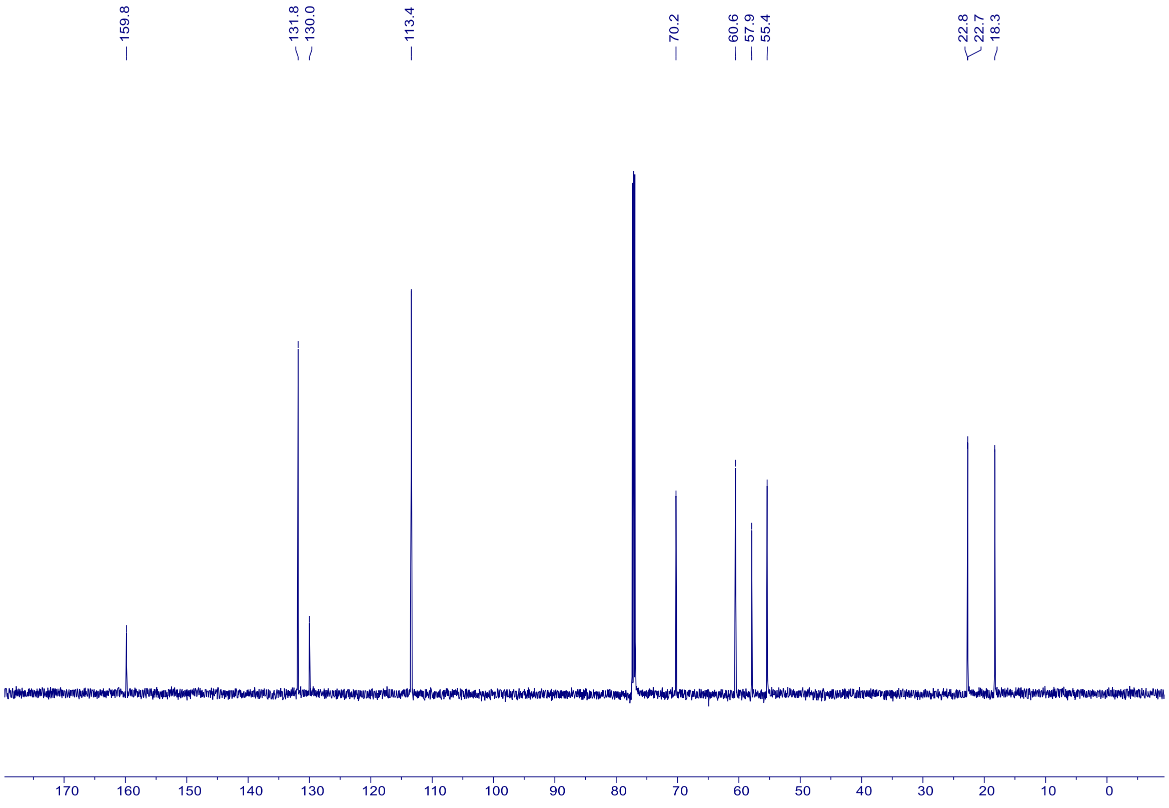

S1 – ^{11}B NMR (192 MHz, $CDCl_3$)

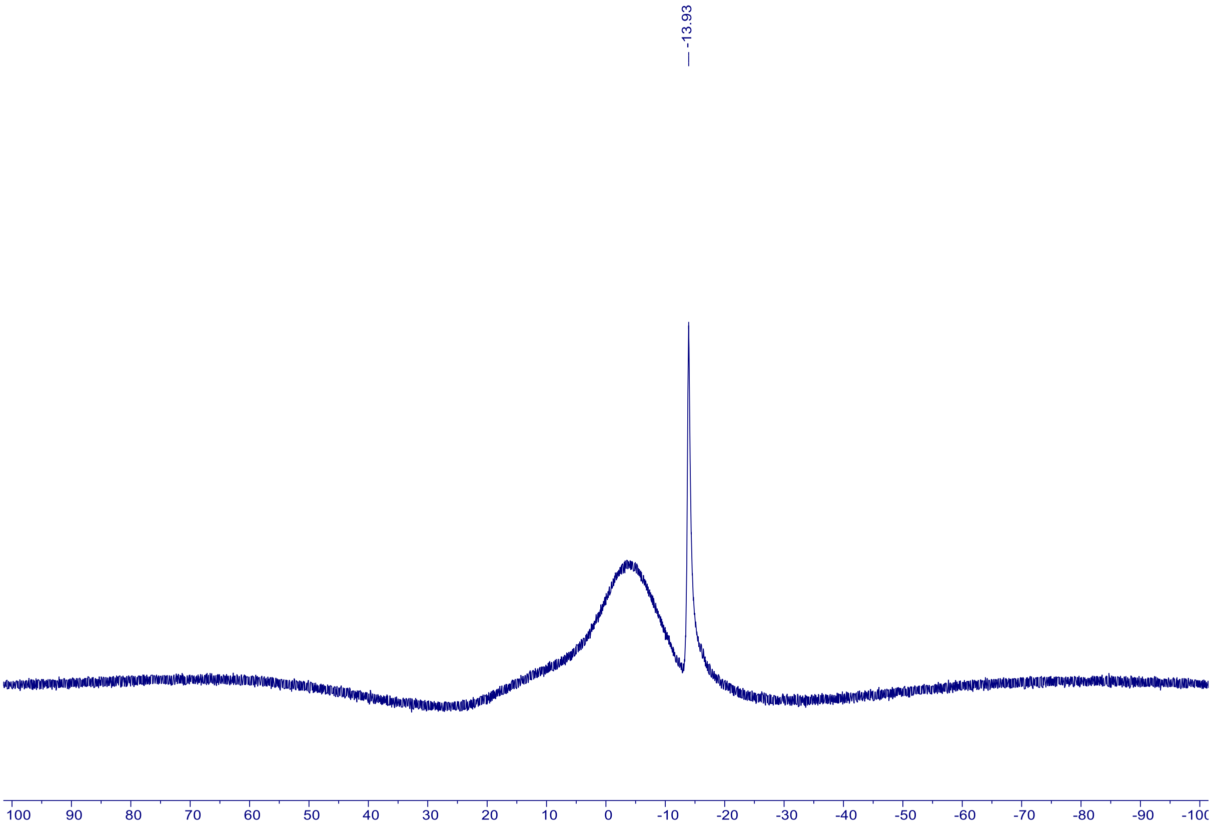

1o – ^{1}H NMR (600 MHz, $CDCl_3$)

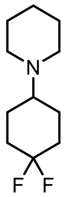

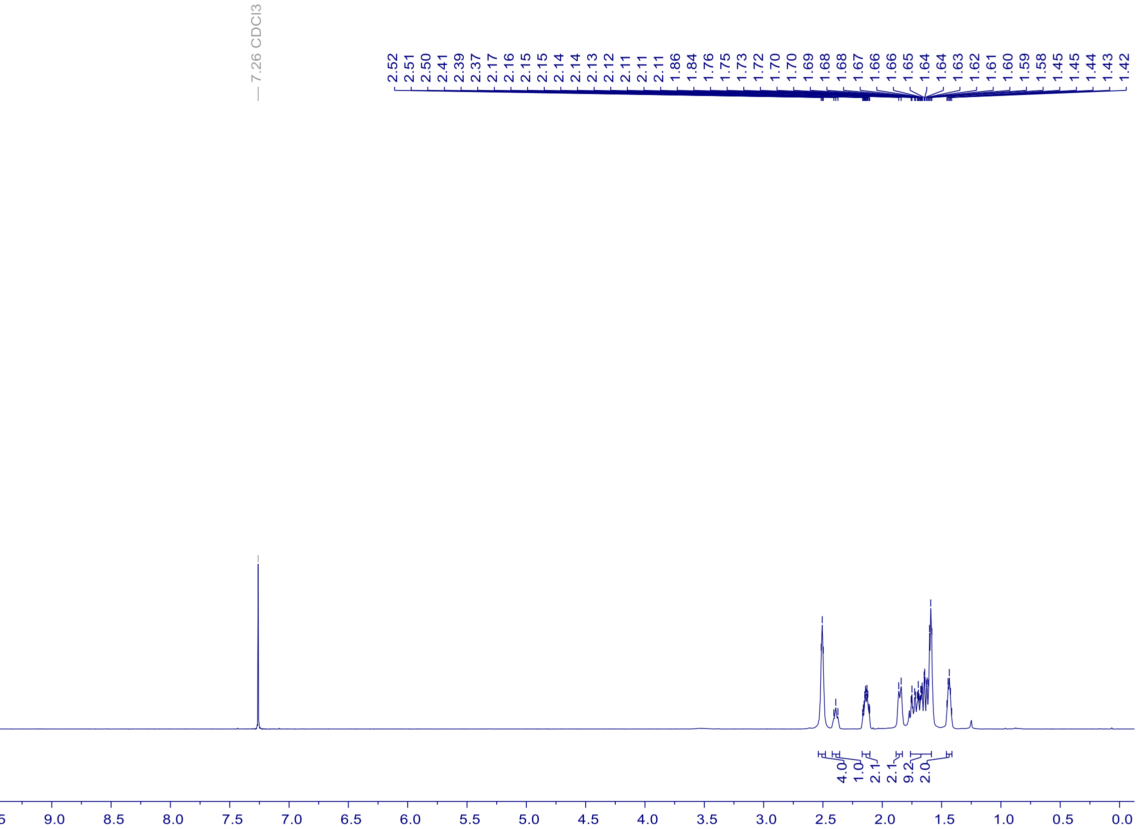

1o – ^{13}C NMR (151 MHz, $CDCl_3$)

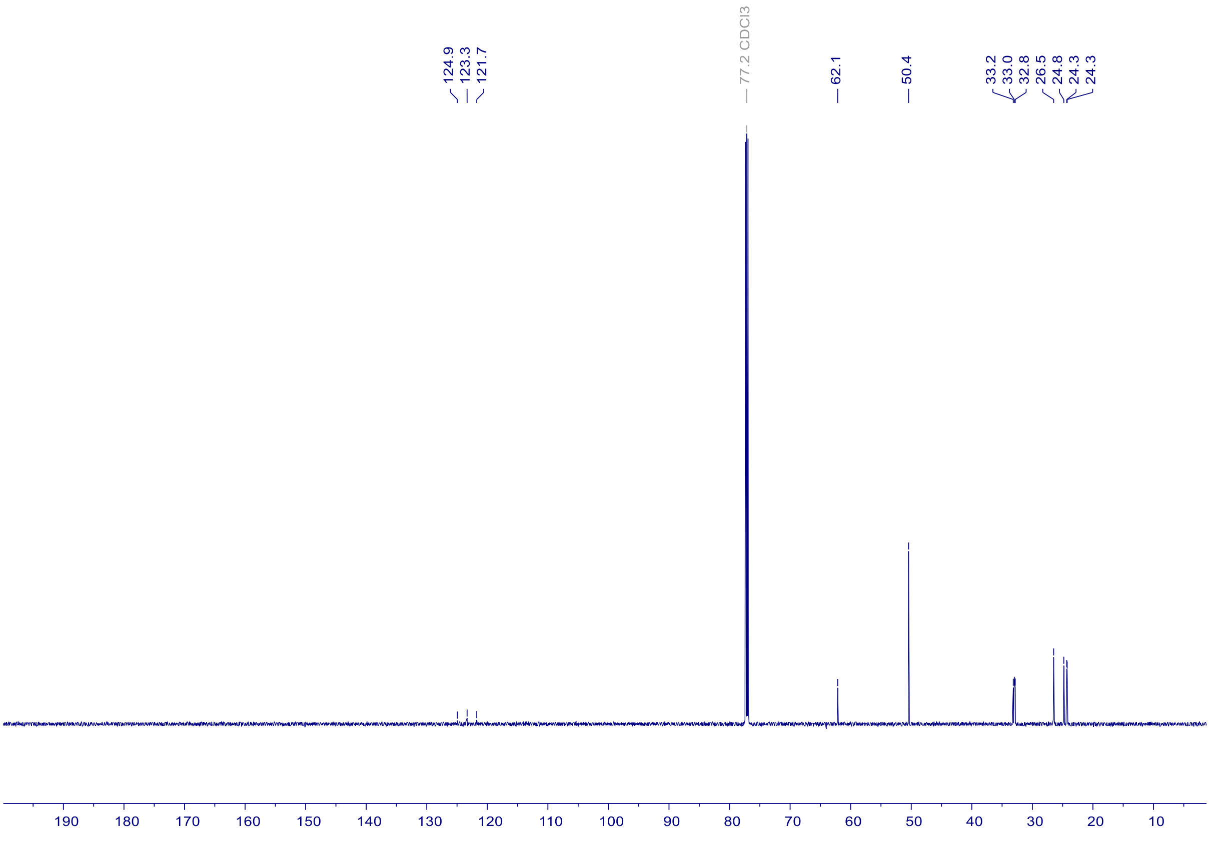

1s – ^{1}H NMR (600 MHz, $CDCl_3$)

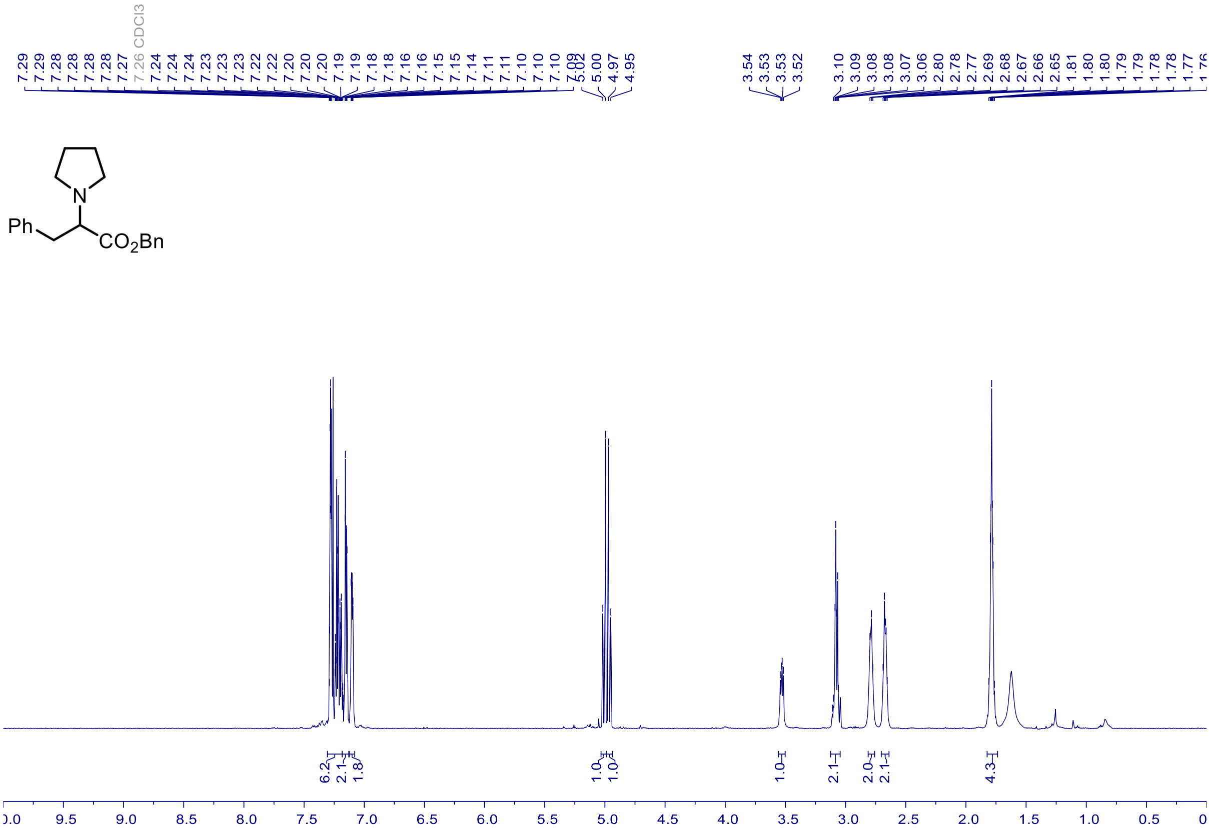

1s – ^{13}C NMR (151 MHz, $CDCl_3$)

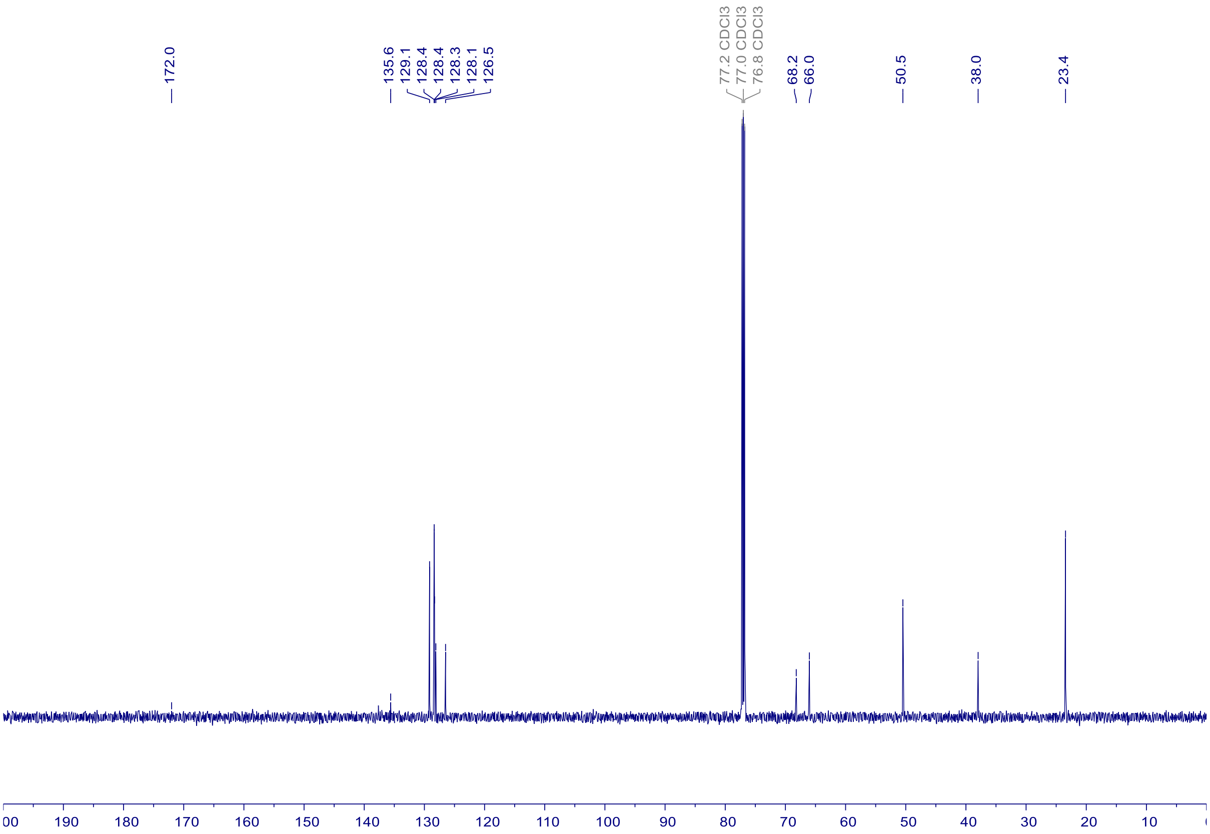

1t – ^{1}H NMR (600 MHz, $CDCl_3$)

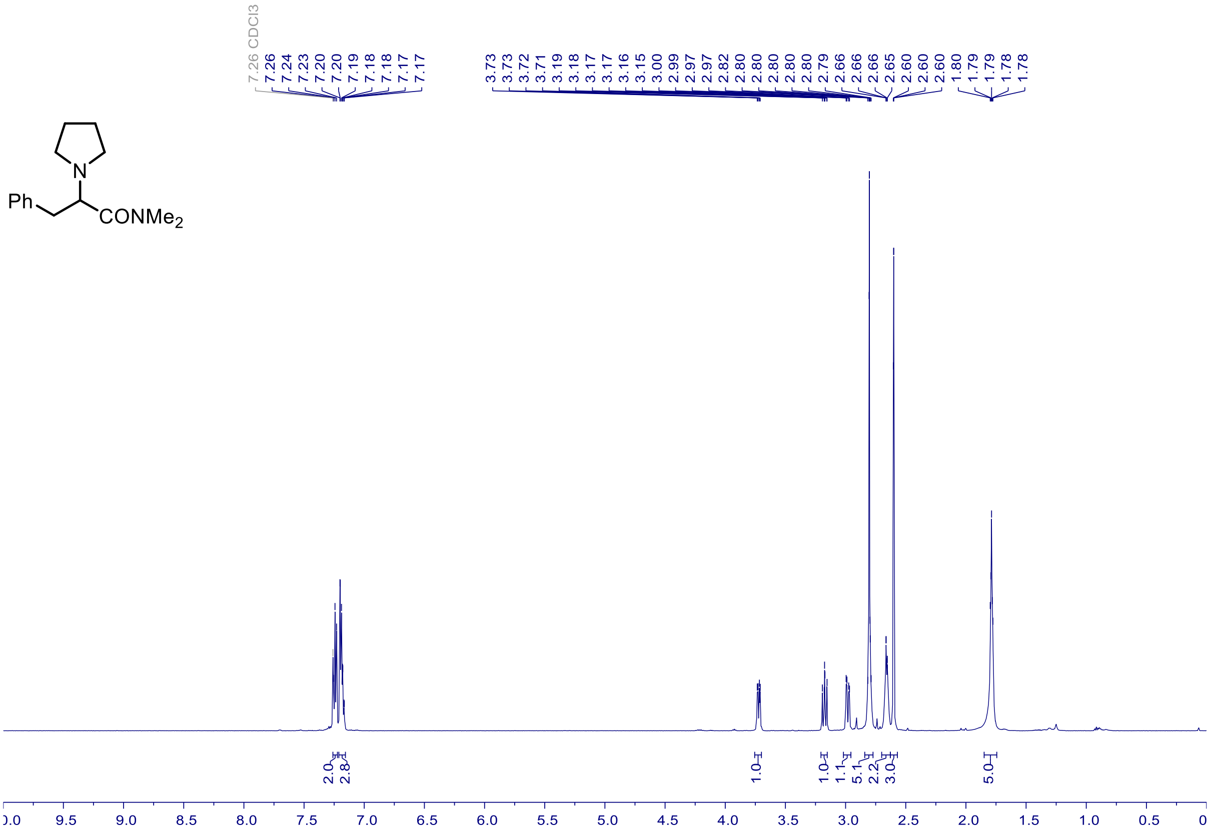

1t – ^{13}C NMR (151 MHz, $CDCl_3$)

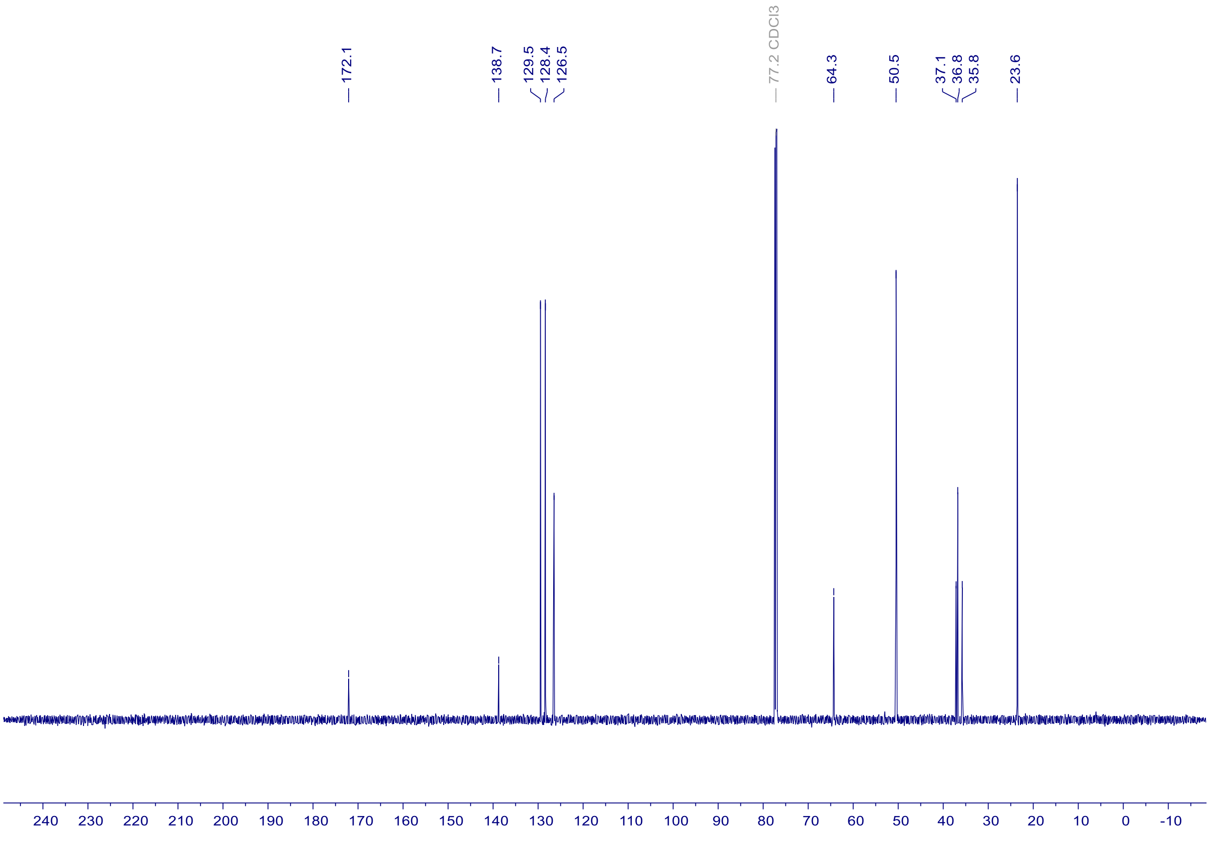

1u – ^{1}H NMR (400 MHz, $CDCl_3$)

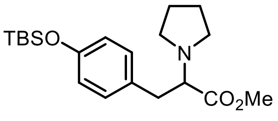

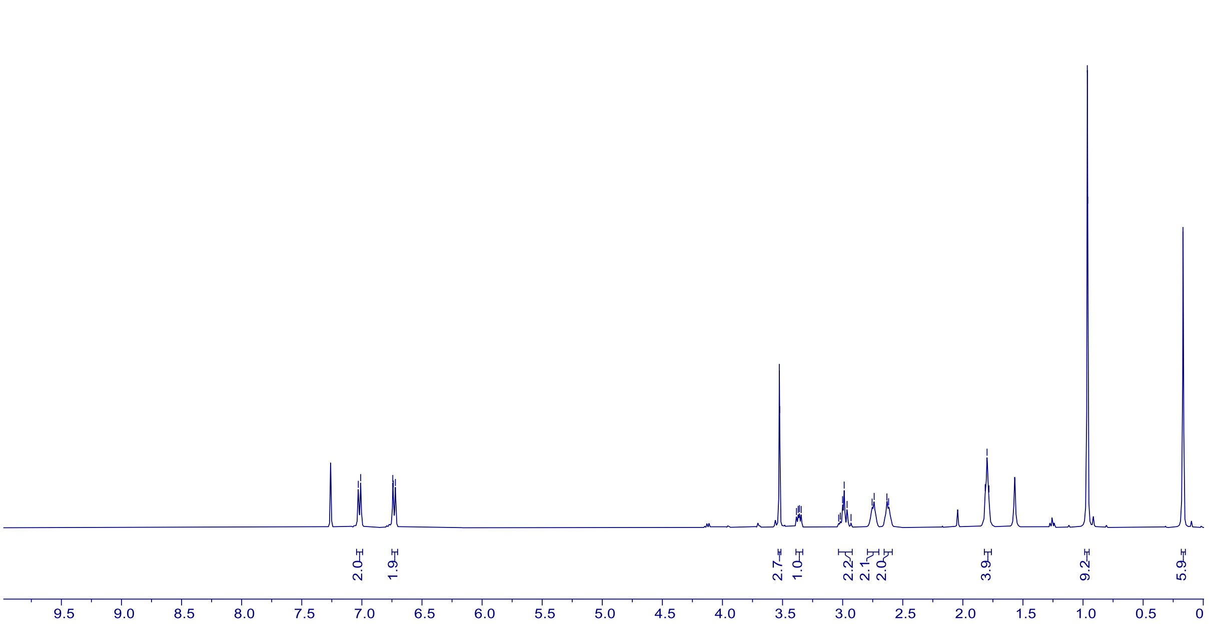

1u – ^{13}C NMR (101 MHz, $CDCl_3$)

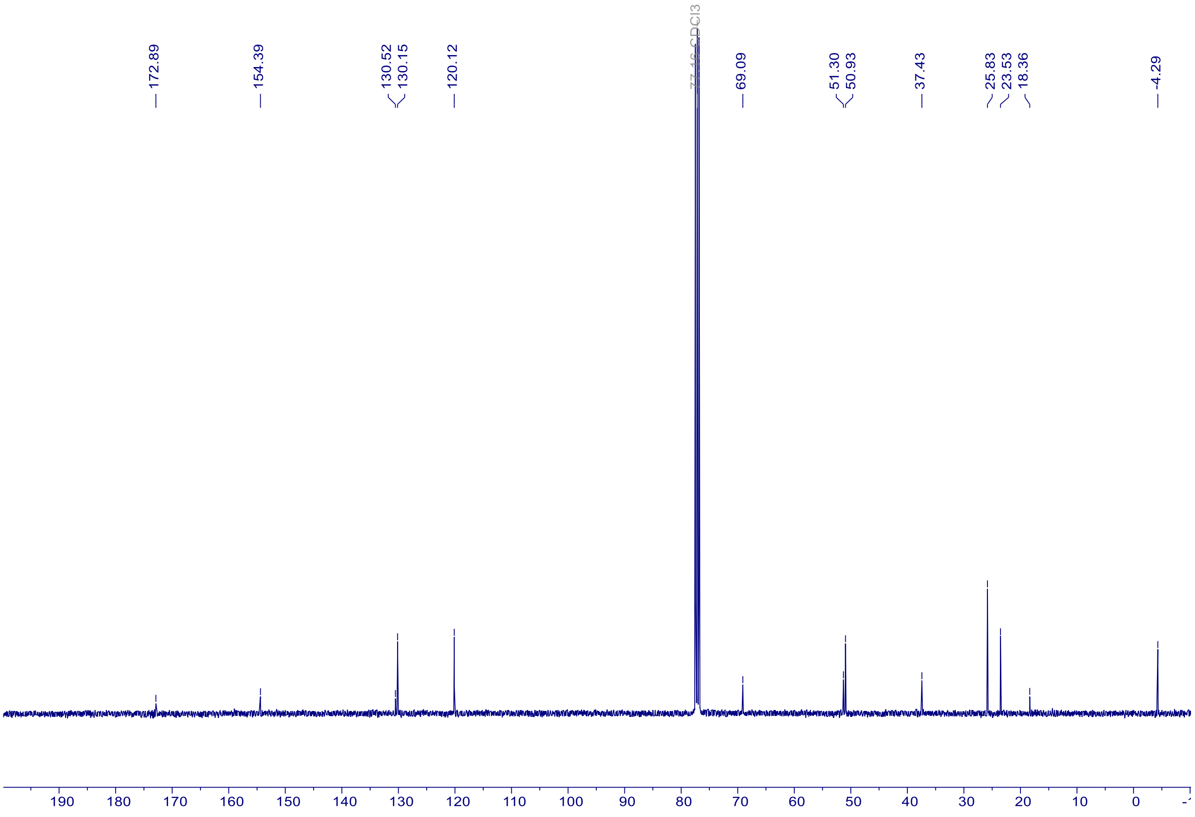

2g – ^{1}H NMR (400 MHz, $CDCl_3$)

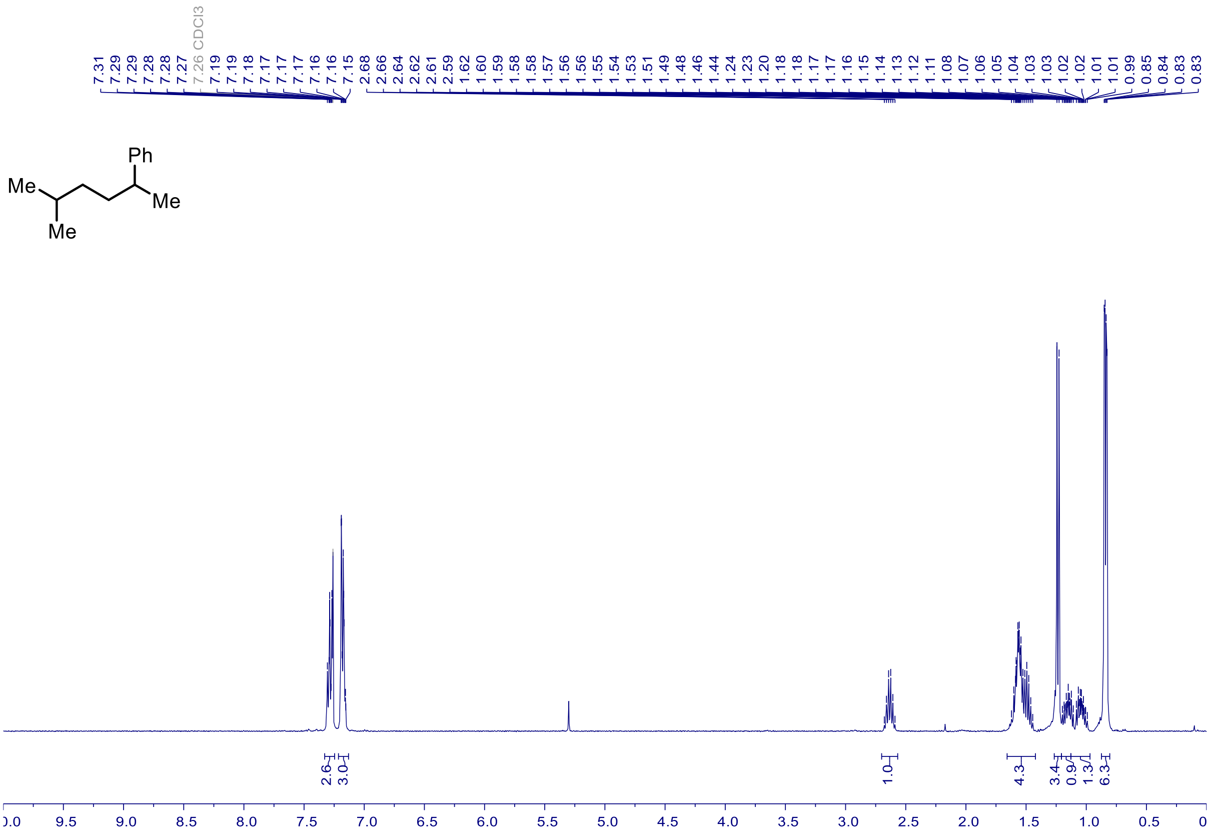

2g – ^{13}C NMR (101 MHz, $CDCl_3$)

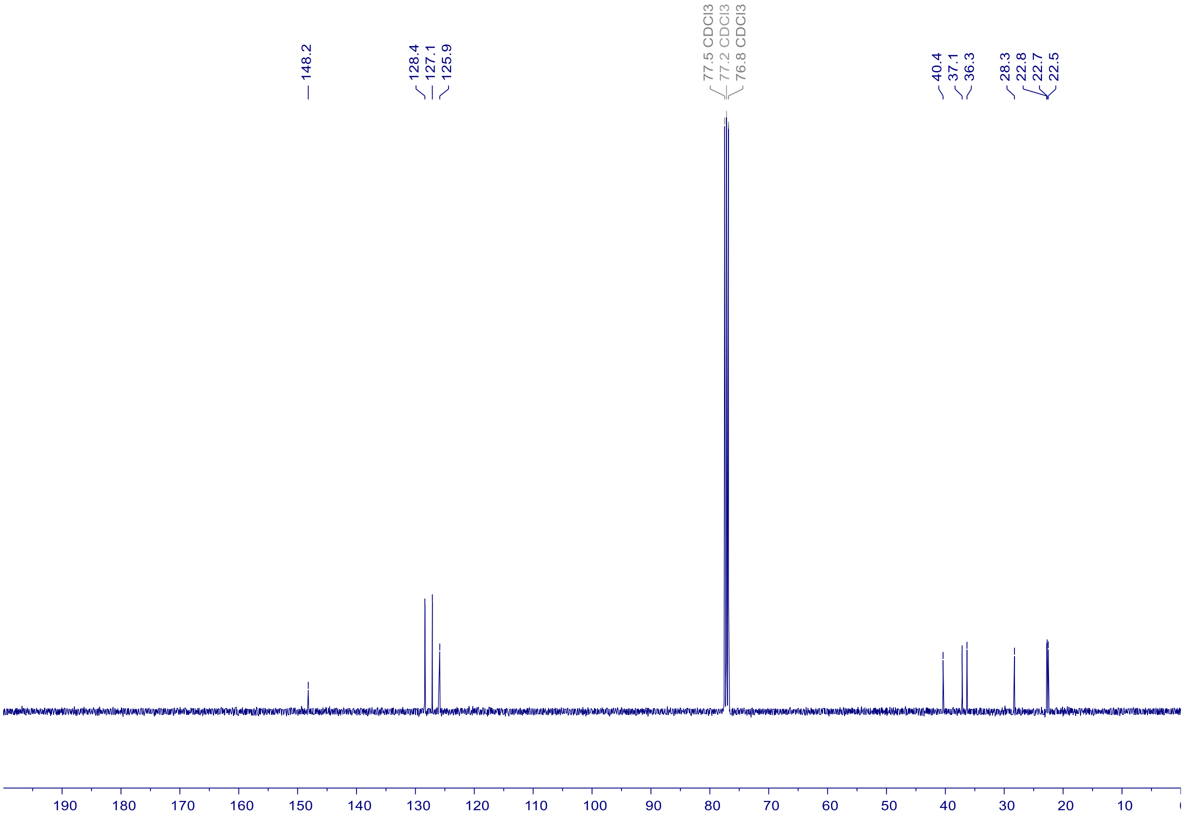

2p – ^{1}H NMR (400 MHz, $CDCl_3$)

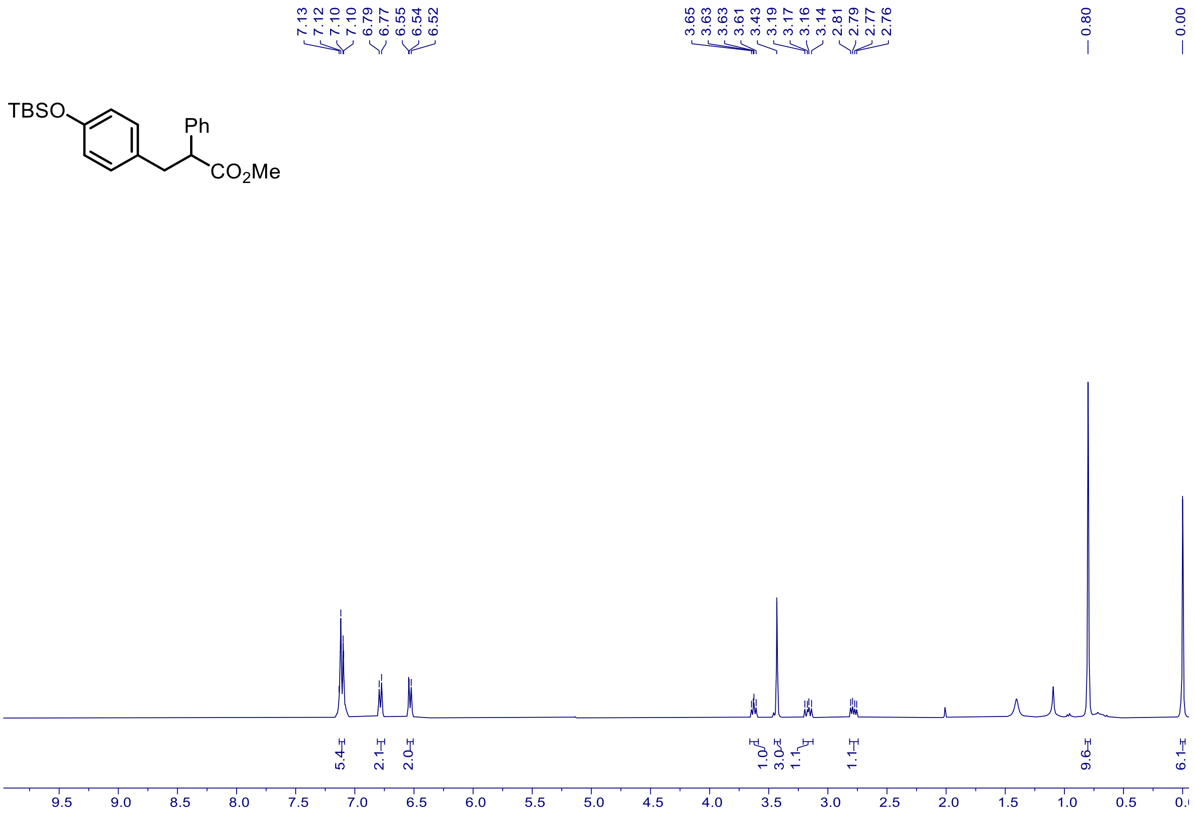

2p – ^{13}C NMR (101 MHz, $CDCl_3$)

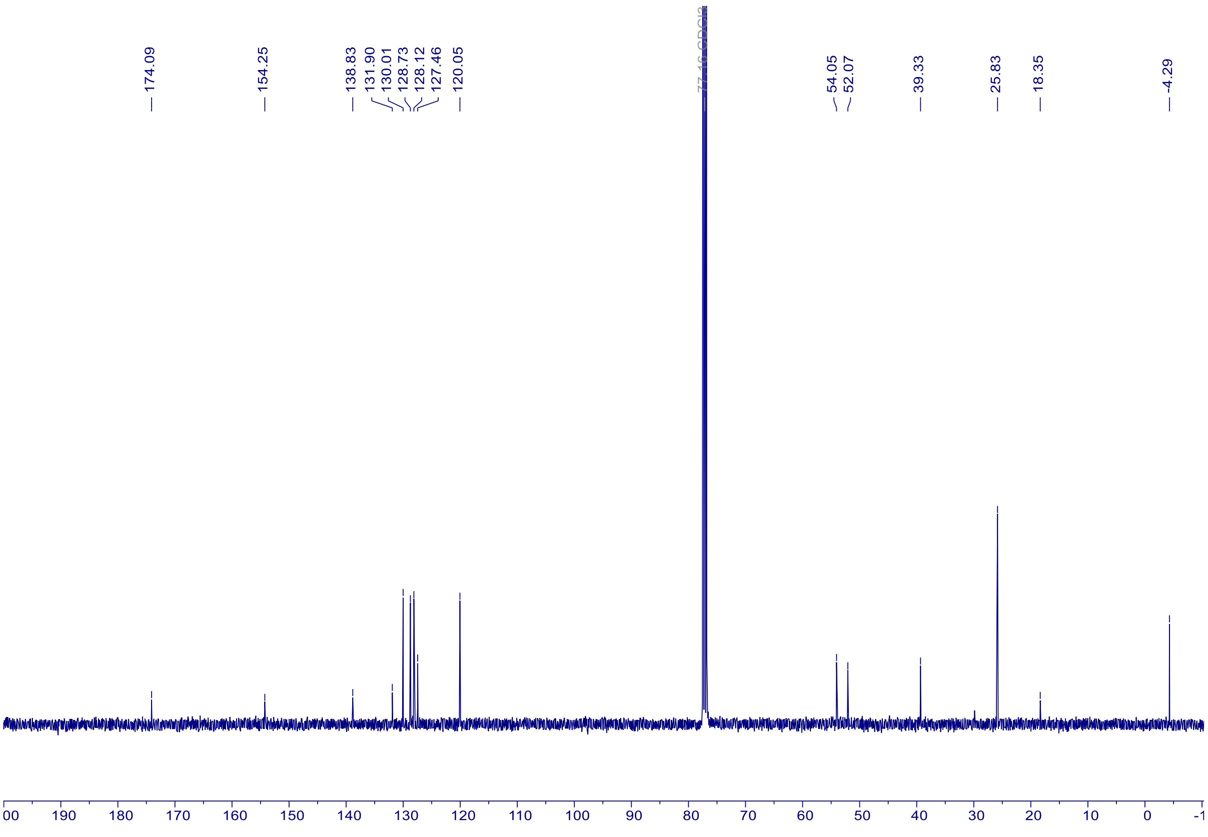

2m – ^{1}H NMR (400 MHz, $CDCl_3$)

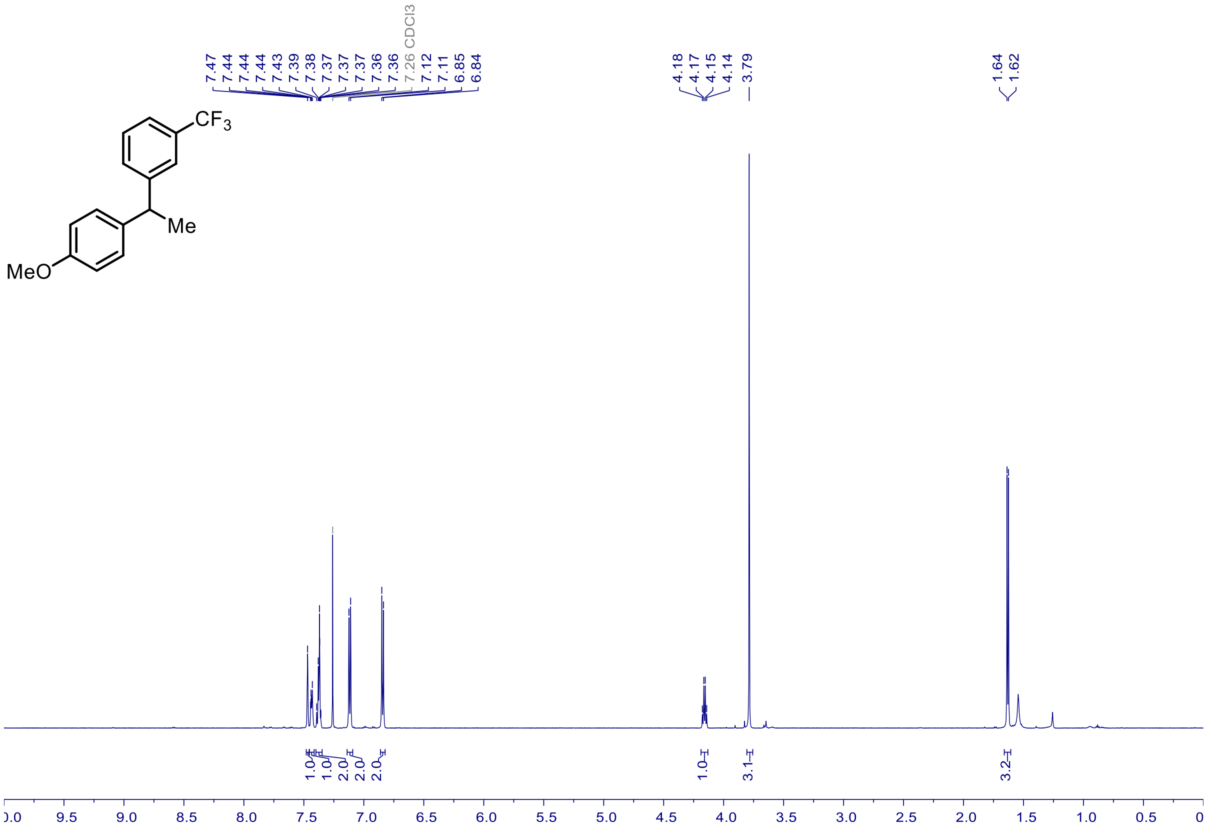

2m – ^{13}C NMR (101 MHz, $CDCl_3$)

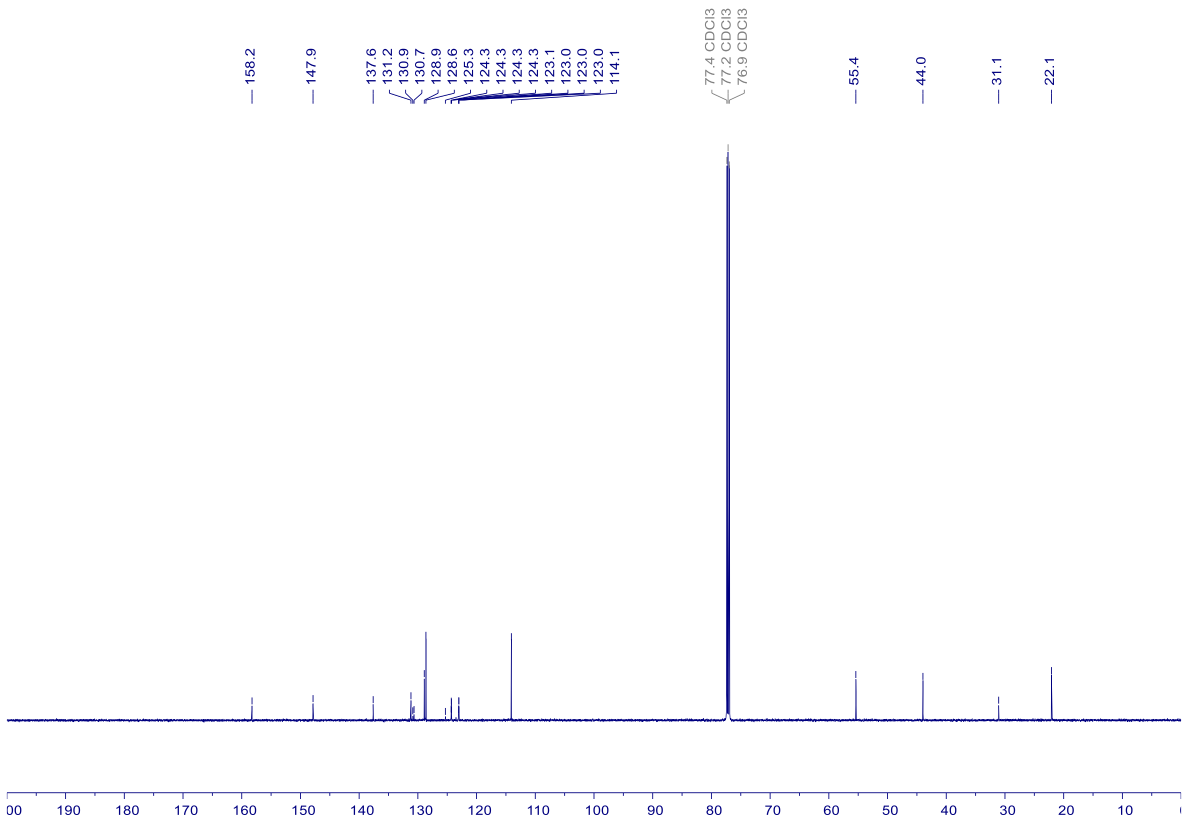

2m – ^{19}F NMR (565 MHz, $CDCl_3$)

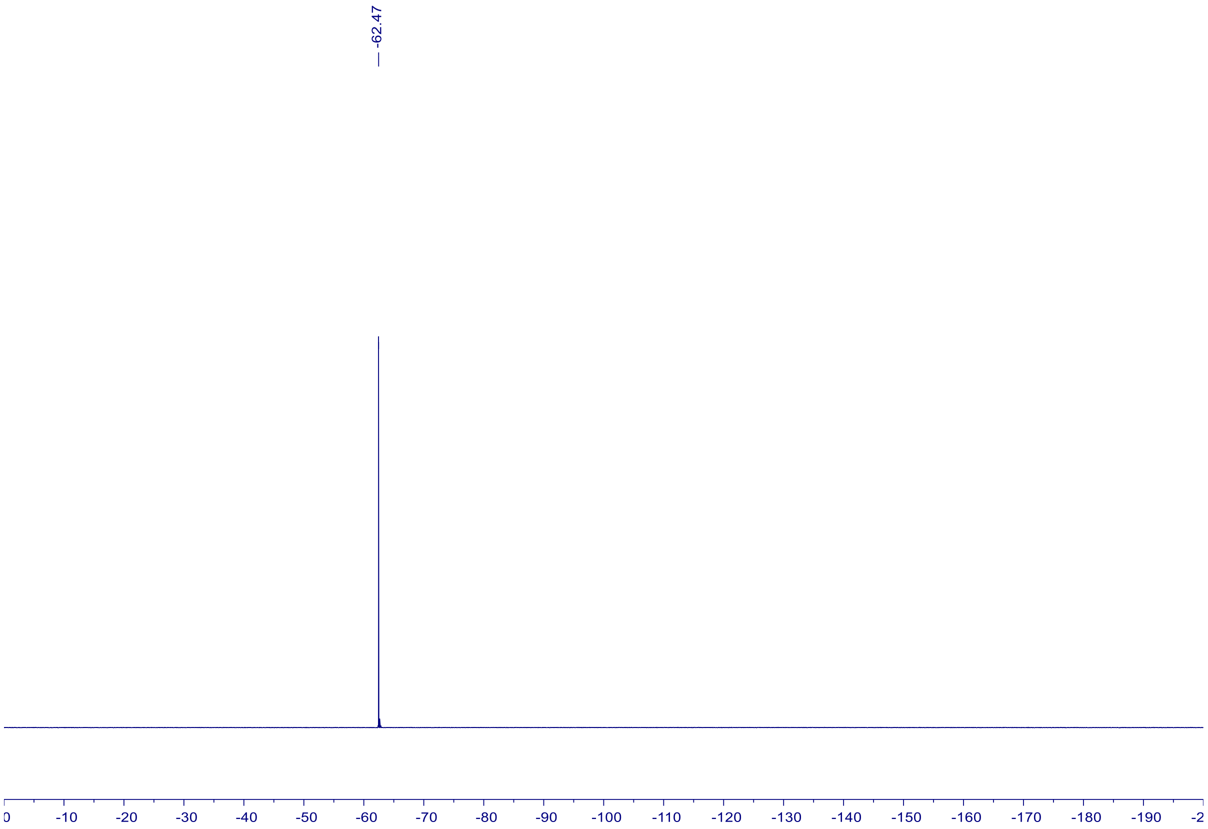

2aa – ^{1}H NMR (600 MHz, CDCl$_3$)

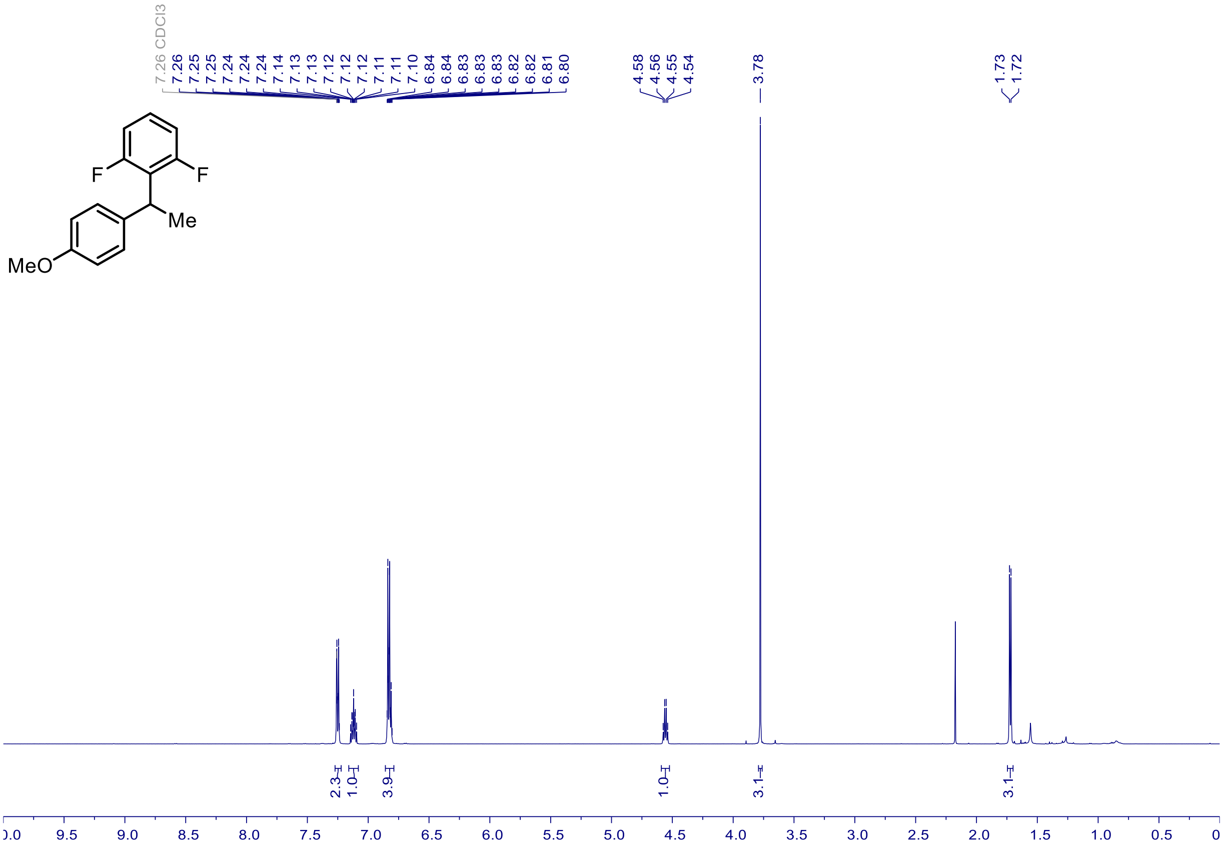

2aa – ^{13}C NMR (101 MHz, CDCl$_3$)

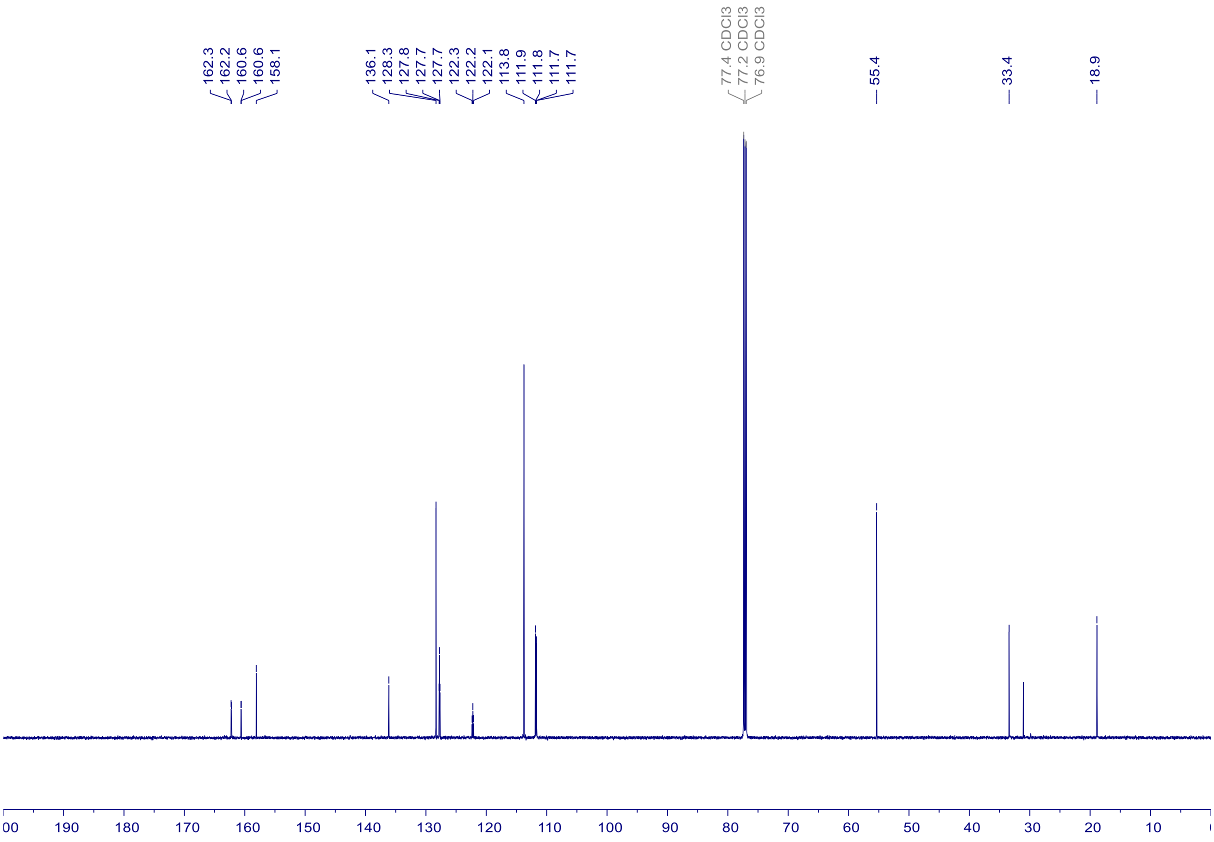

2aa – ^{19}F NMR (565 MHz, $CDCl_3$)

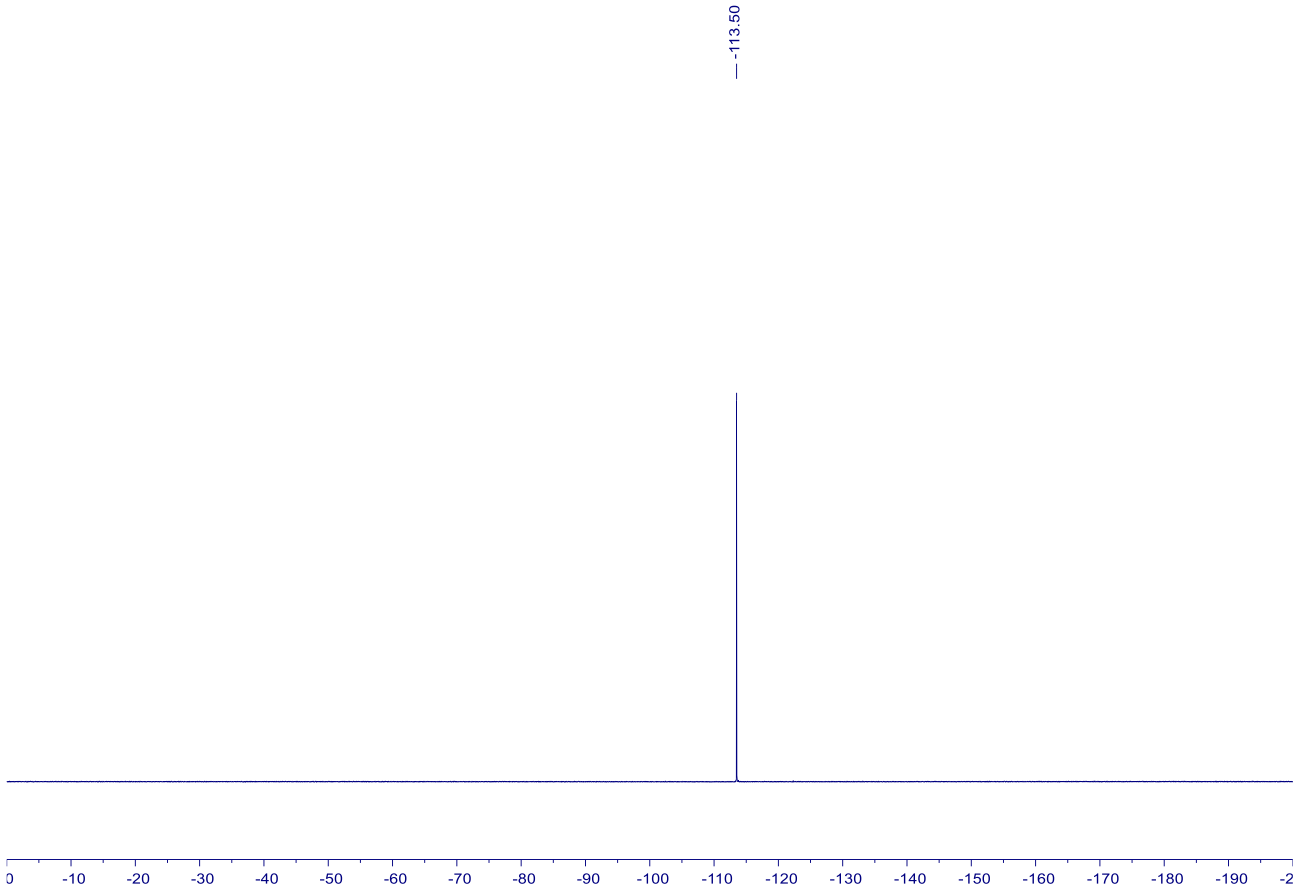

2ab – ^{1}H NMR (600 MHz, $CDCl_3$)

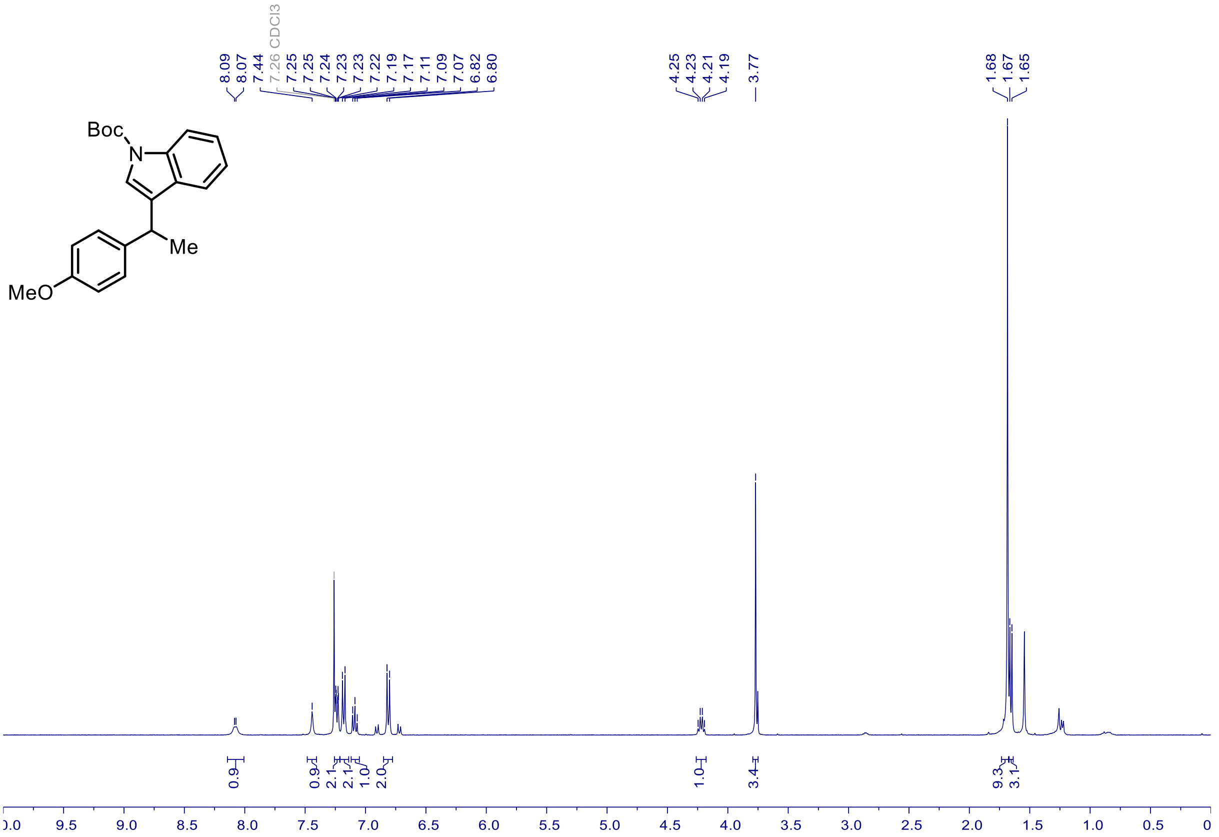

2ab – ^{13}C NMR (101 MHz, $CDCl_3$)

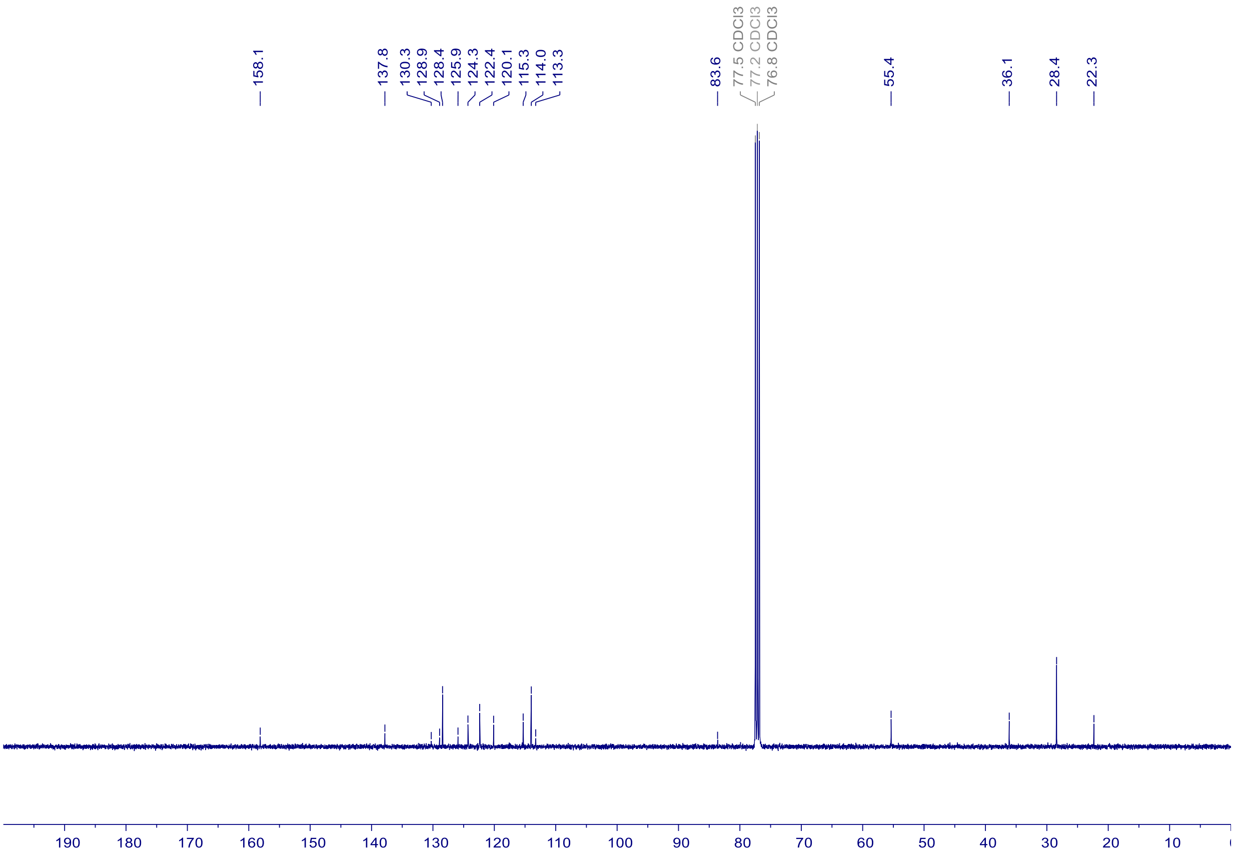

2ac – ^{1}H NMR (600 MHz, CDCl$_3$)

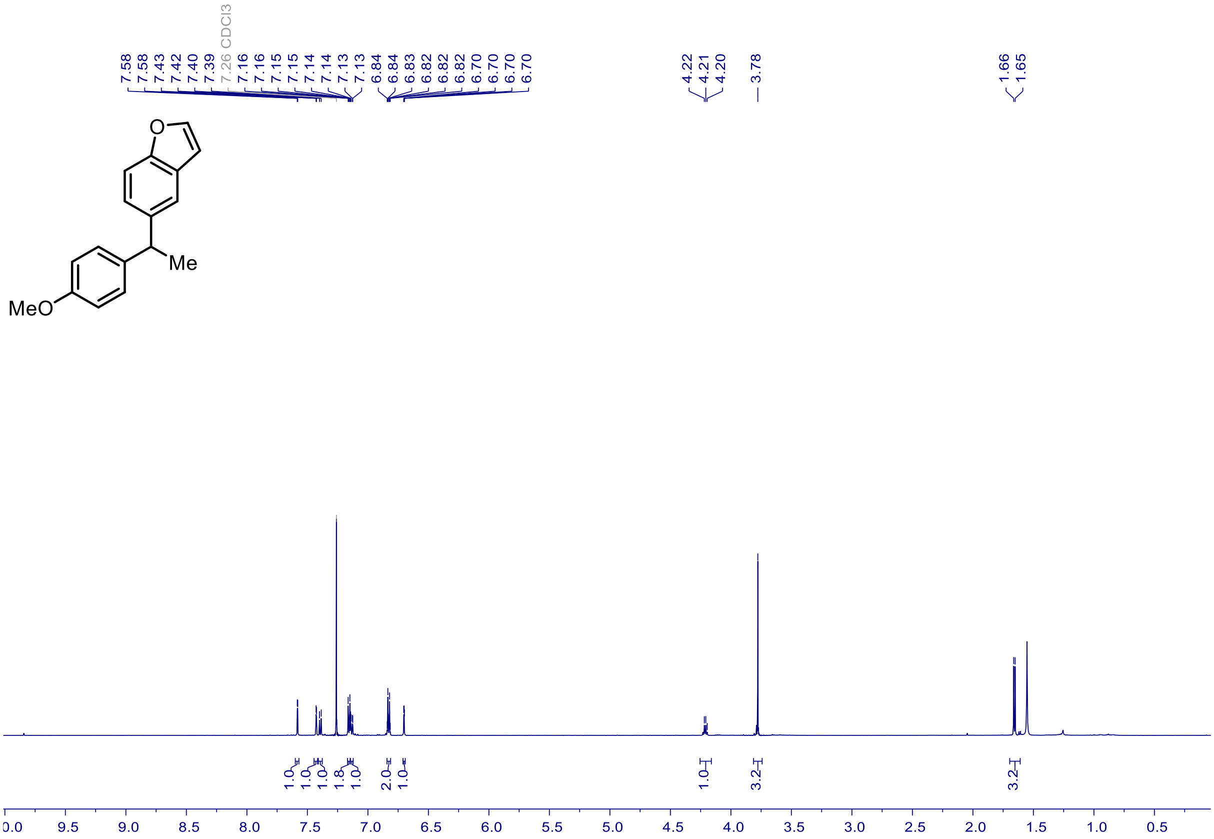

2ac – ^{13}C NMR (151 MHz, CDCl$_3$)

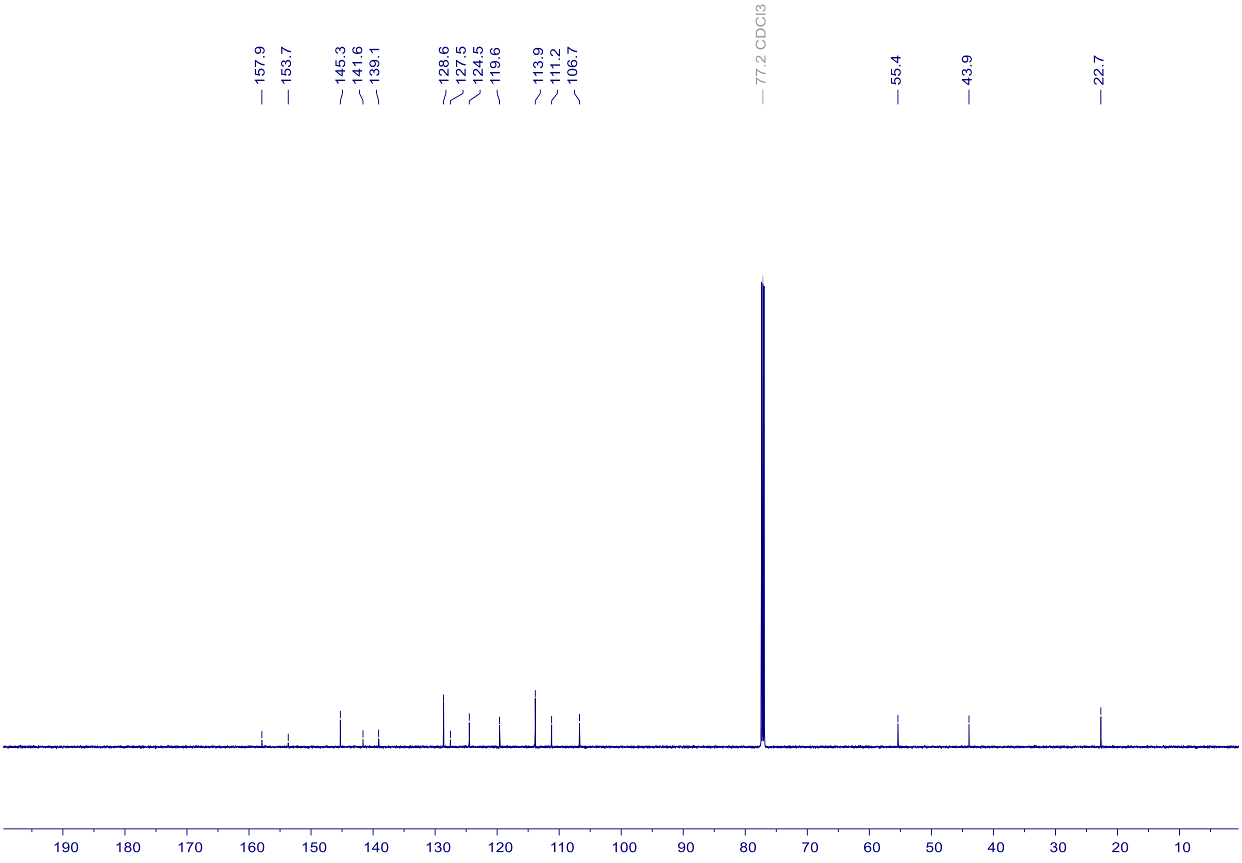

2ad – ^{1}H NMR (600 MHz, CDCl$_3$)

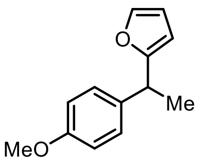

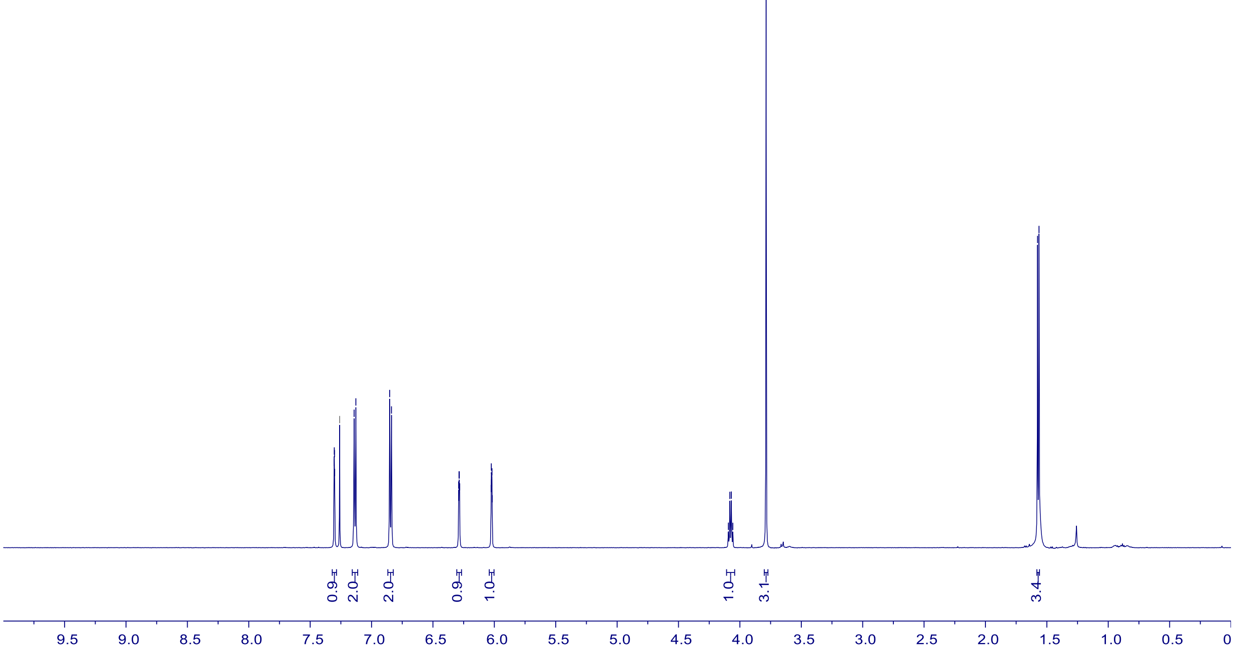

2ad – ^{13}C NMR (151 MHz, CDCl$_3$)

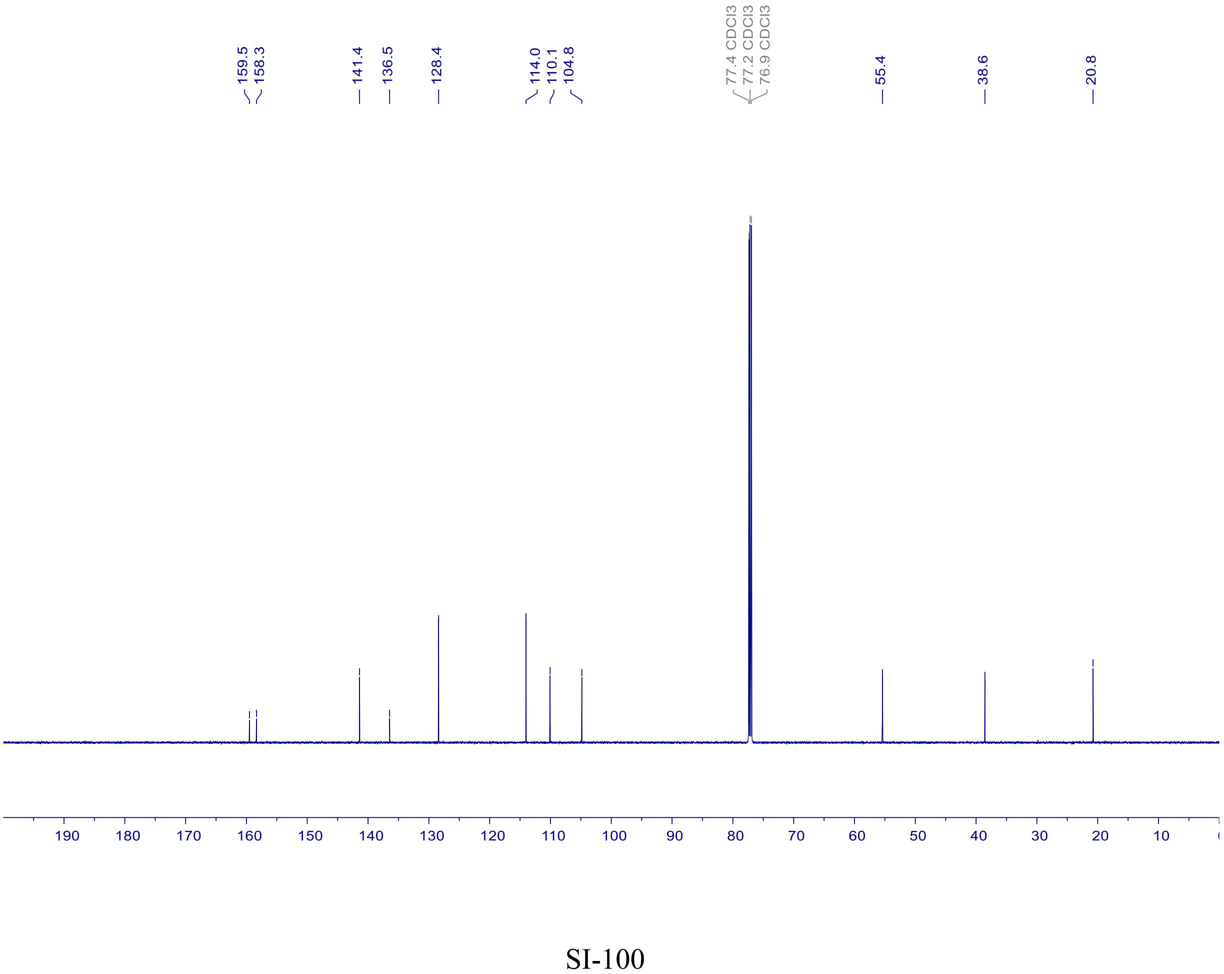

2ae – ^{1}H NMR (600 MHz, $CDCl_3$)

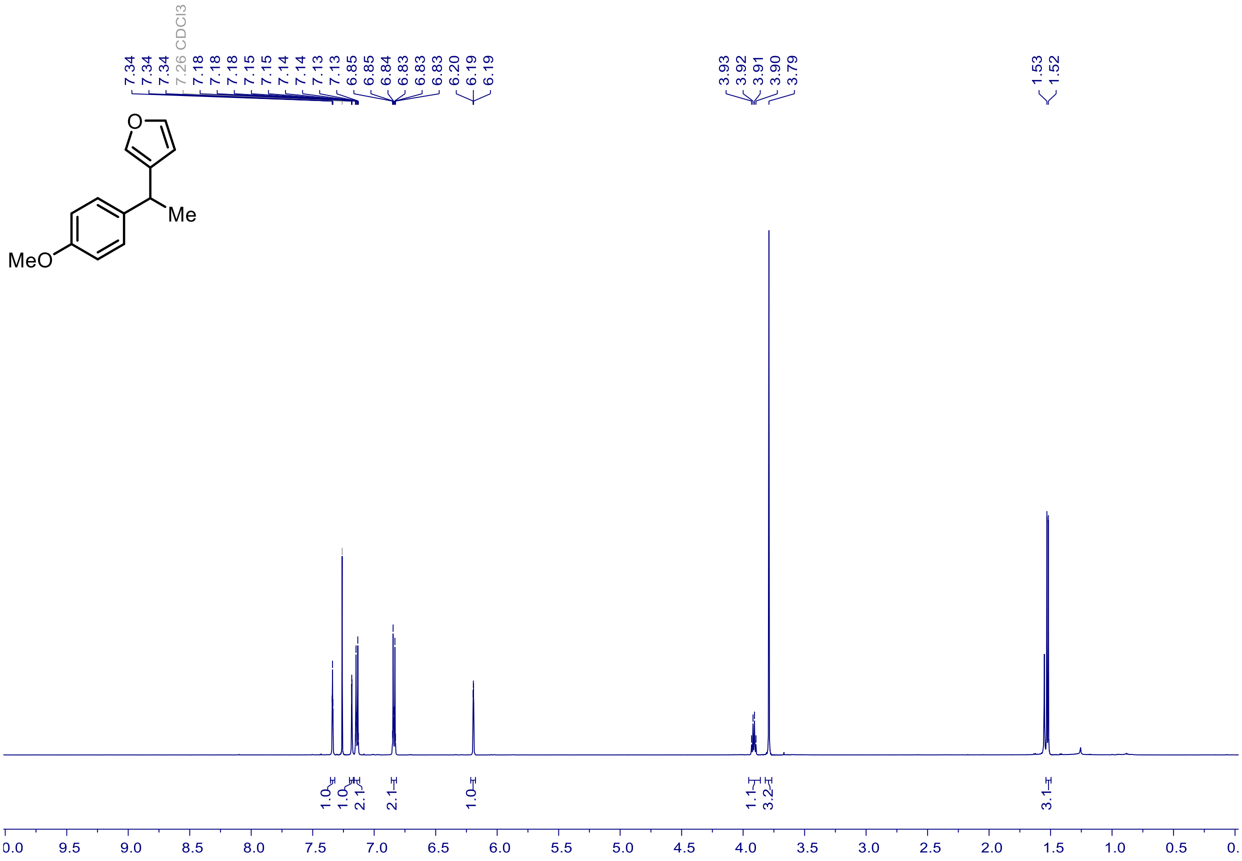

2ae – ^{13}C NMR (151 MHz, $CDCl_3$)

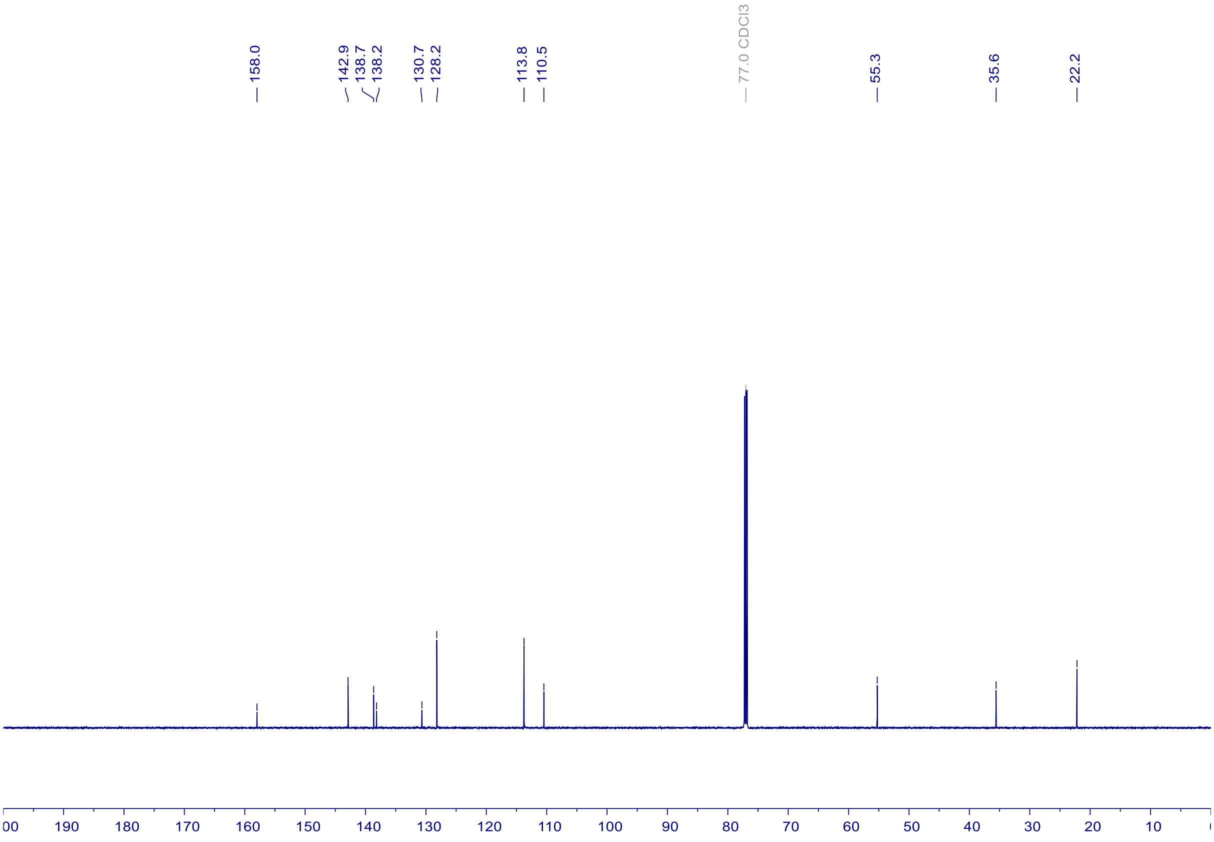

2ag – ^{1}H NMR (400 MHz, $CDCl_3$)

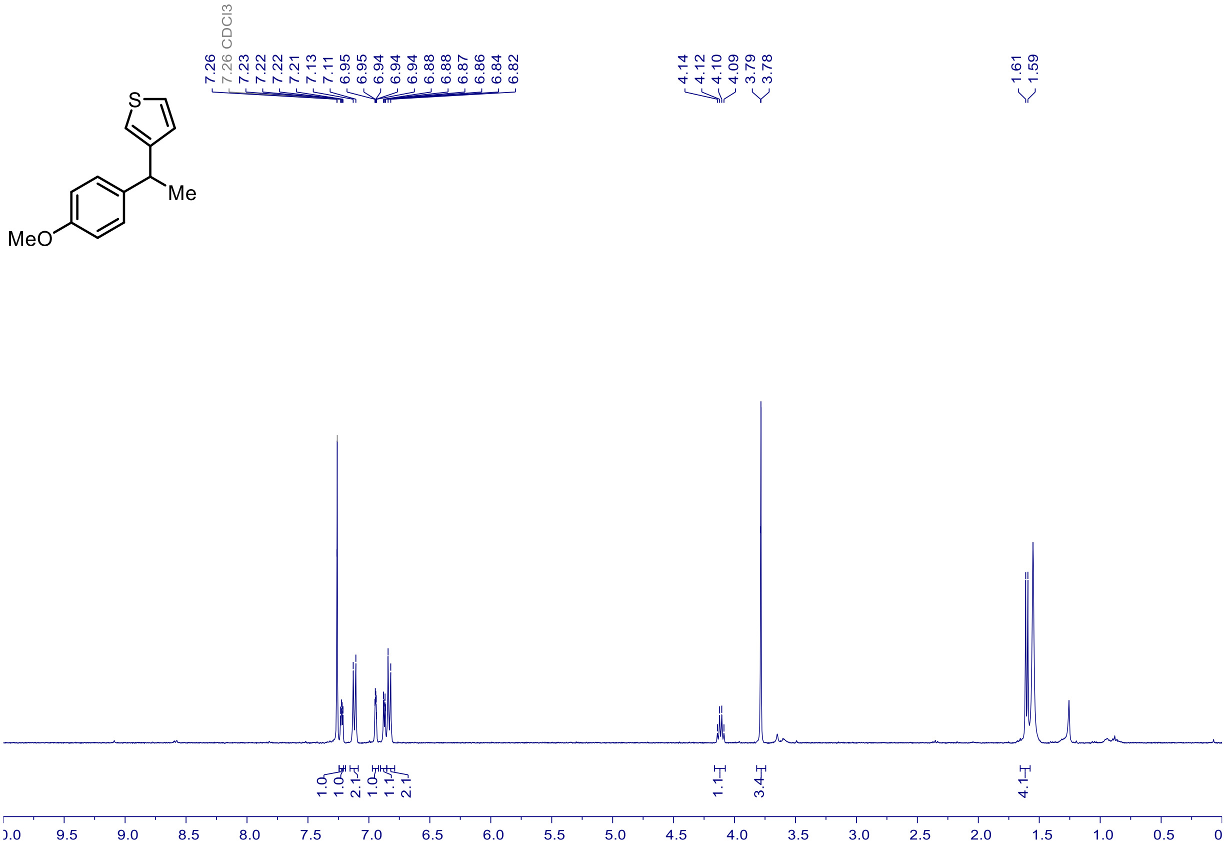

2ag – ^{13}C NMR (101 MHz, $CDCl_3$)

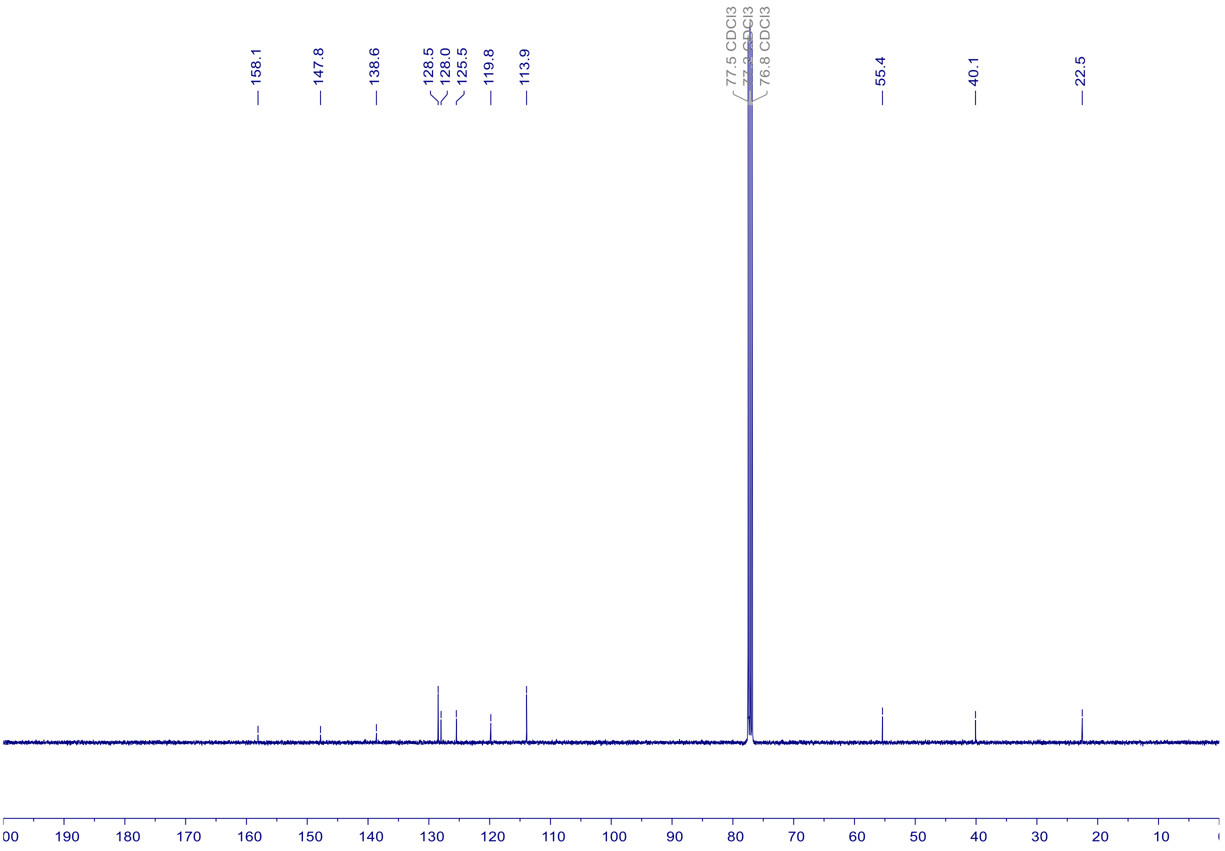

2ah – ^{1}H NMR (400 MHz, $CDCl_3$)

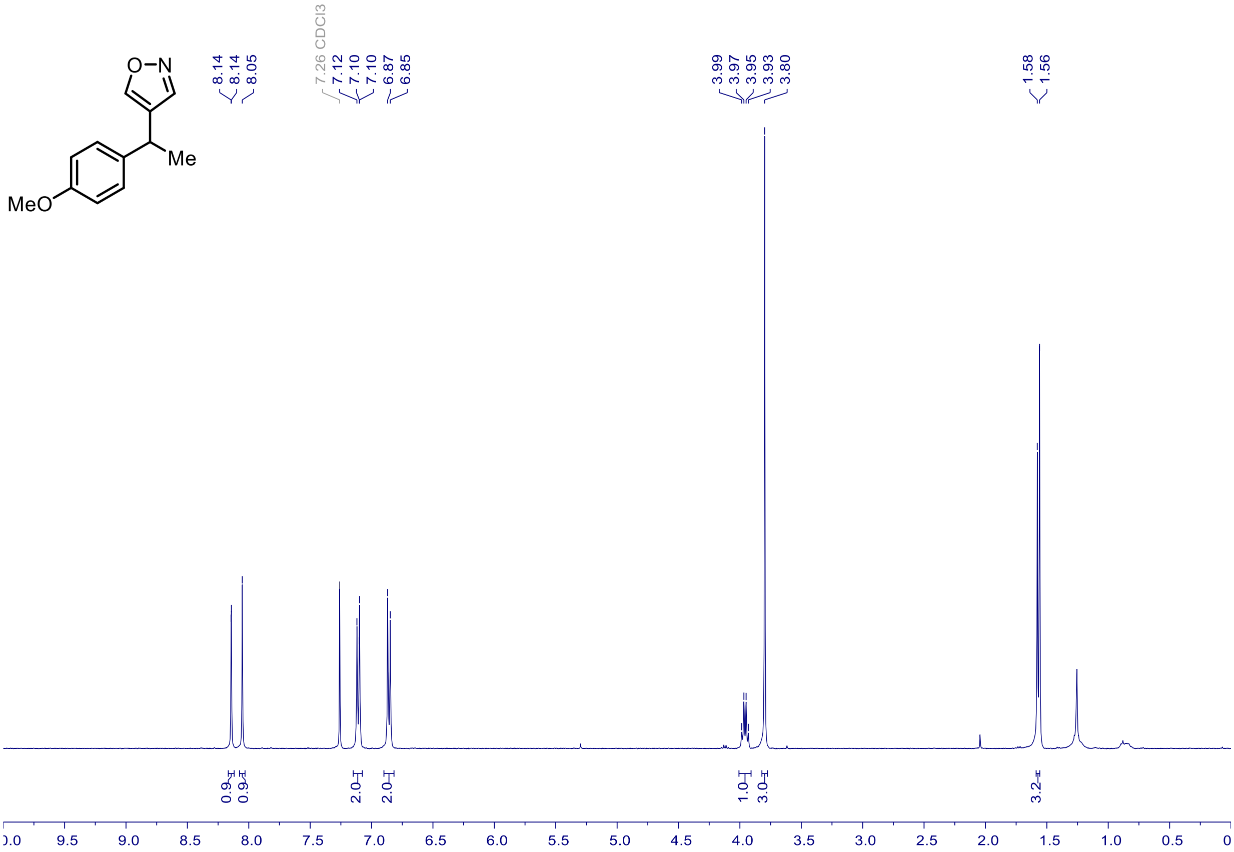

2ah – ^{13}C NMR (101 MHz, $CDCl_3$)

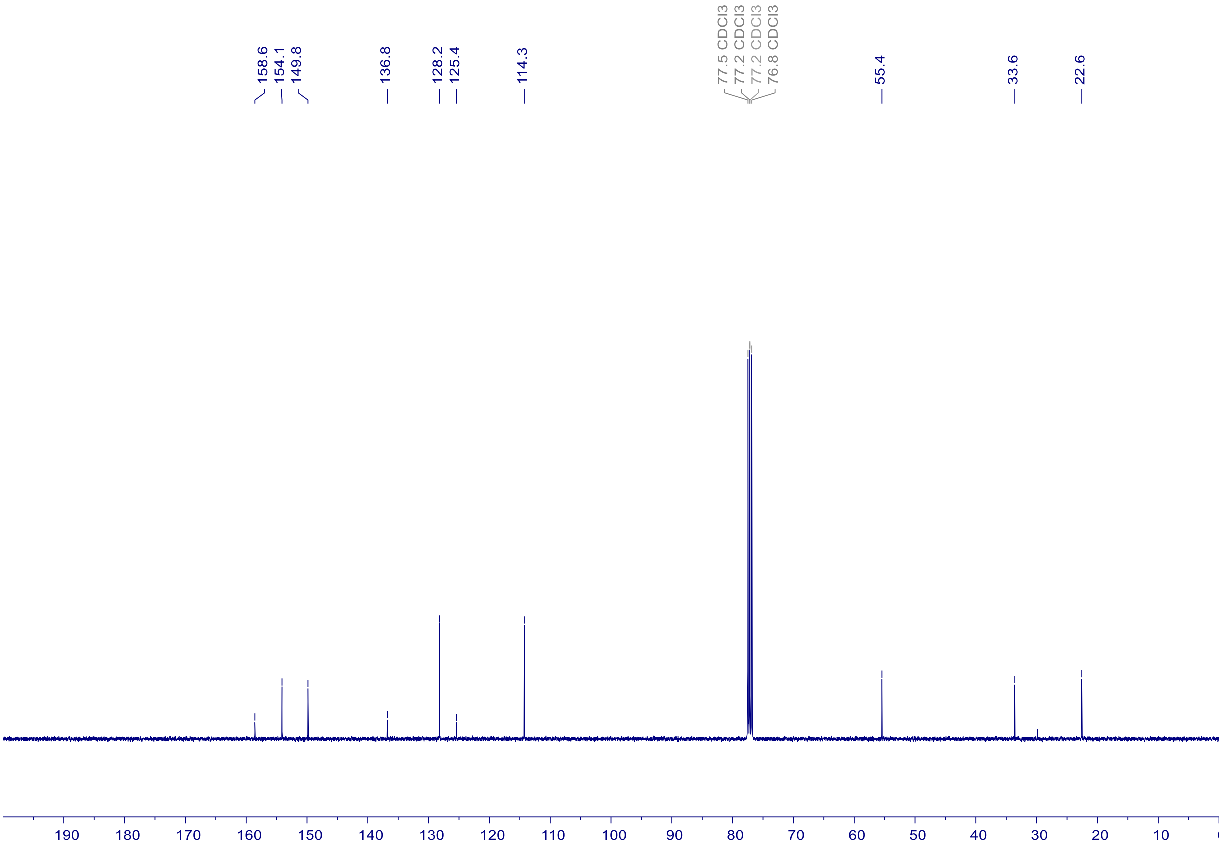

2ai – ^{1}H NMR (400 MHz, $CDCl_3$)

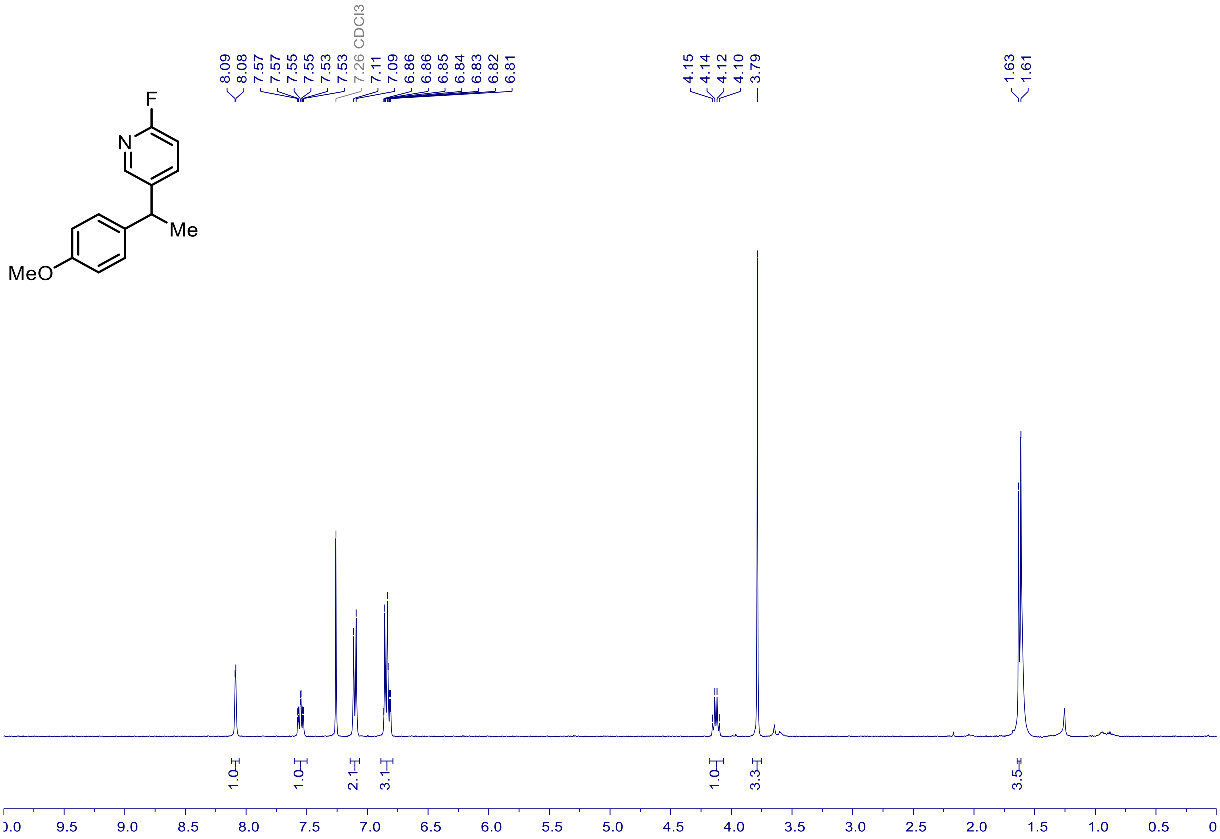

2ai – ^{13}C NMR (101 MHz, $CDCl_3$)

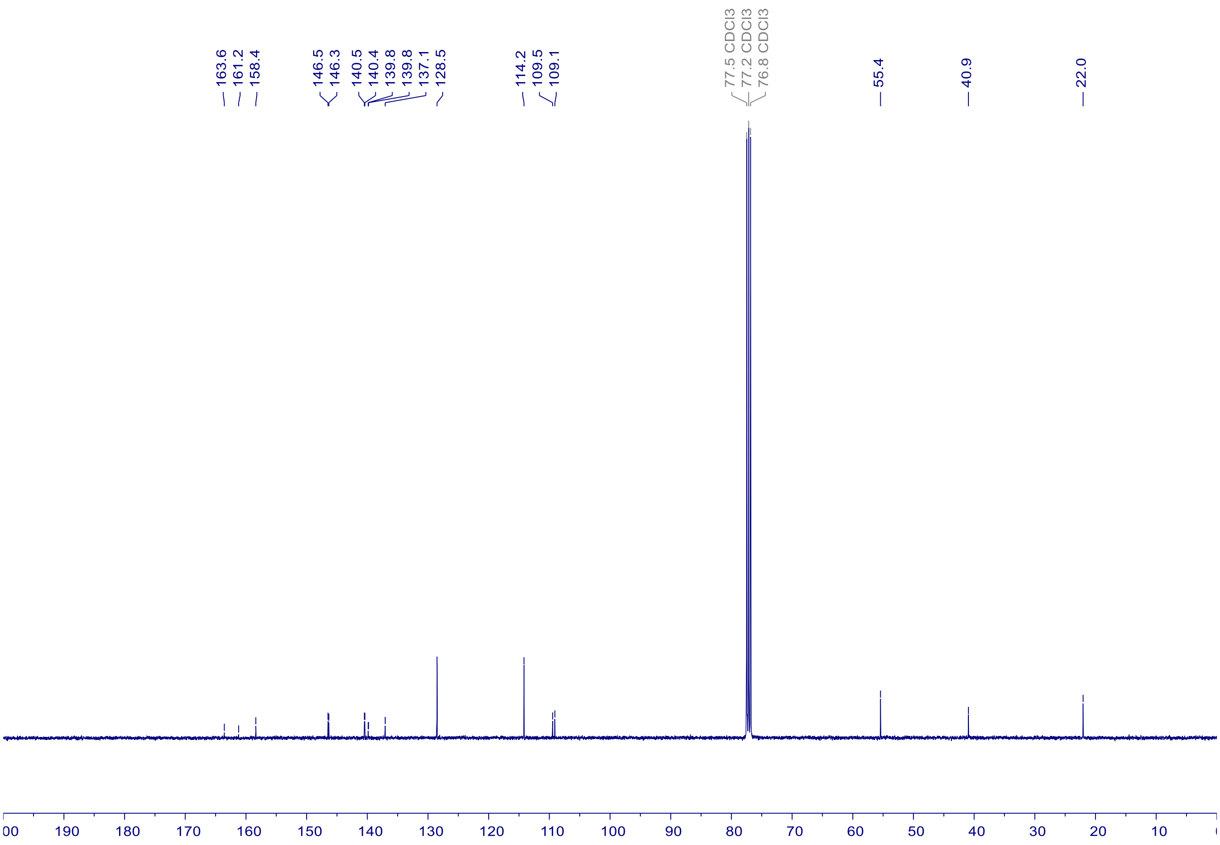

2ai – ^{19}F NMR (565 MHz, $CDCl_3$)

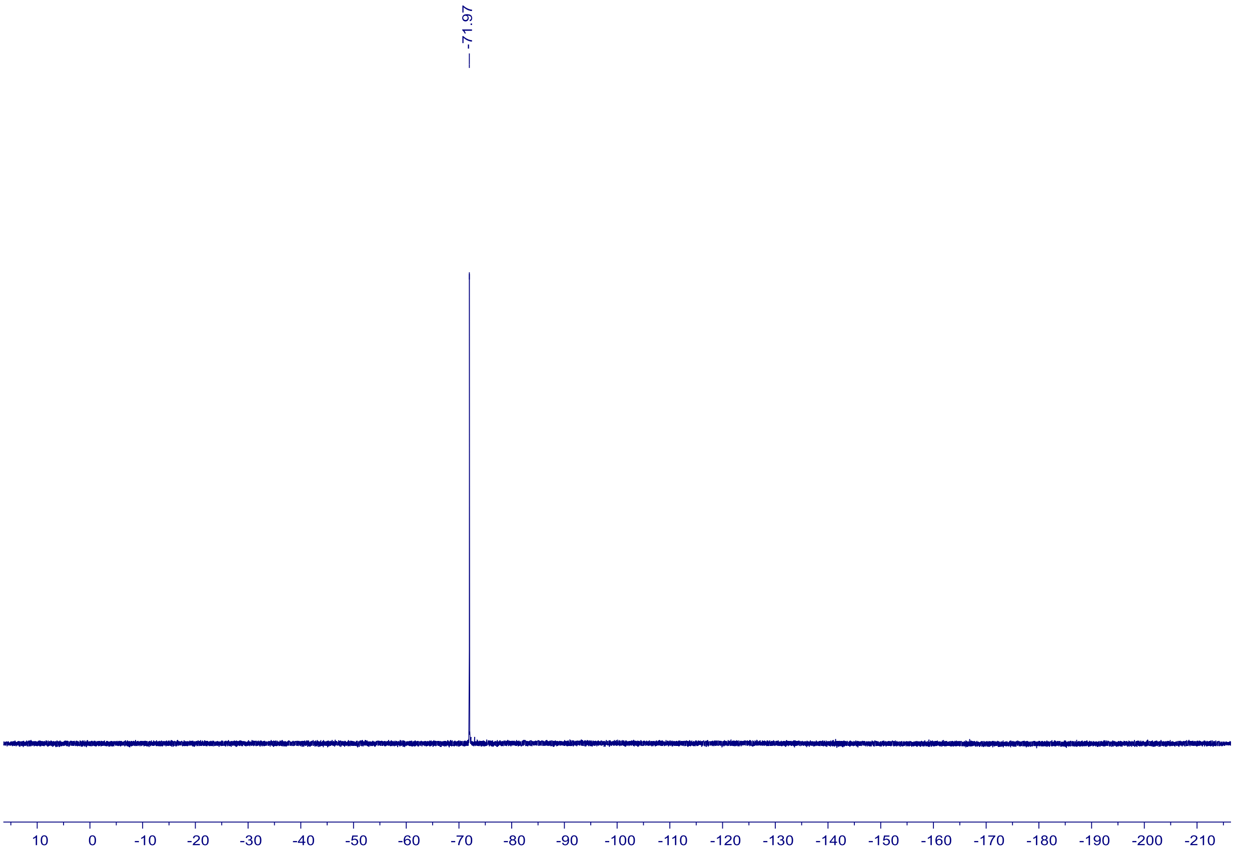

2aj – ^{1}H NMR (600 MHz, $CDCl_3$)

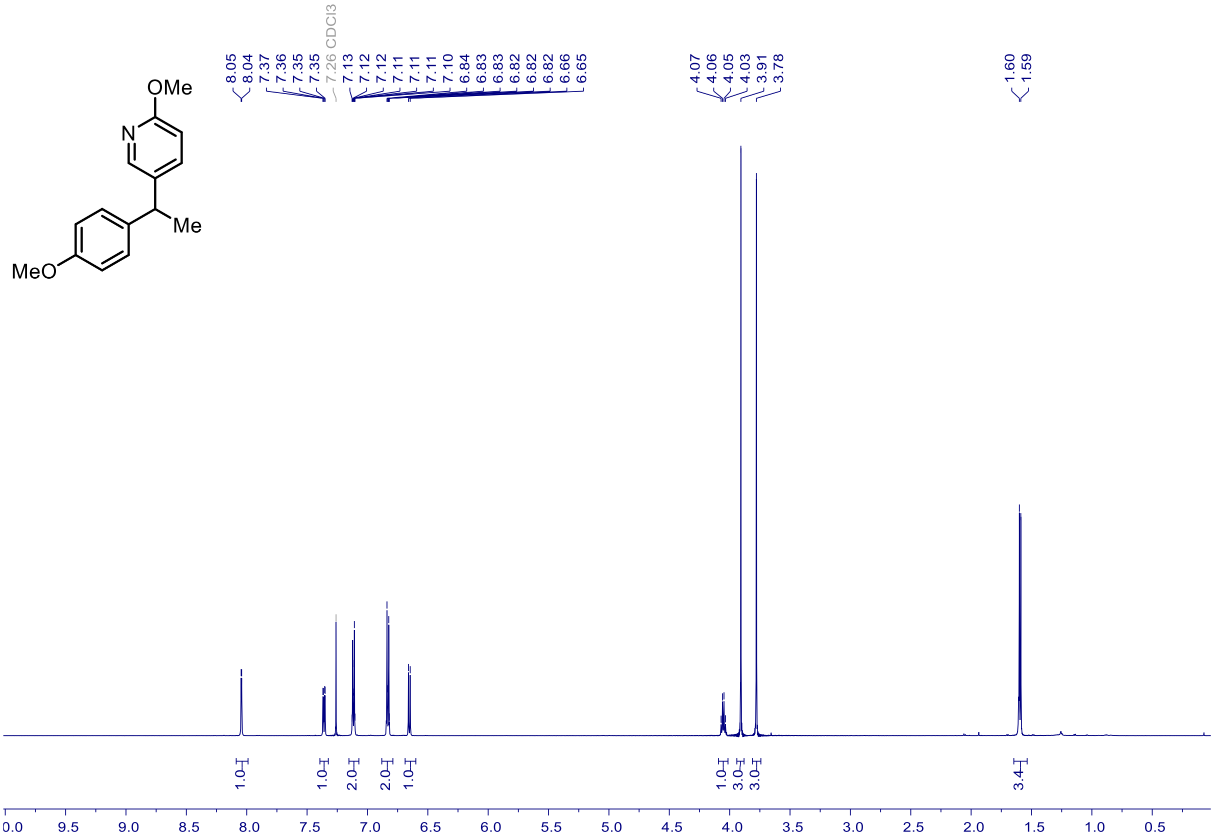

2aj – ^{13}C NMR (151 MHz, $CDCl_3$)

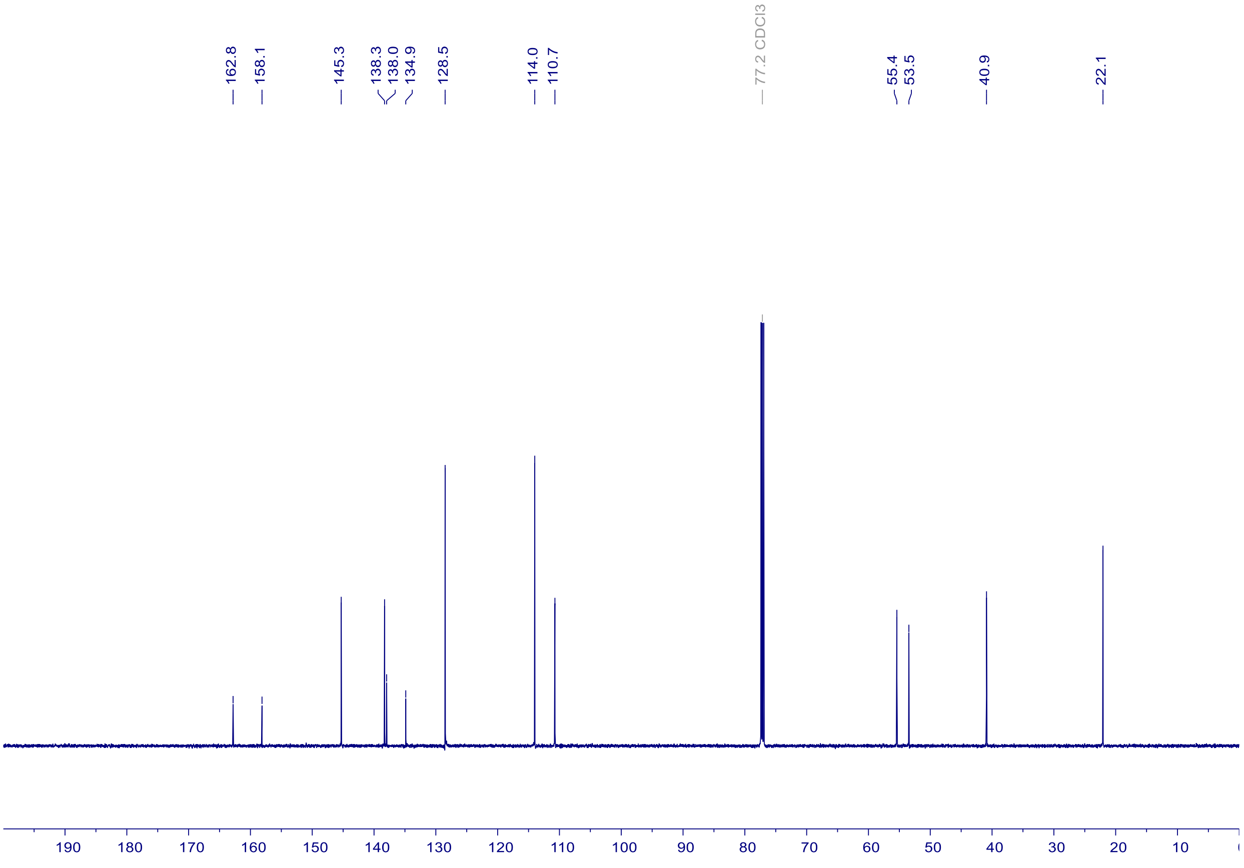

2ak – ^{1}H NMR (400 MHz, $CDCl_3$)

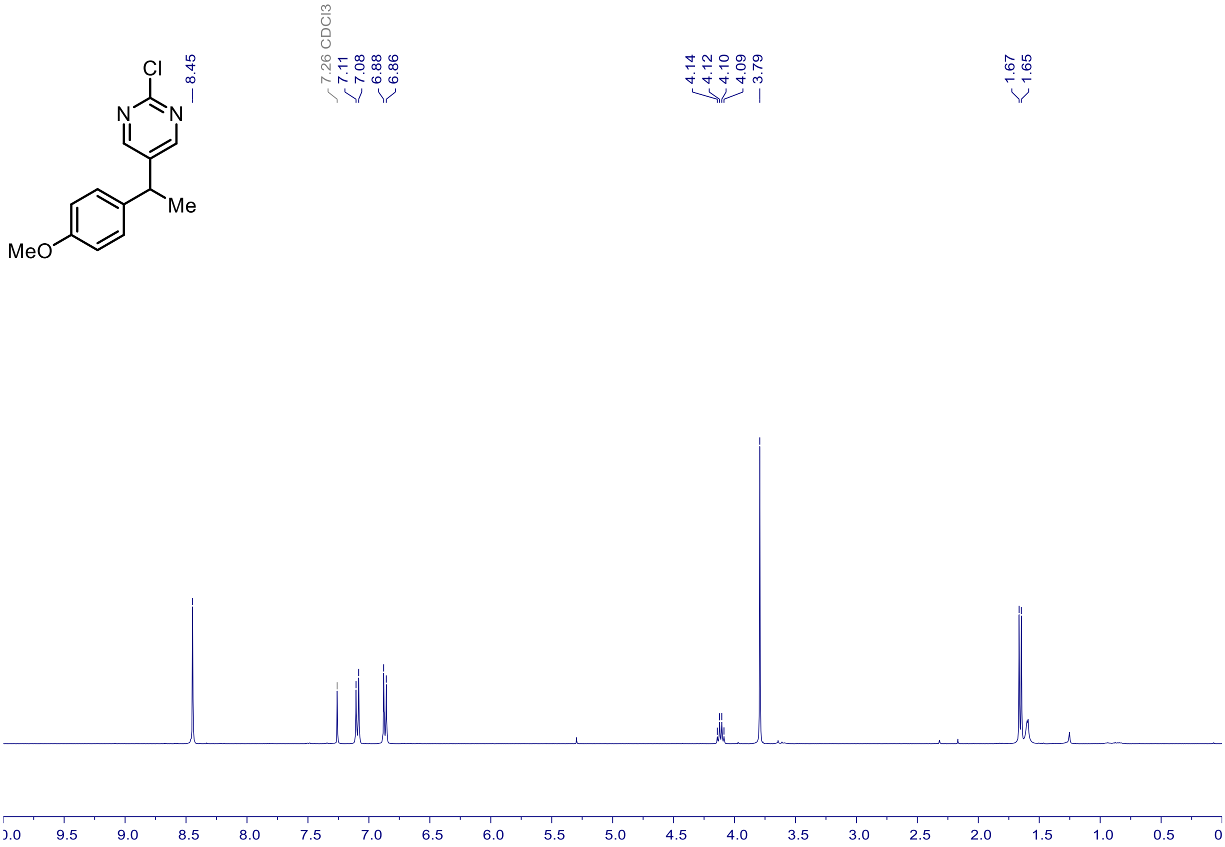

2ak – ^{13}C NMR (101 MHz, $CDCl_3$)

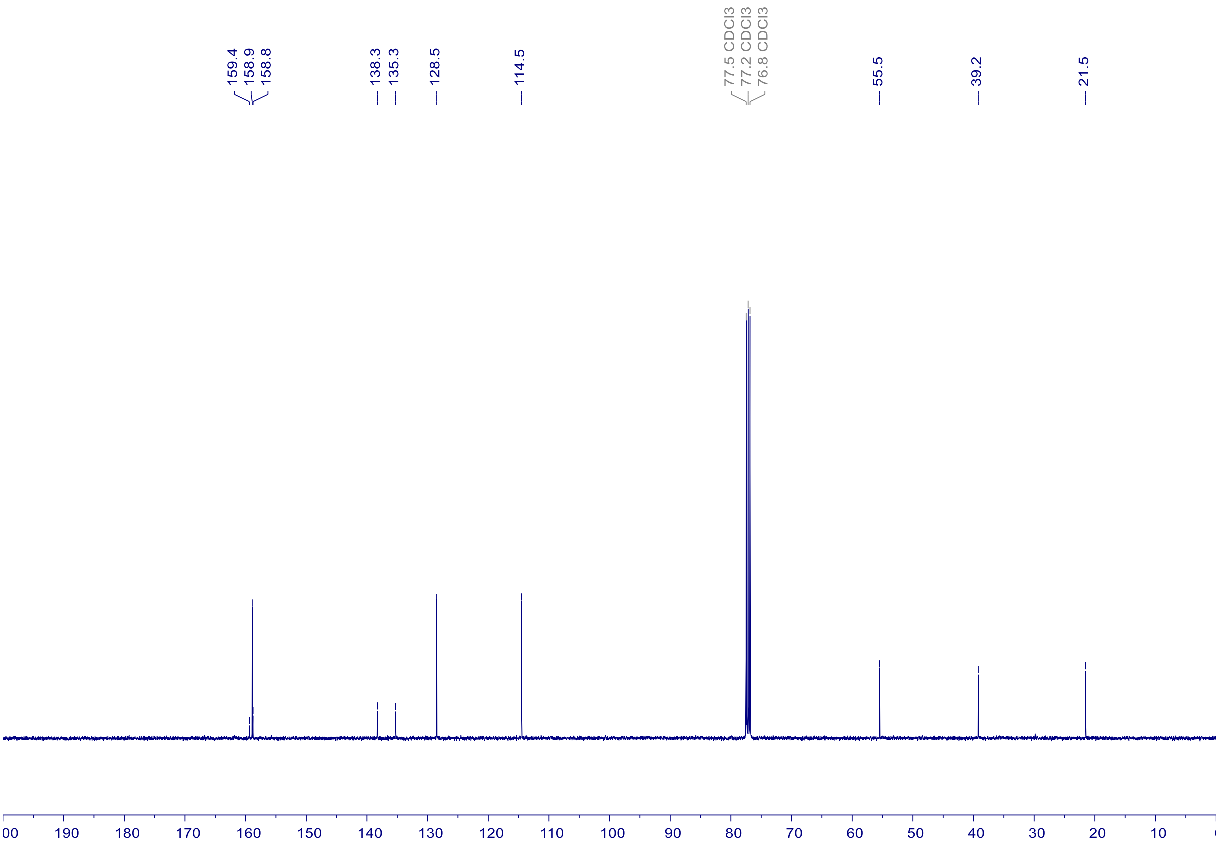

2al – ^{1}H NMR (400 MHz, $CDCl_3$)

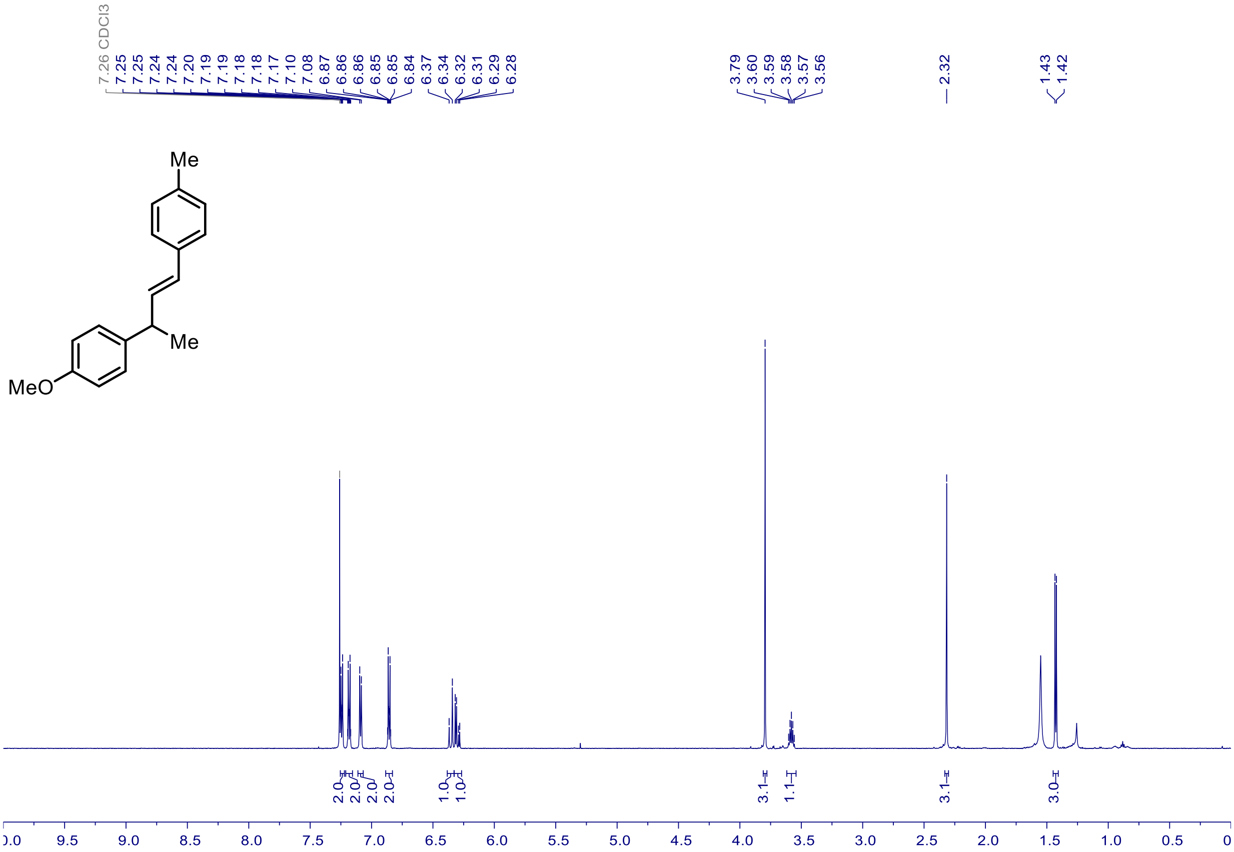

2al – ^{13}C NMR (101 MHz, $CDCl_3$)

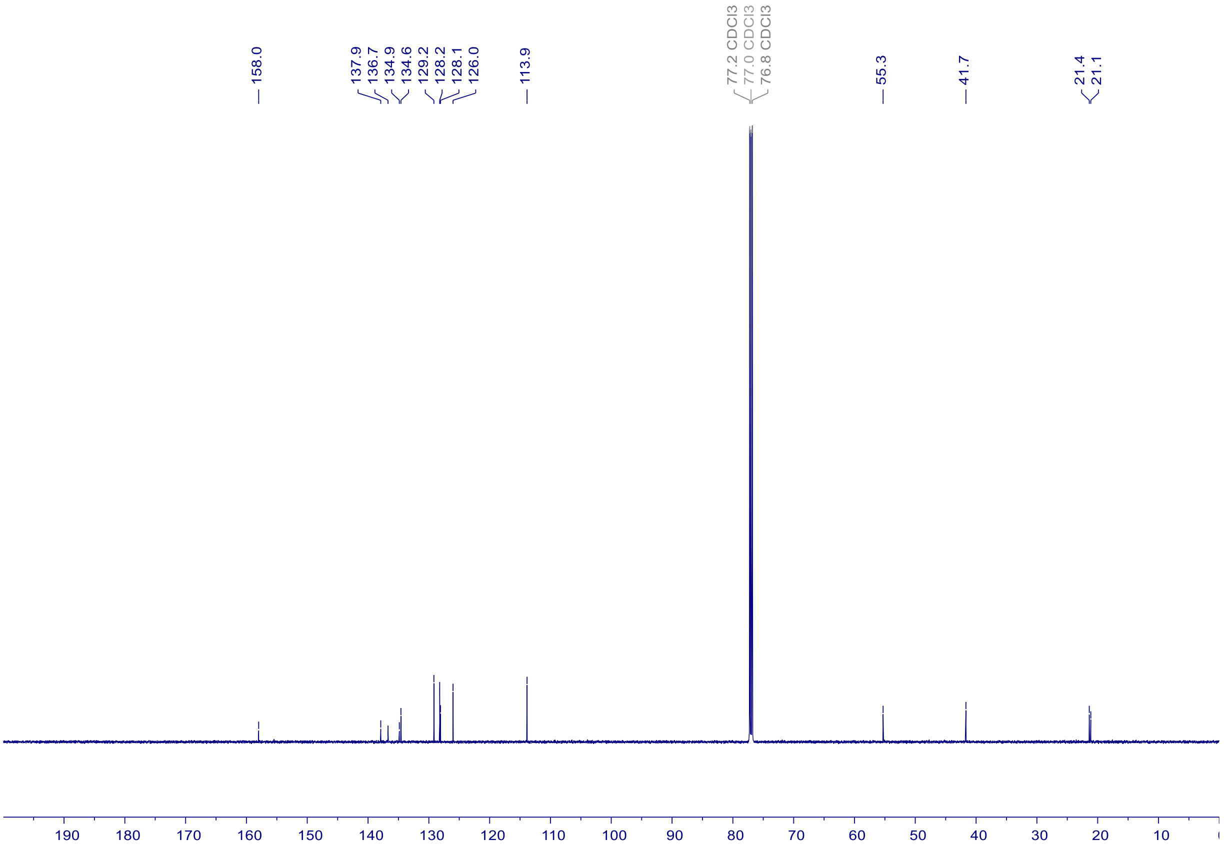

3f – ¹H NMR (400 MHz, $CDCl_3$)

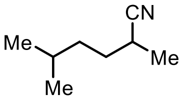

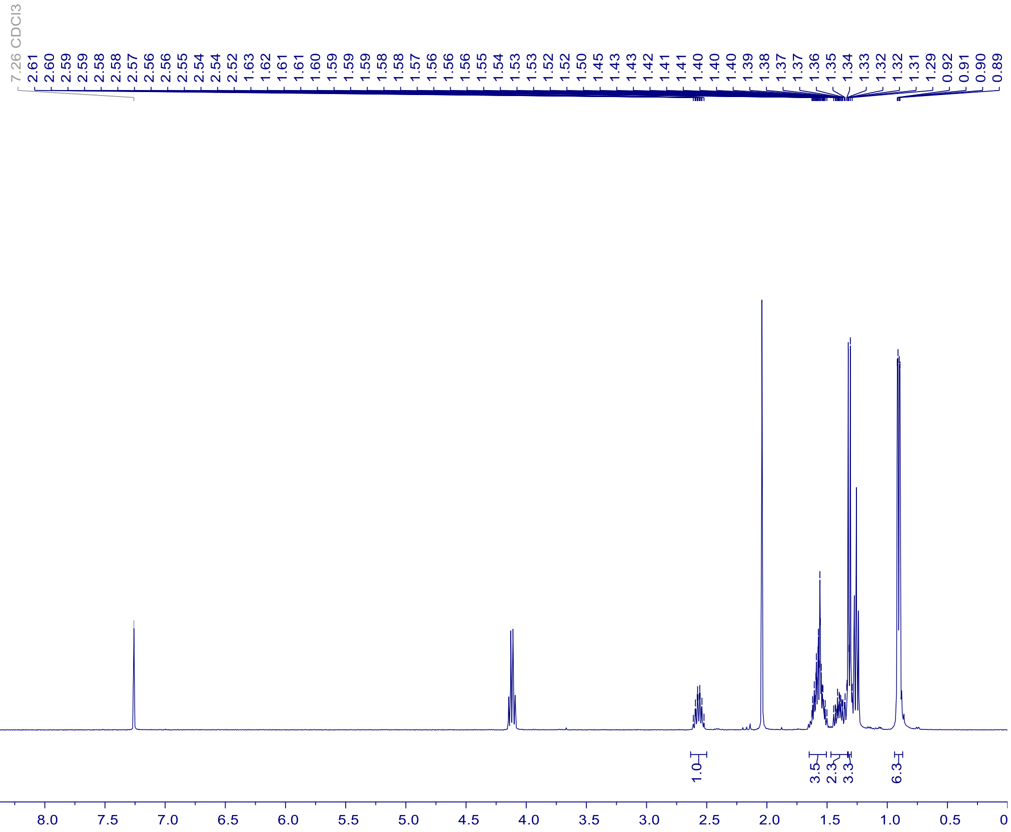

3f – ¹³C NMR (101 MHz, $CDCl_3$

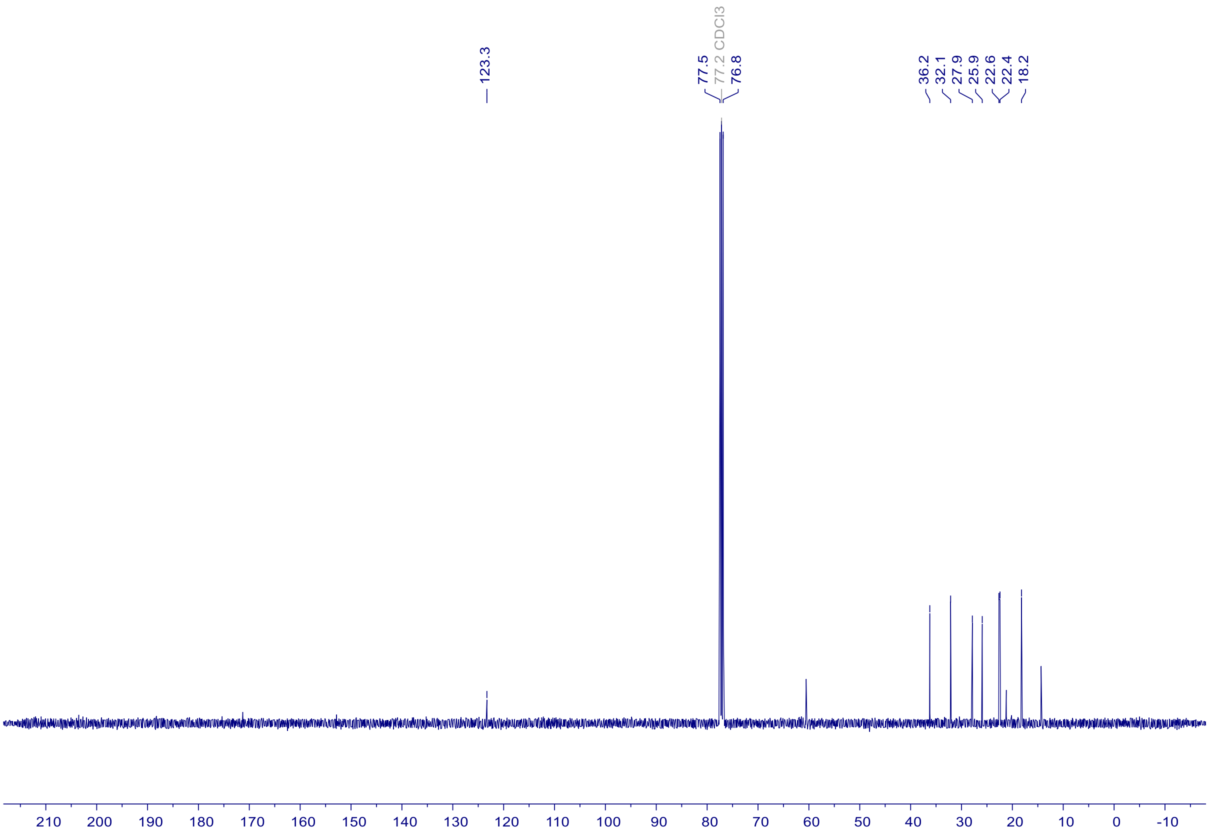

3ja – ^{1}H NMR (400 MHz, $CDCl_3$)

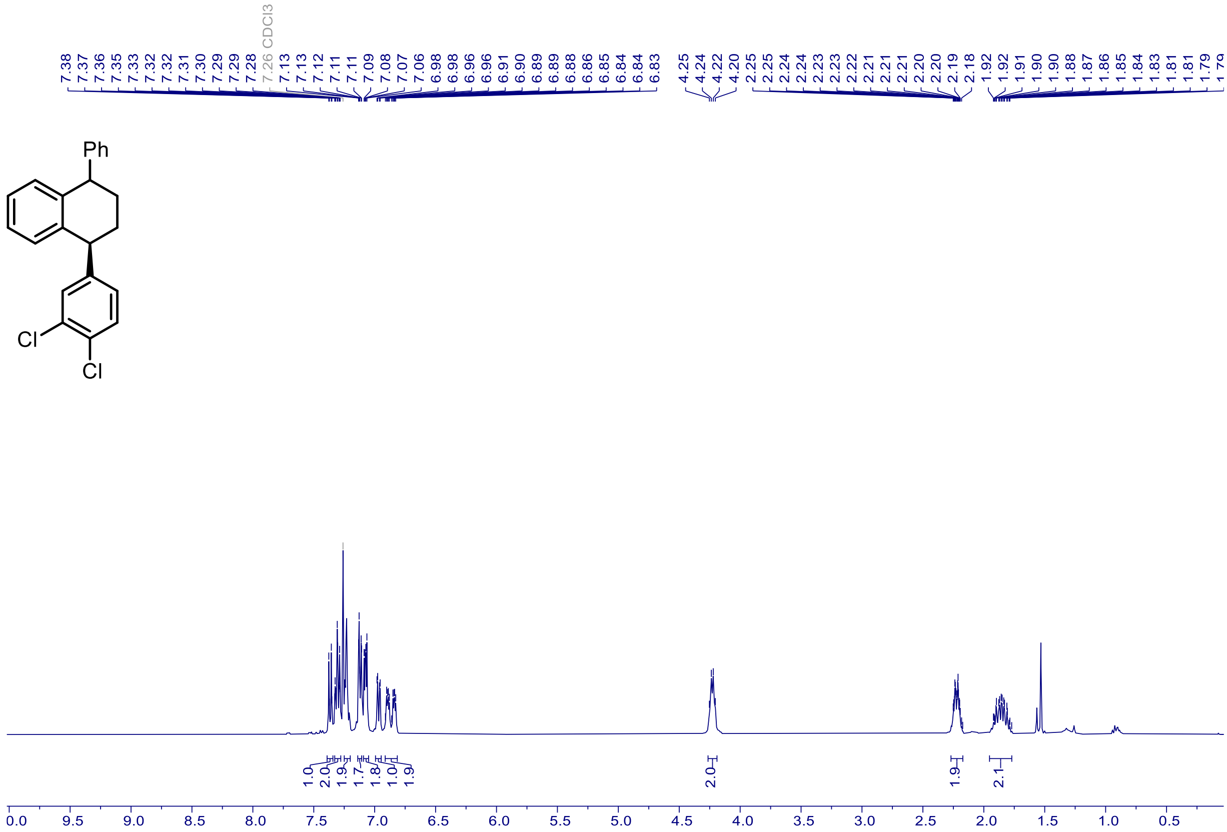

3ja – ^{13}C NMR (101 MHz, $CDCl_3$)

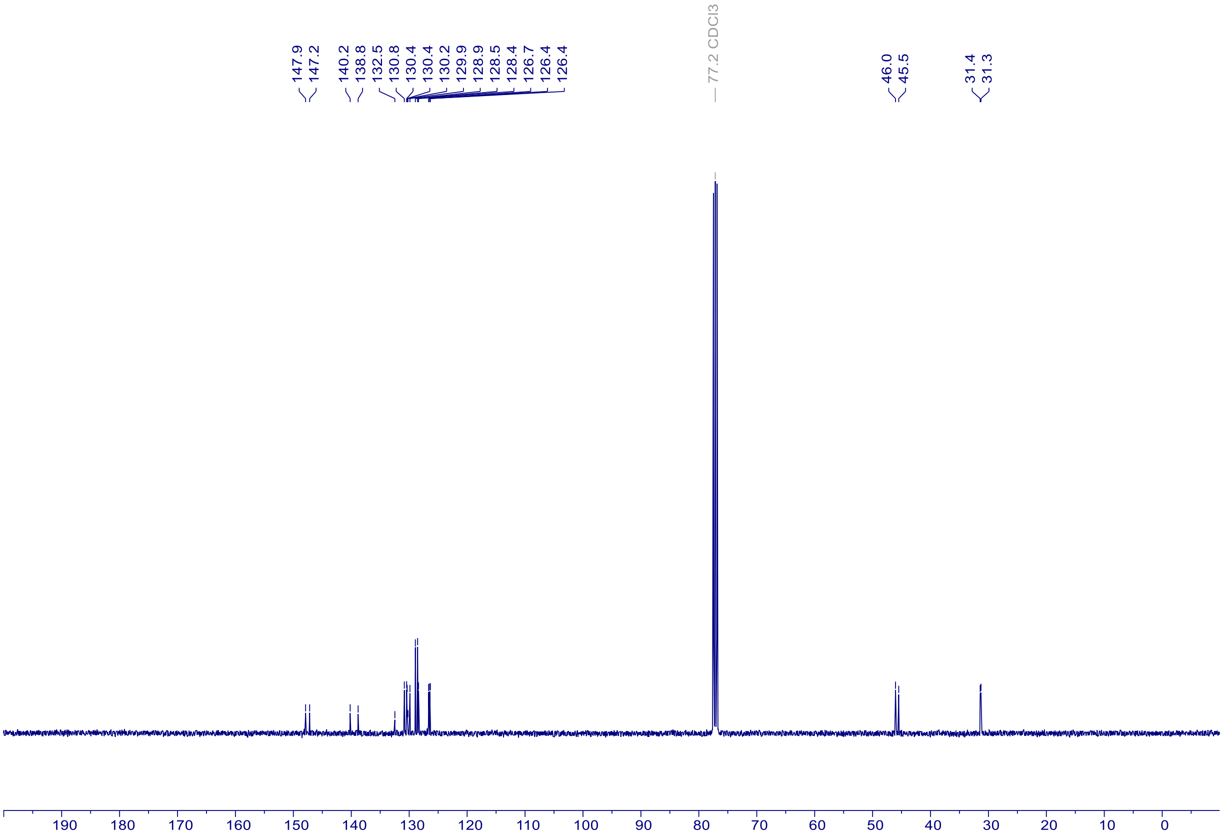

3jb – ^{1}H NMR (400 MHz, $CDCl_3$)

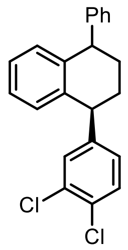

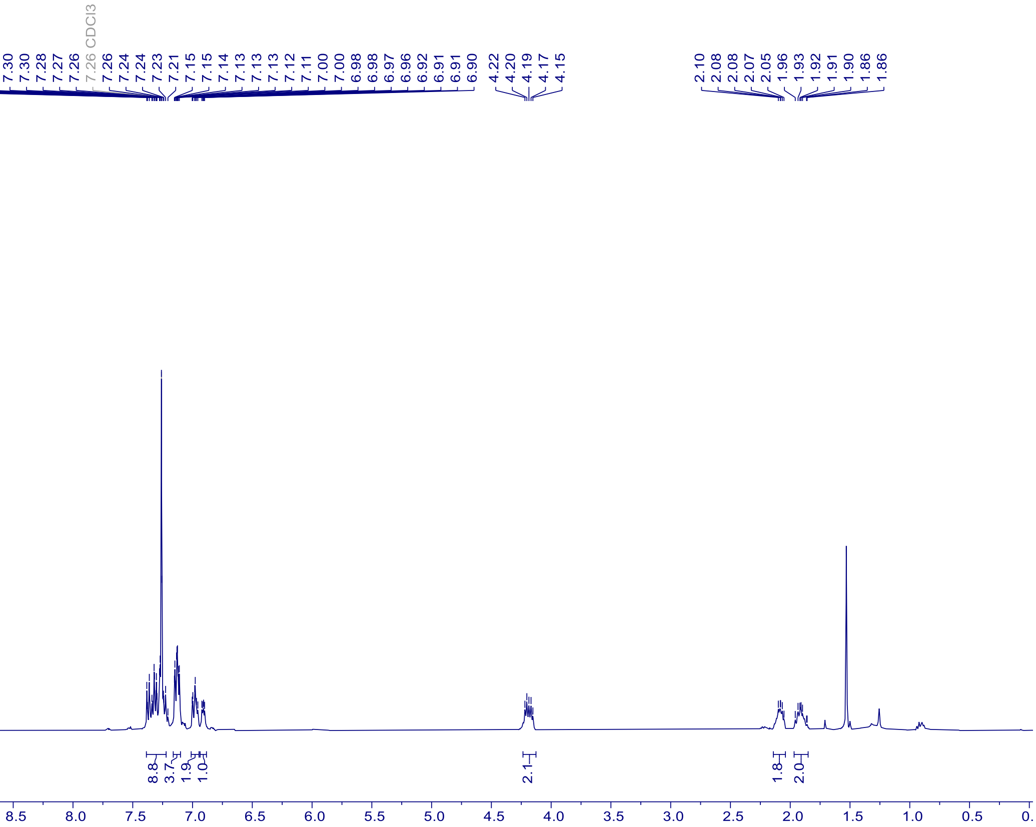

3jb – ^{13}C NMR (101 MHz, $CDCl_3$)

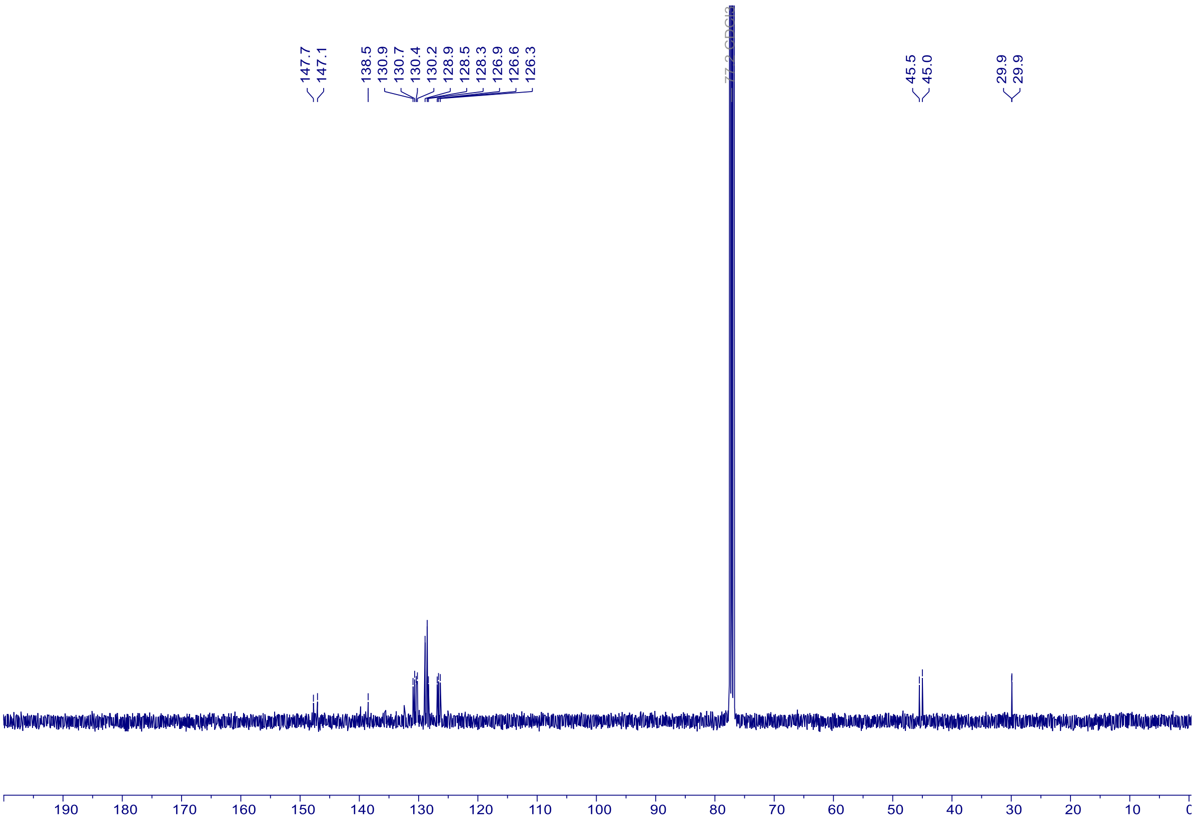

3k – ^{1}H NMR (600 MHz, $CDCl_3$)

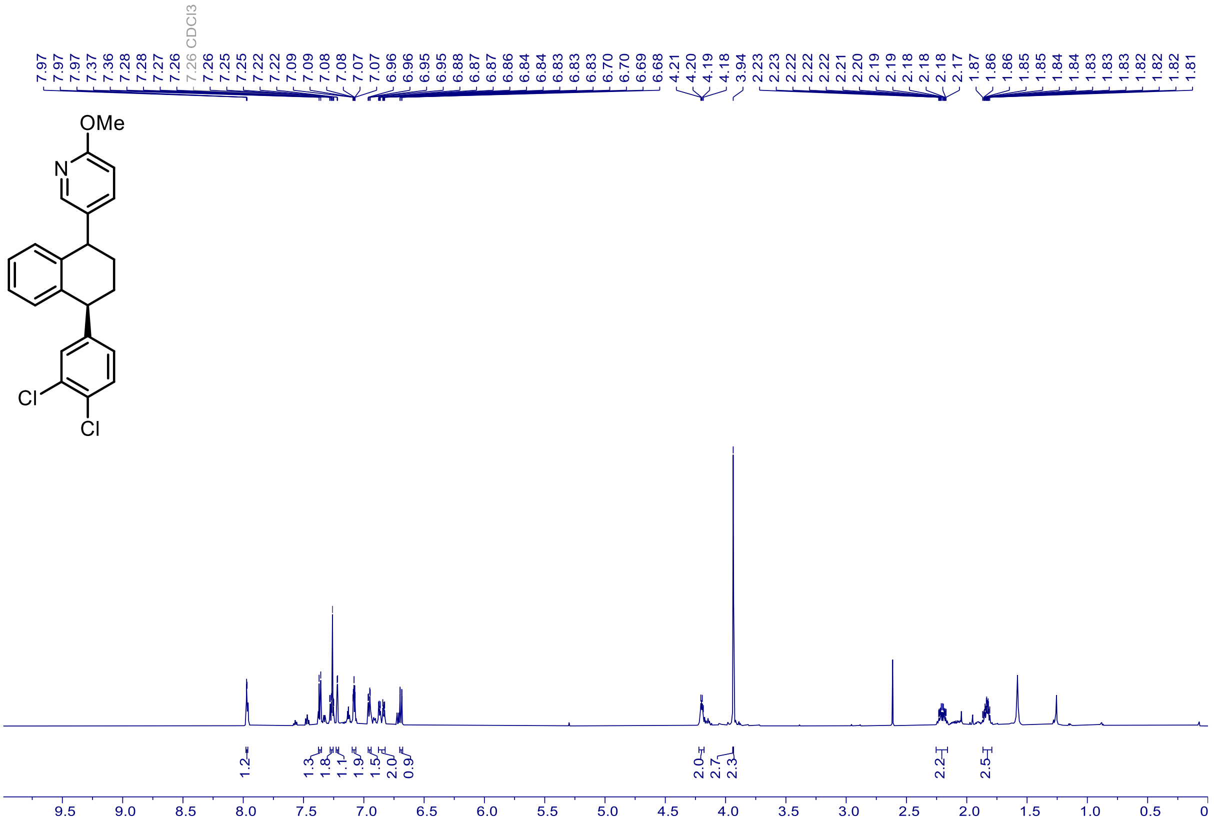

3k – ^{13}C NMR (101 MHz, $CDCl_3$)

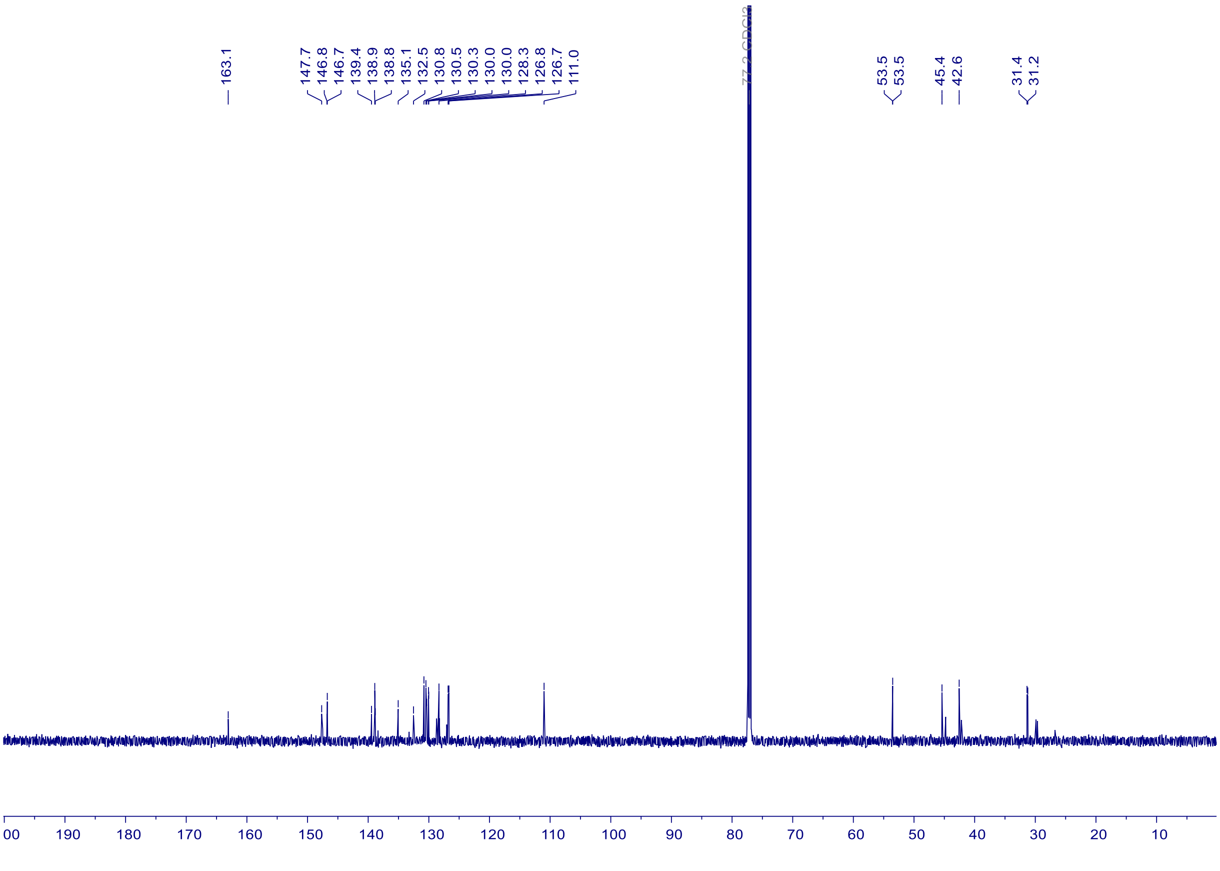

3la – ^{1}H NMR (400 MHz, $CDCl_3$)

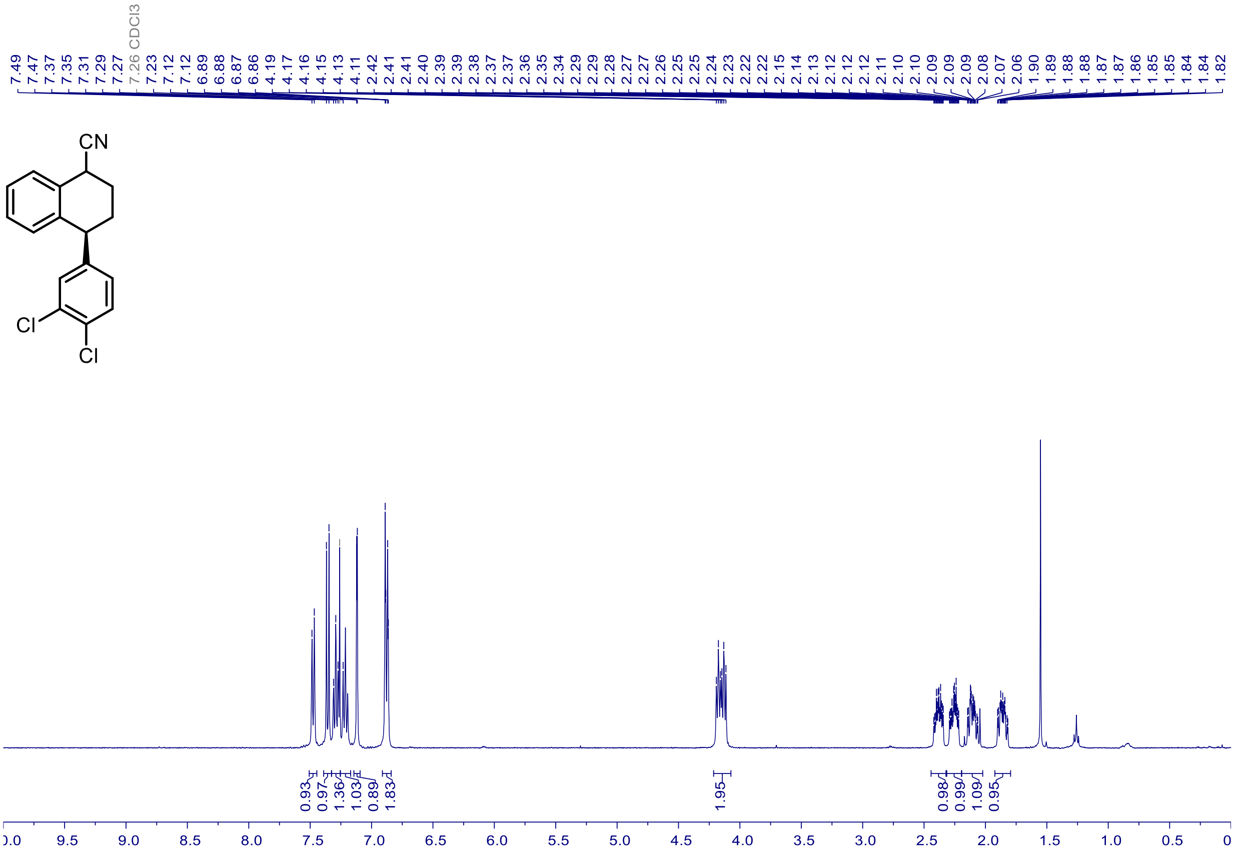

3la – ^{13}C NMR (101 MHz, $CDCl_3$)

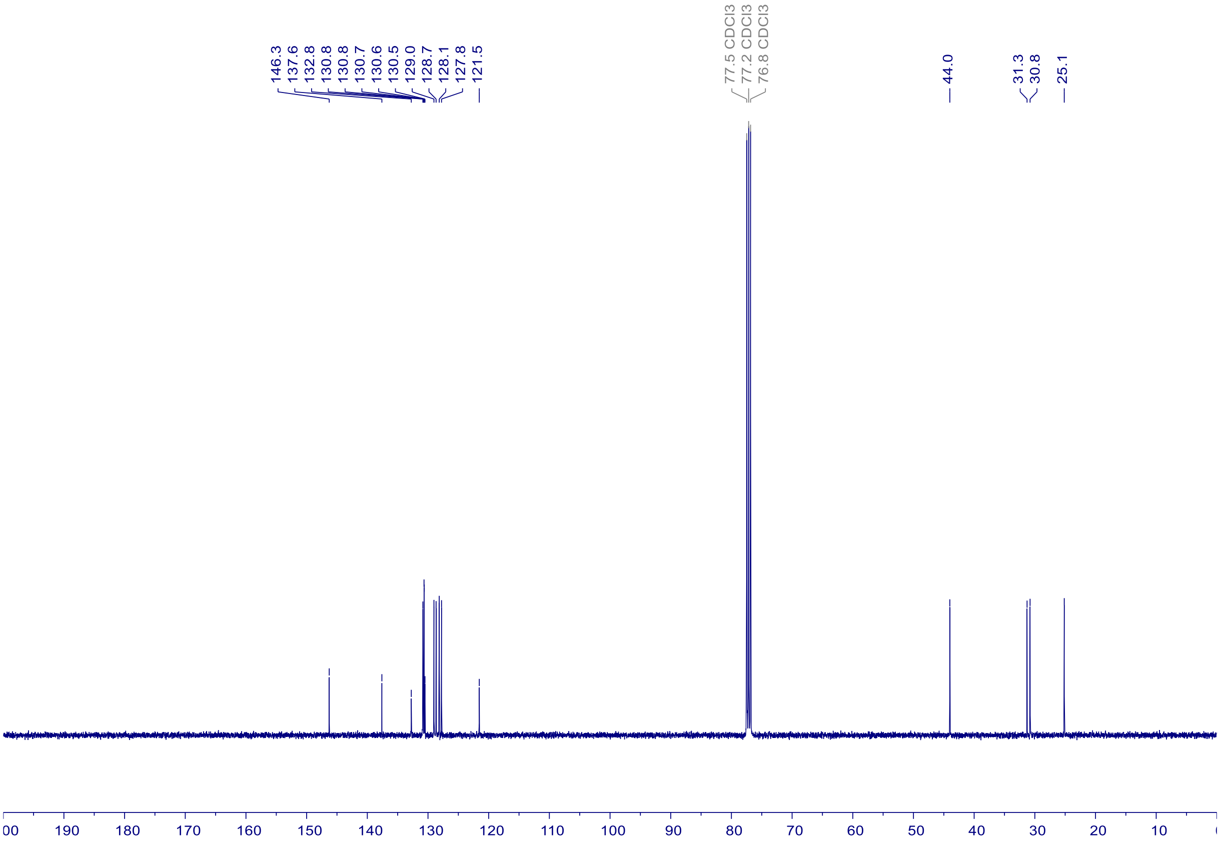

3lb – ^{1}H NMR (400 MHz, $CDCl_3$)

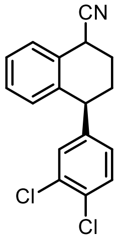

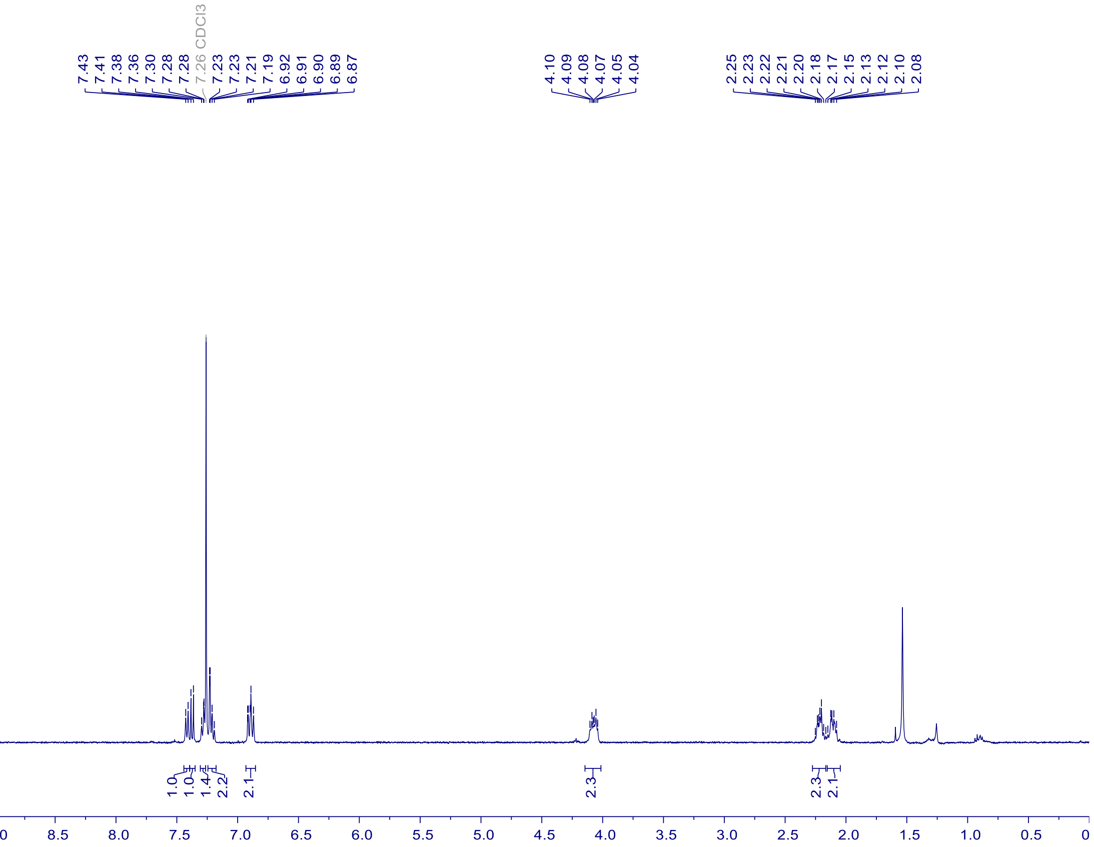

3lb – ^{13}C NMR (101 MHz, $CDCl_3$)

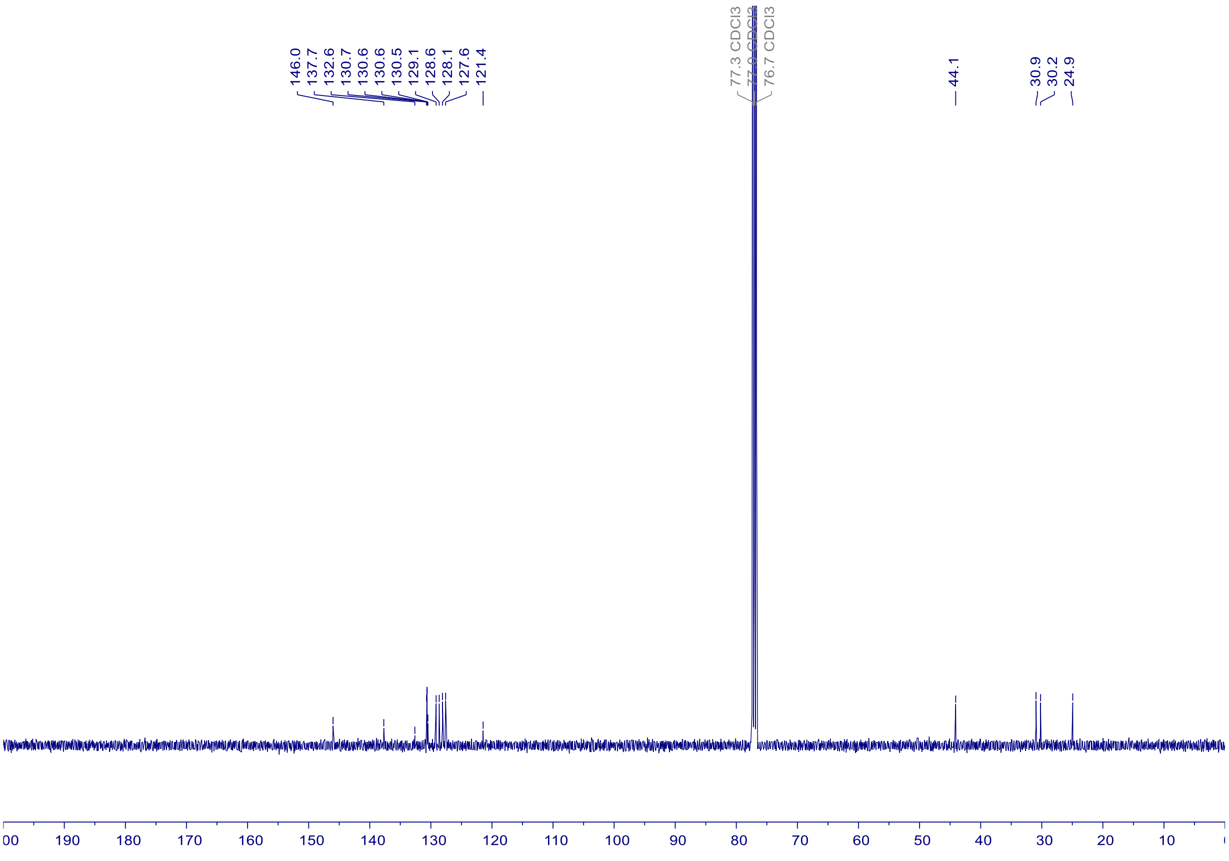

3m – ^{1}H NMR (400 MHz, $CDCl_3$)

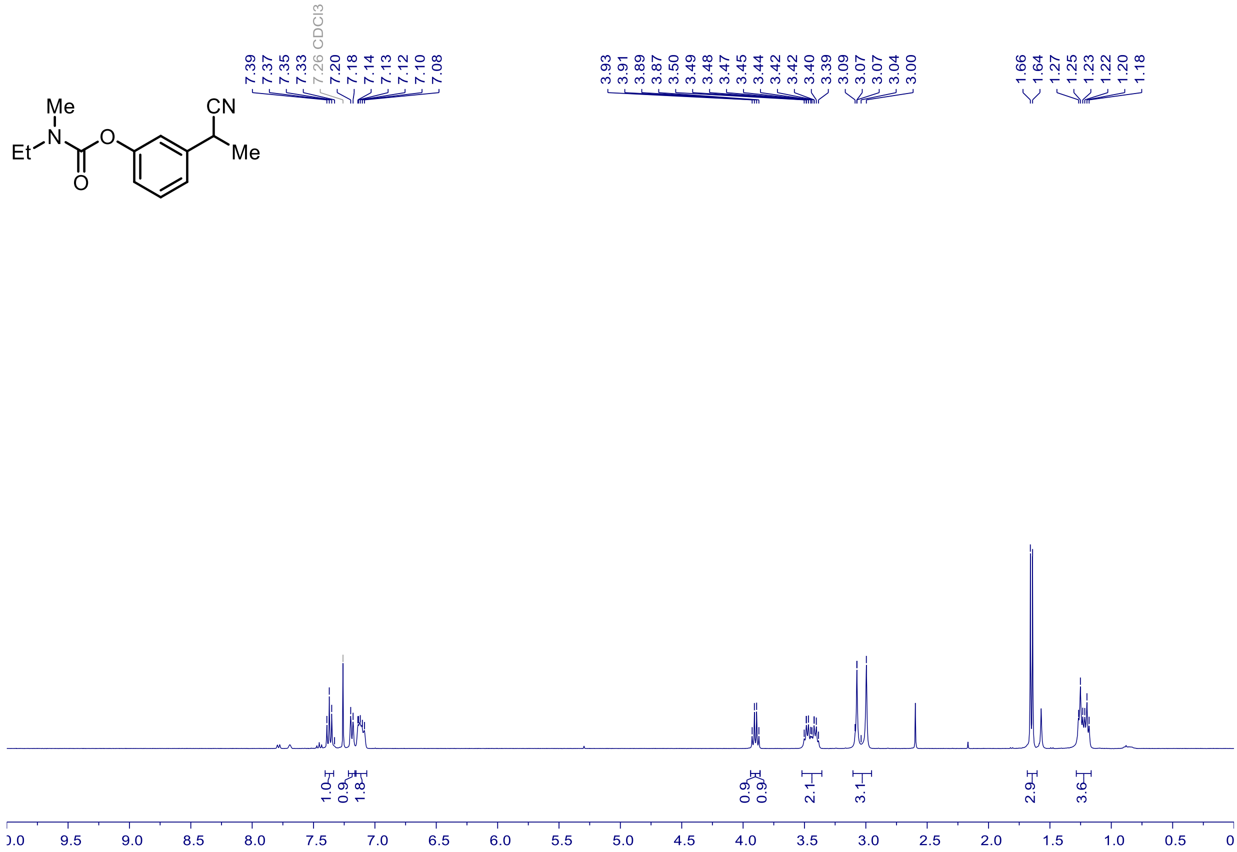

3m – ^{13}C NMR (101 MHz, $CDCl_3$)

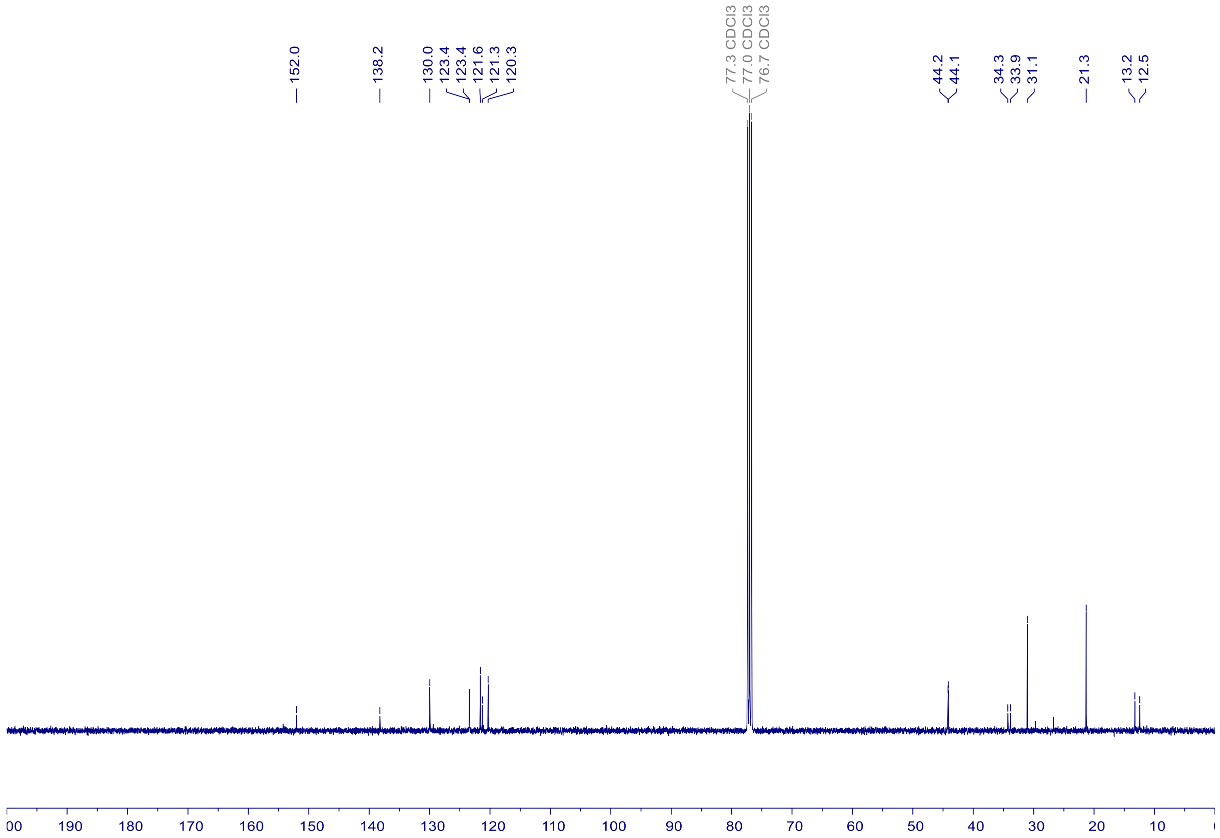

3o – ^{1}H NMR (400 MHz, $CDCl_3$)

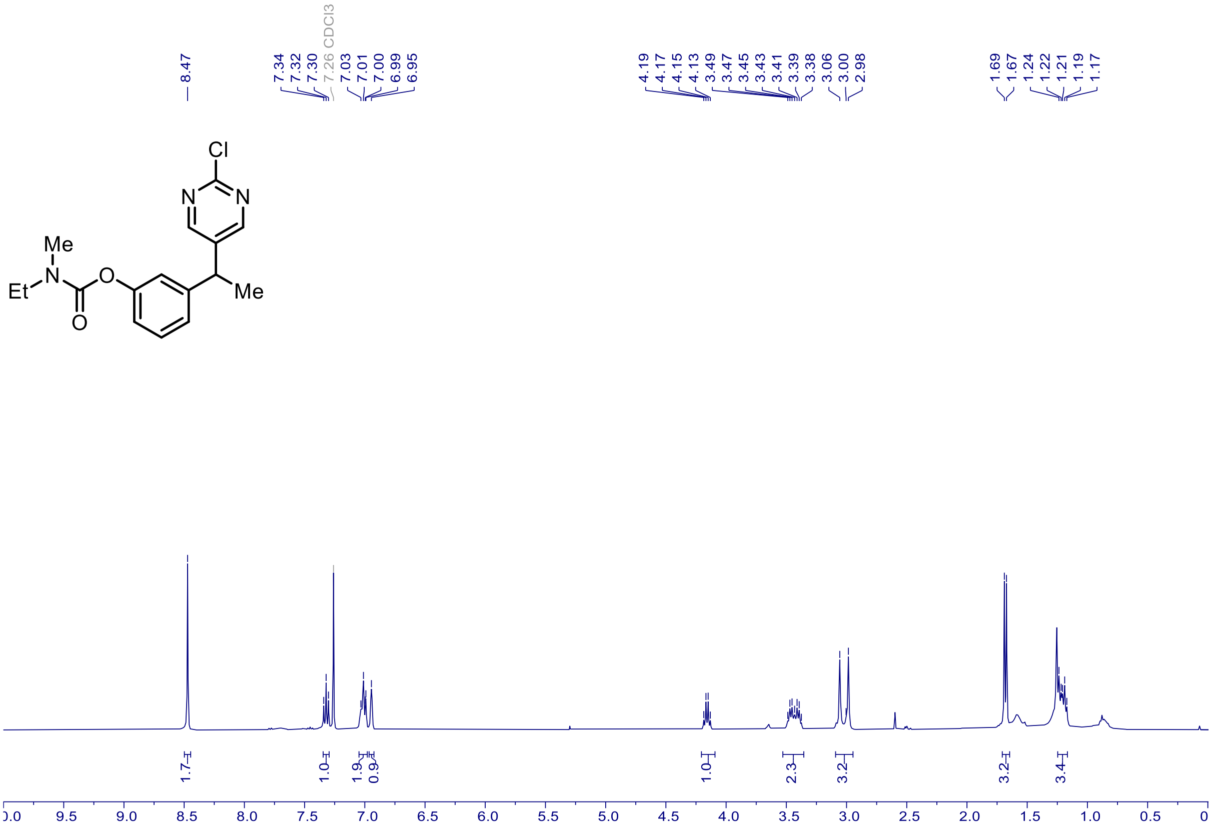

3o– ^{13}C NMR (101 MHz, $CDCl_3$)

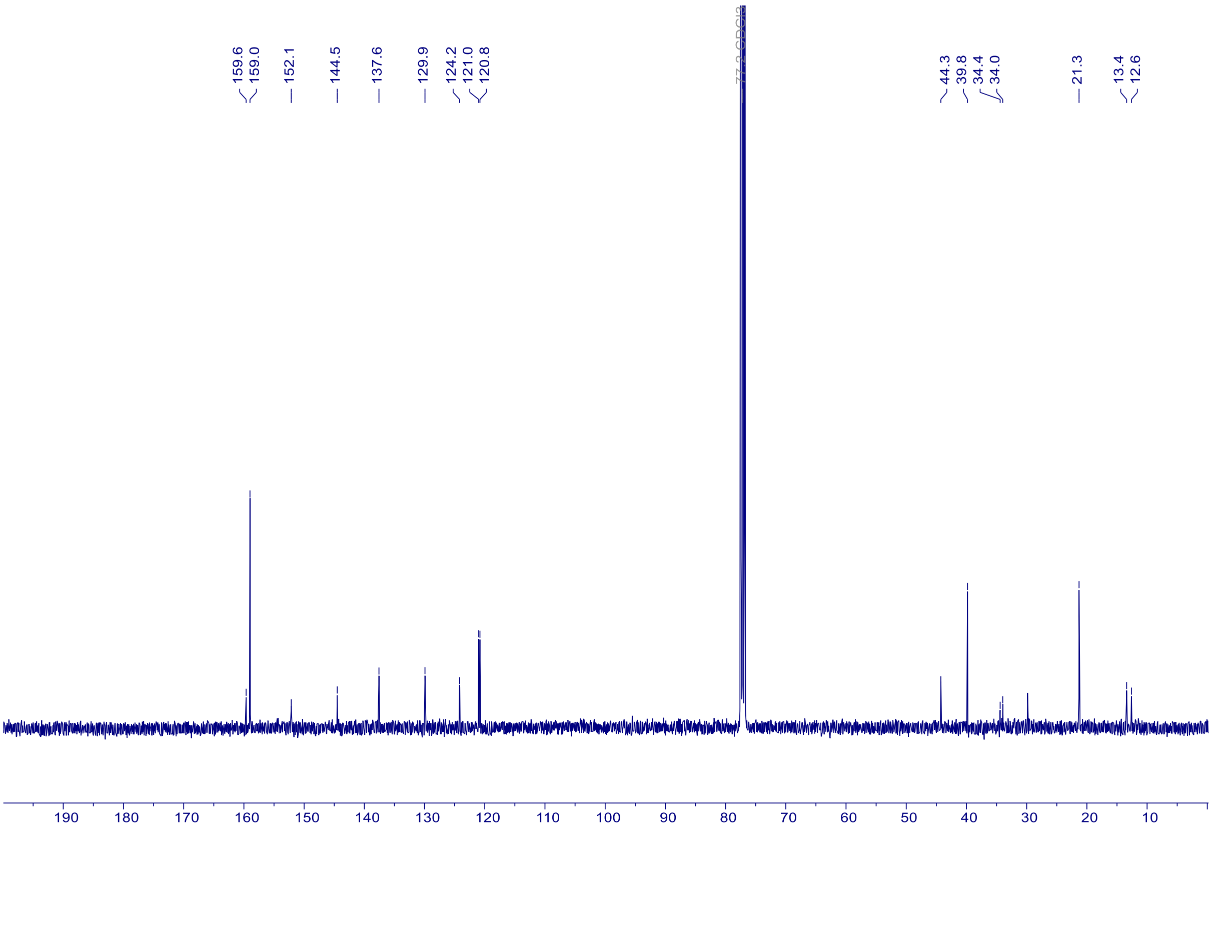

3p – ^{1}H NMR (400 MHz, $CDCl_3$)

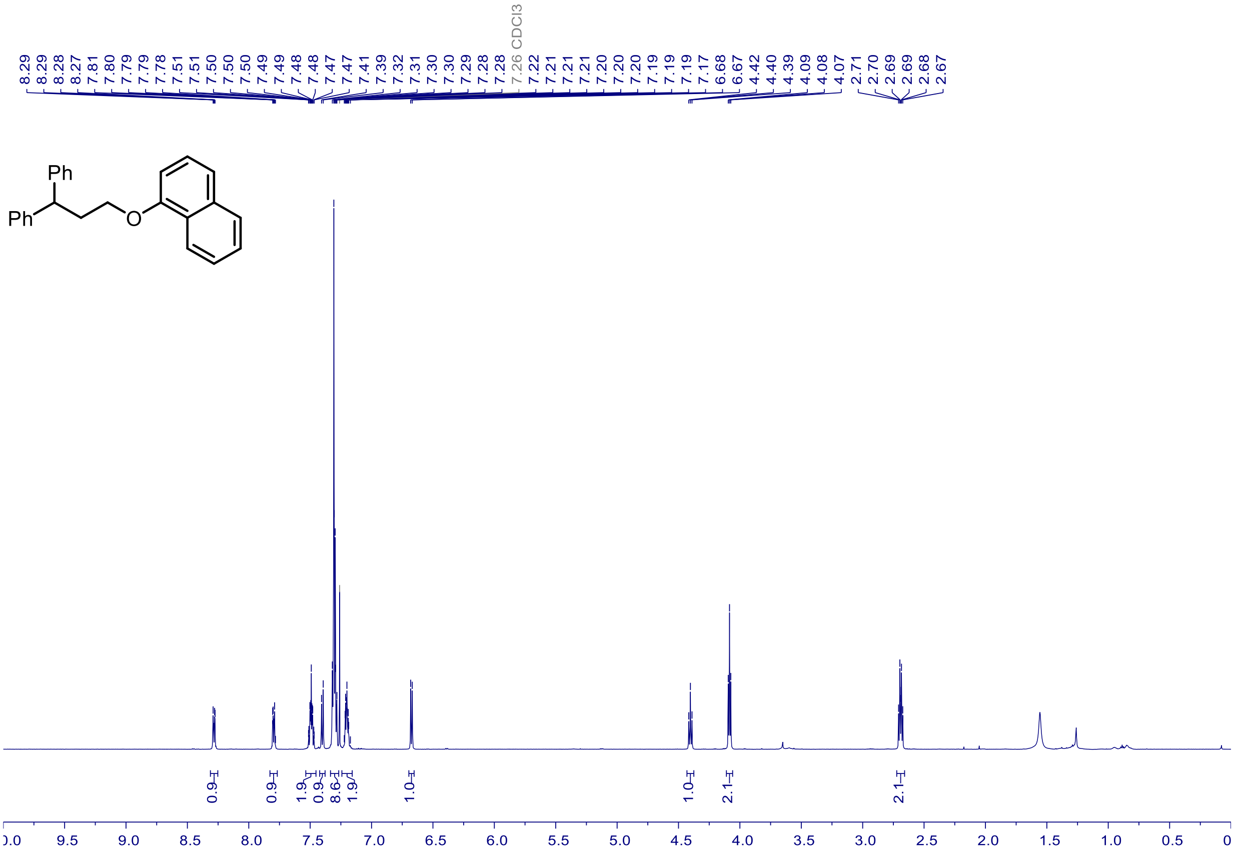

3p – ^{13}C NMR (101 MHz, $CDCl_3$)

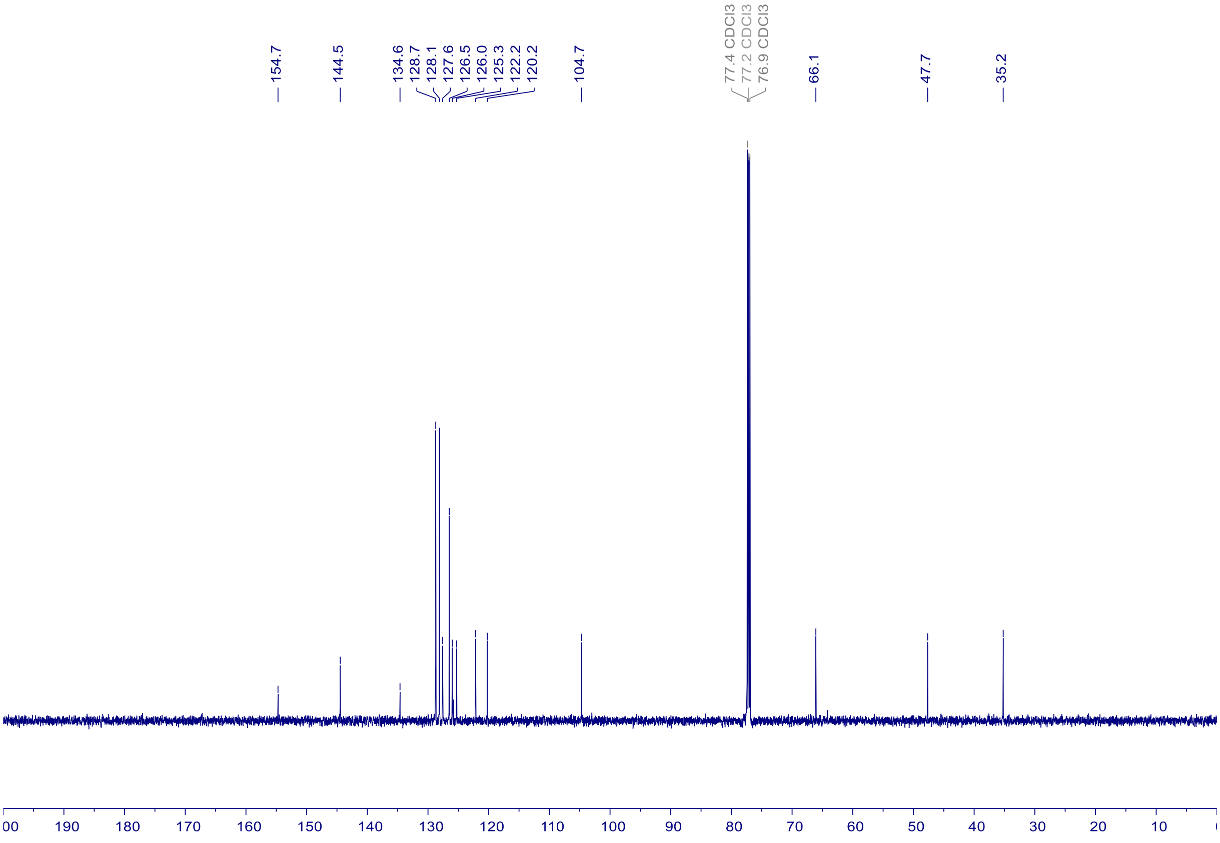

3q – ^{1}H NMR (600 MHz, $CDCl_3$)

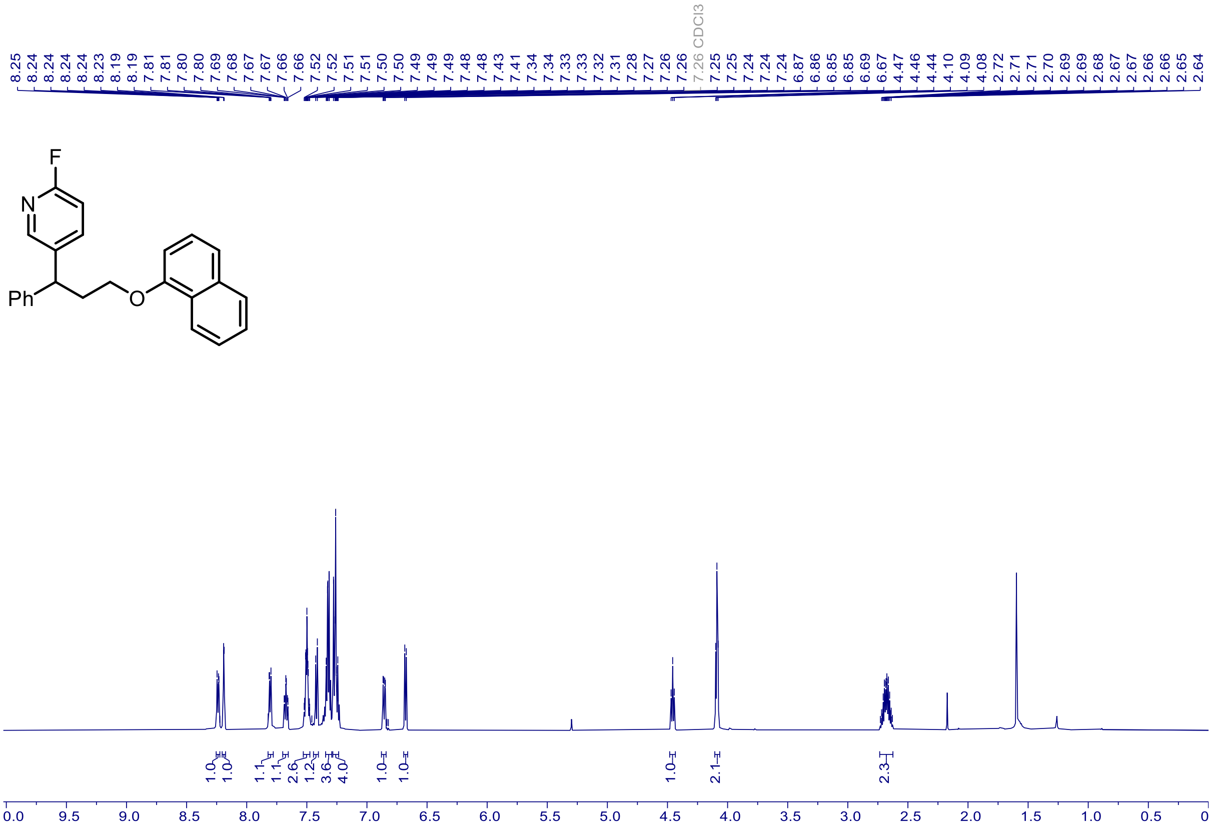

3q – ^{13}C NMR (151 MHz, $CDCl_3$)

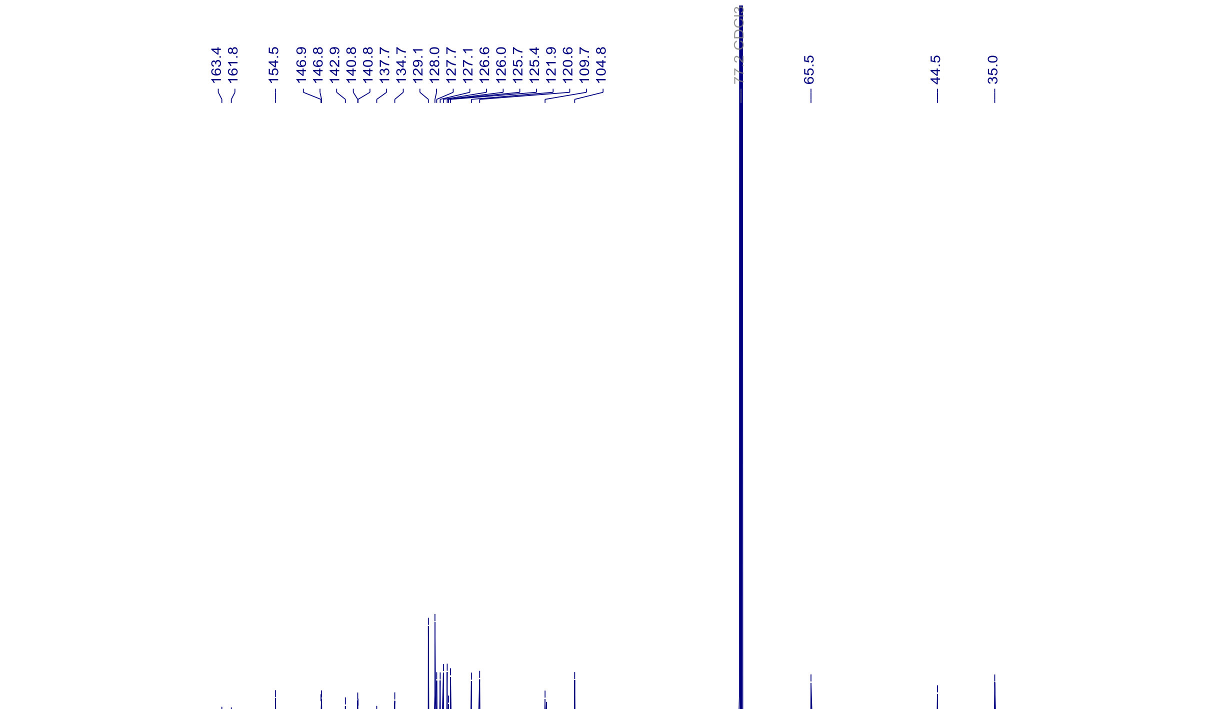

3q – ^{19}F NMR (565 MHz, $CDCl_3$)

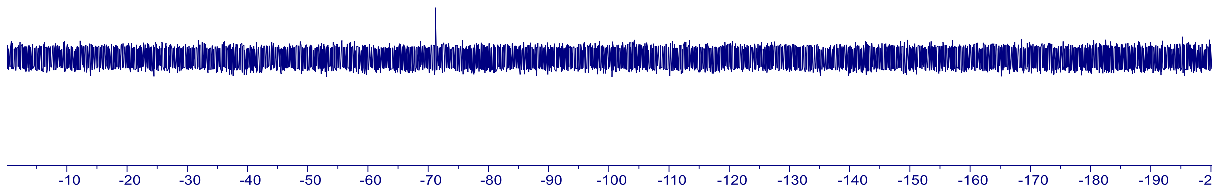

3r – ^{1}H NMR (400 MHz, $CDCl_3$)

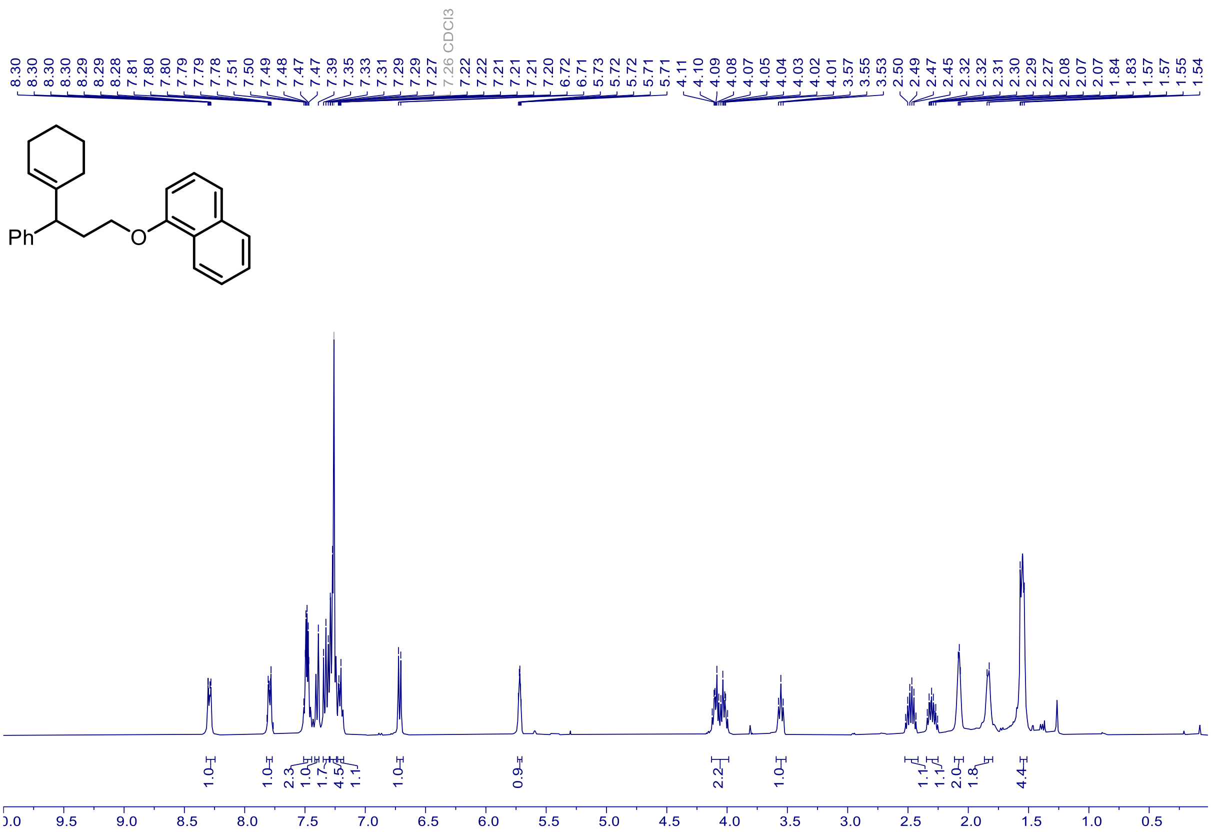

3r – ^{13}C NMR (101 MHz, $CDCl_3$)

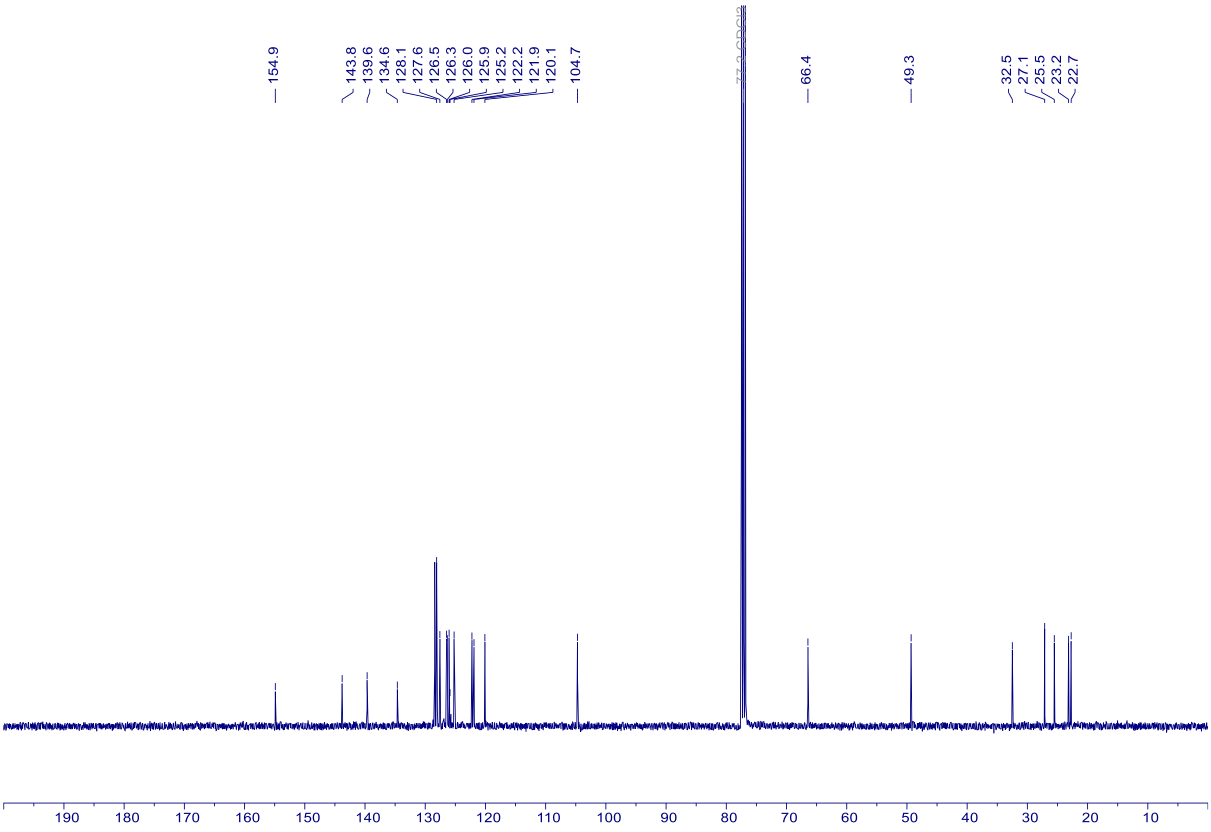

3s – ^{1}H NMR (400 MHz, $CDCl_3$)

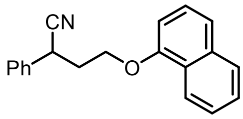

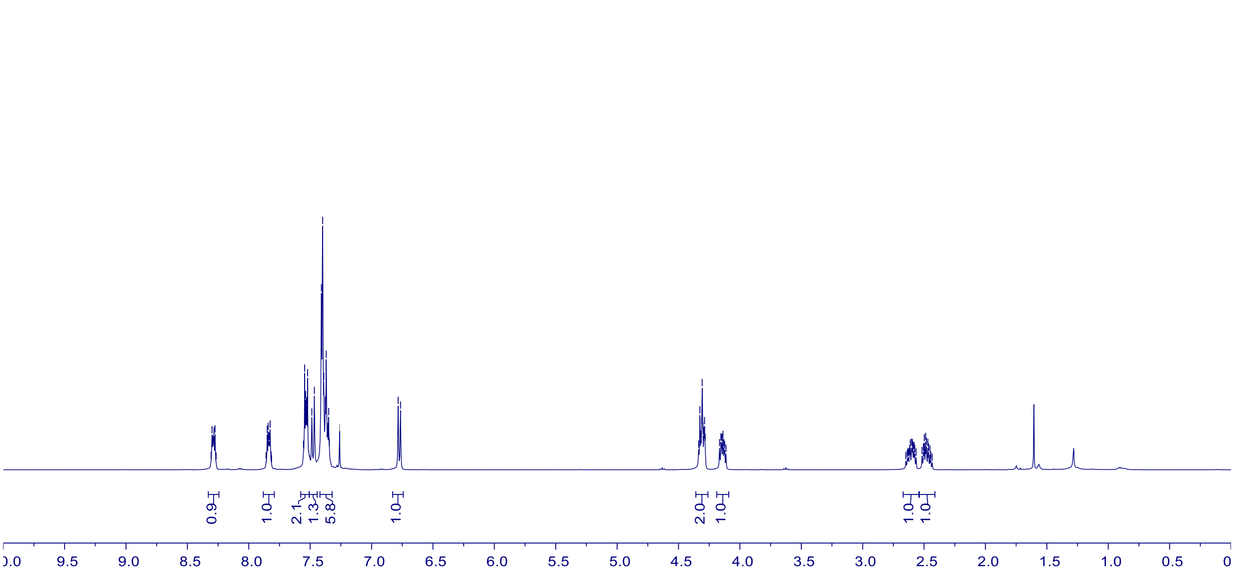

3s – ^{13}C NMR (101 MHz, $CDCl_3$)

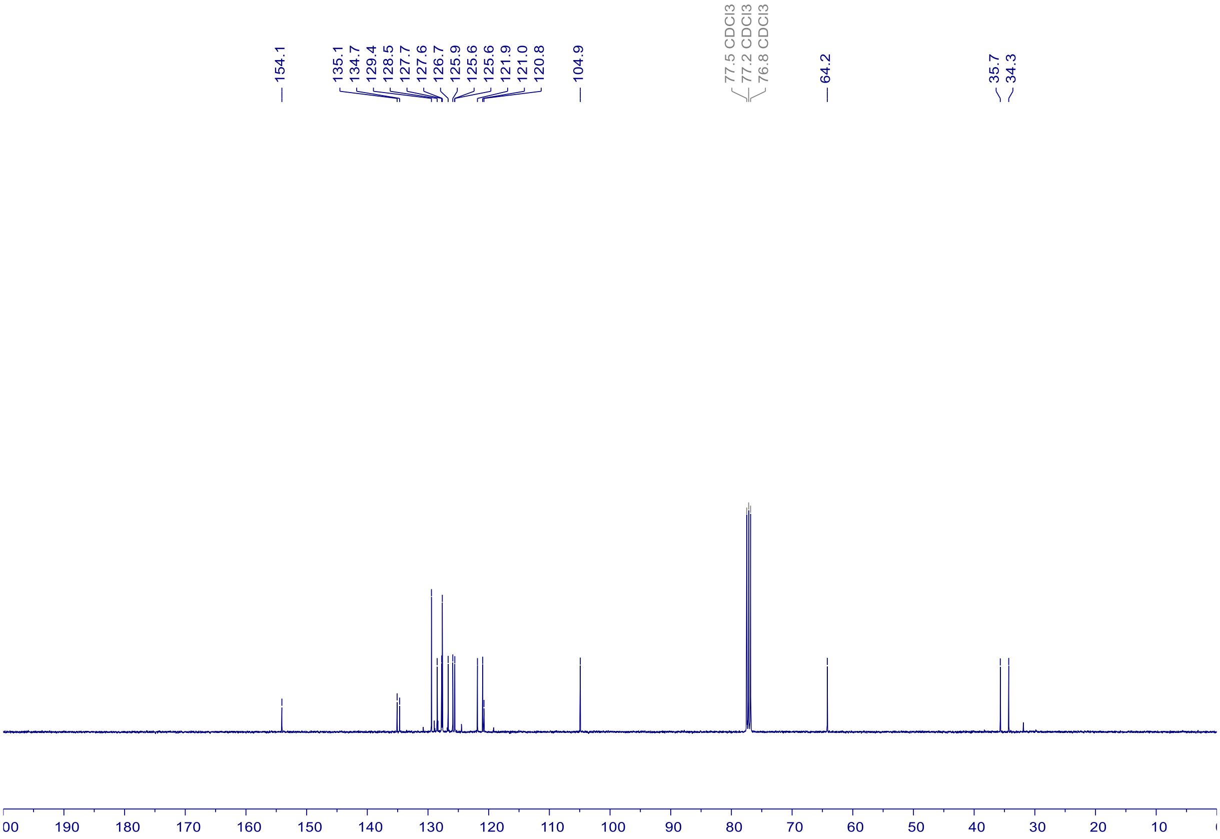

4c – ^{1}H NMR (400 MHz, $CDCl_3$)

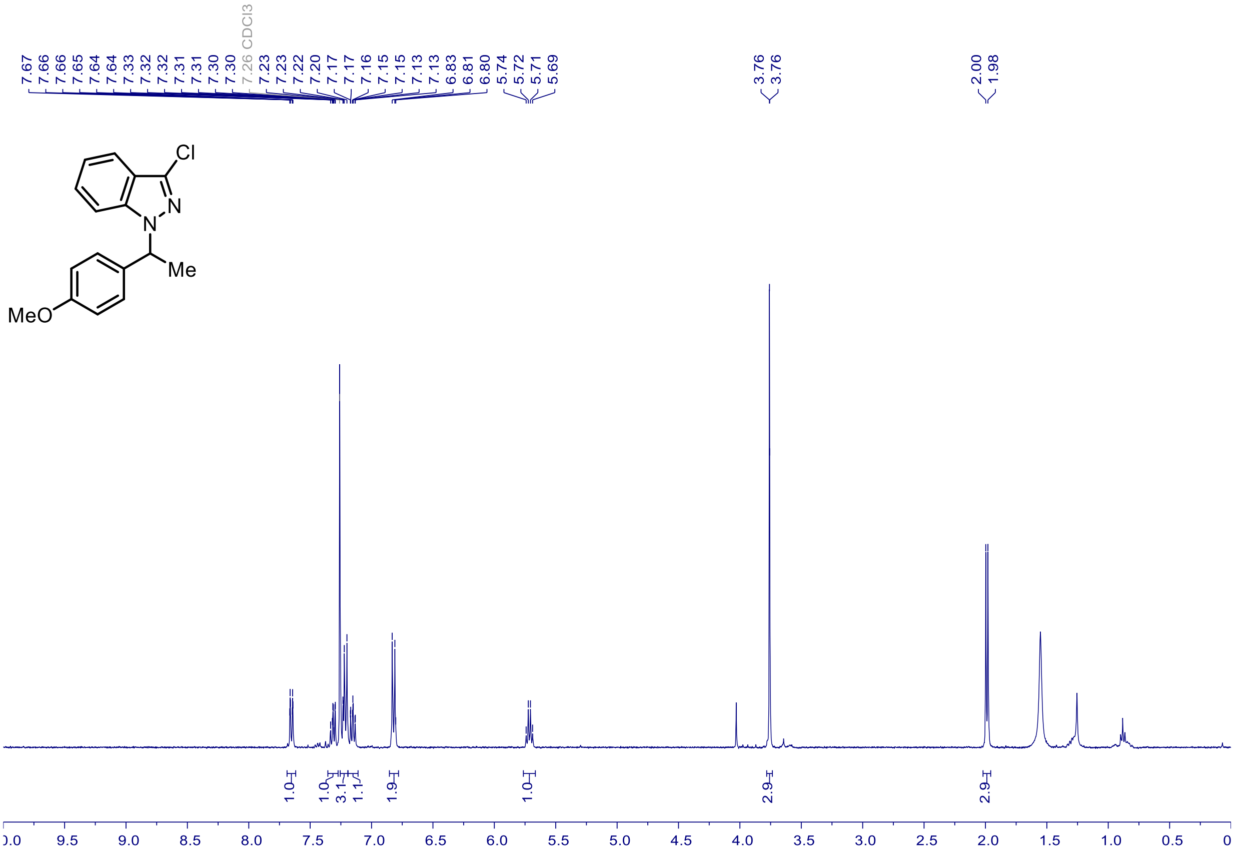

4c – ^{13}C NMR (101 MHz, $CDCl_3$)

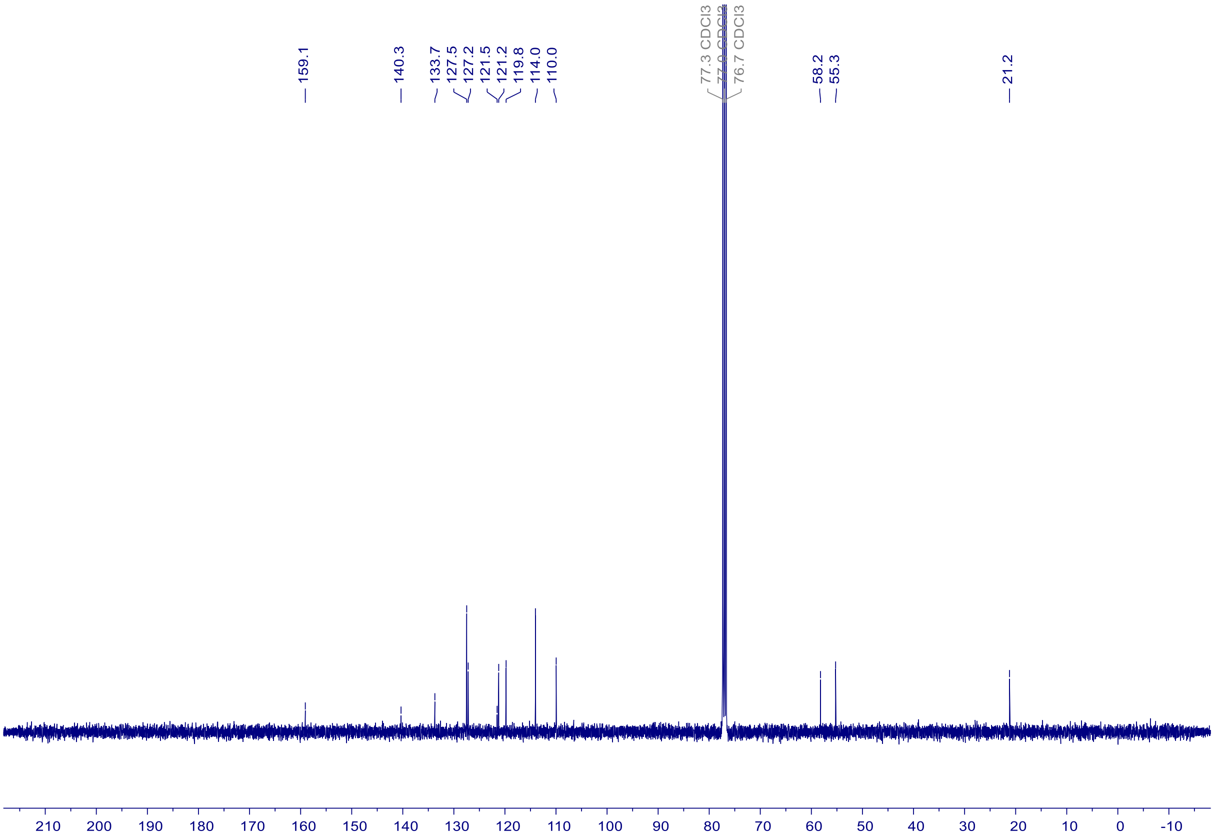

4f – ^{1}H NMR (600 MHz, $CDCl_3$)

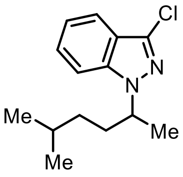

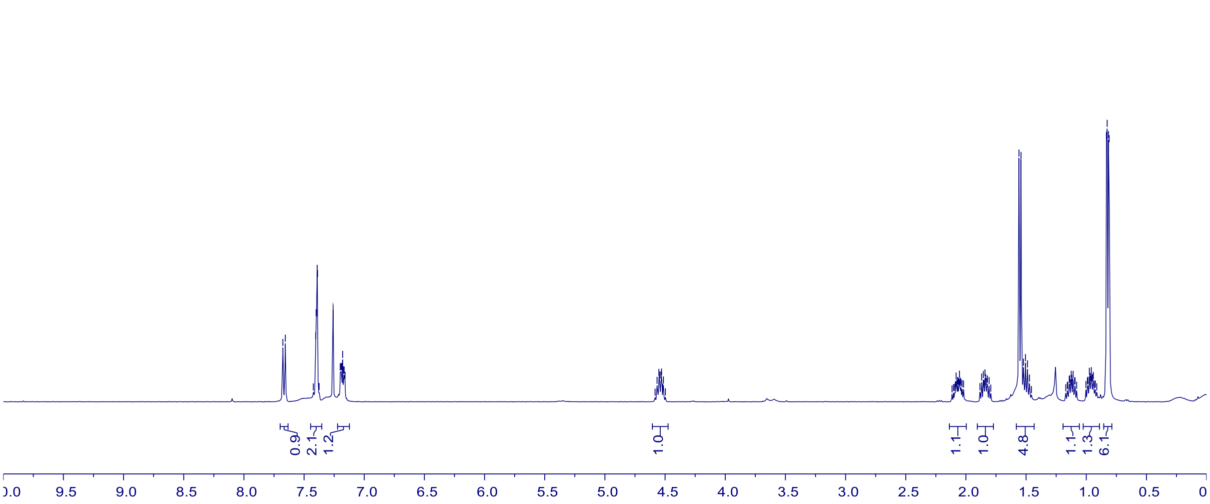

4f – ^{13}C NMR (151 MHz, $CDCl_3$)

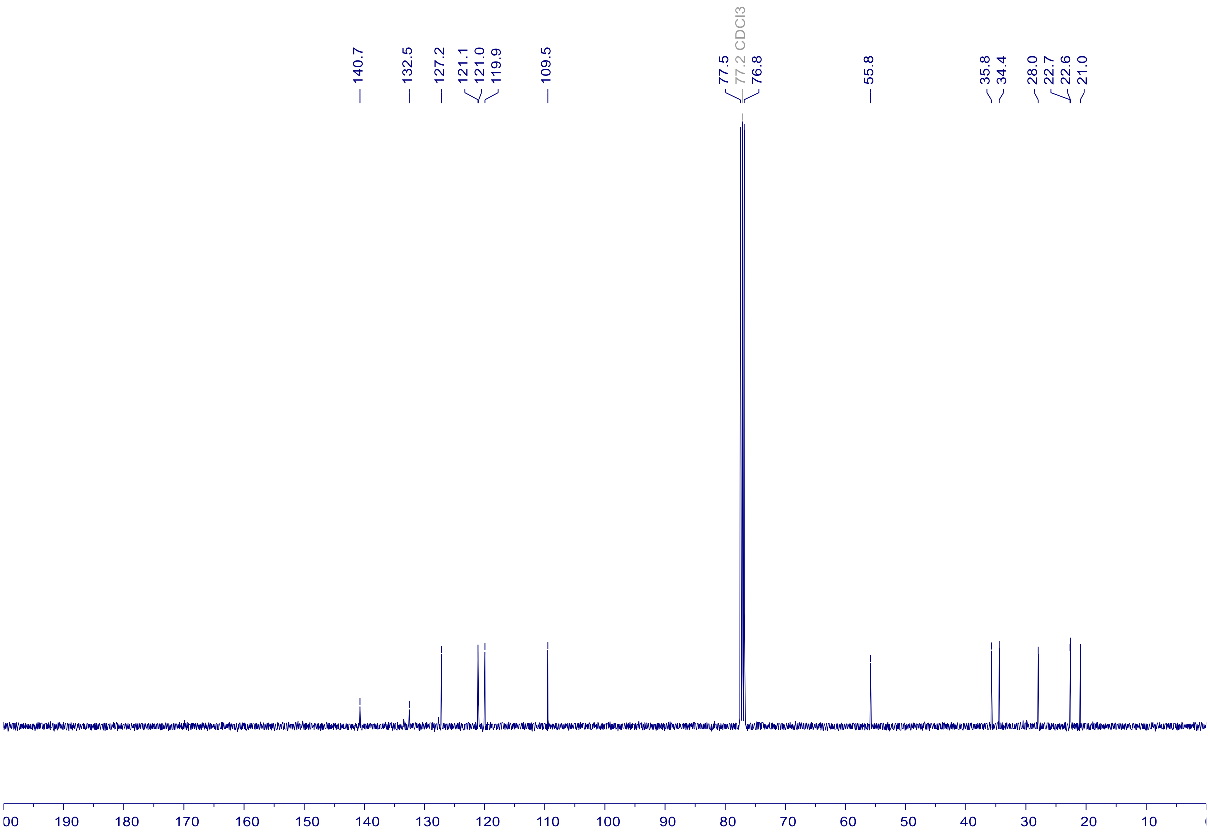

4o – ^{1}H NMR (400 MHz, CDCl$_3$)

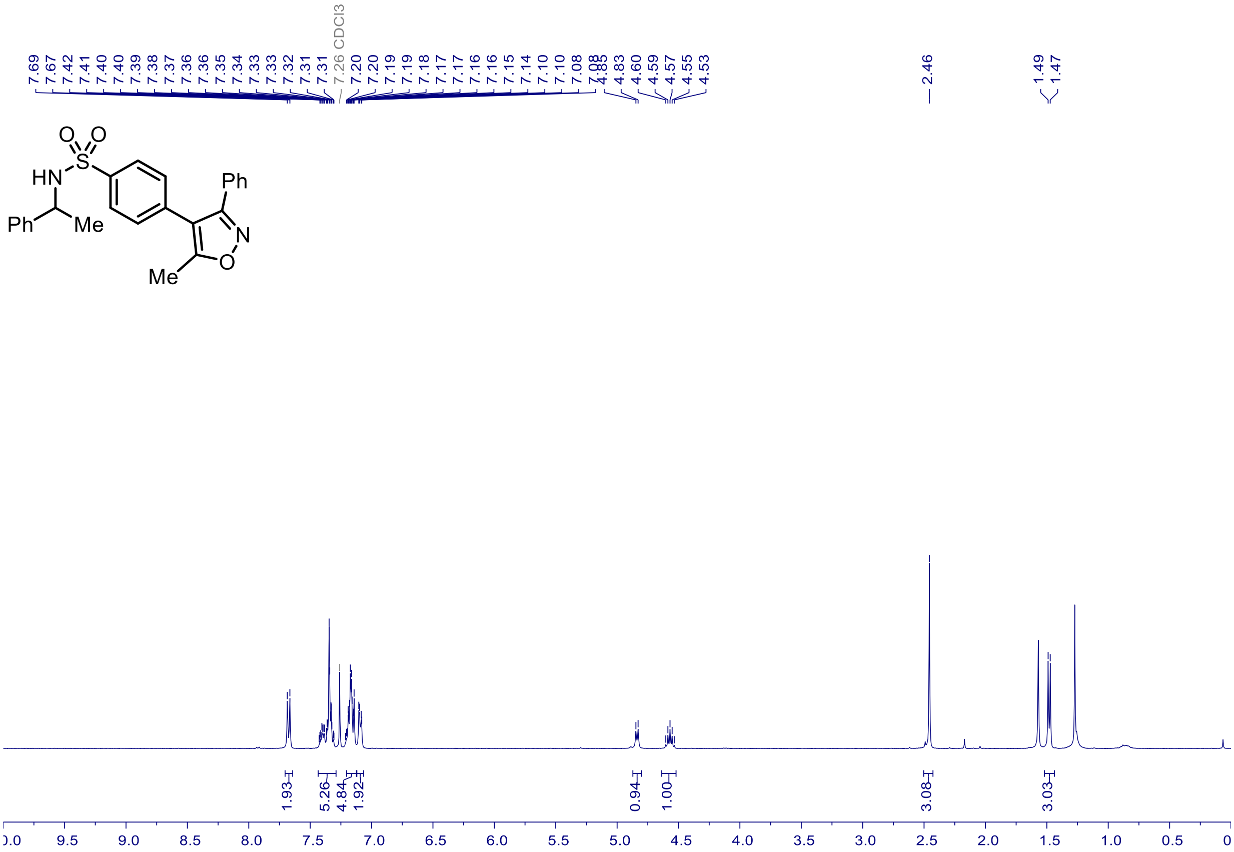

4o – ^{13}C NMR (101 MHz, CDCl$_3$)

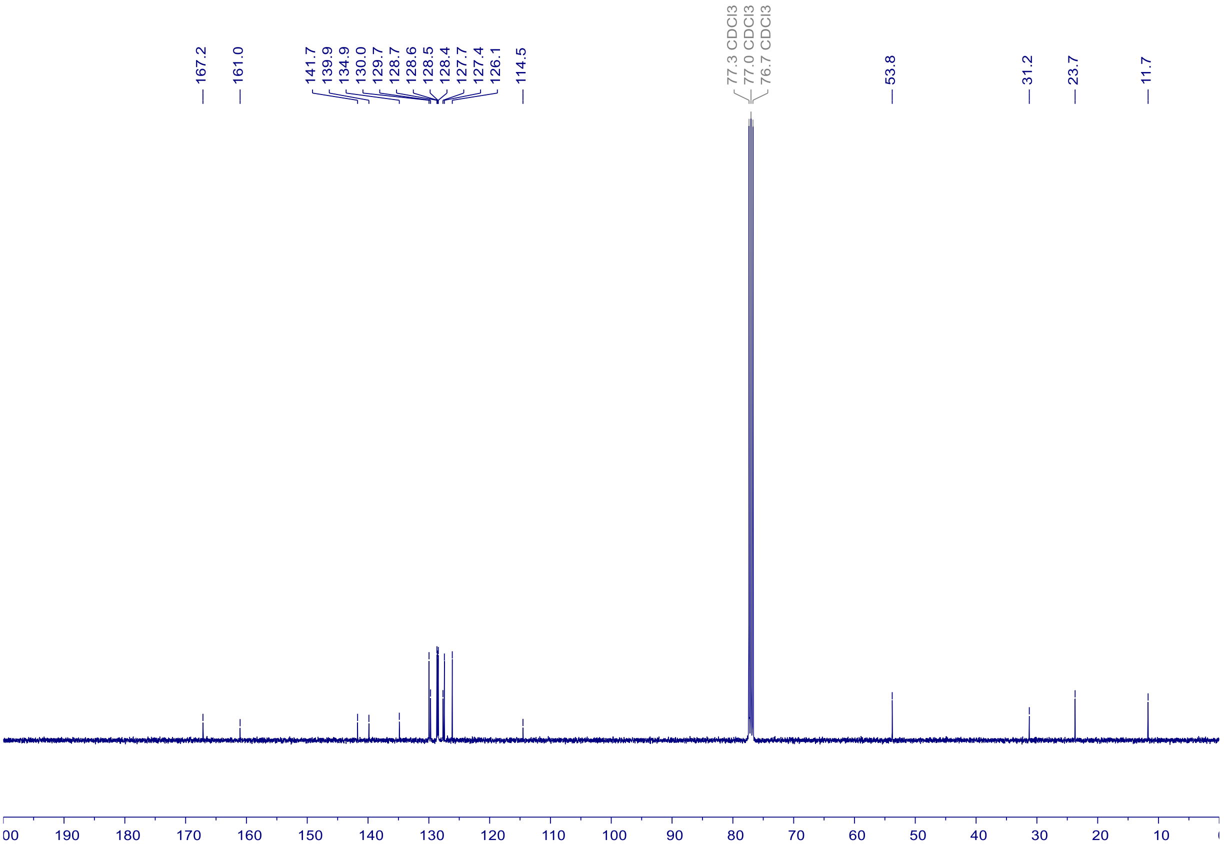

4qa – ^{1}H NMR (400 MHz, $CDCl_3$)

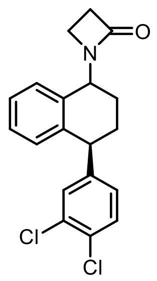

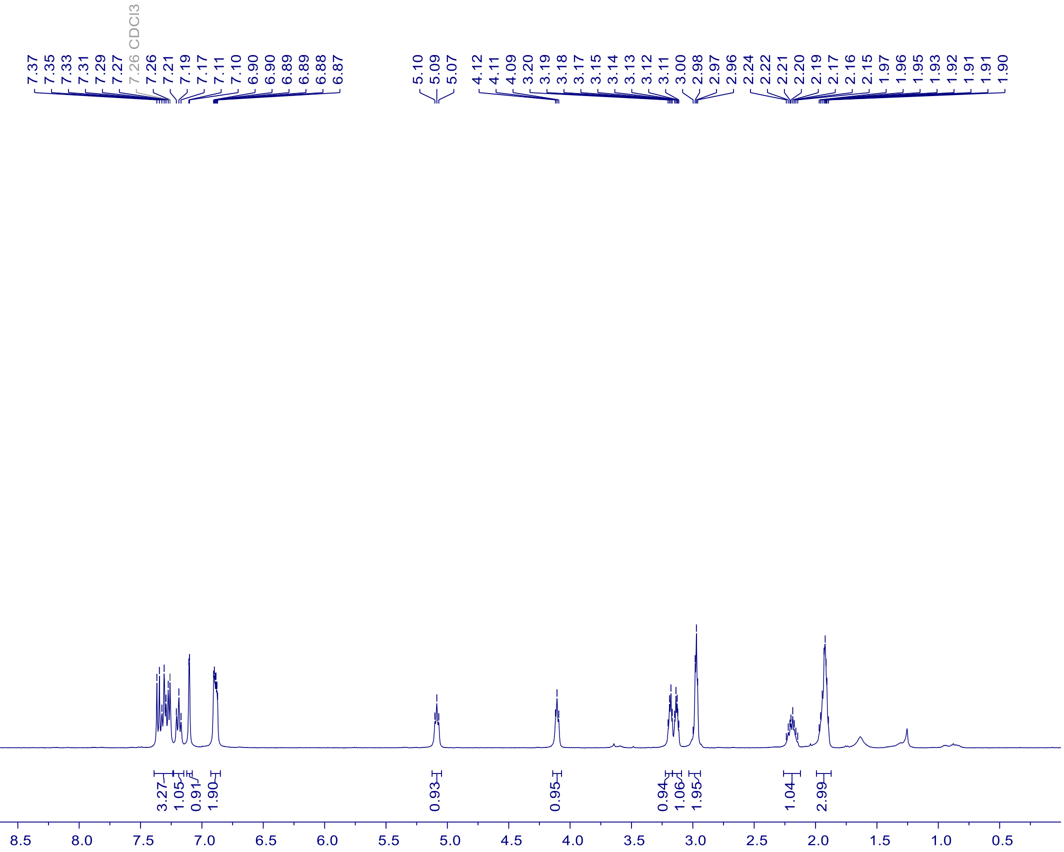

4qa – ^{13}C NMR (101 MHz, $CDCl_3$)

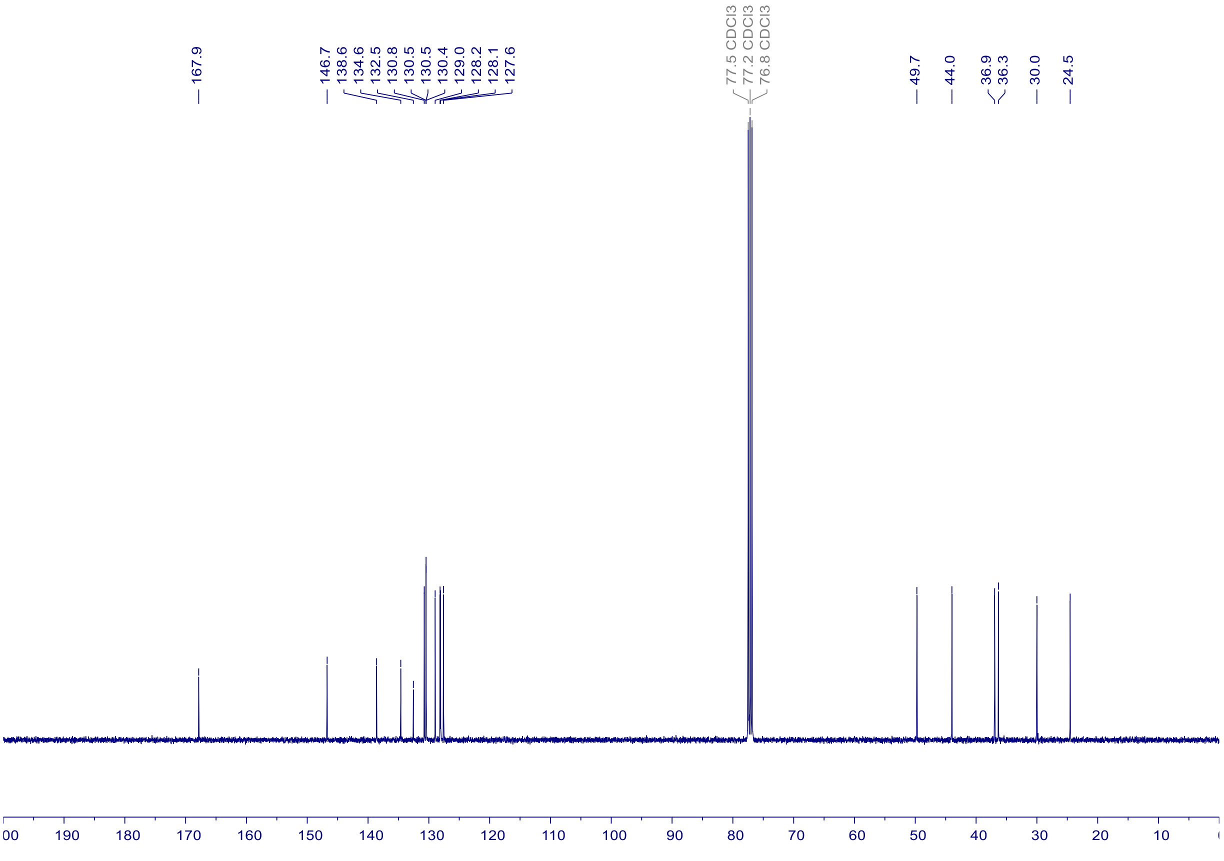

4qb – ^{1}H NMR (400 MHz, $CDCl_3$)

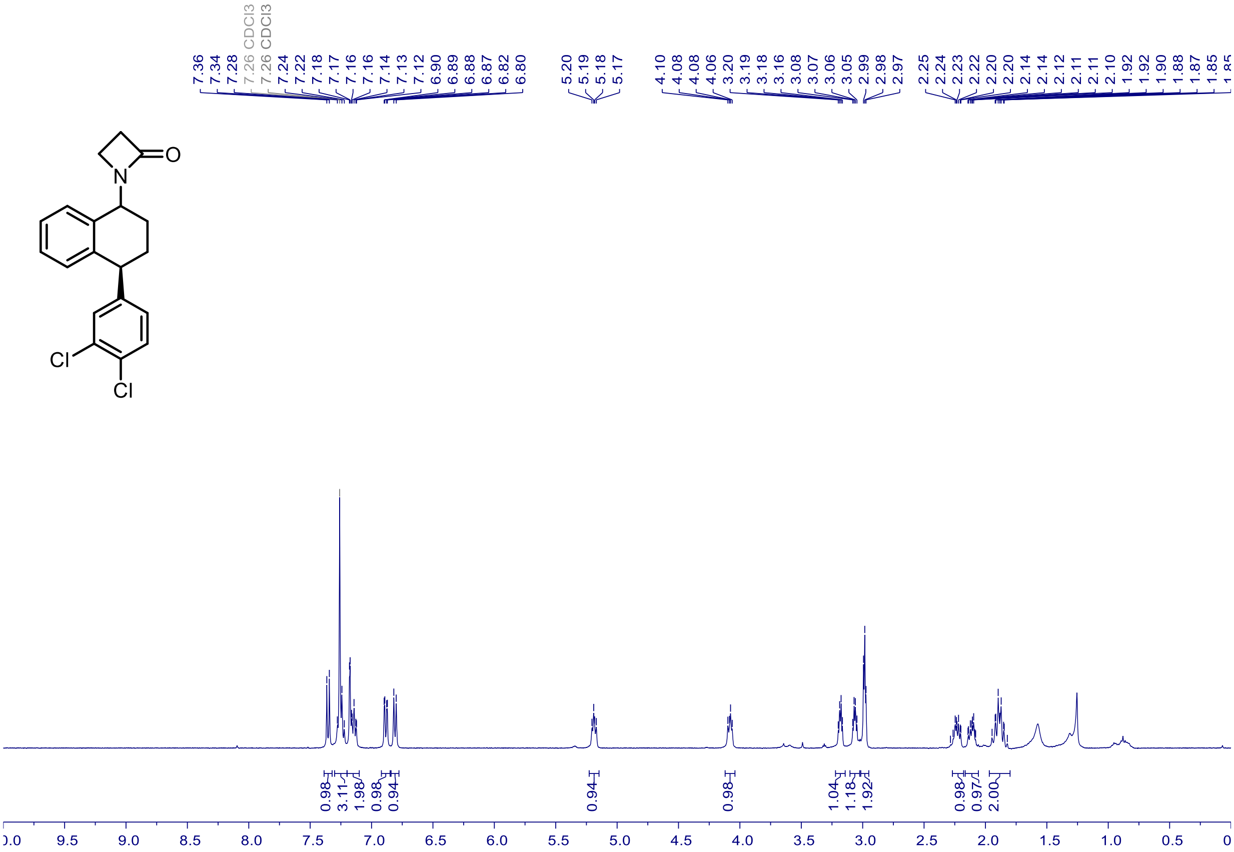

4qb – ^{13}C NMR (101 MHz, $CDCl_3$)

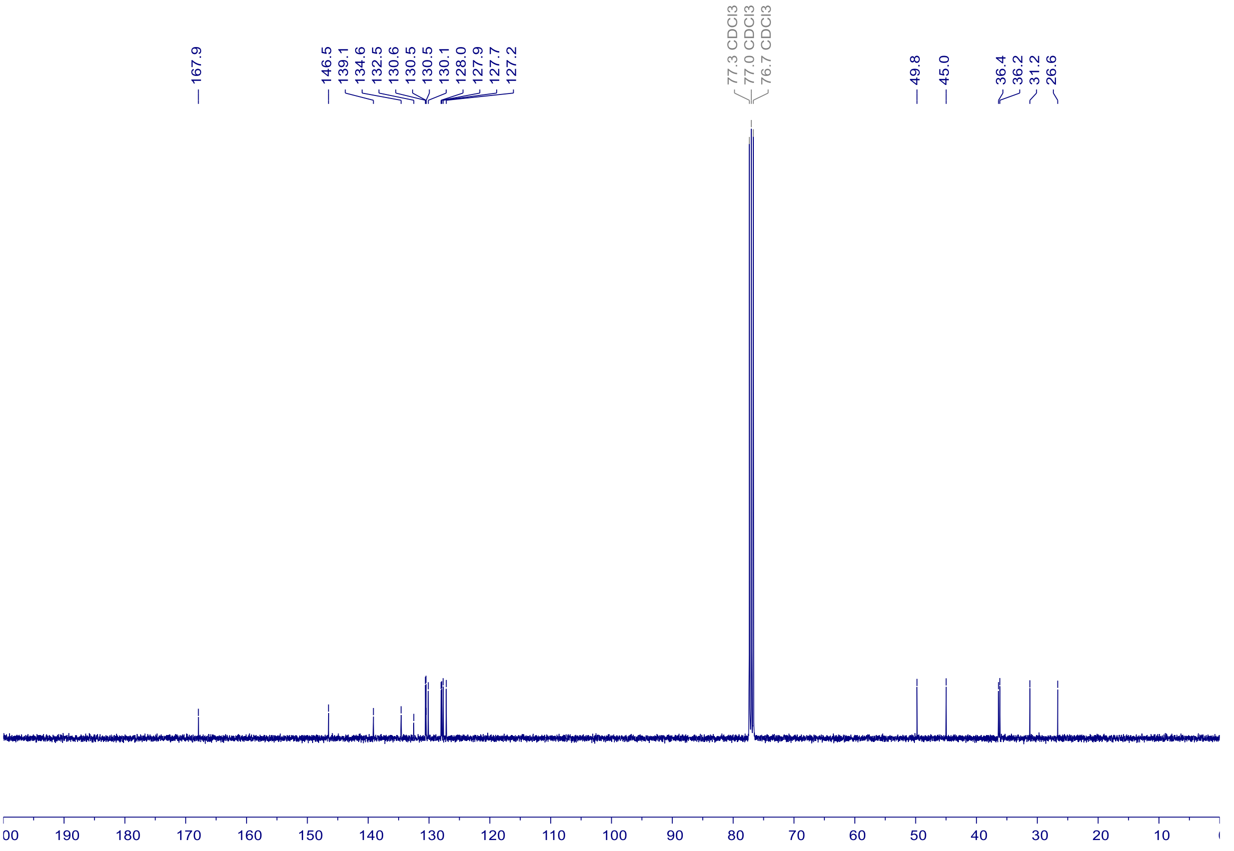

4r – ^{1}H NMR (400 MHz, $CDCl_3$)

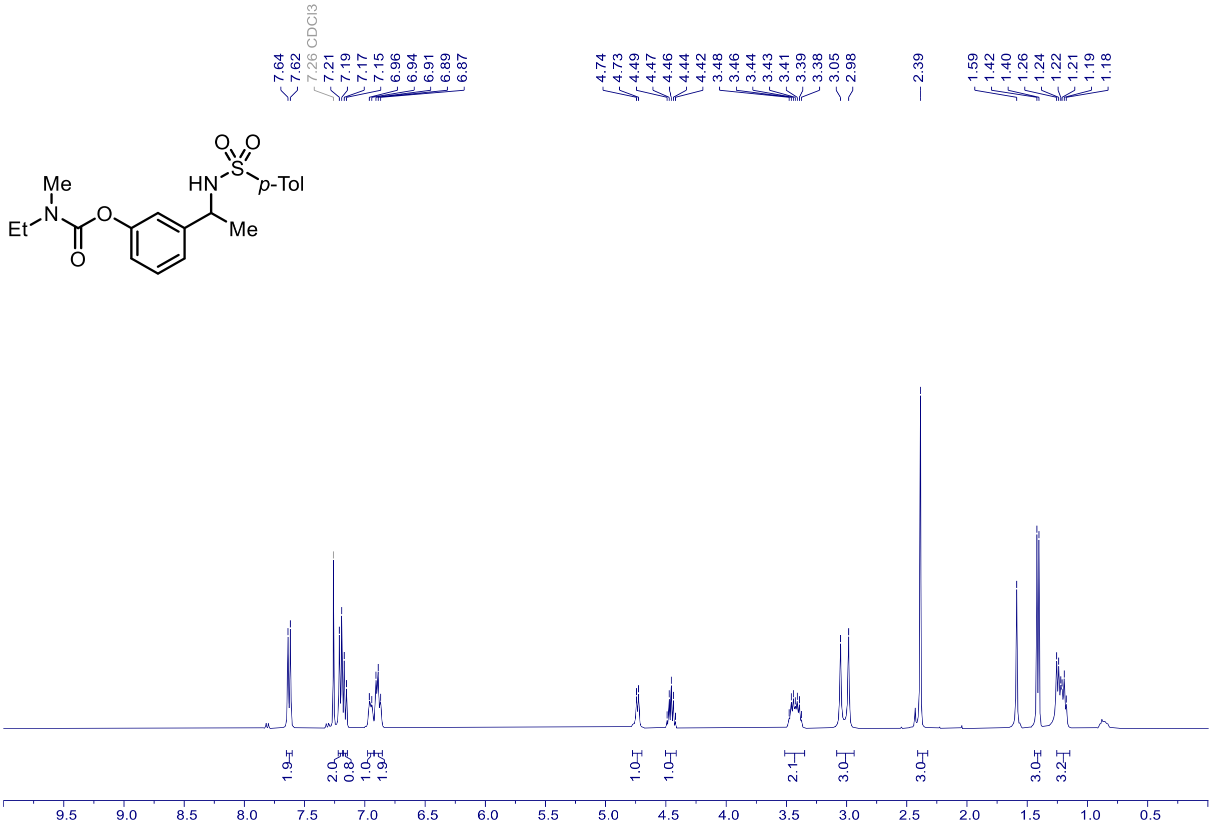

4r – ^{13}C NMR (101 MHz, $CDCl_3$)

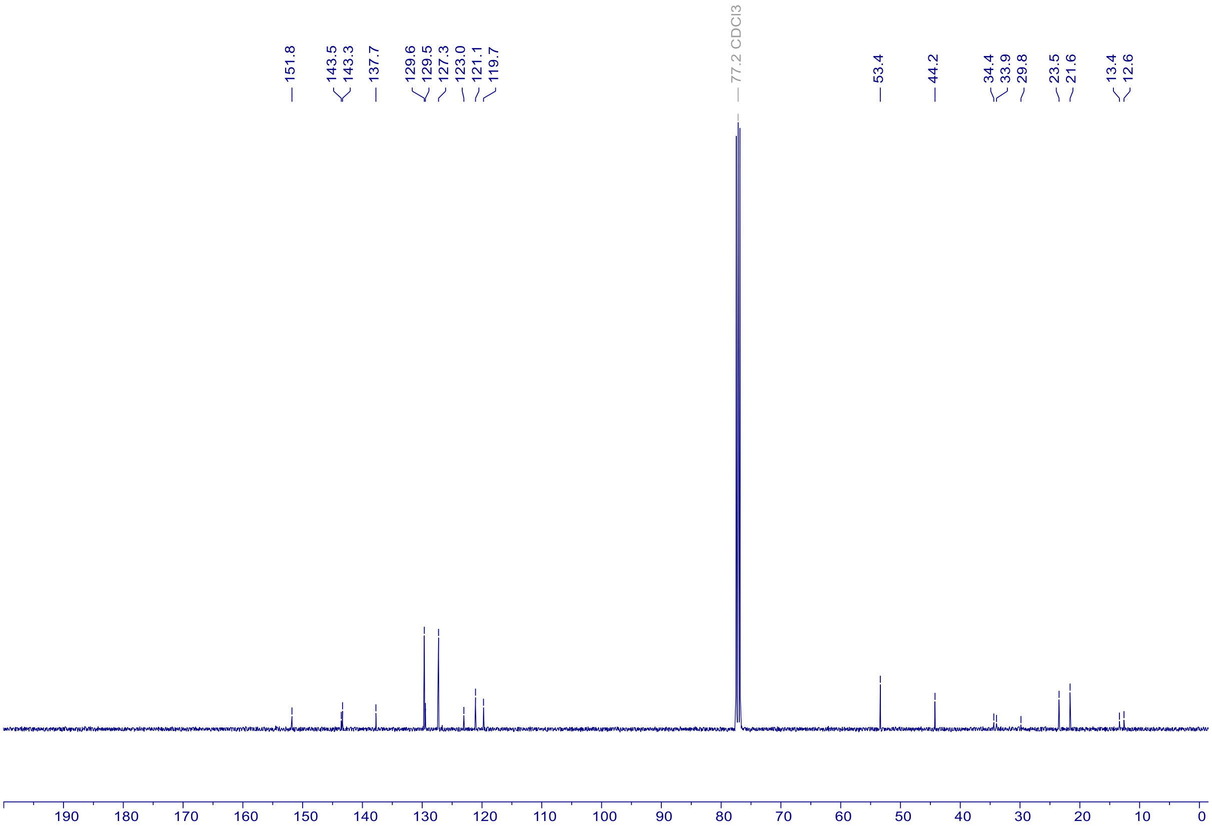

4s – ^{1}H NMR (600 MHz, $CDCl_3$)

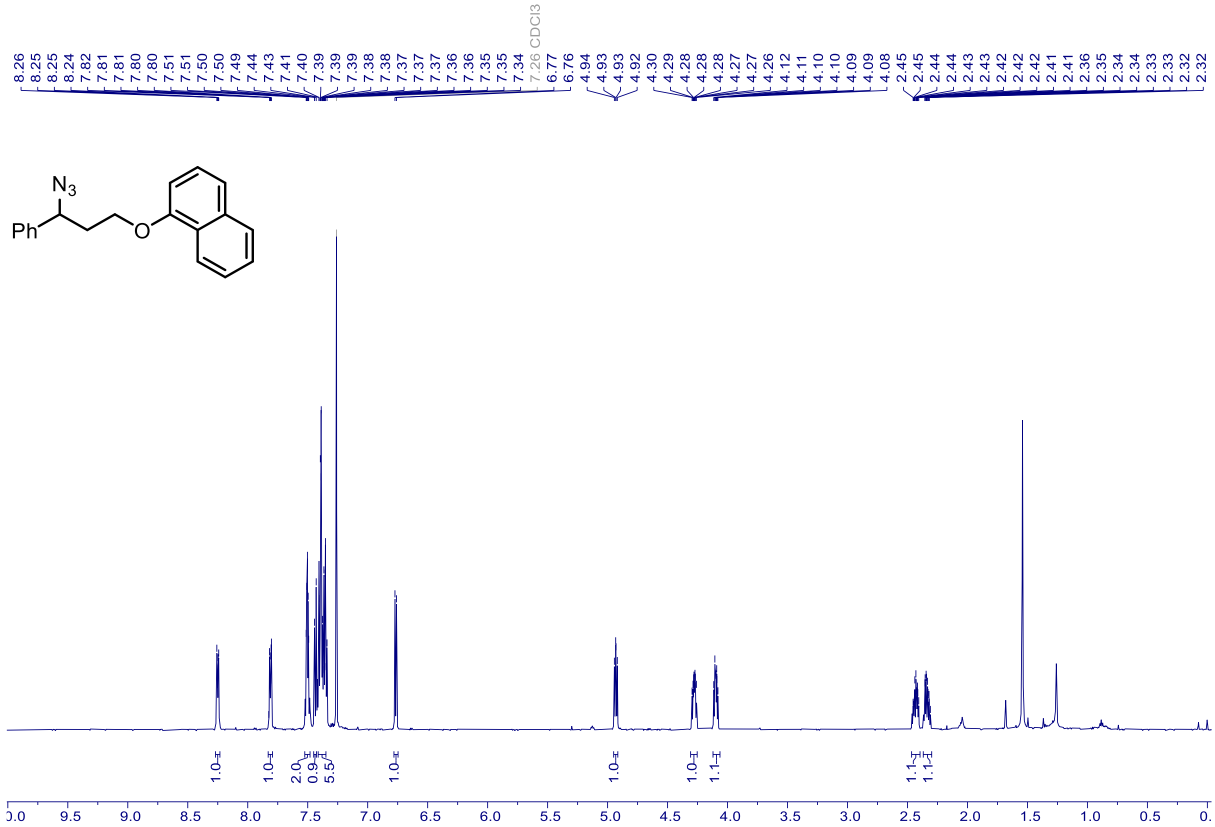

4s – ^{13}C NMR (151 MHz, $CDCl_3$)

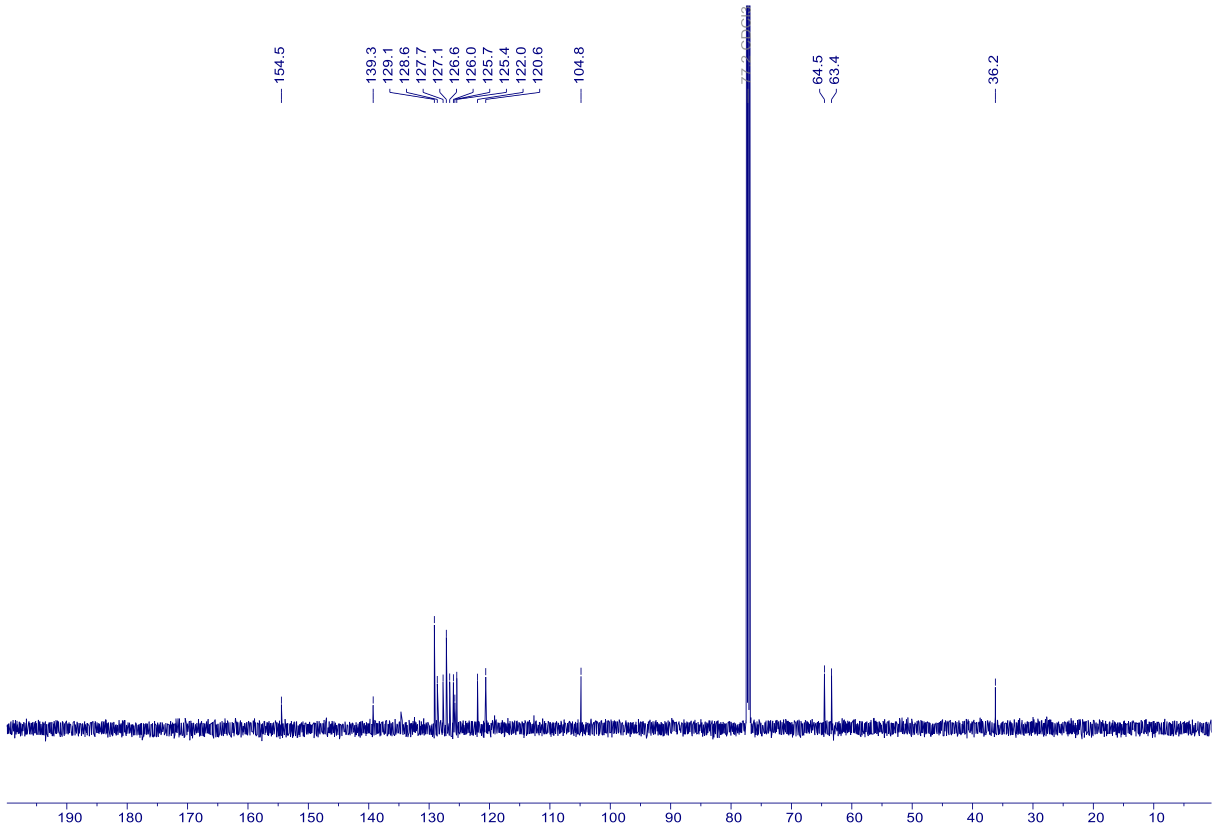

4t – ^{1}H NMR (400 MHz, $CDCl_3$)

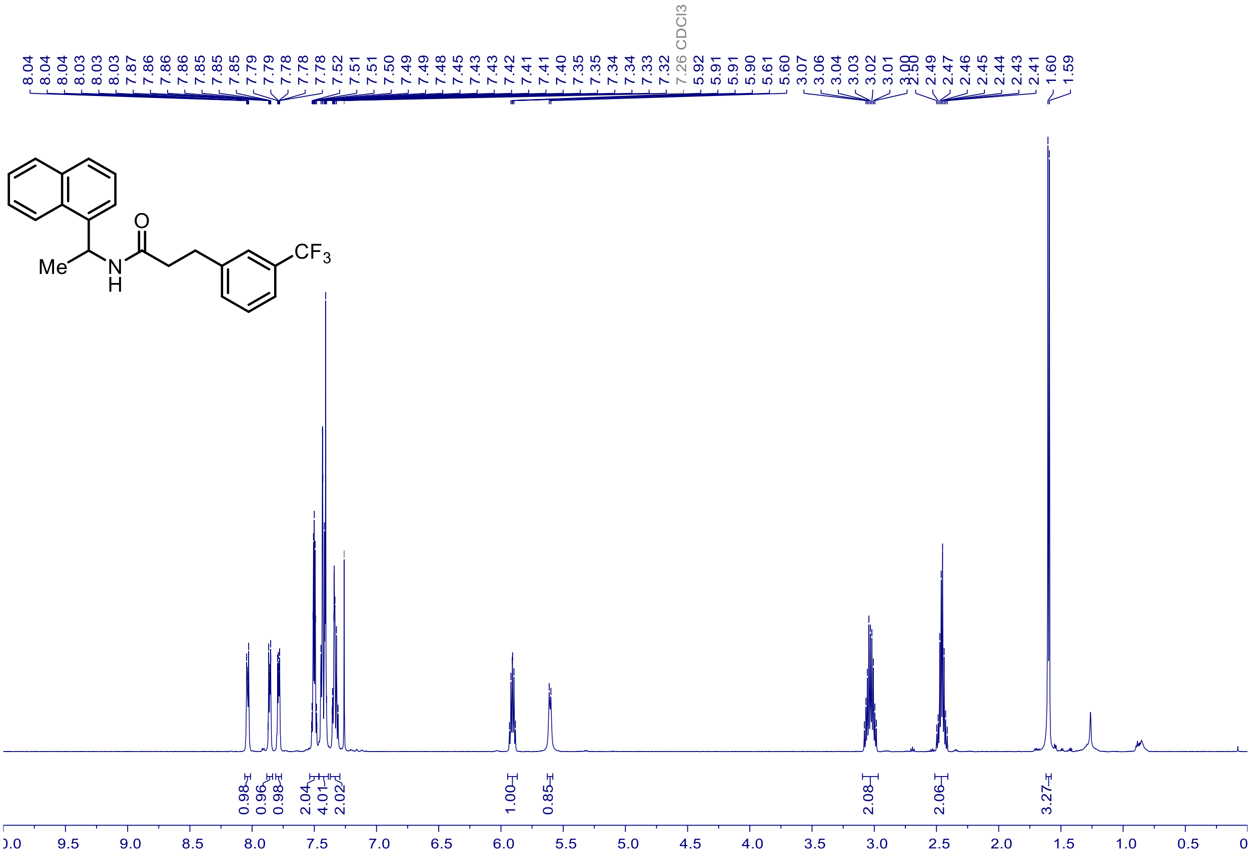

4t – ^{13}C NMR (101 MHz, $CDCl_3$)

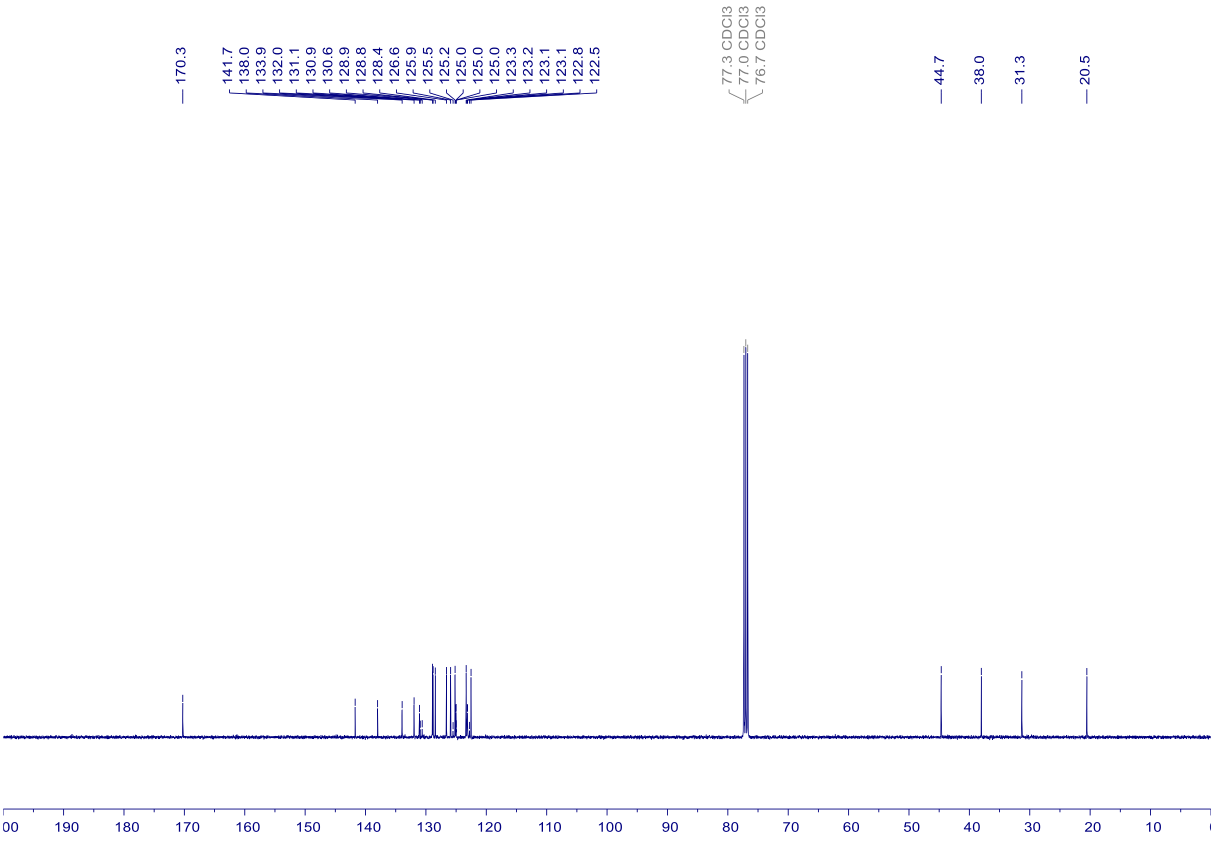

4t – ^{19}F NMR (565 MHz, $CDCl_3$)

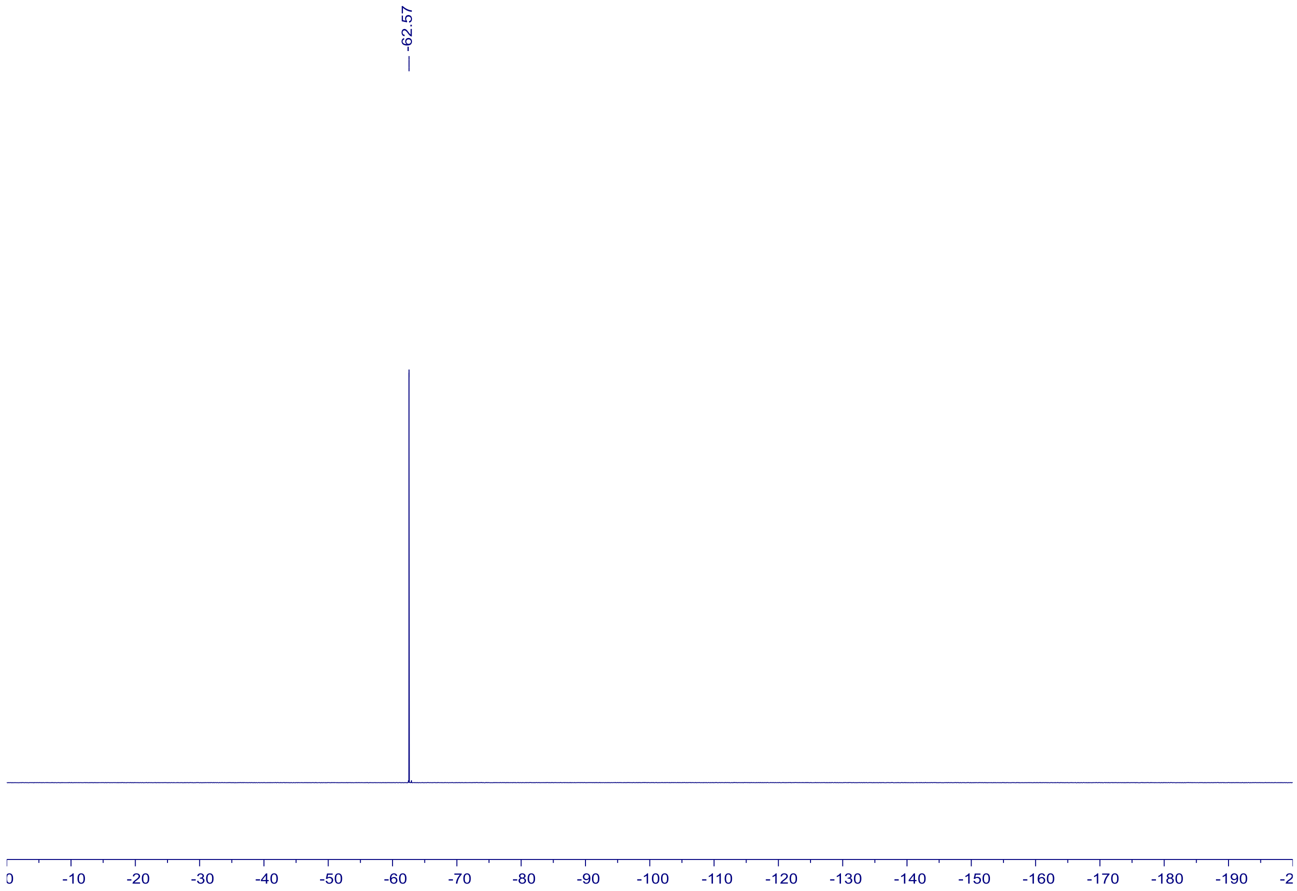

4v – ^{1}H NMR (400 MHz, $CDCl_3$)

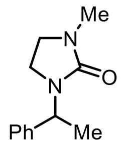

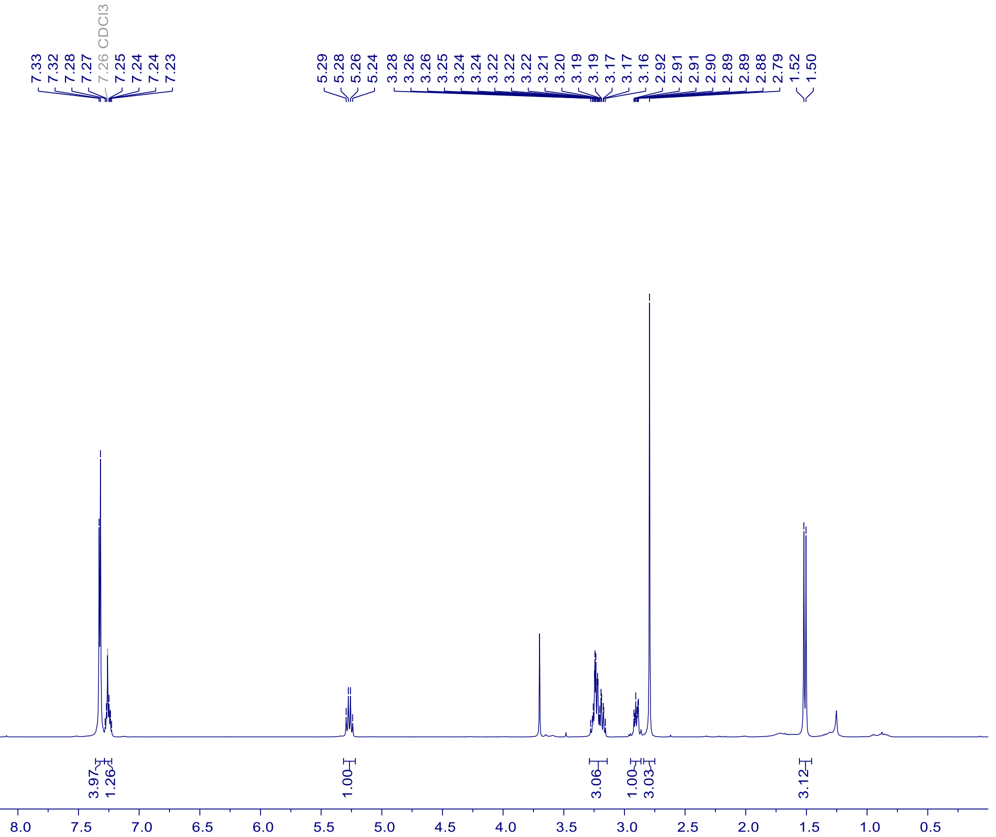

4v – ^{13}C NMR (101 MHz, $CDCl_3$)

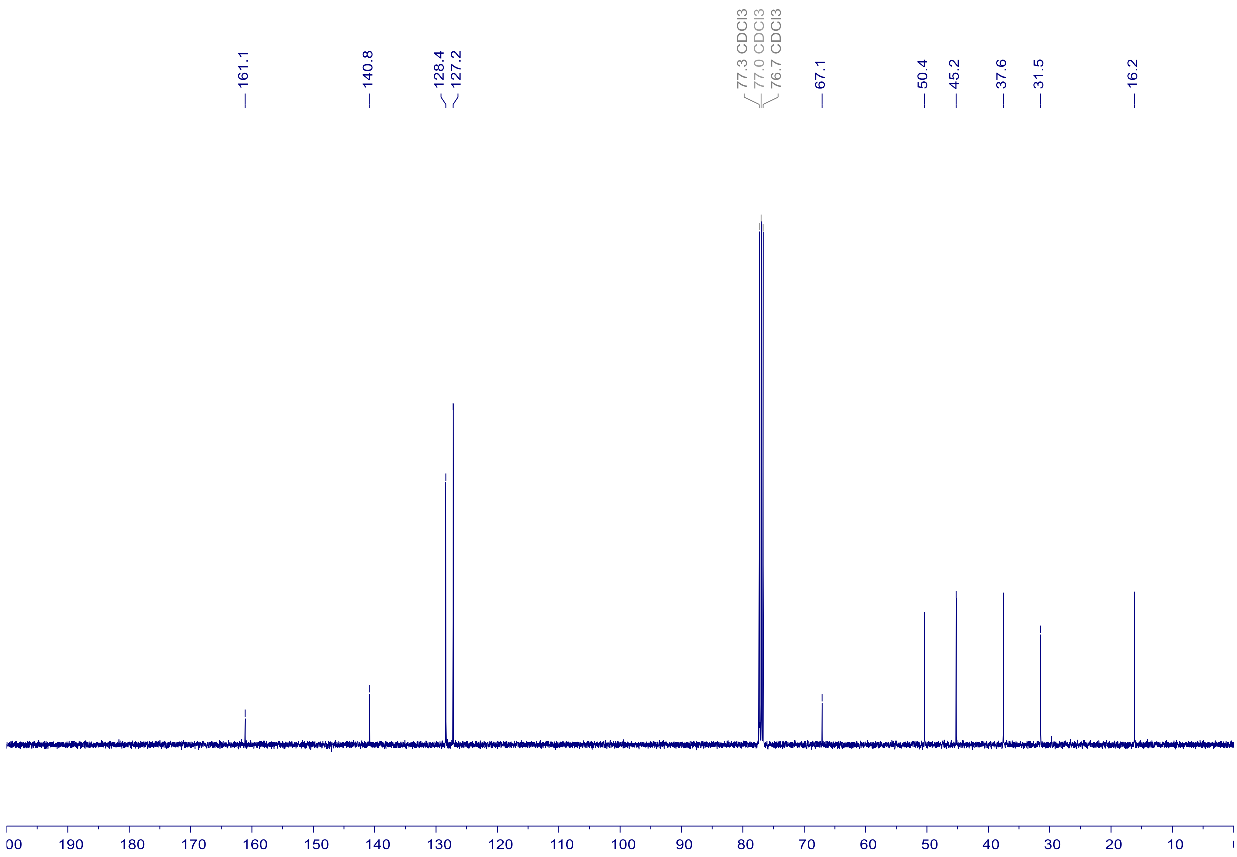

5b – ^{1}H NMR (400 MHz, $CDCl_3$)

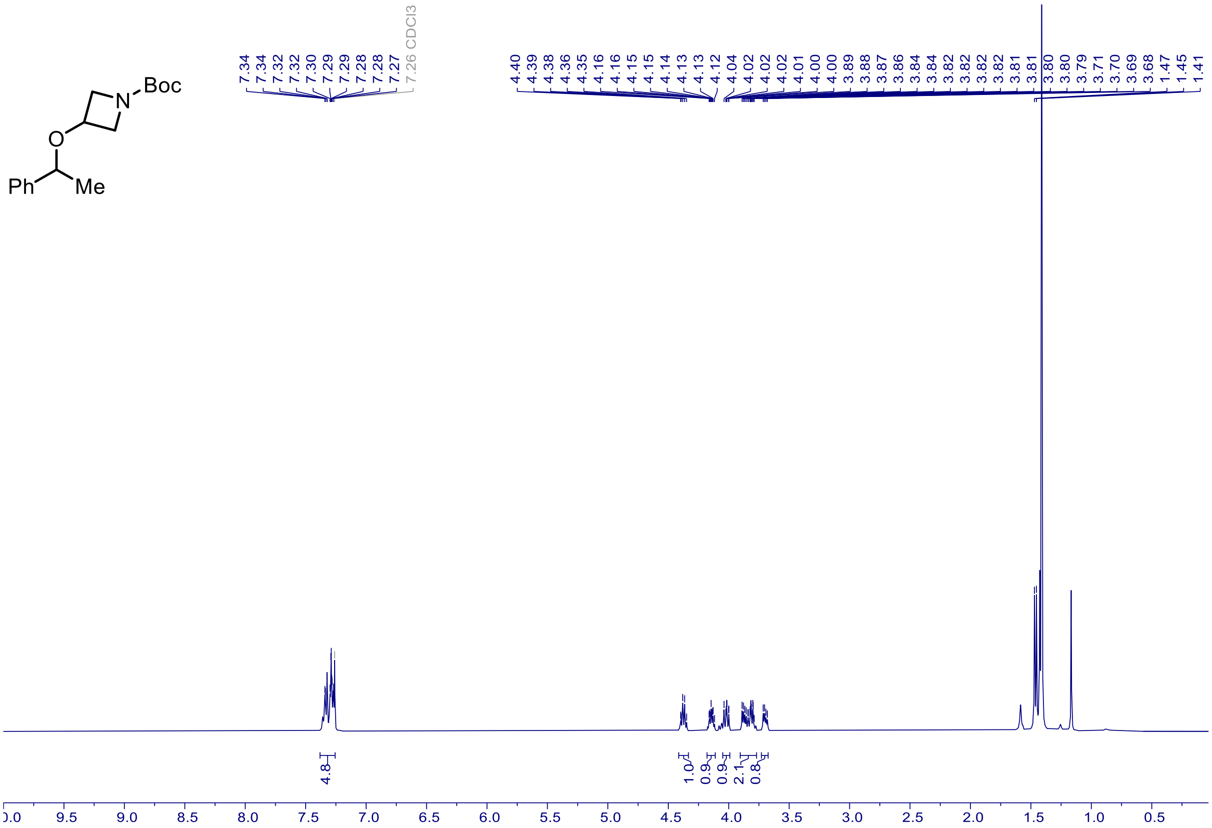

5b – ^{13}C NMR (101 MHz, $CDCl_3$)

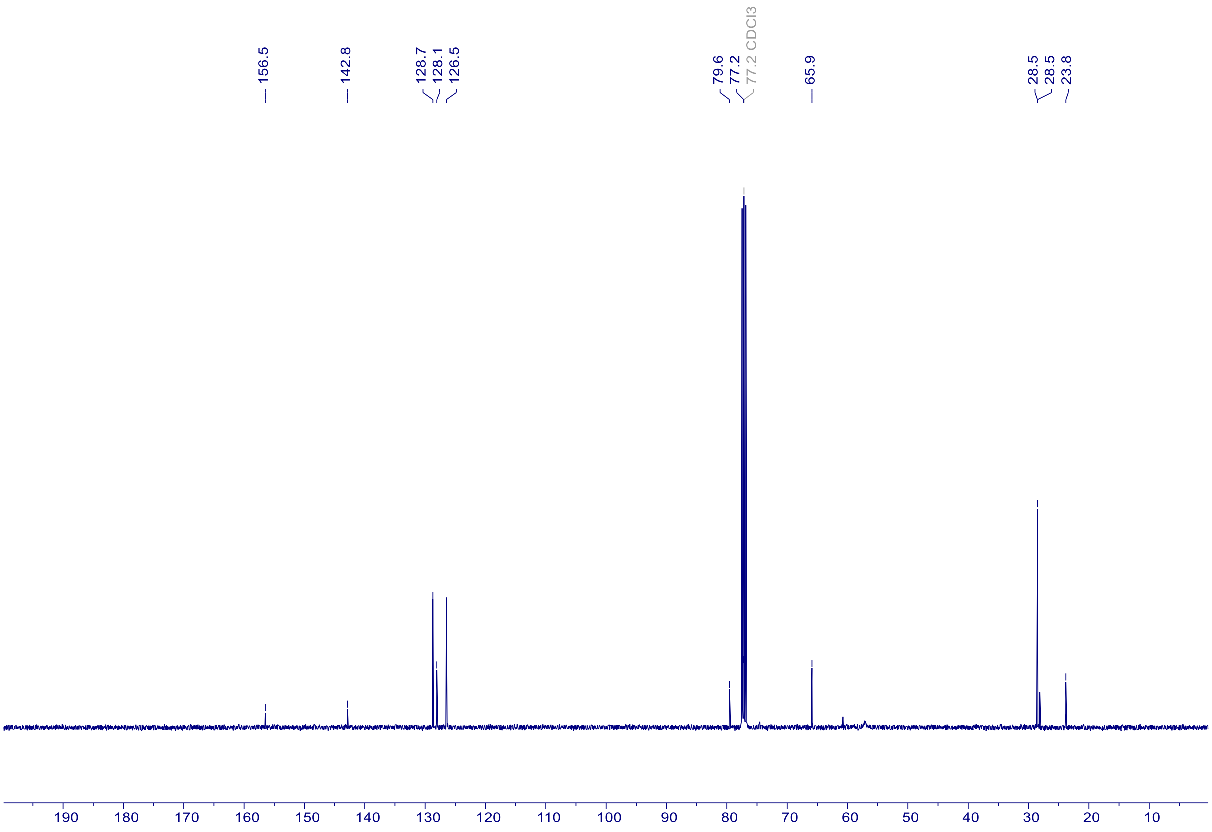

5e – ^{1}H NMR (400 MHz, $CDCl_3$)

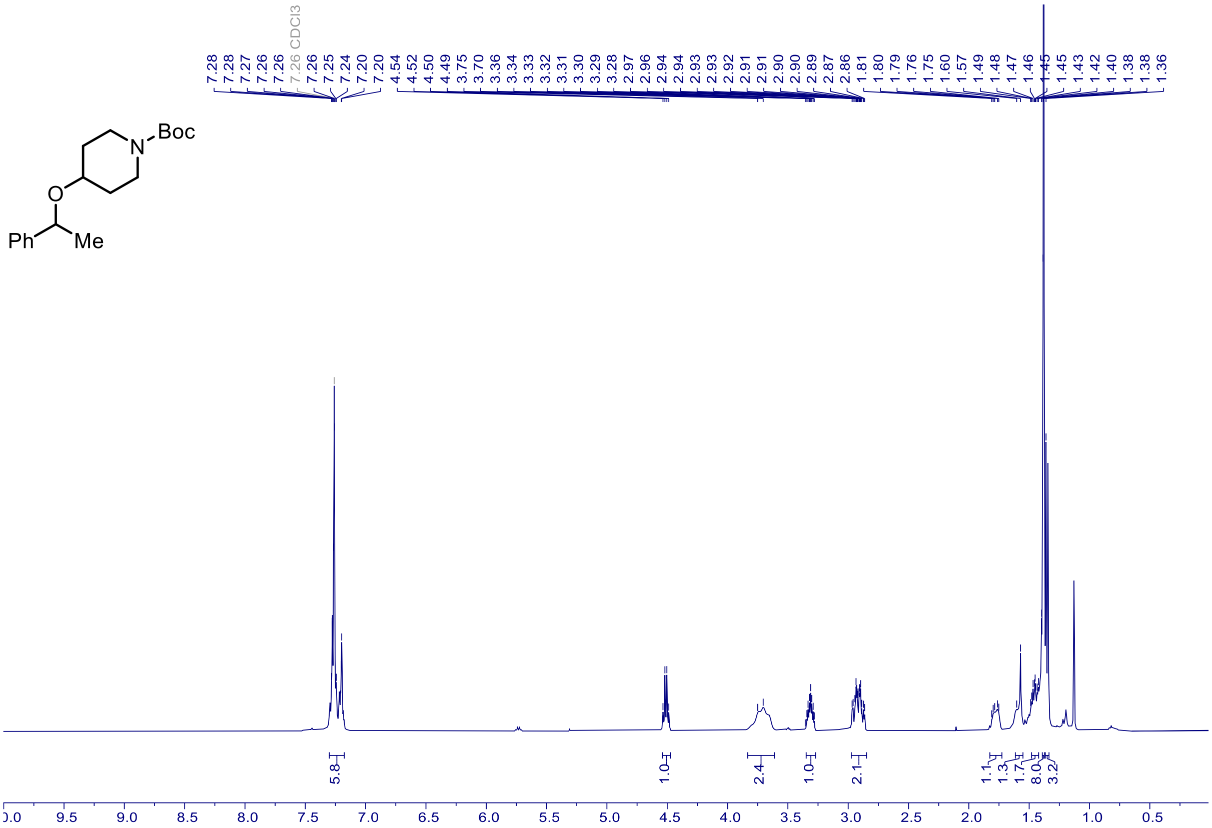

5e – ^{13}C NMR (101 MHz, $CDCl_3$)

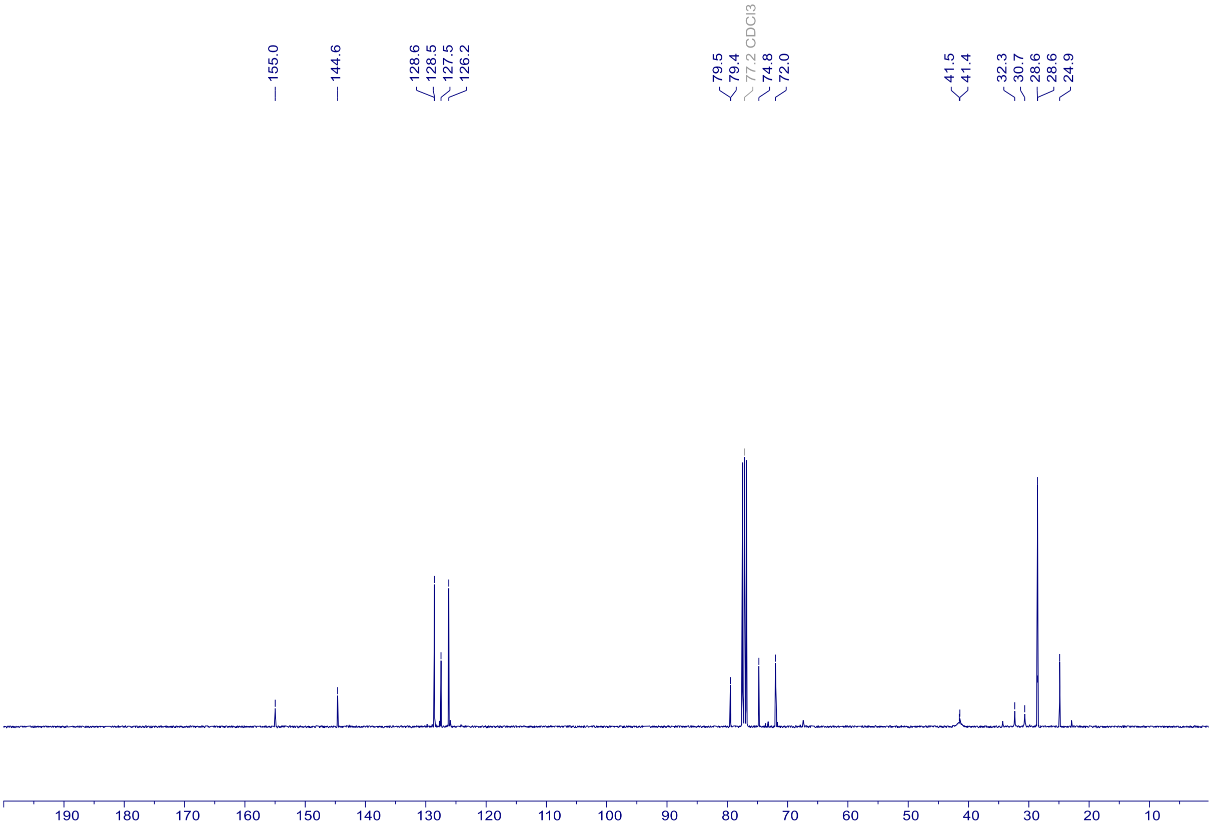

5f – ^{1}H NMR (400 MHz, $CDCl_3$)

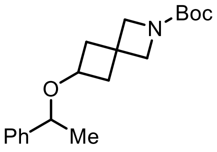

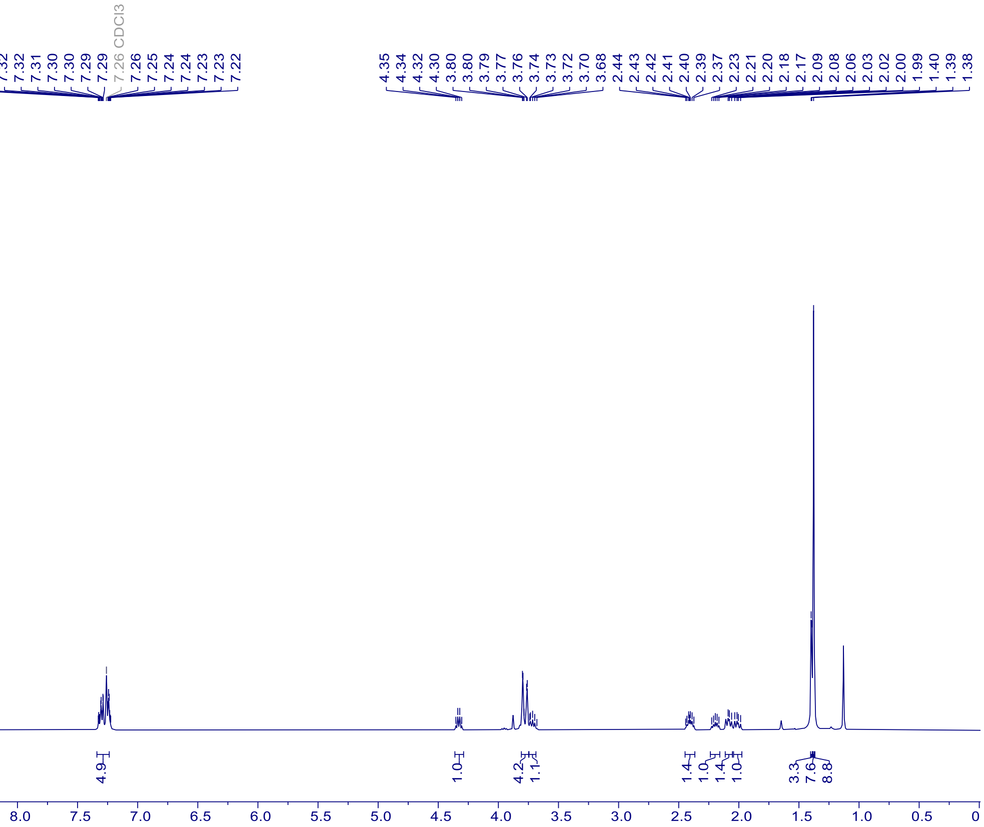

5f – ^{13}C NMR (101 MHz, $CDCl_3$)

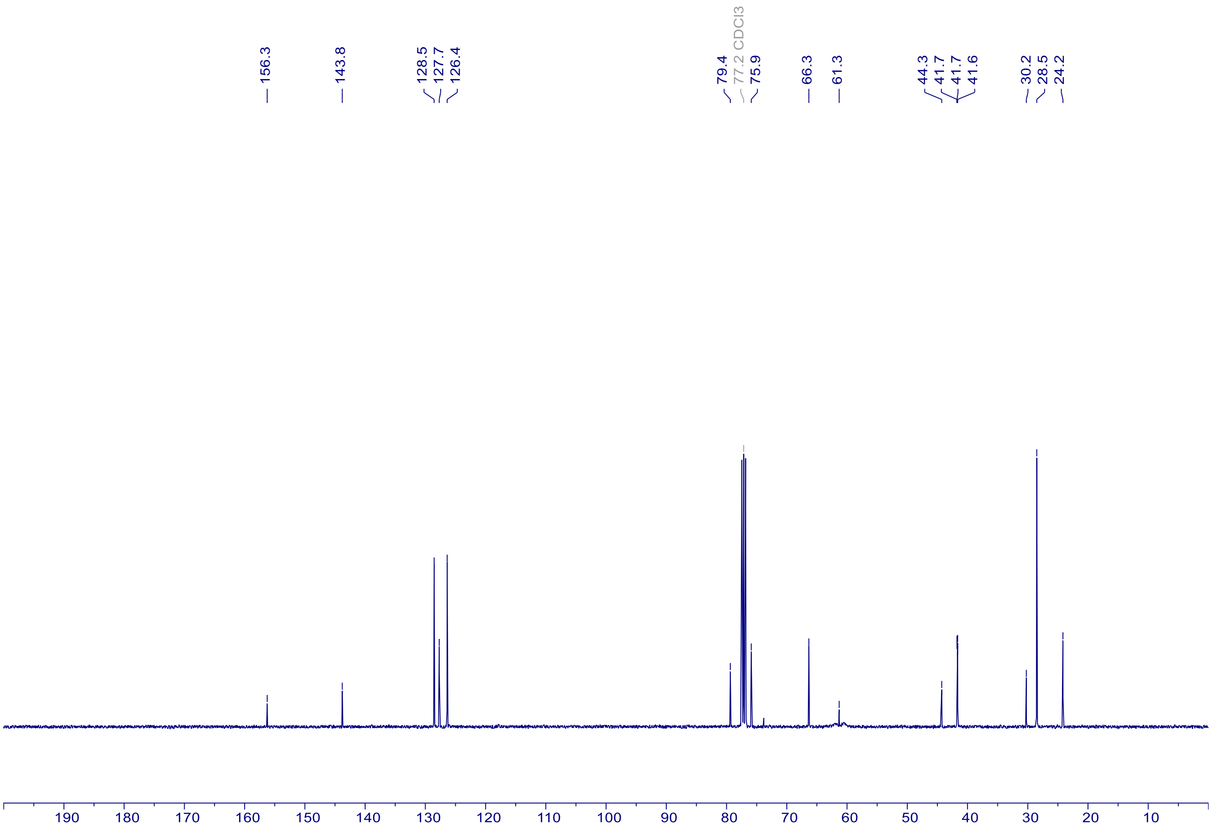

5i – ^{1}H NMR (400 MHz, $CDCl_3$)

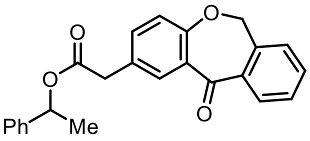

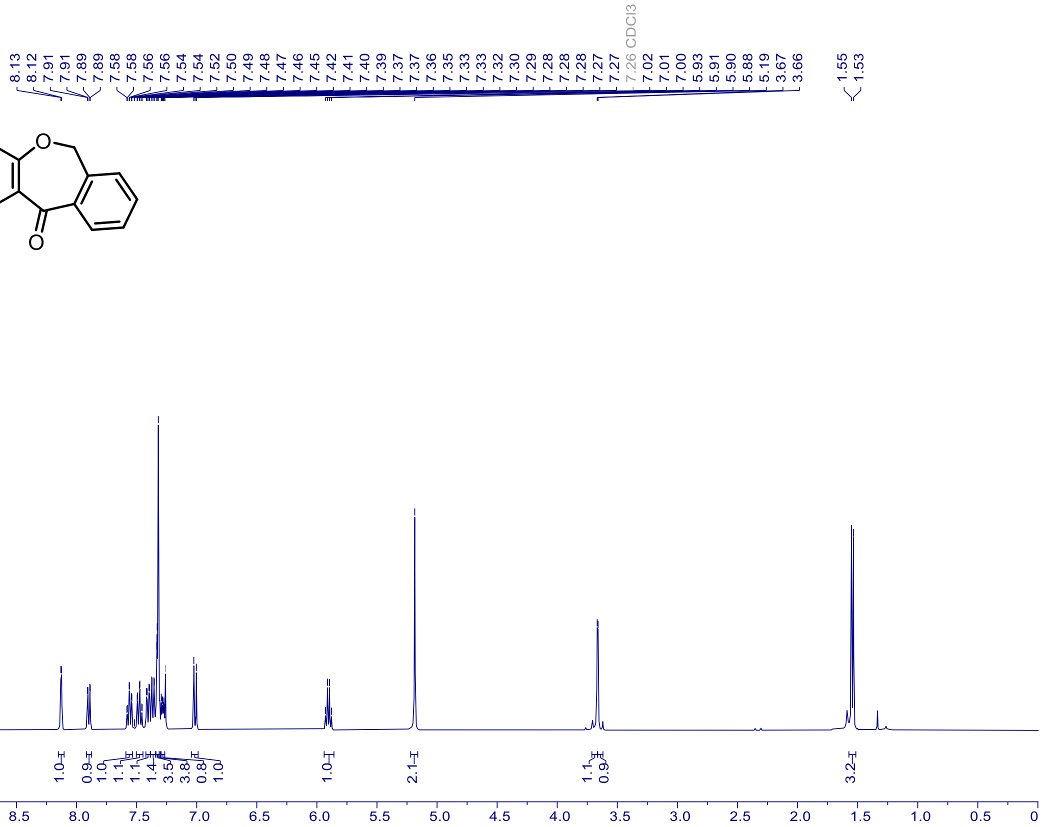

5i – ^{13}C NMR (101 MHz, $CDCl_3$)

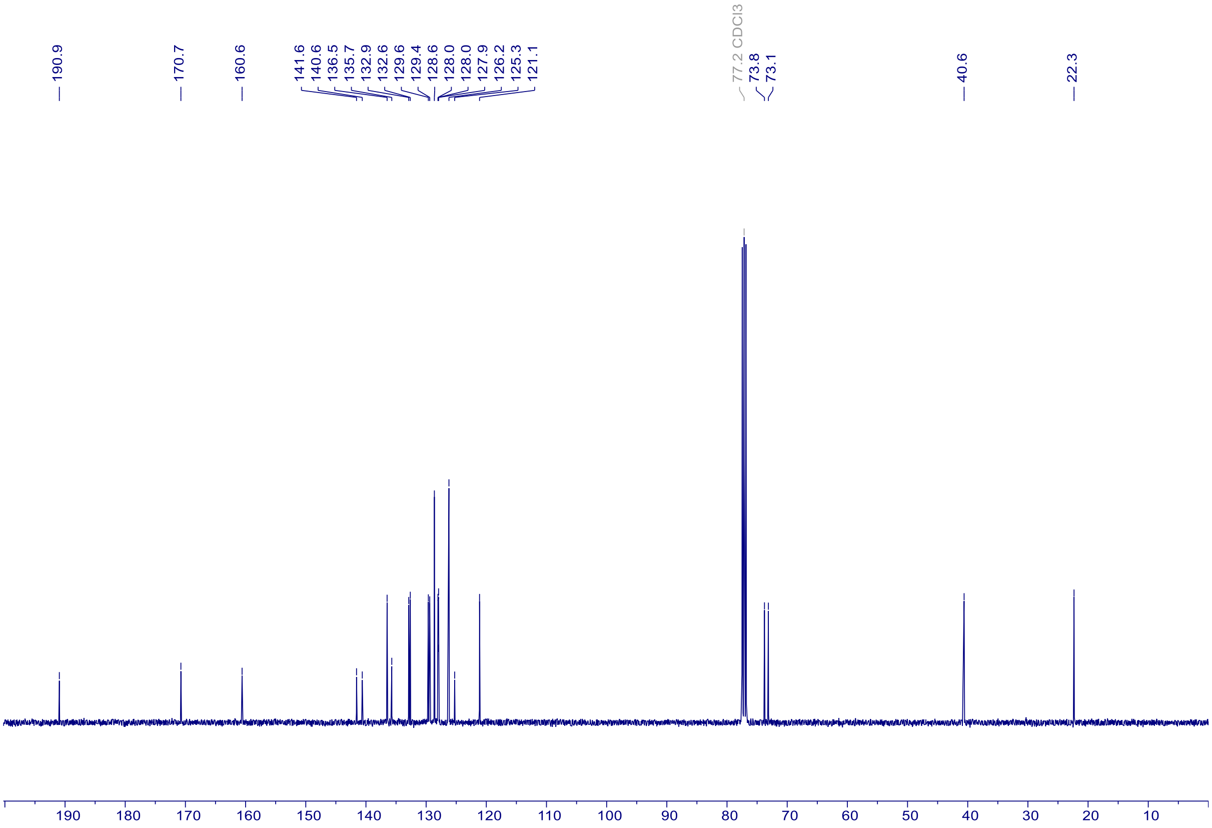

5j – ^{1}H NMR (600 MHz, $CDCl_3$)

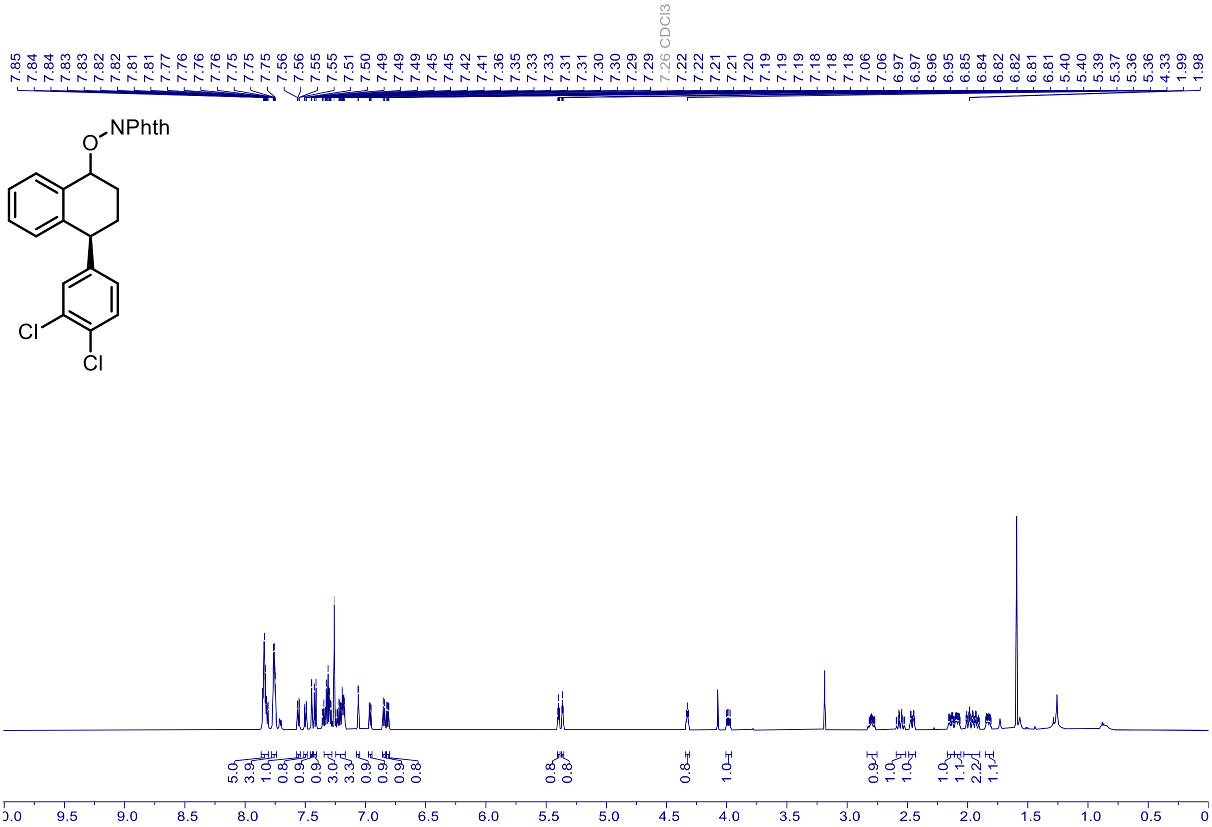

5j – ^{13}C NMR (151 MHz, $CDCl_3$)

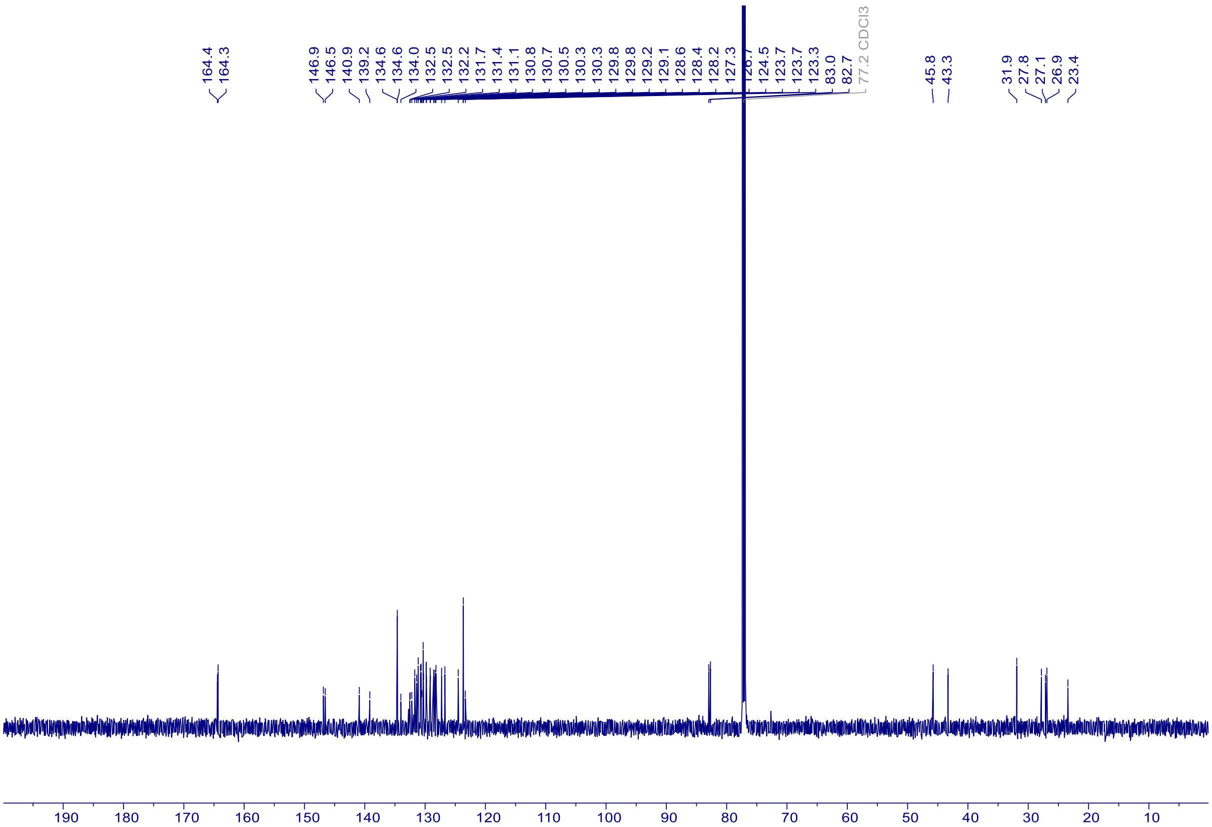

5ka – ^{1}H NMR (400 MHz, $CDCl_3$)

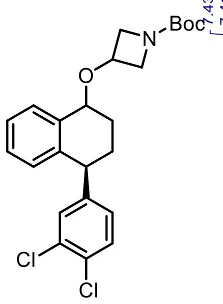

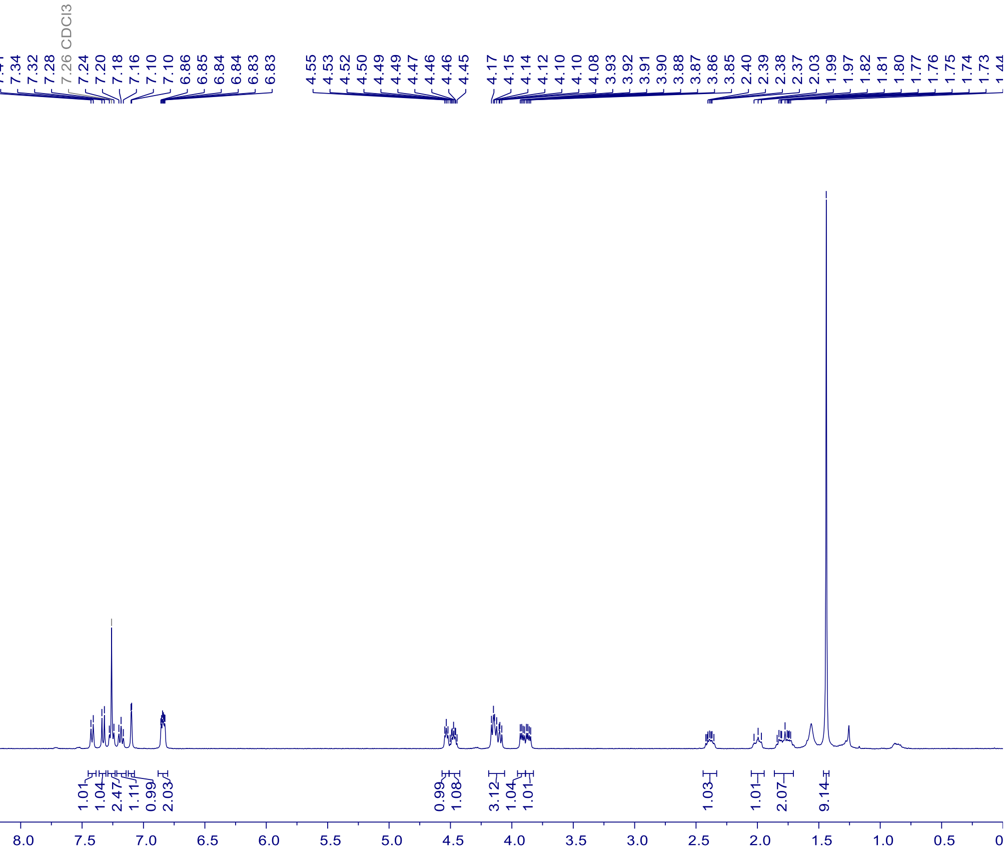

5ka – ^{13}C NMR (101 MHz, $CDCl_3$)

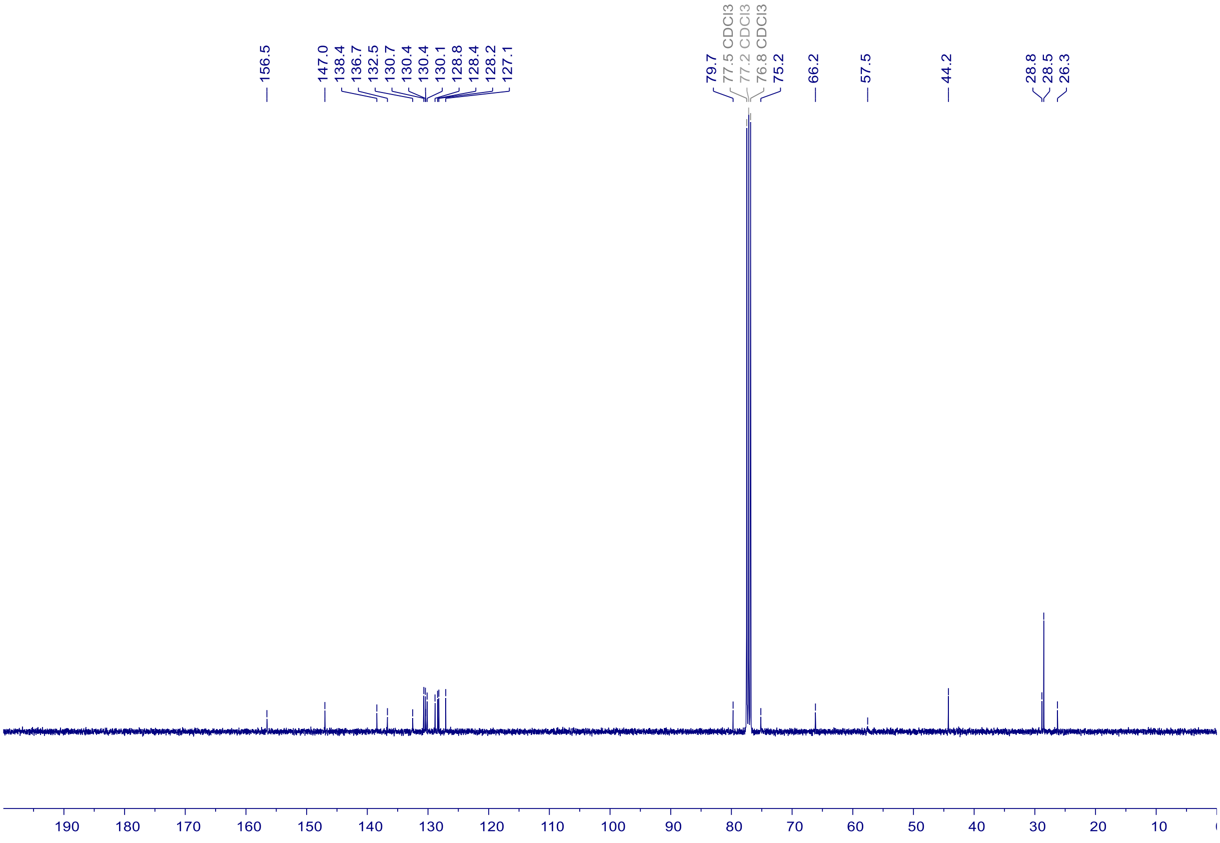

5kb – ^{1}H NMR (400 MHz, $CDCl_3$)

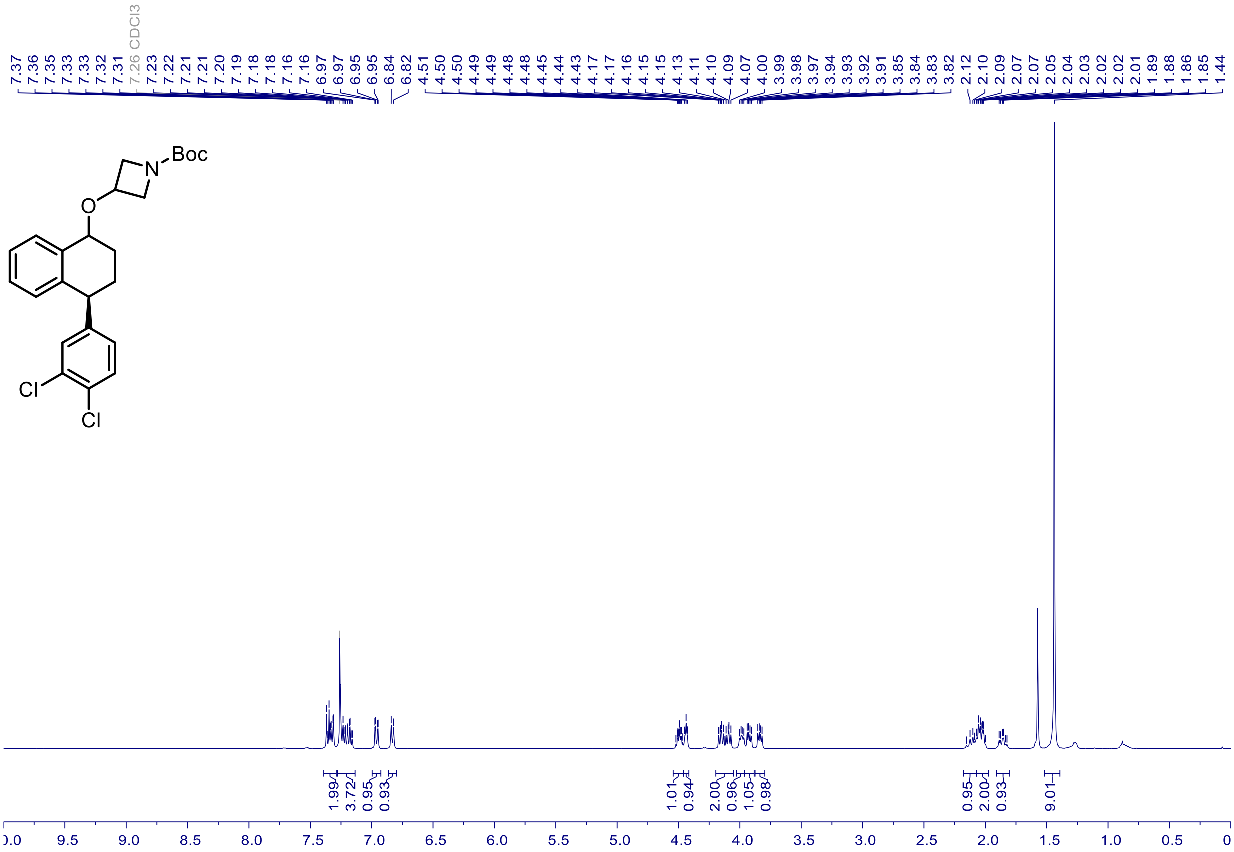

5kb – ^{13}C NMR (101 MHz, $CDCl_3$)

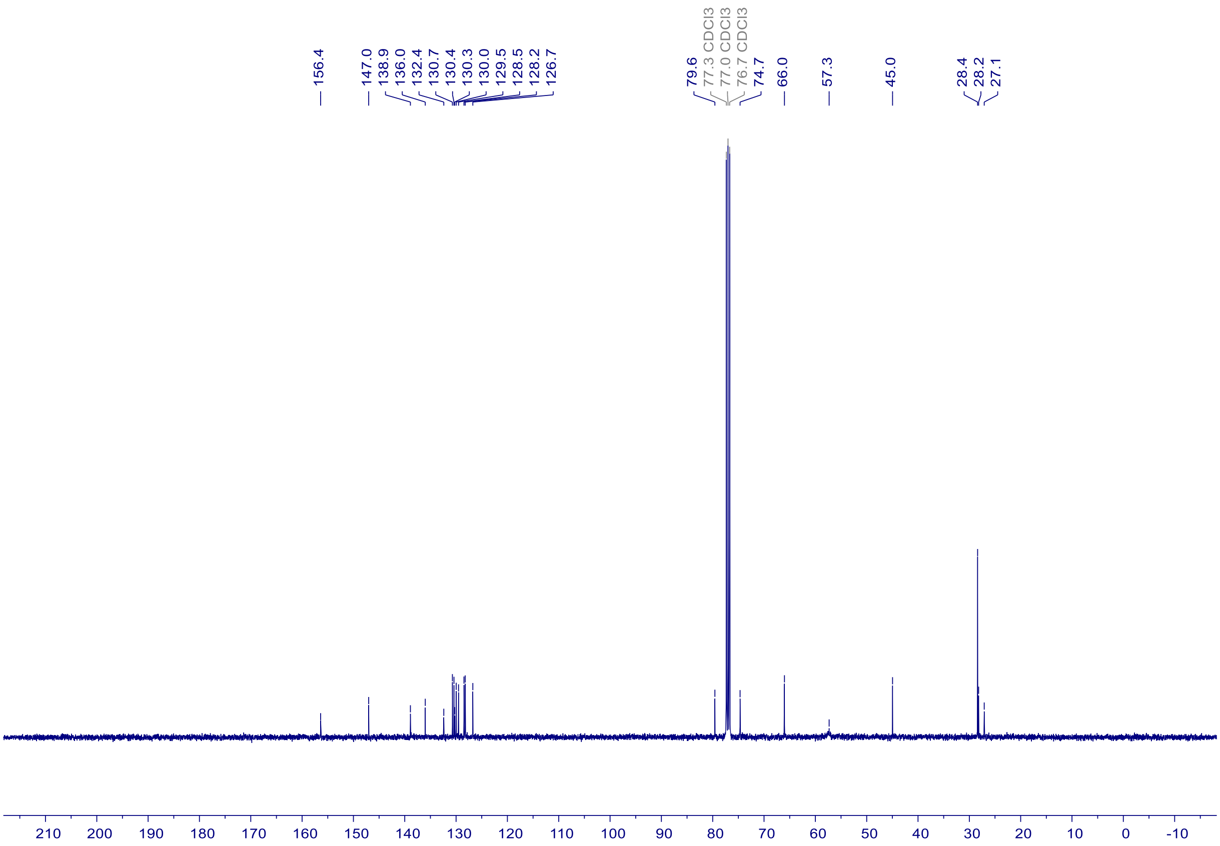

5l –^{1}H NMR (400 MHz, $CDCl_3$)

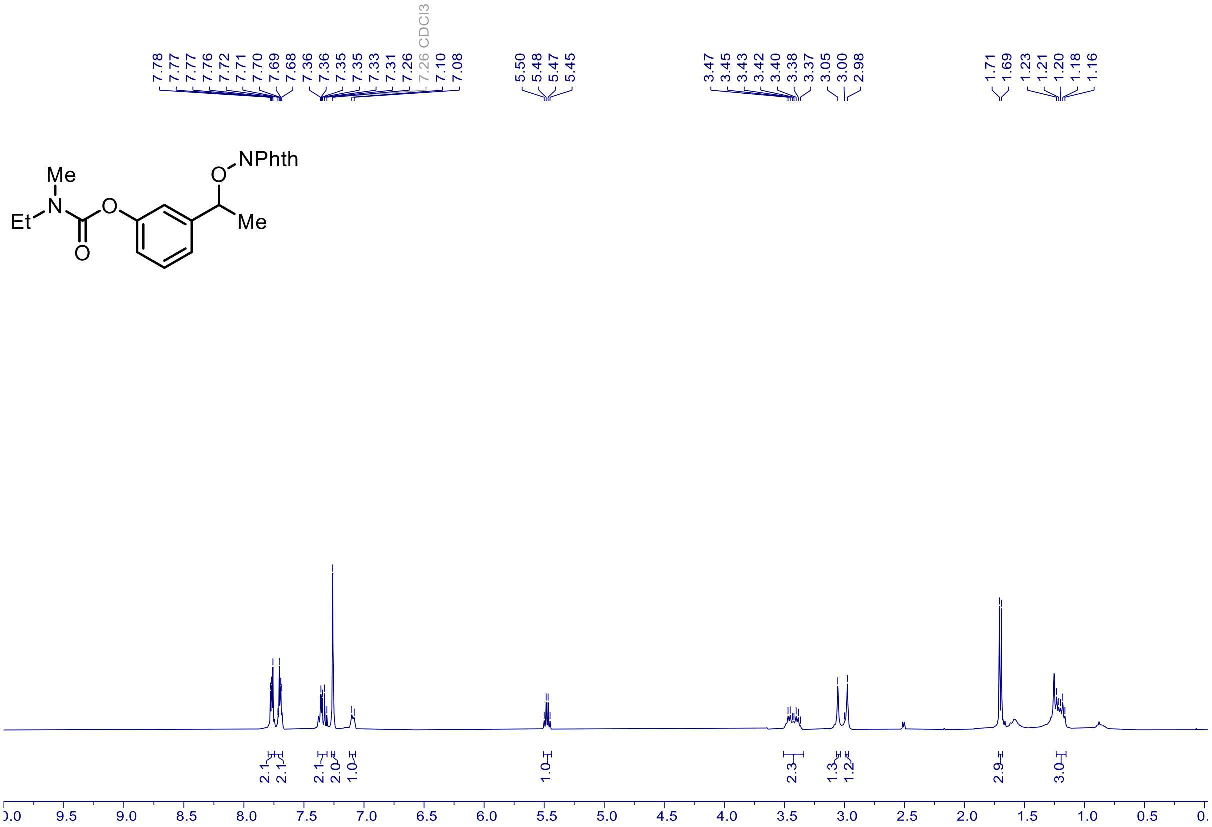

5l – ^{13}C NMR (101 MHz, $CDCl_3$)

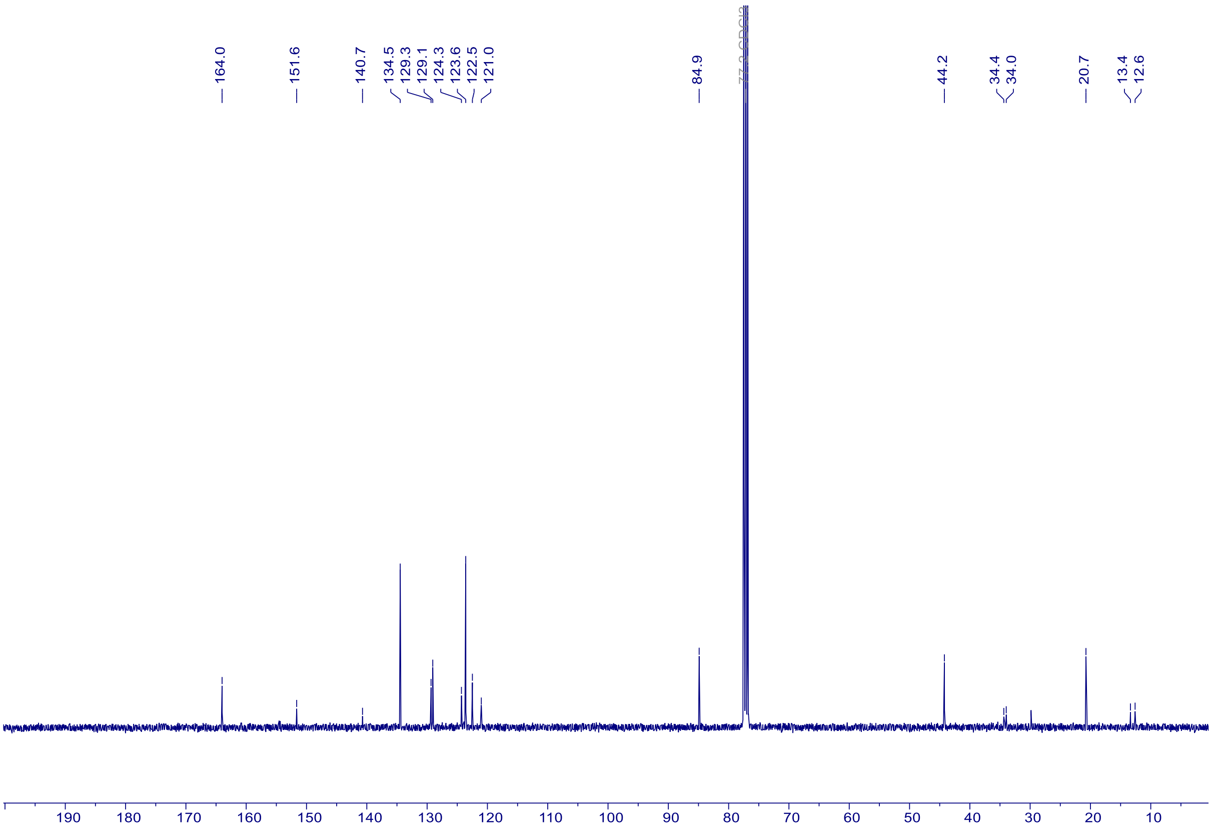

5m – ^{1}H NMR (400 MHz, $CDCl_3$)

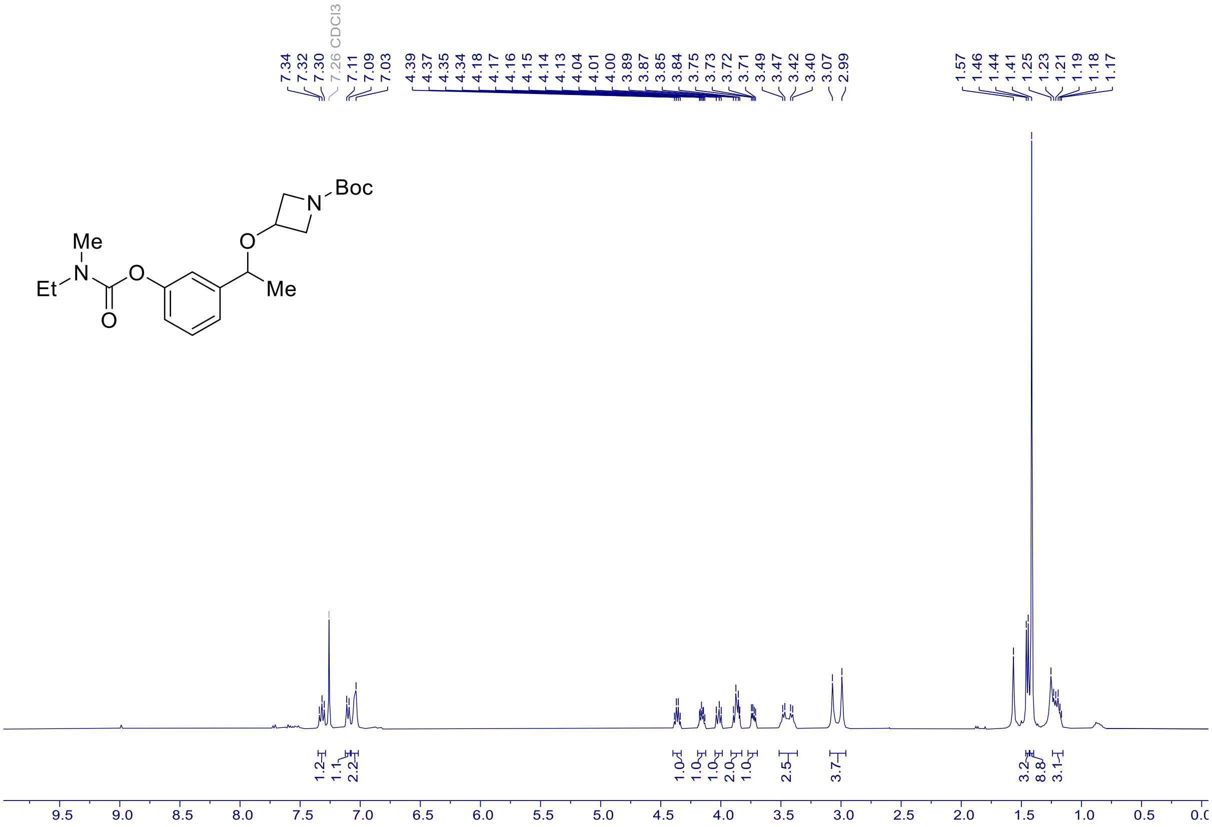

5m – ^{13}C NMR (101 MHz, $CDCl_3$)

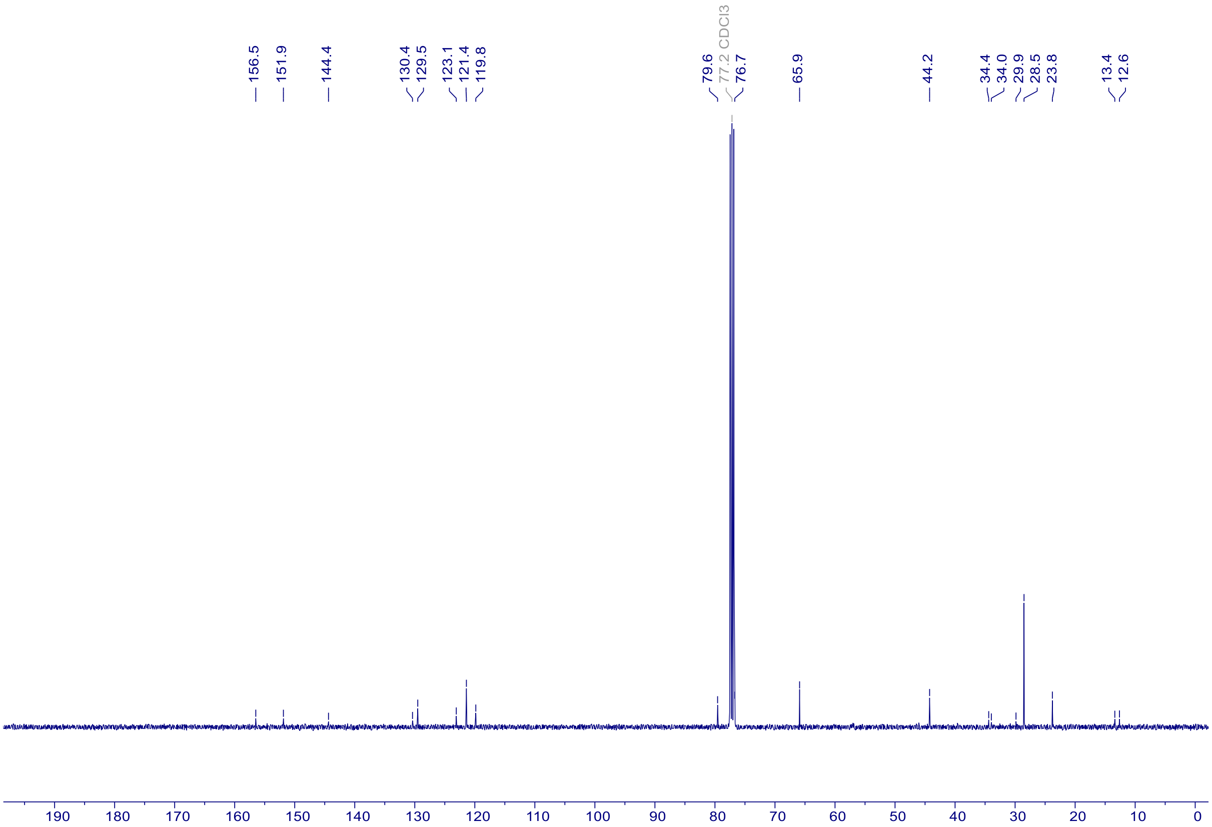

5n – ^{1}H NMR (400 MHz, $CDCl_3$)

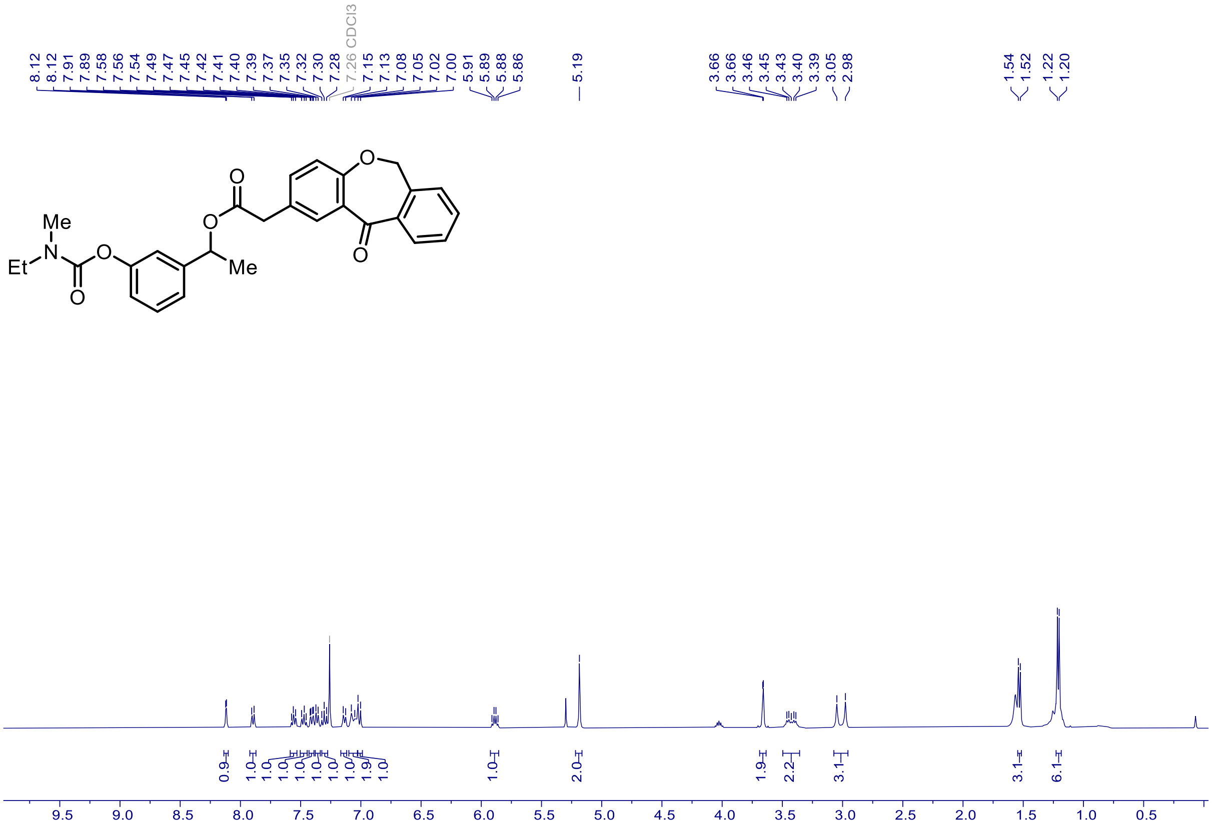

5n – ^{13}C NMR (101 MHz, $CDCl_3$)

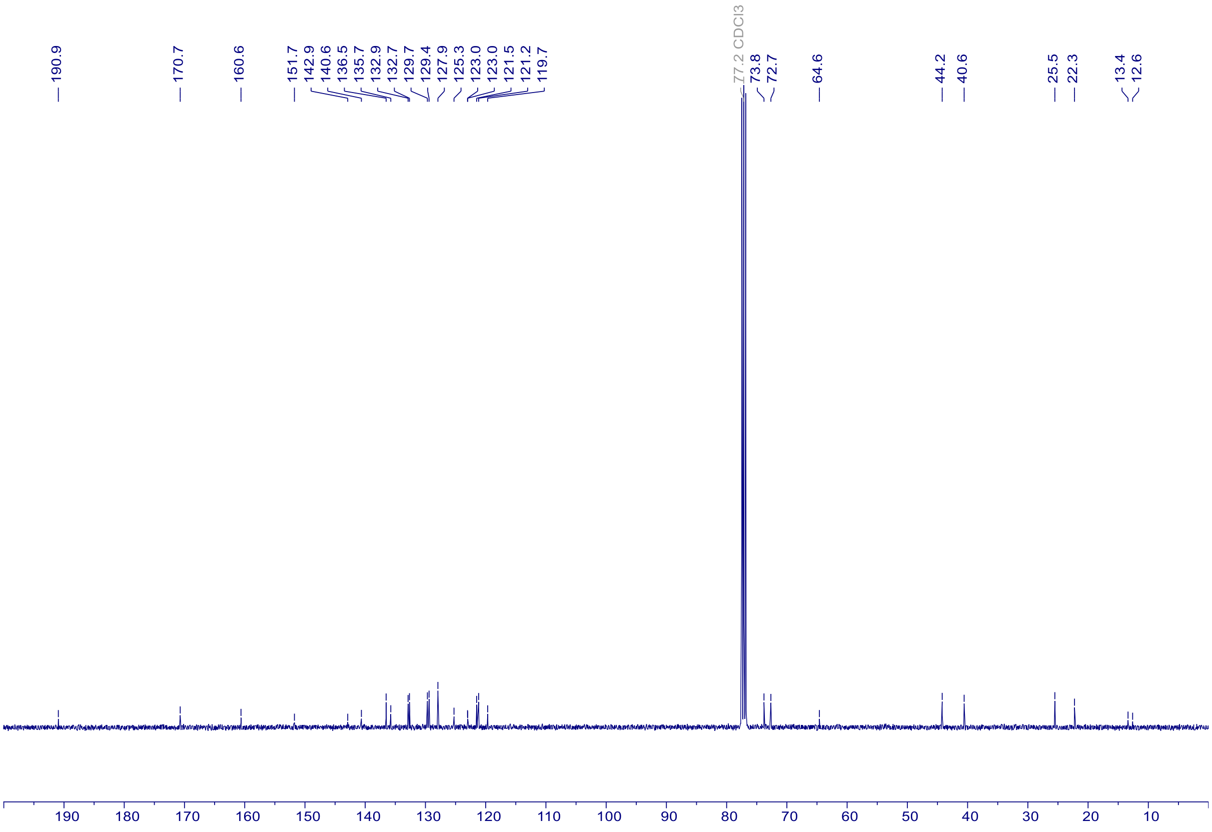

5o – ^{1}H NMR (600 MHz, CDCl$_3$)

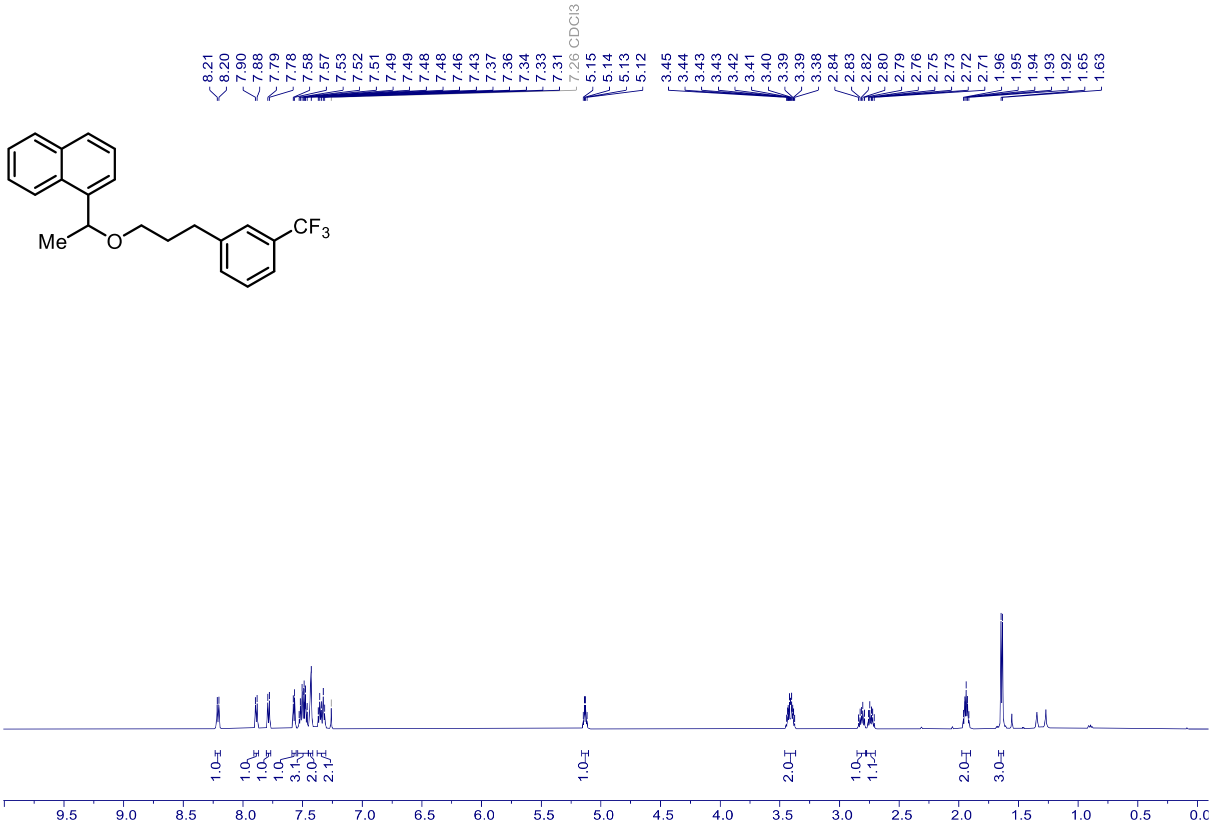

5o – ^{13}C NMR (151 MHz, CDCl$_3$)

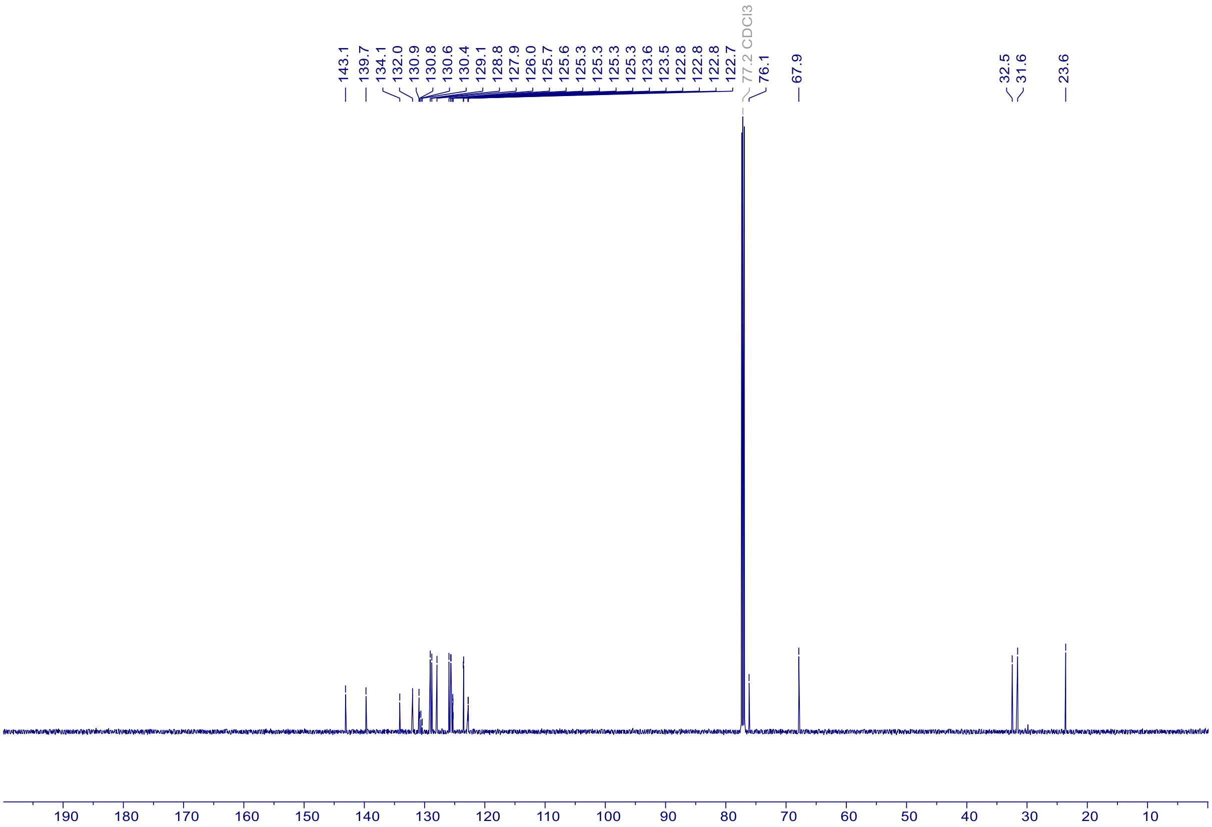

5o – ^{19}F NMR (565 MHz, $CDCl_3$)

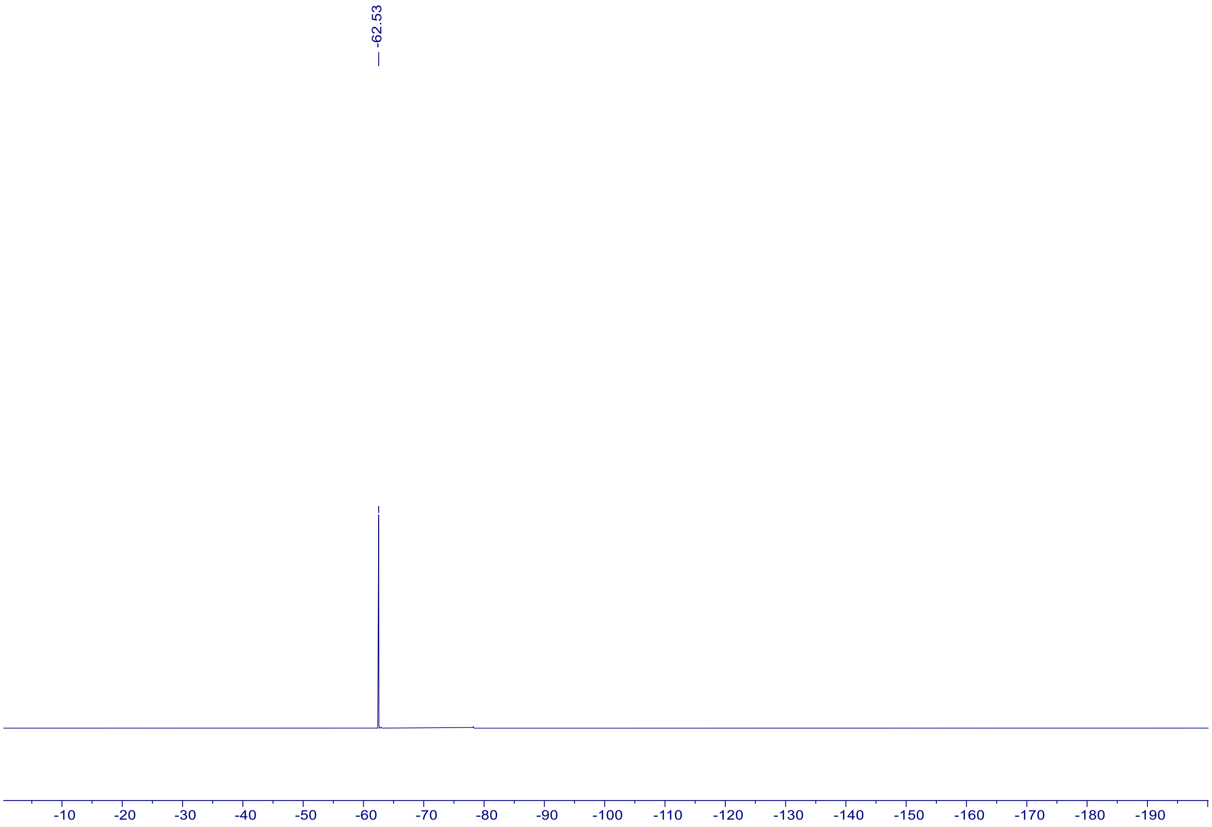

5p – ^{1}H NMR (600 MHz, $CDCl_3$)

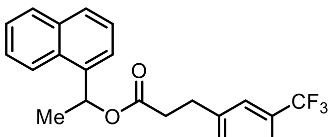

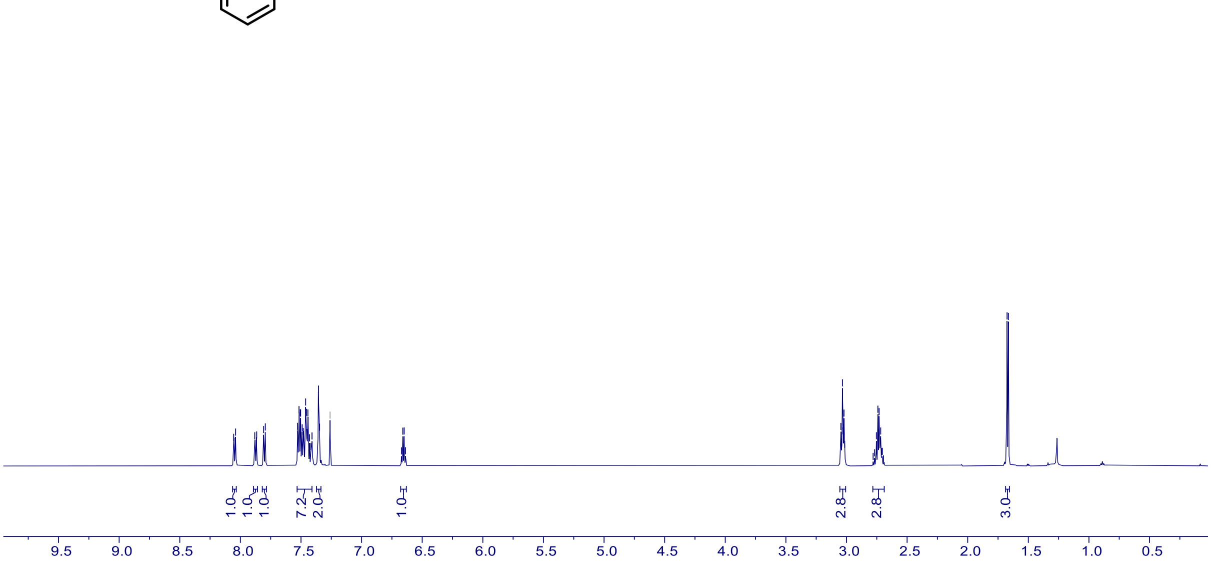

5p – ^{13}C NMR (151 MHz, $CDCl_3$)

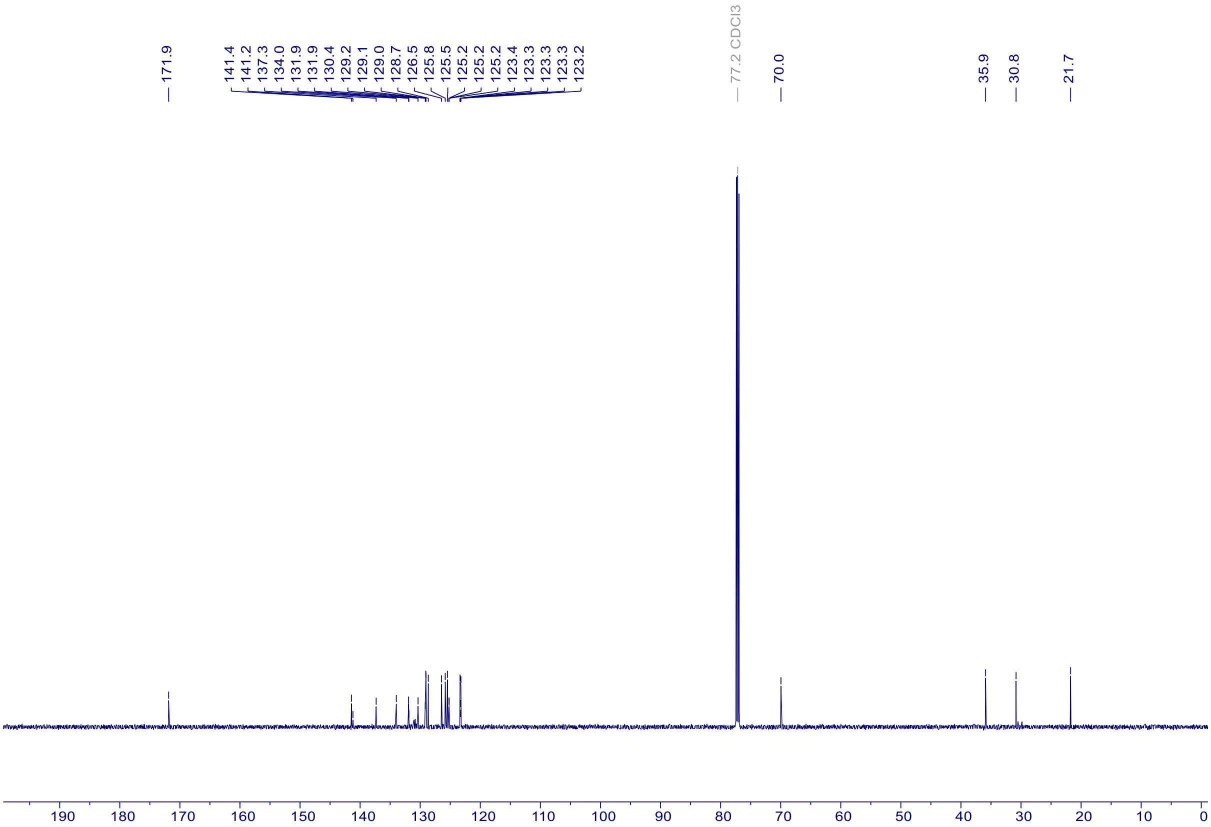

5p – ^{19}F NMR (565 MHz, $CDCl_3$)

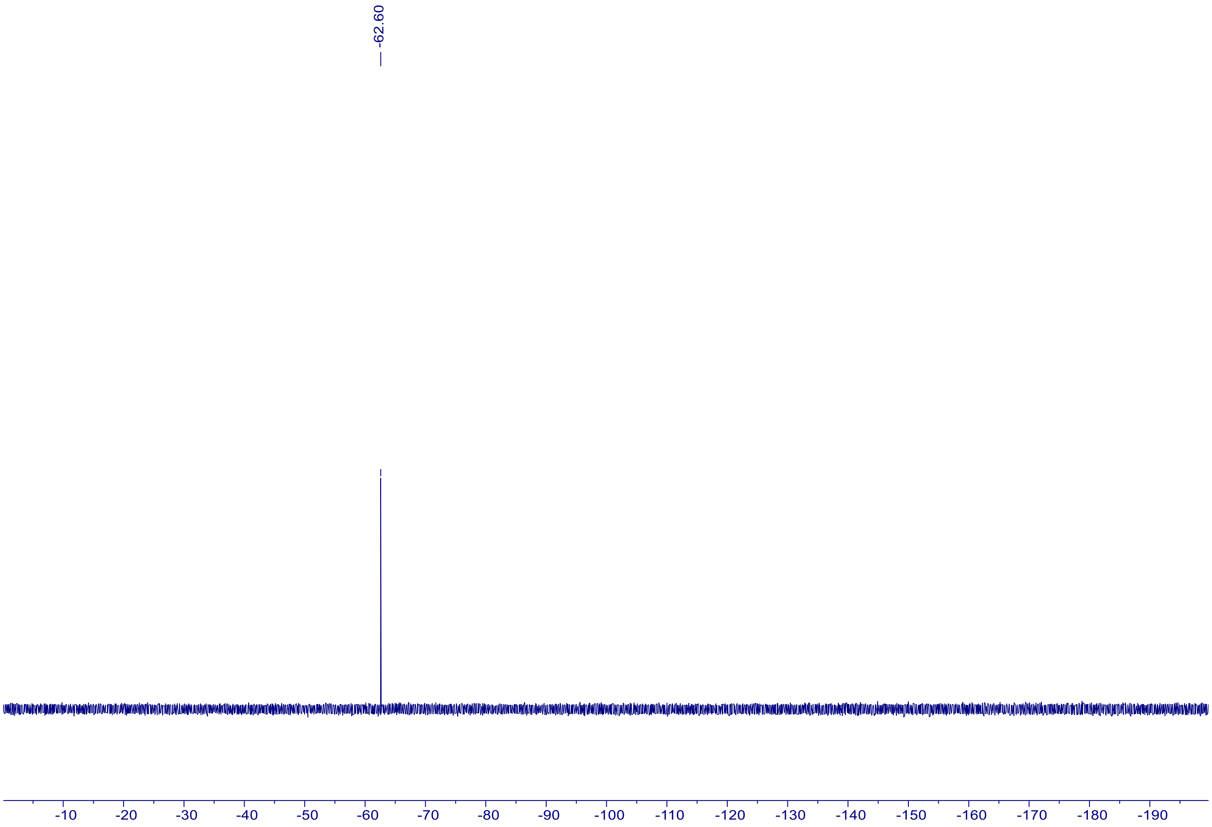

6c – ^{1}H NMR (600 MHz, $CDCl_3$)

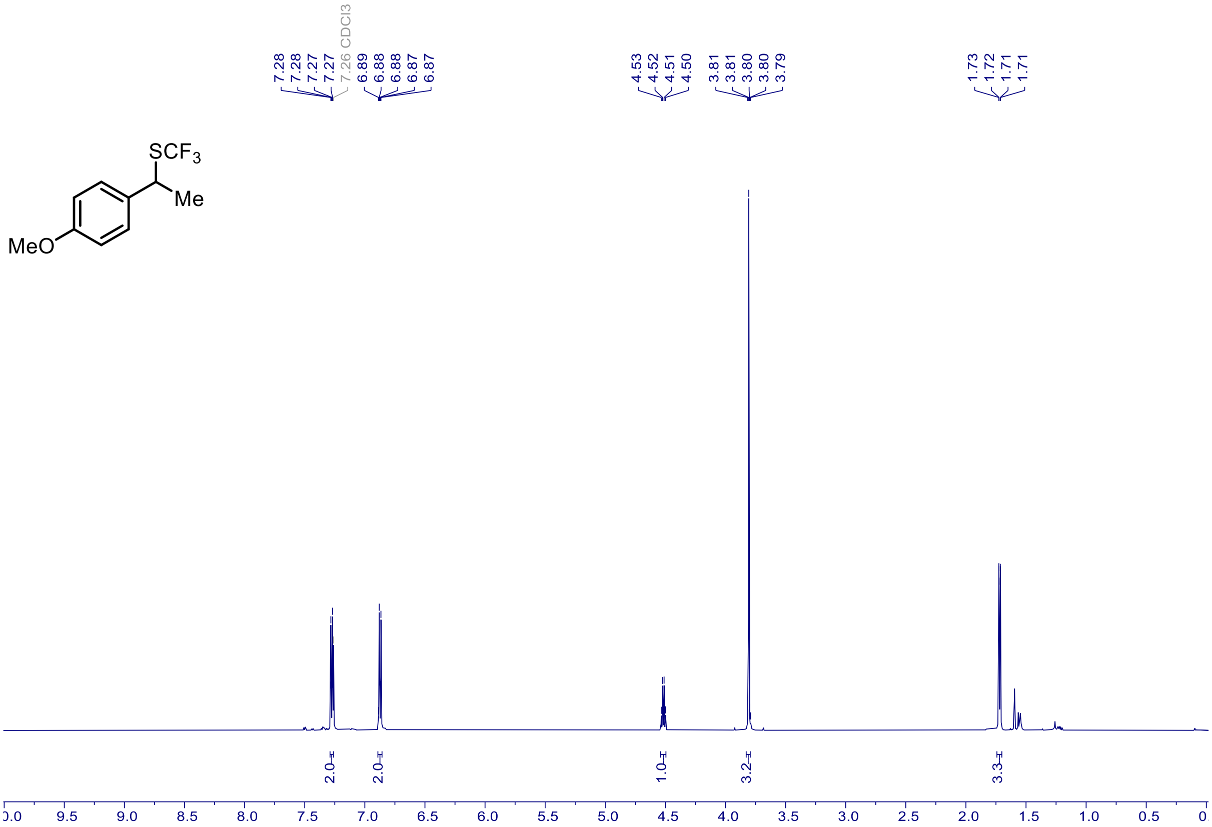

6c – ^{13}C NMR (151 MHz, $CDCl_3$)

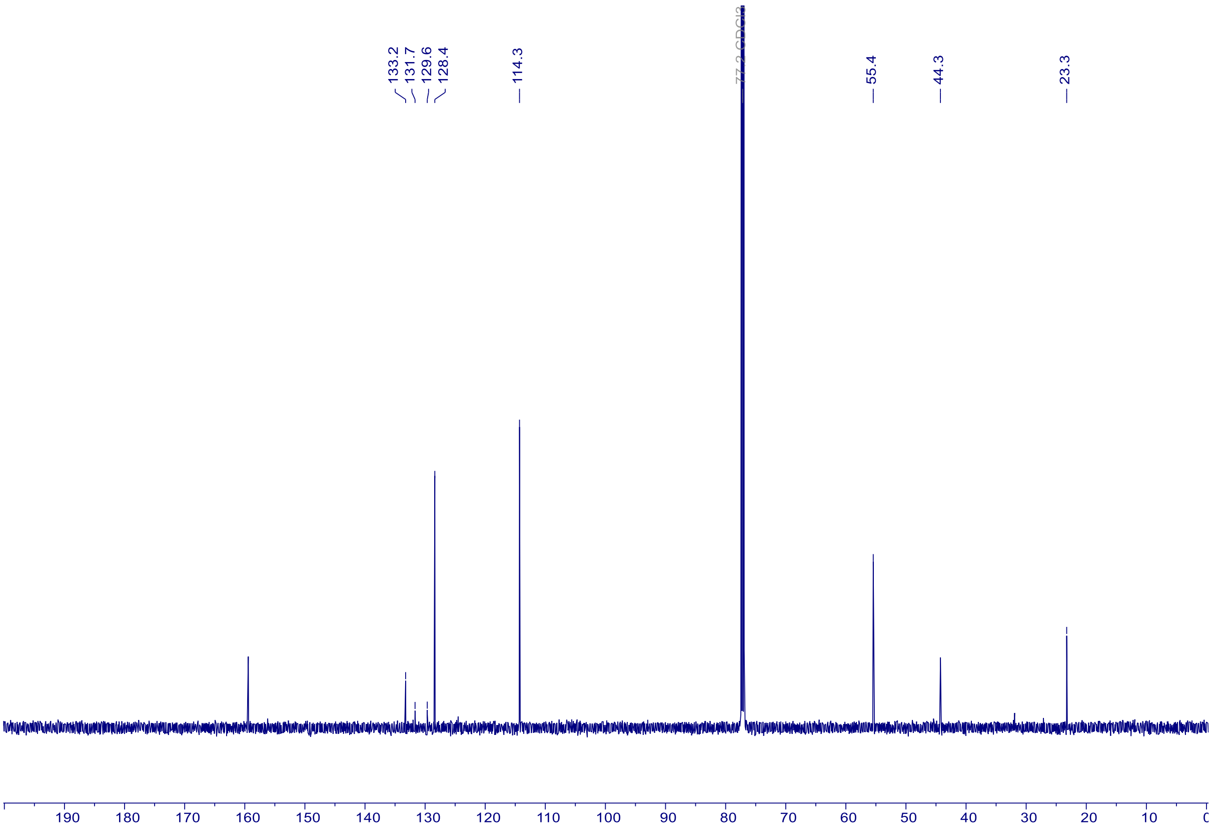

6c – ^{19}F NMR (565 MHz, $CDCl_3$)

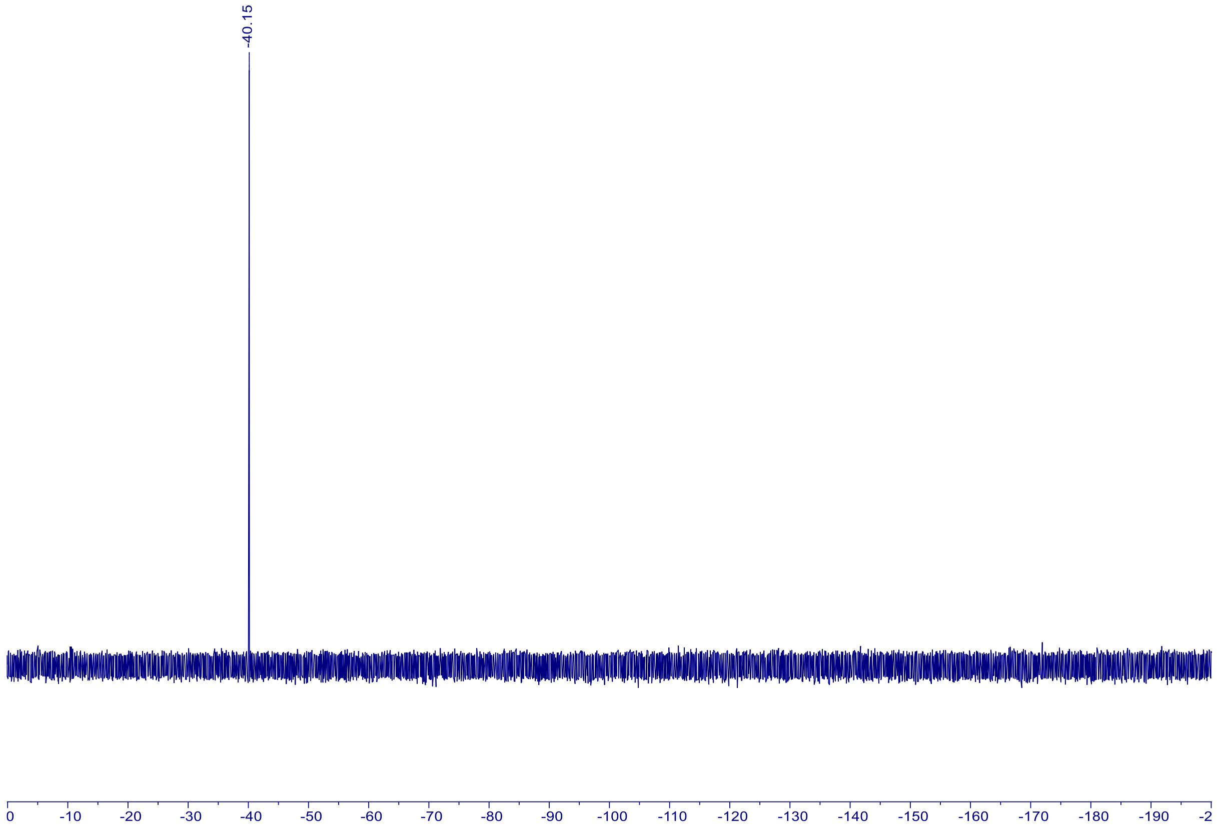

6e – ¹H NMR (600 MHz, $CDCl_3$)

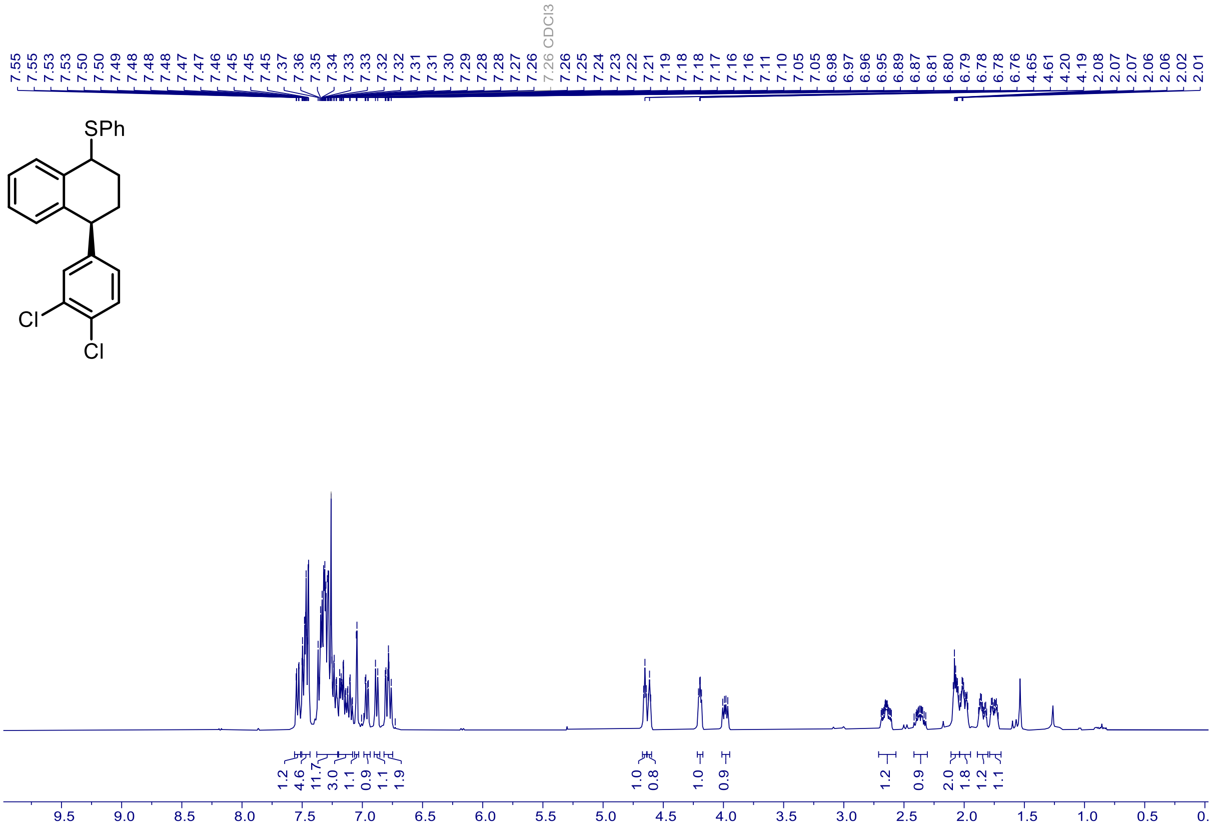

6e – ¹³C NMR (151 MHz, $CDCl_3$)

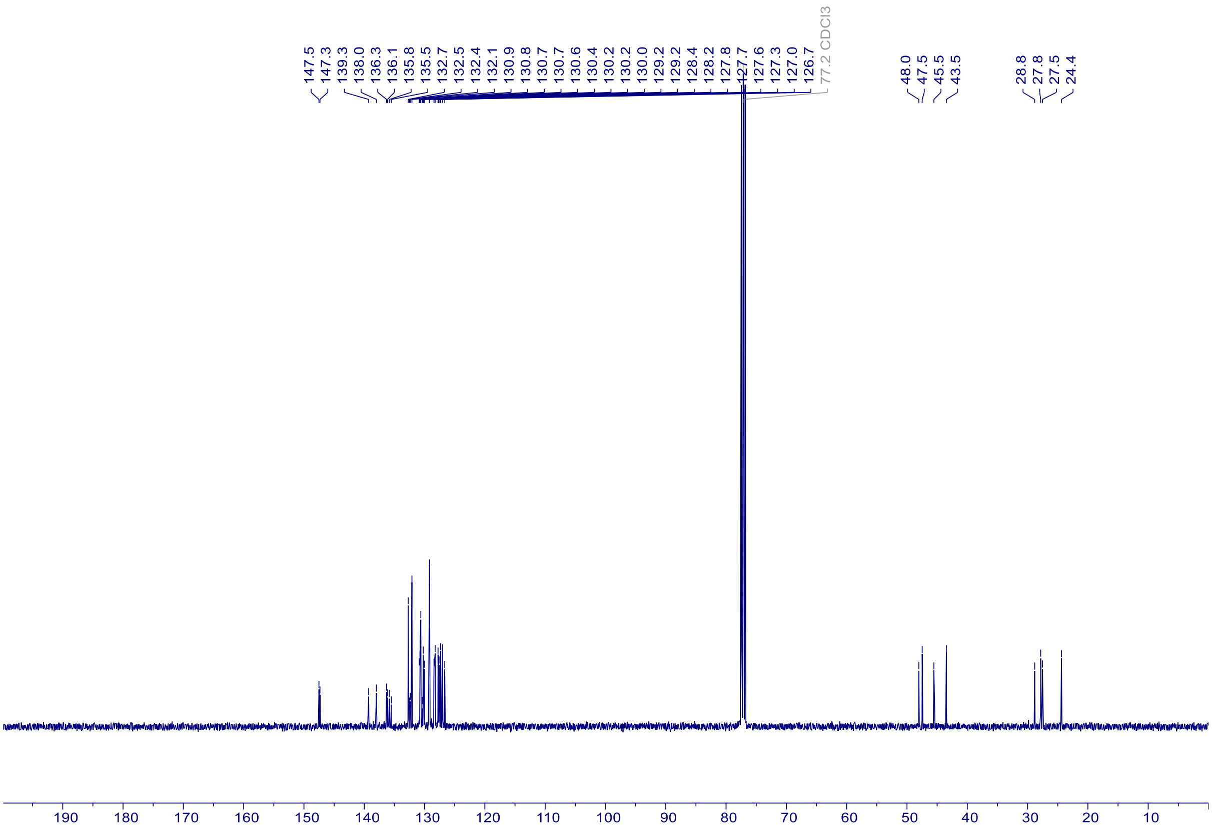

6fa – ^{1}H NMR (600 MHz, $CDCl_3$)

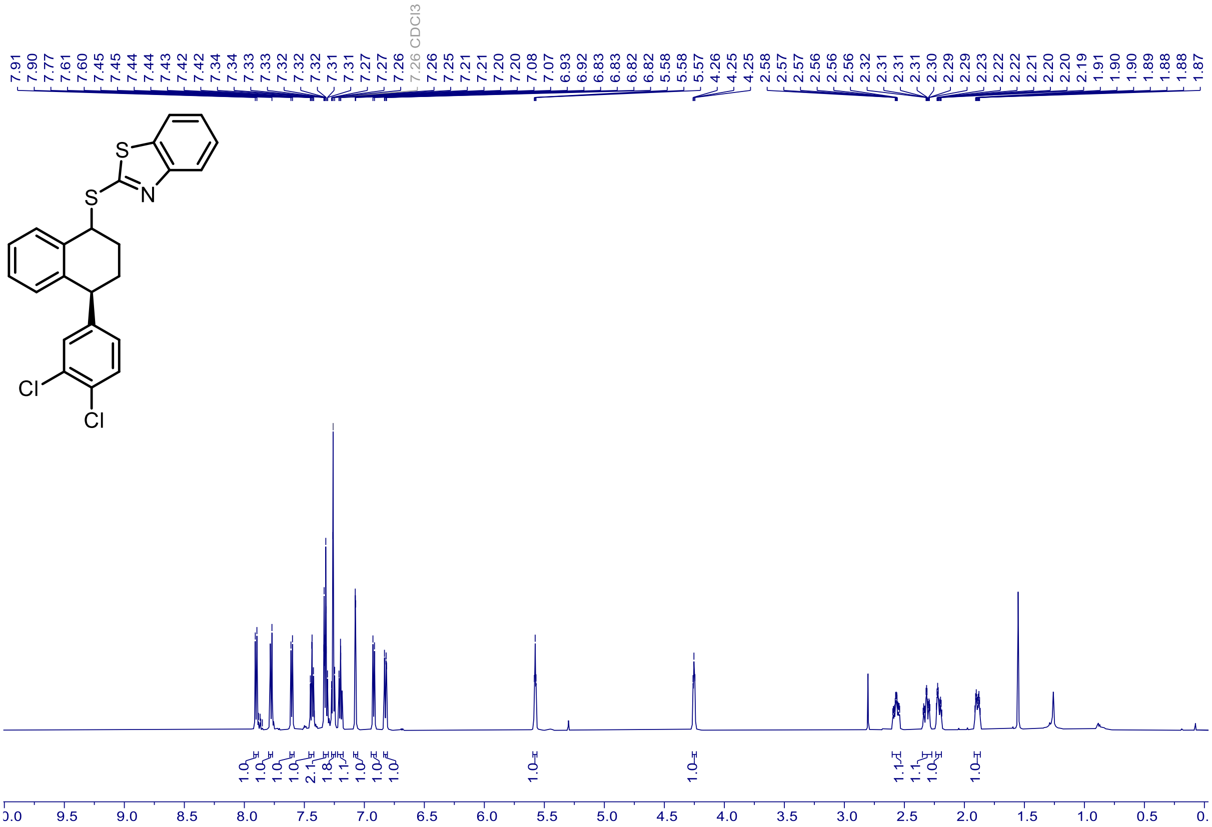

6fa – ^{13}C NMR (151 MHz, $CDCl_3$)

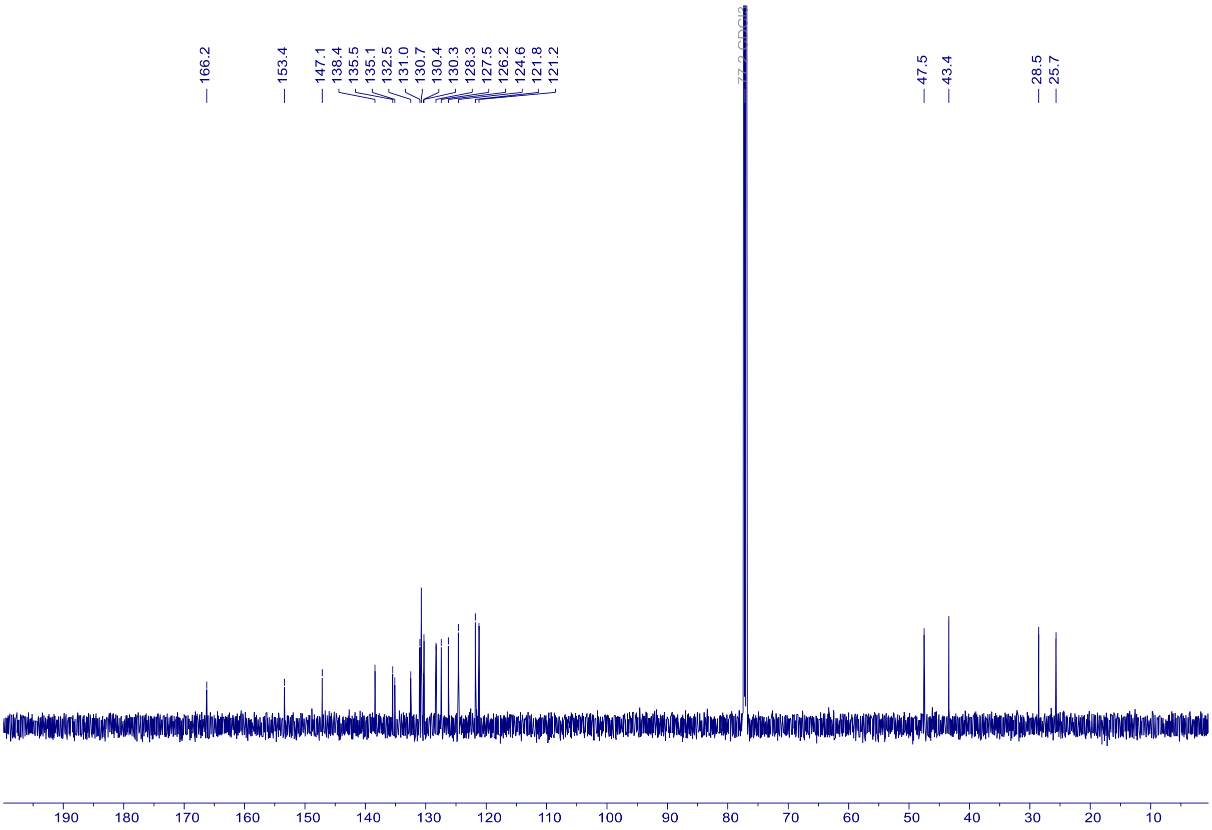

6fb – ^{1}H NMR (400 MHz, $CDCl_3$)

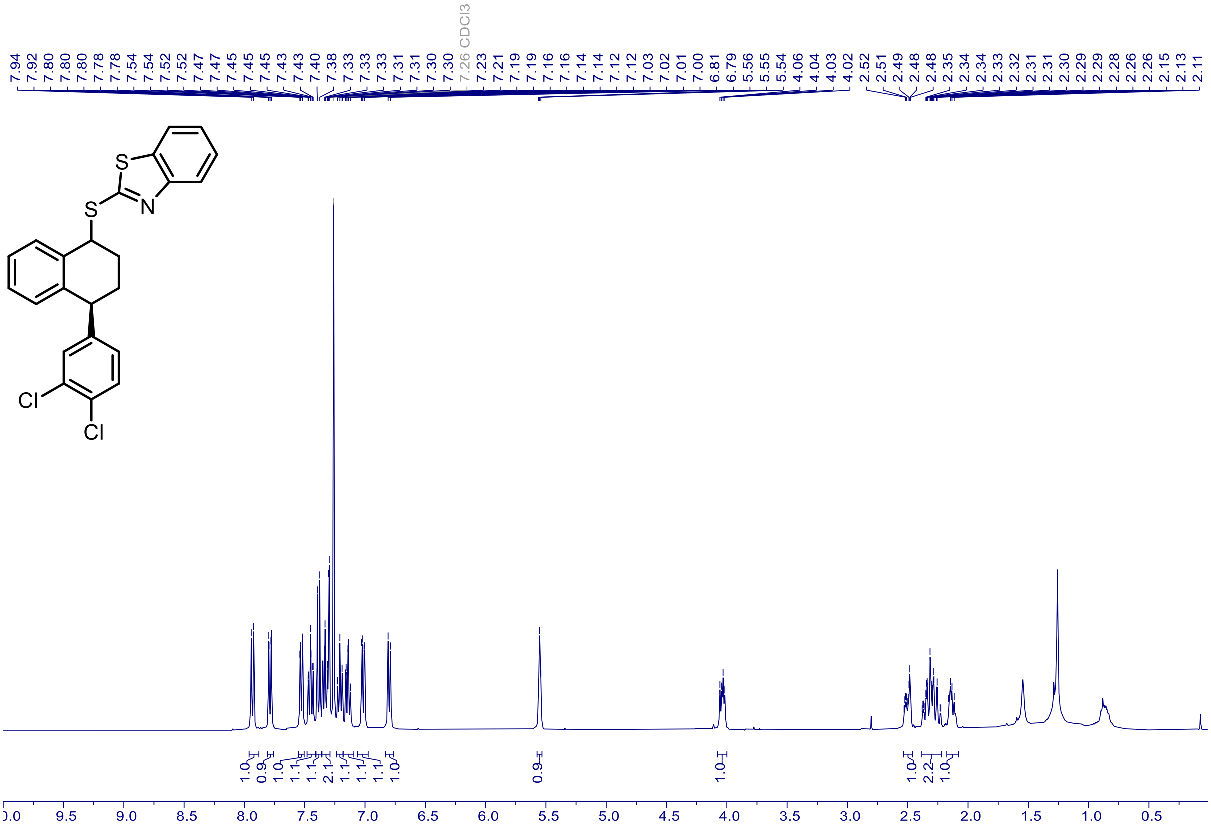

6fb – ^{13}C NMR (101 MHz, $CDCl_3$)

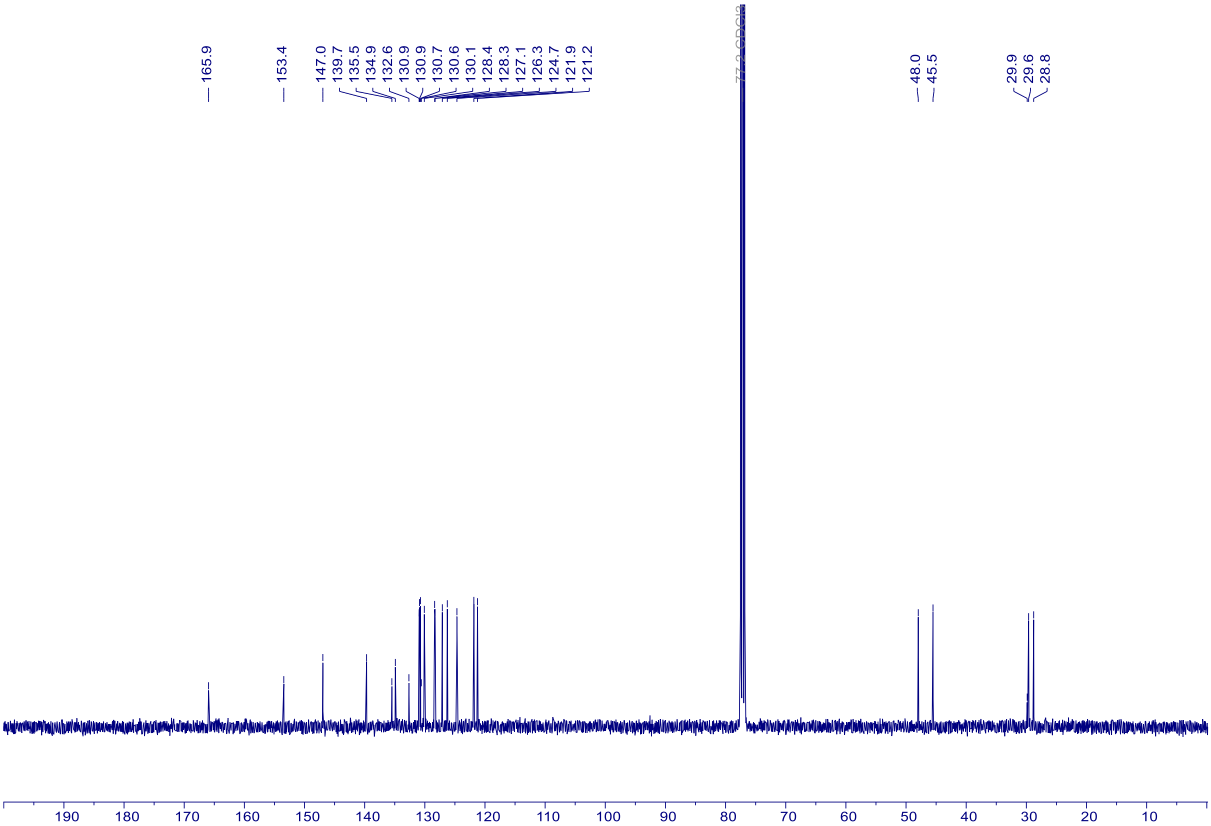

6ga – ^{1}H NMR (400 MHz, $CDCl_3$)

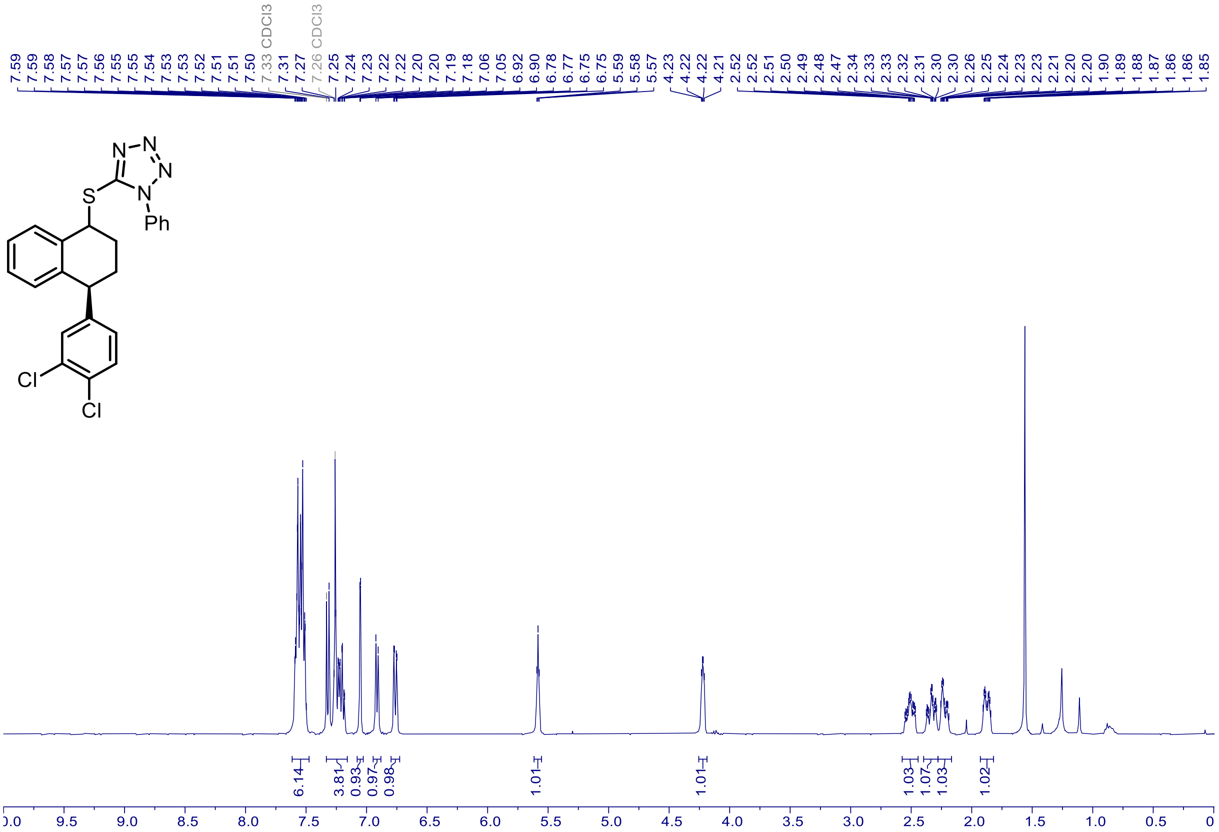

6ga – ^{13}C NMR (101 MHz, $CDCl_3$)

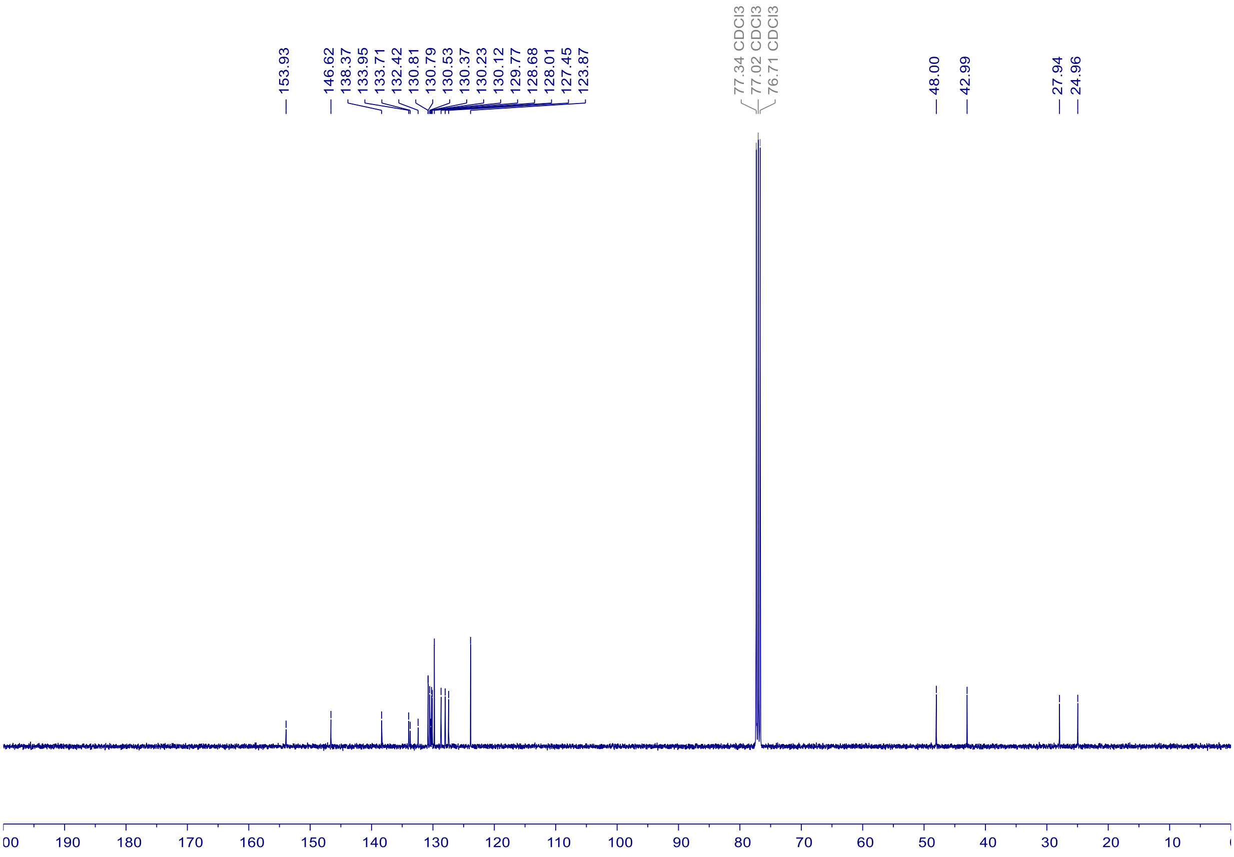

6gb – ^{1}H NMR (400 MHz, $CDCl_3$)

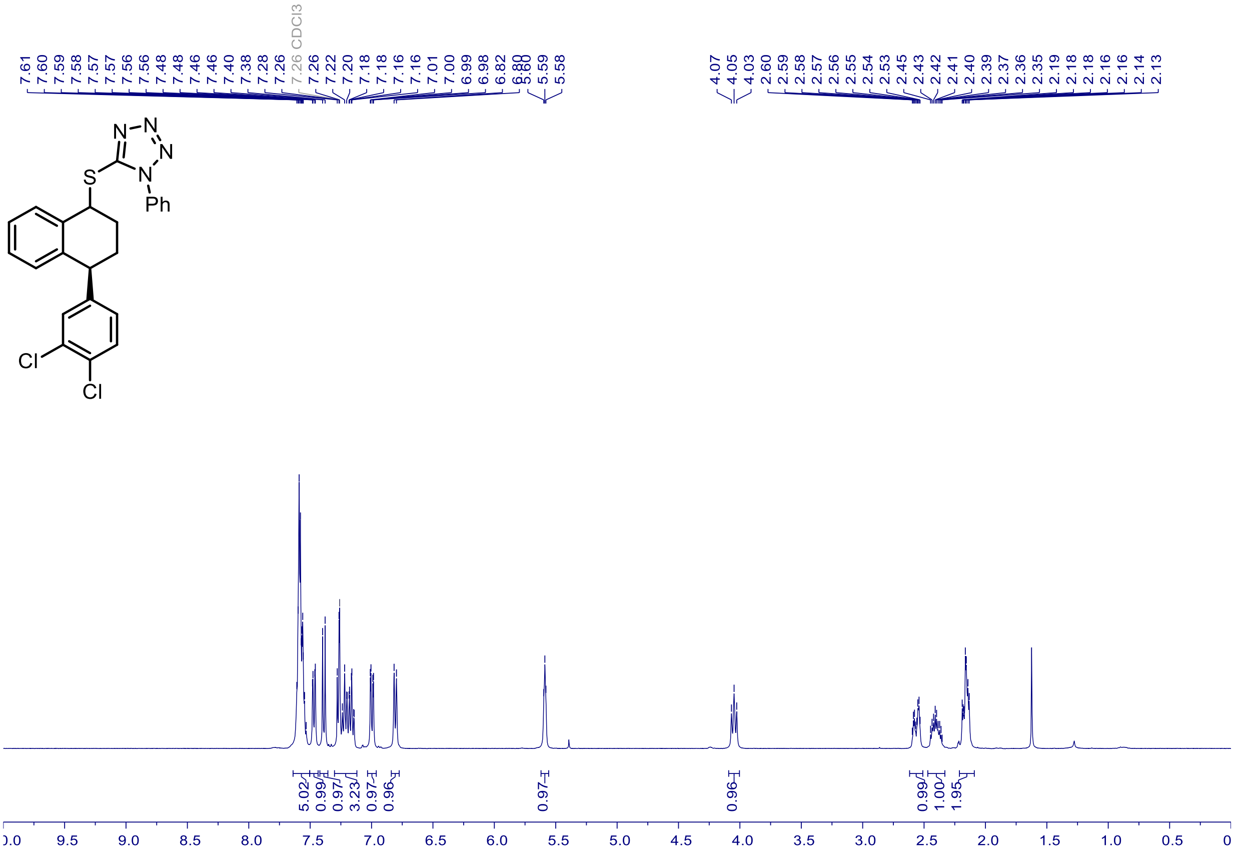

6gb – ^{13}C NMR (101 MHz, $CDCl_3$)

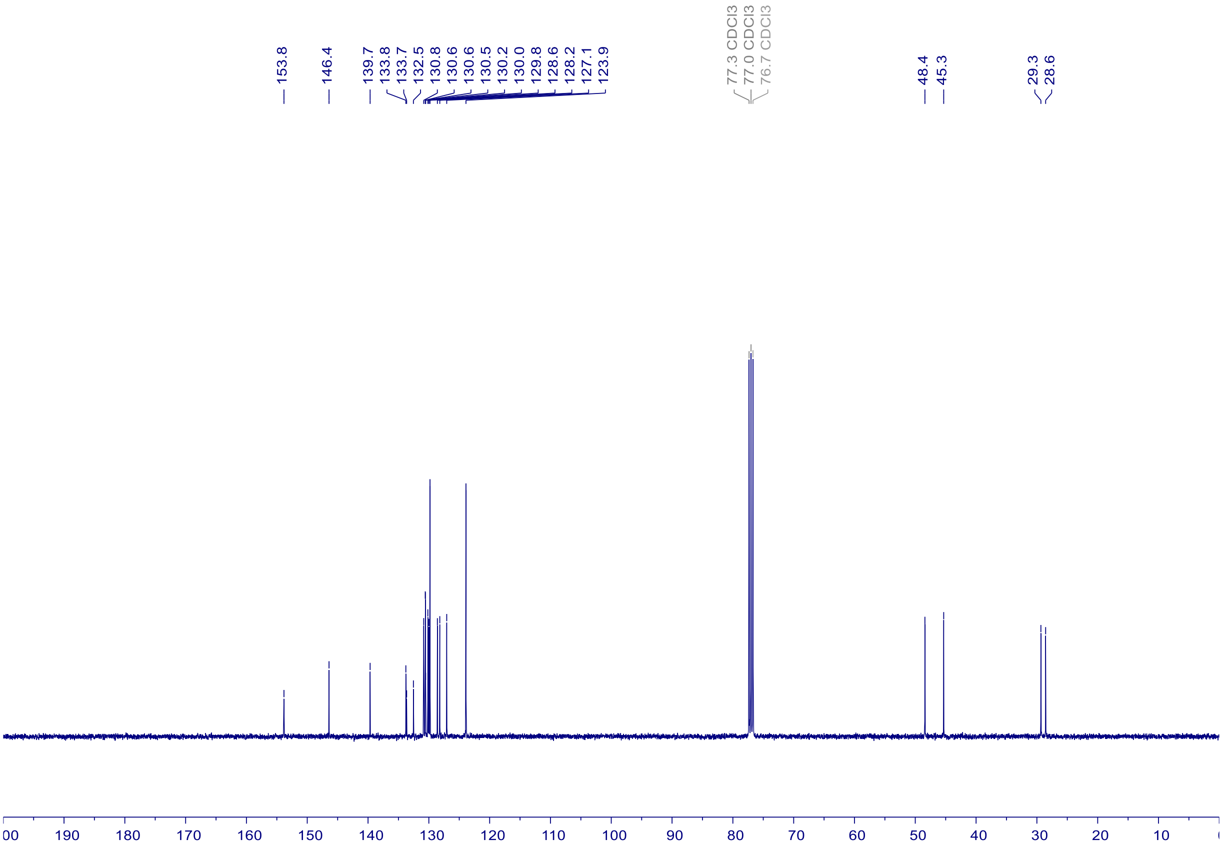

6h – ^{1}H NMR (400 MHz, CDCl$_3$)

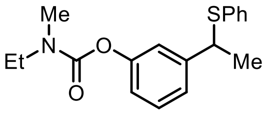

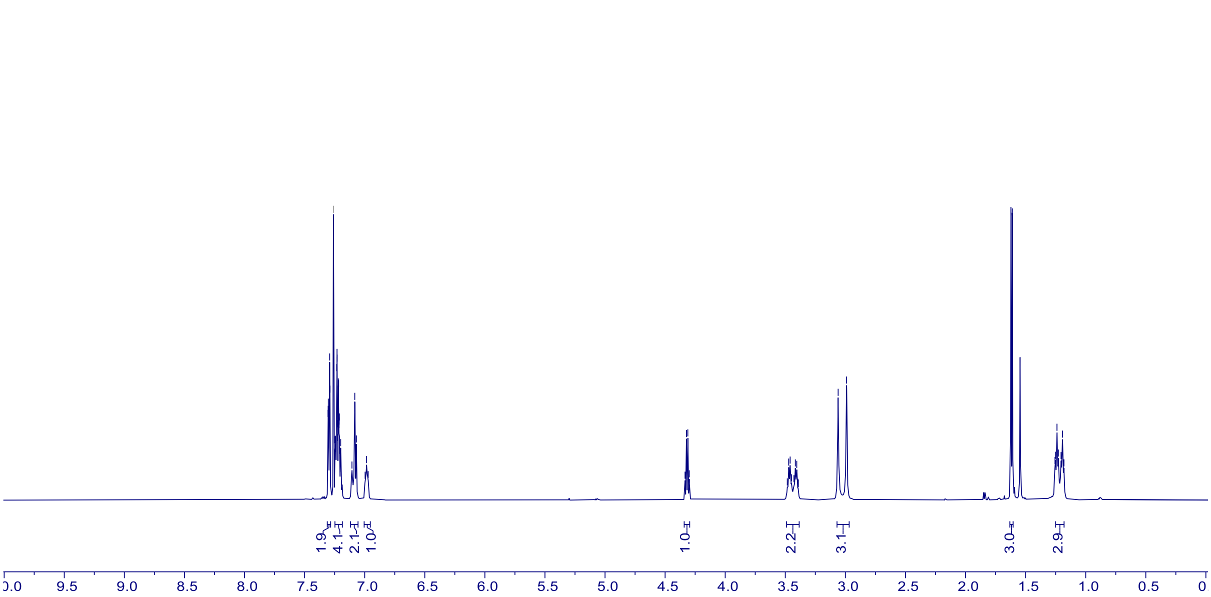

6h – ^{13}C NMR (101 MHz, CDCl$_3$)

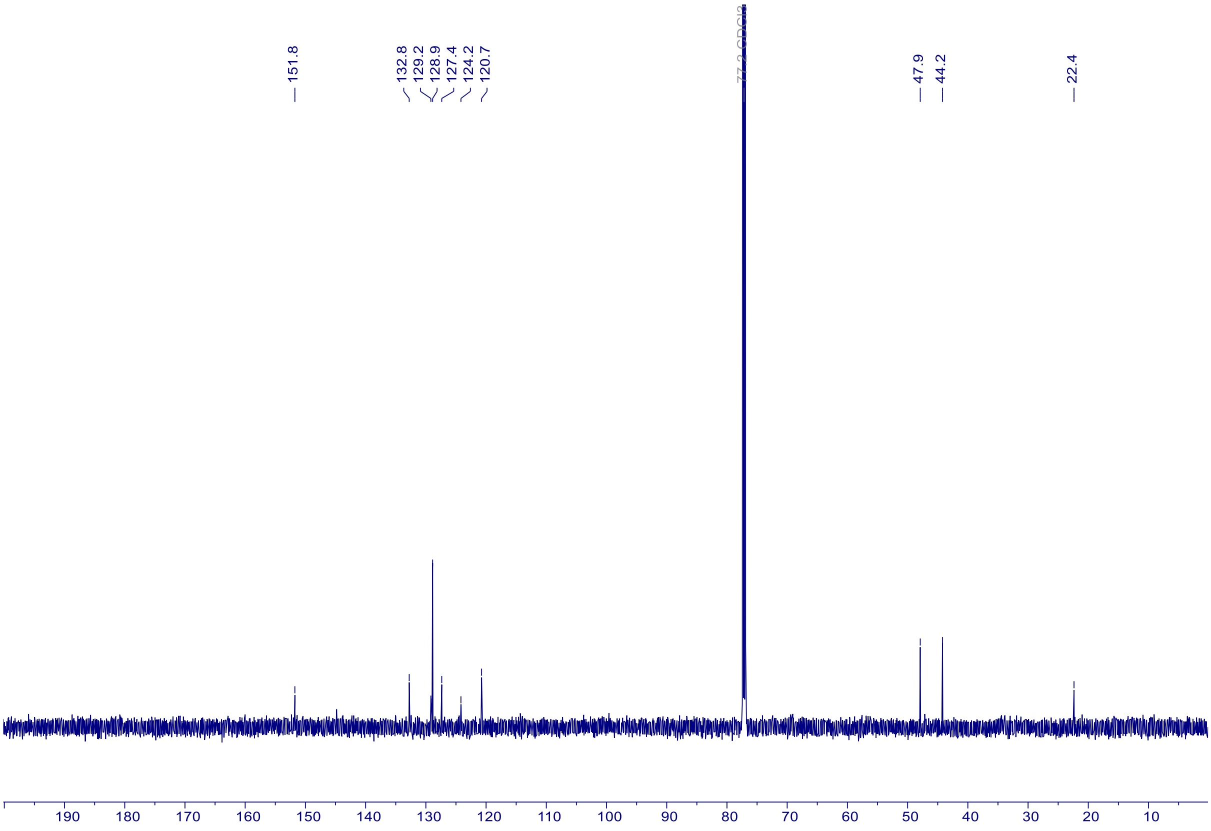

6i – ^{1}H NMR (400 MHz, $CDCl_3$)

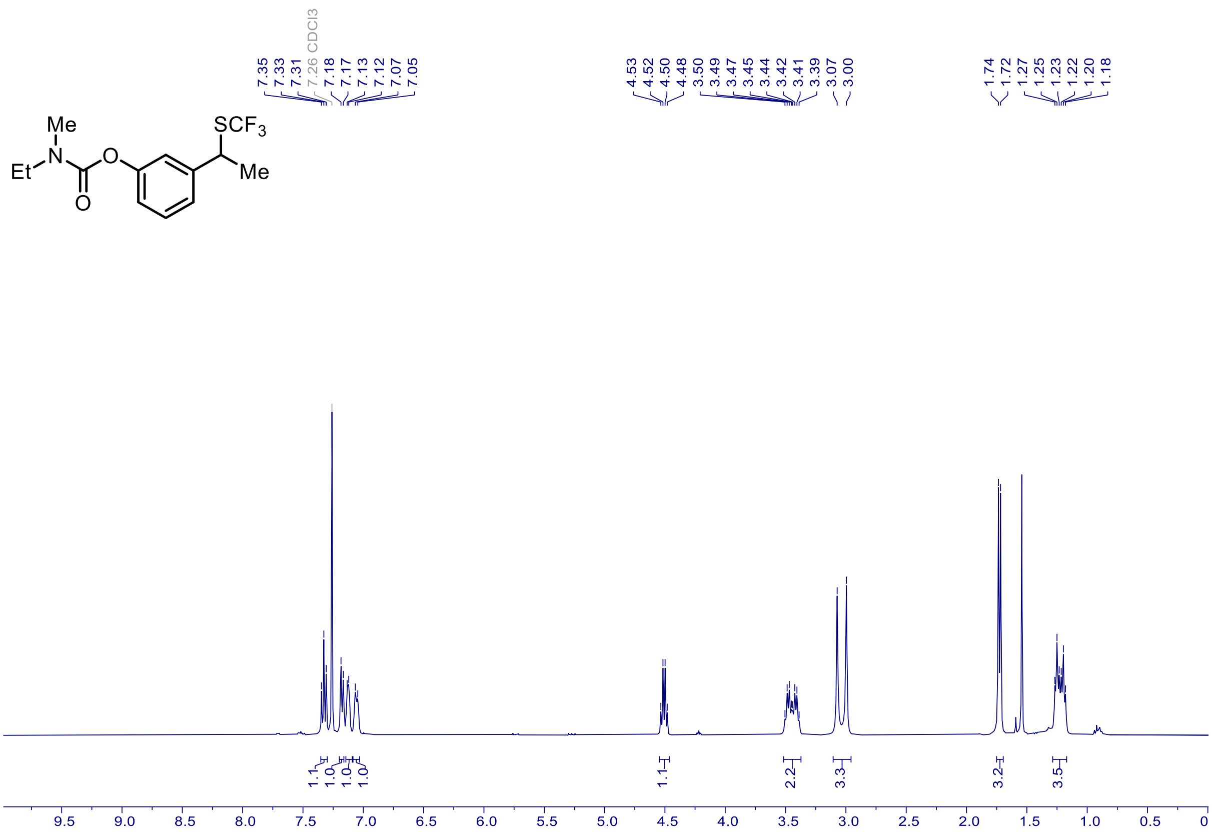

6i – ^{13}C NMR (101 MHz, $CDCl_3$)

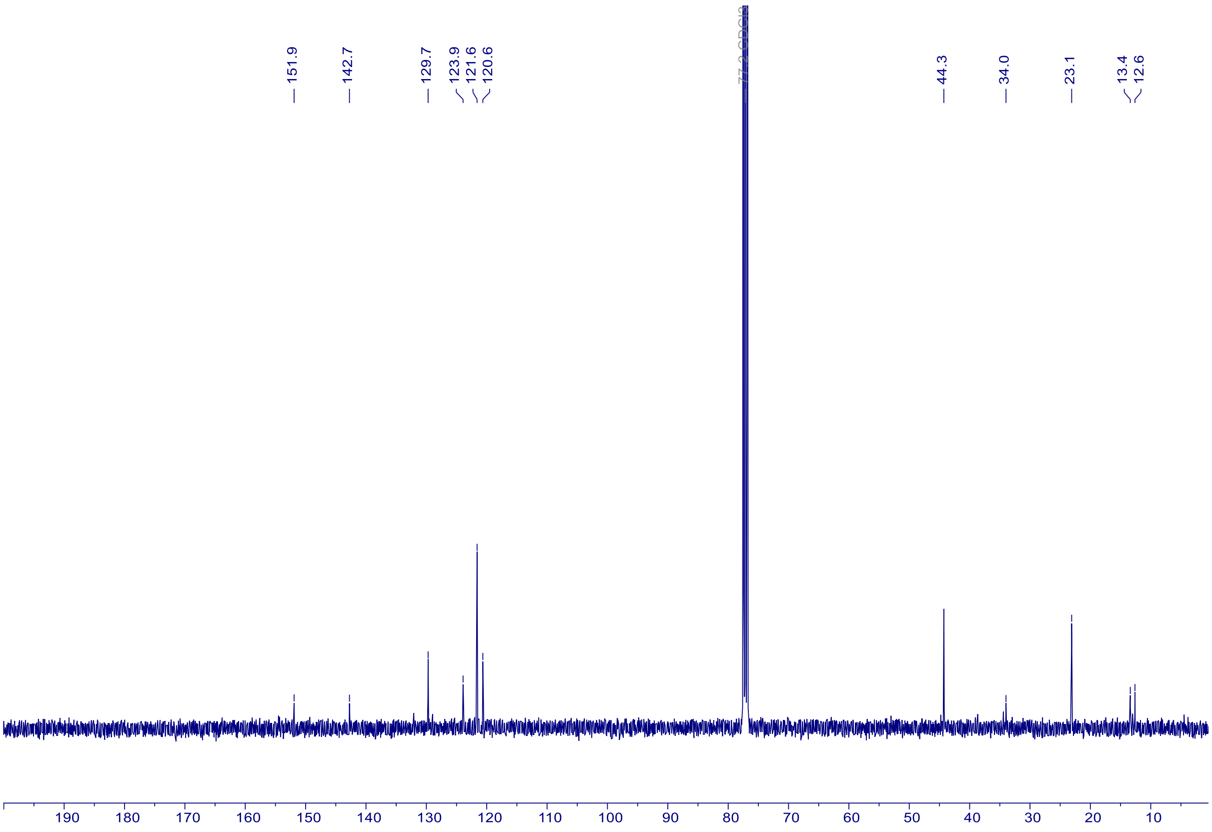

6i – ^{19}F NMR (565 MHz, $CDCl_3$)

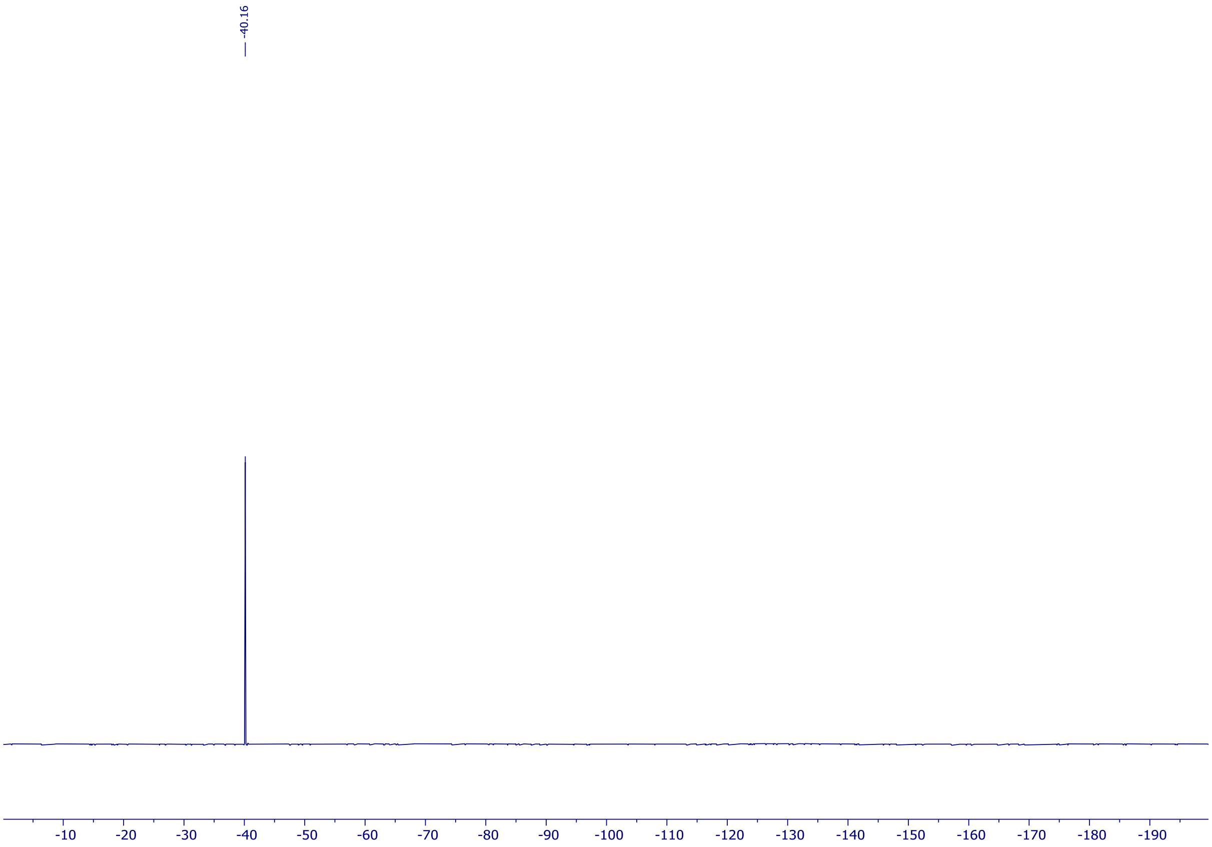

6j – ^{1}H NMR (400 MHz, $CDCl_3$)

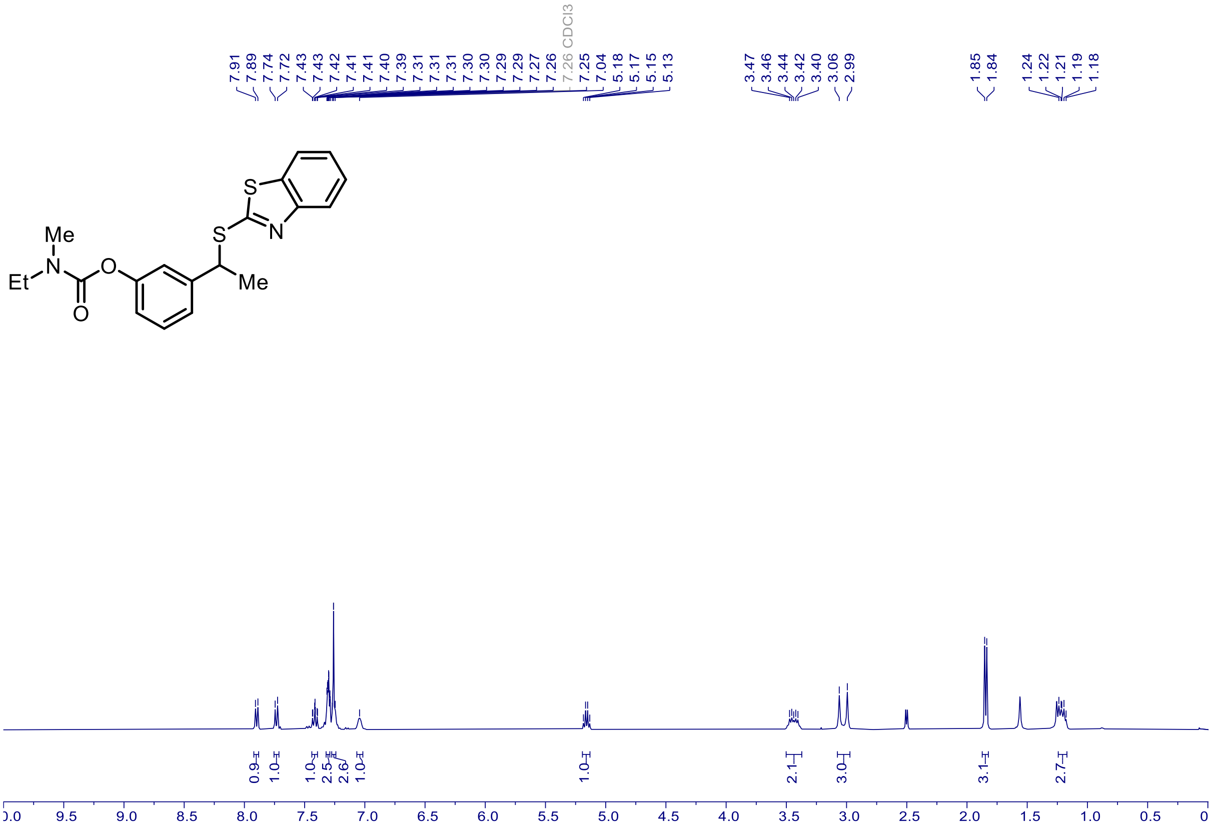

6j – ^{13}C NMR (101 MHz, $CDCl_3$)

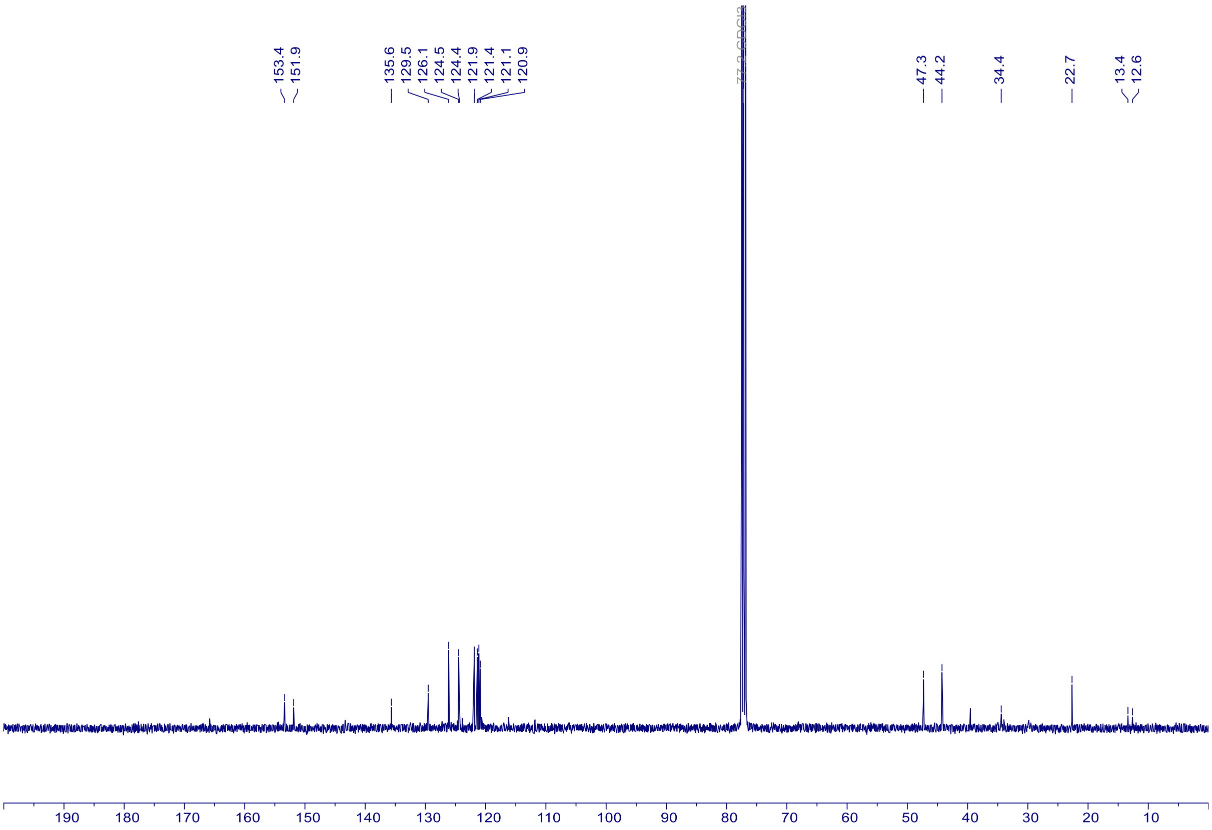

6k – ^{1}H NMR (400 MHz, $CDCl_3$)

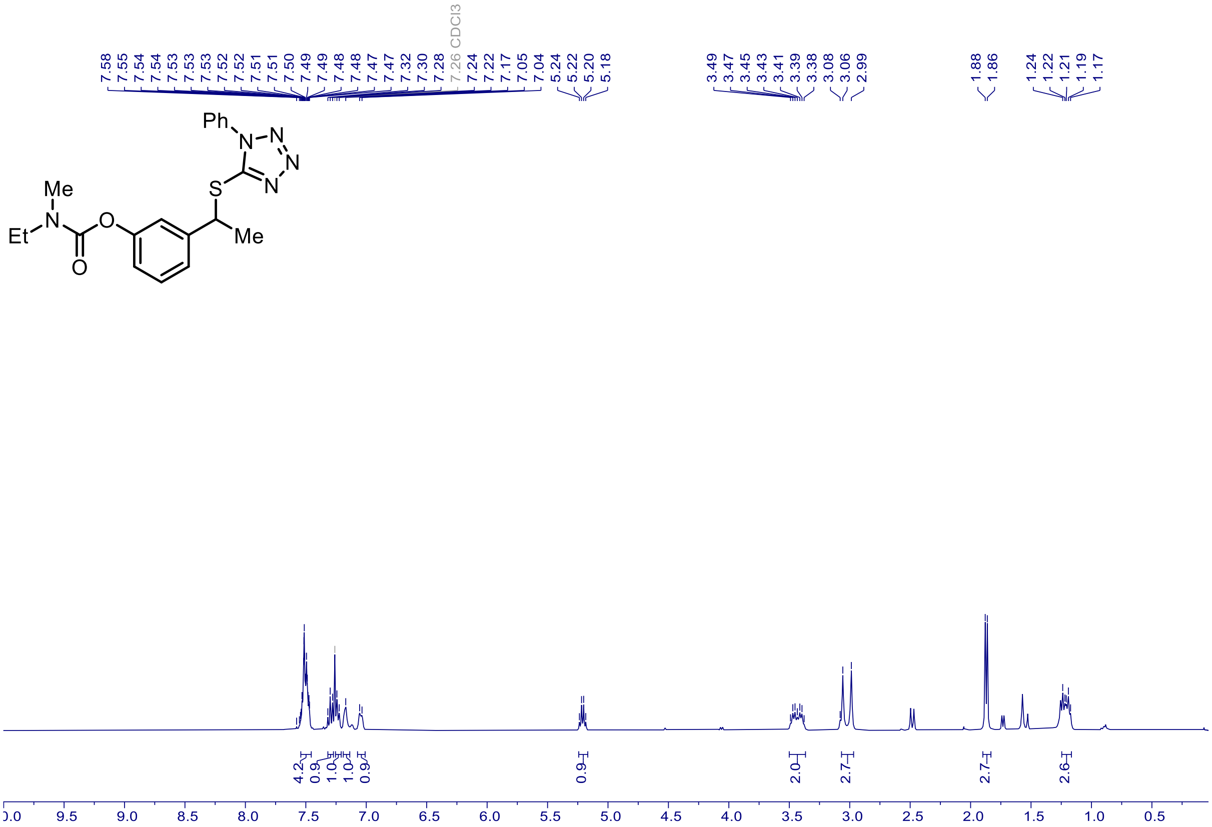

6k – ^{13}C NMR (101 MHz, $CDCl_3$)

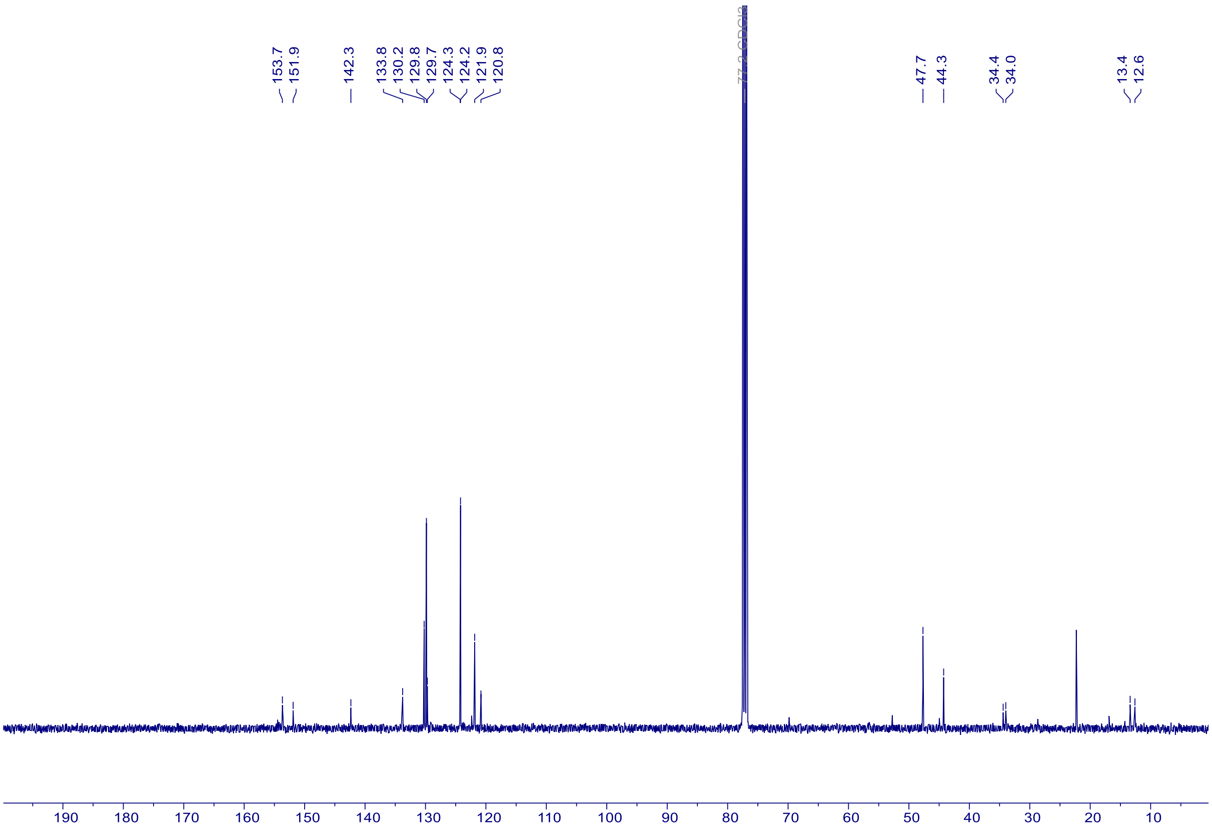

6l – ^{1}H NMR (600 MHz, $CDCl_3$)

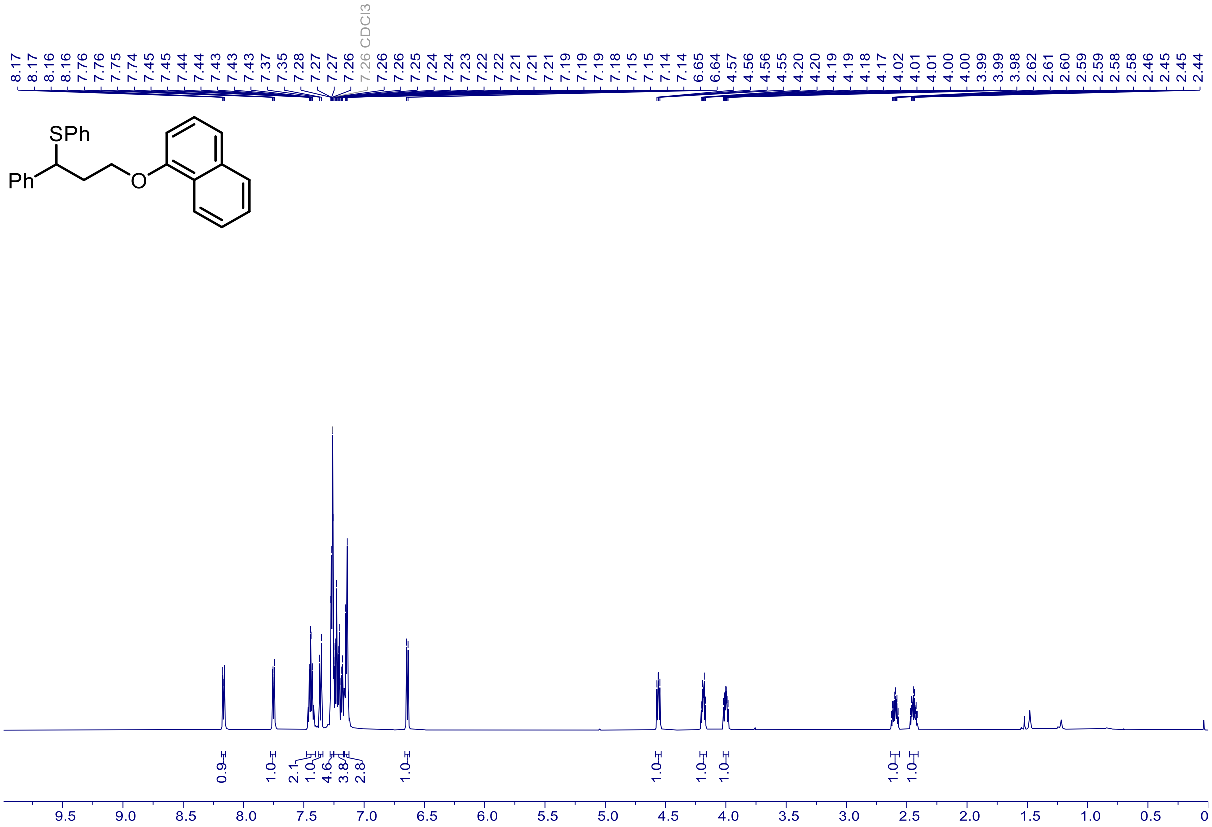

6l – ^{13}C NMR (151 MHz, $CDCl_3$)

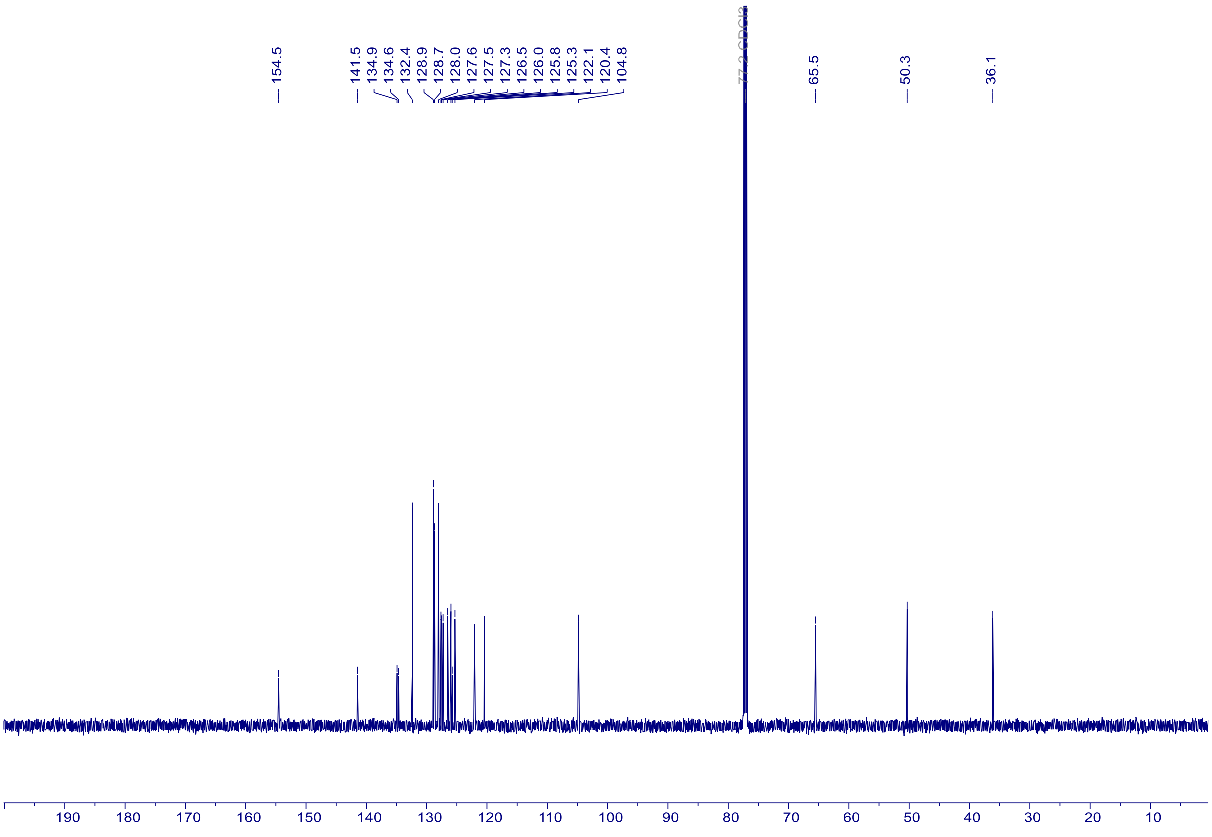

6m – ^{1}H NMR (600 MHz, $CDCl_3$)

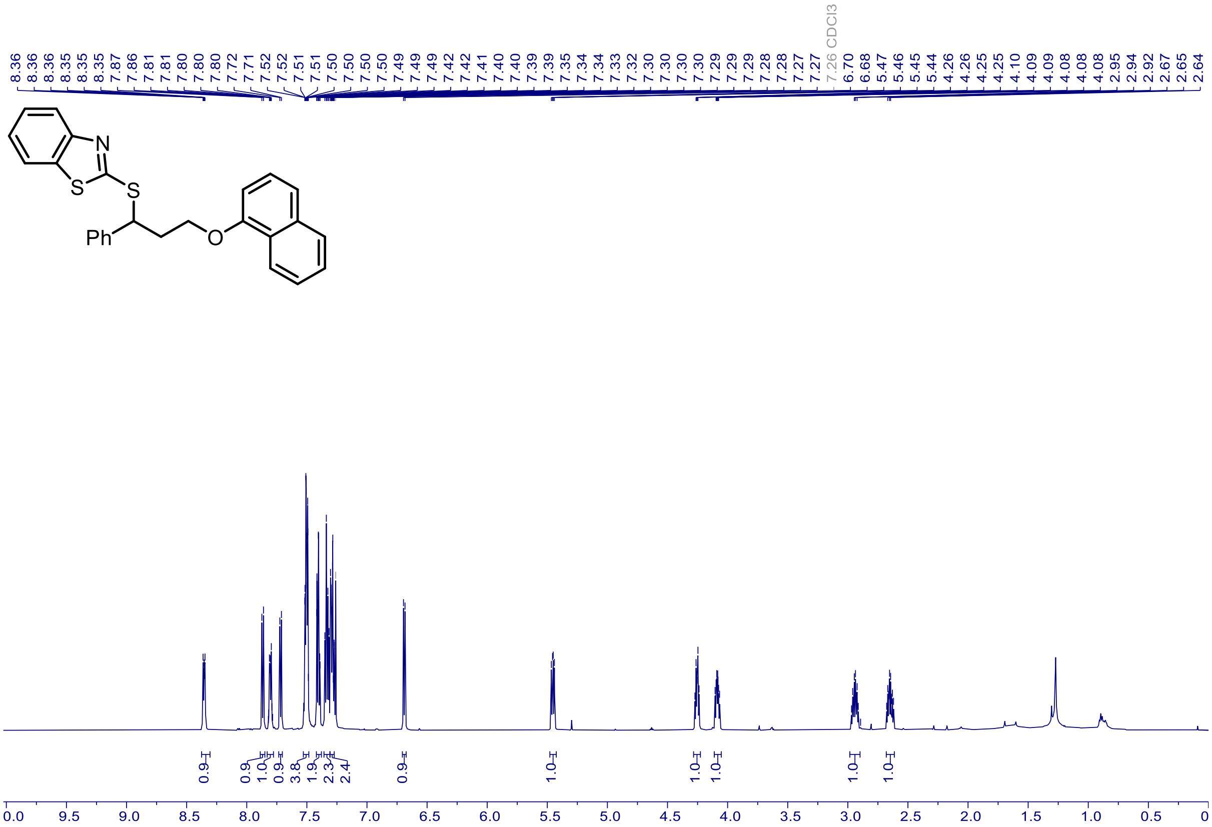

6m – ^{13}C NMR (151 MHz, $CDCl_3$)

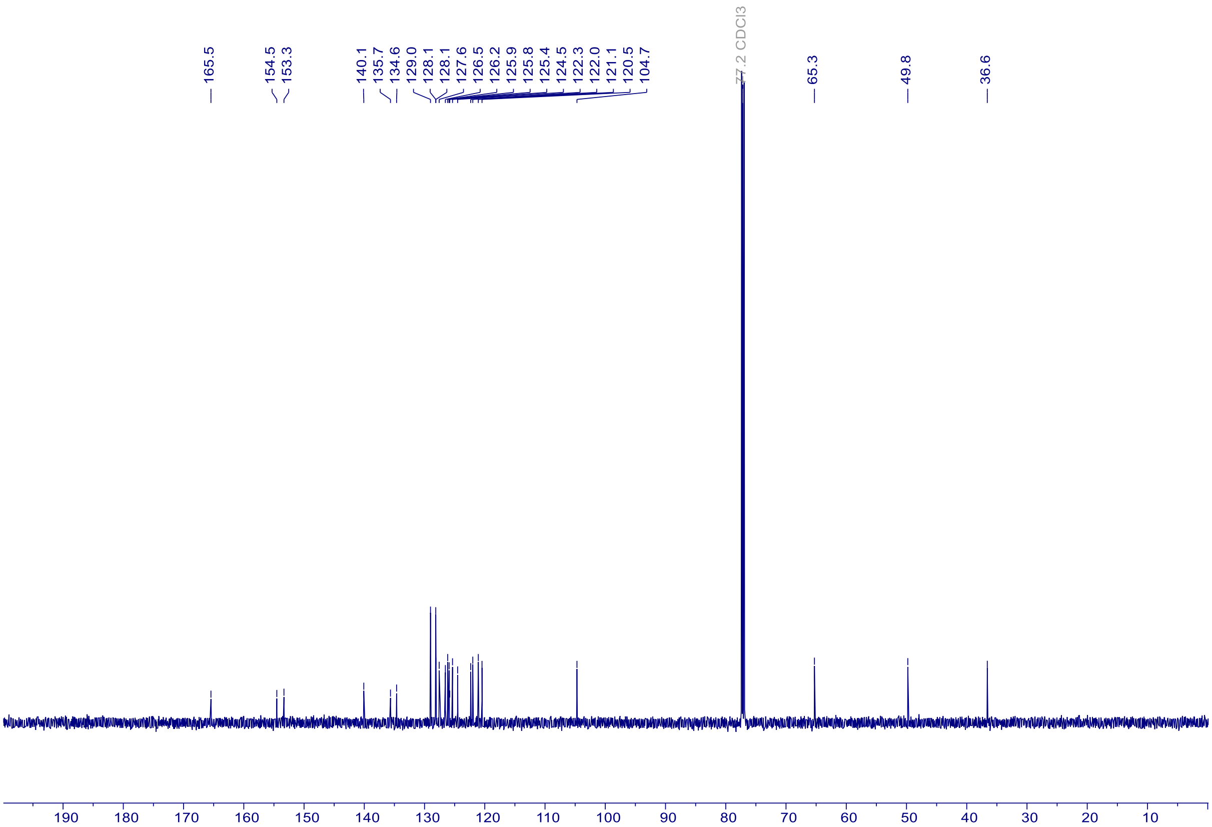

6n – ^{1}H NMR (600 MHz, CDCl$_3$)

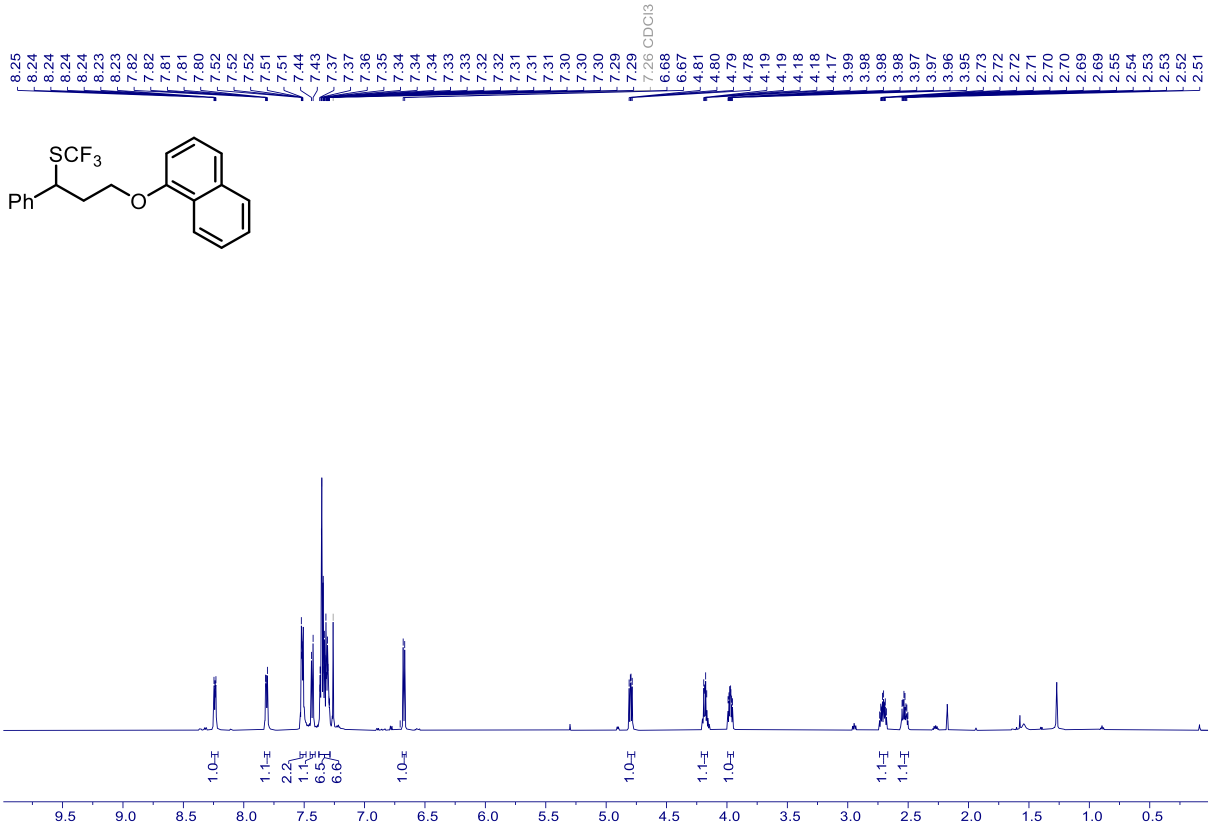

6n – ^{13}C NMR (151 MHz, CDCl$_3$)

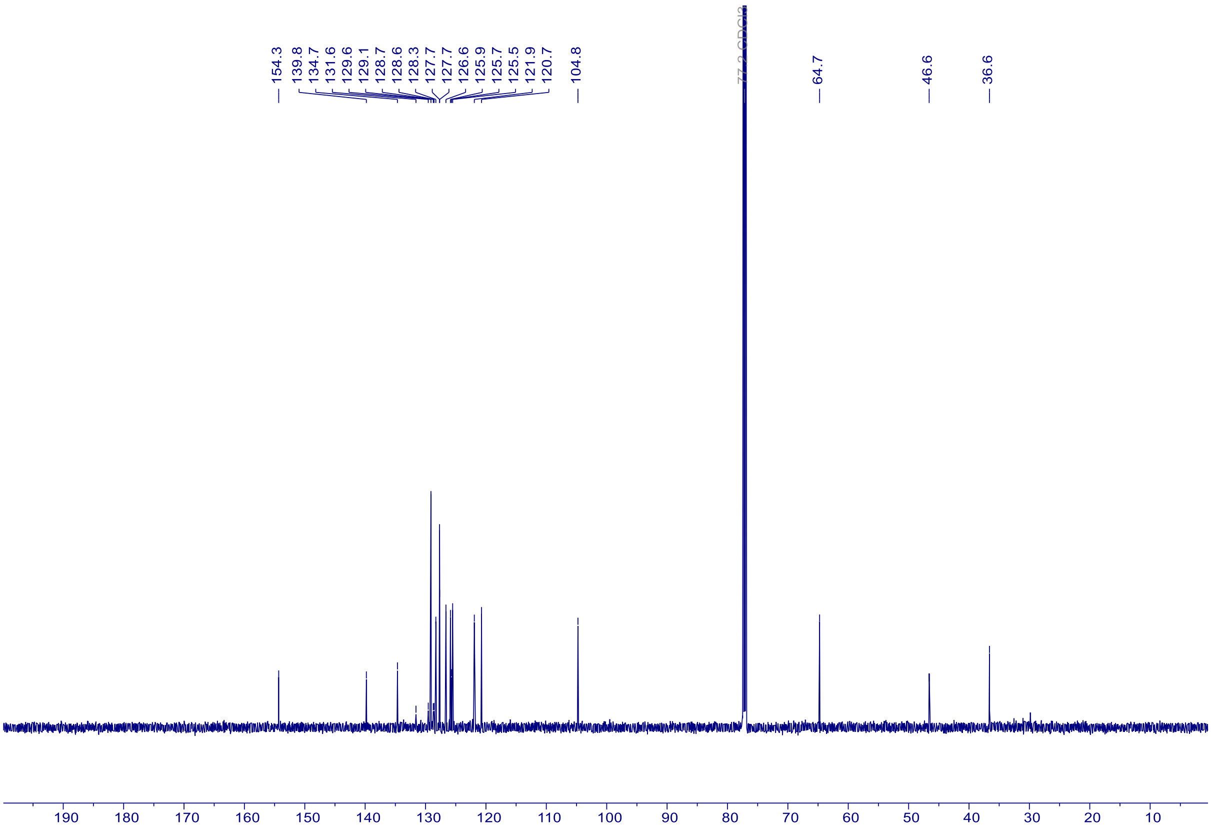

6n – ^{19}F NMR (565 MHz, $CDCl_3$)

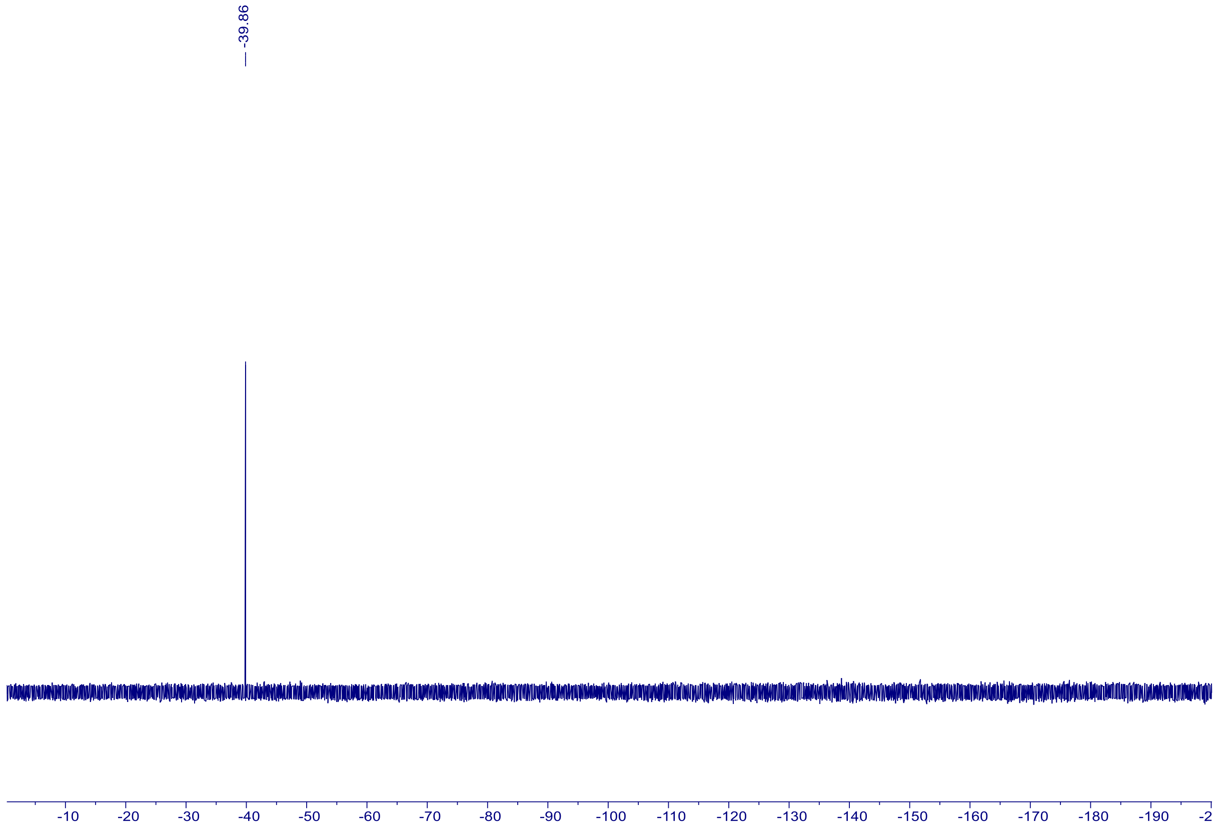

15 Source Data Information

All the source data regarding the computational studies can be found in the provided zip file.

16 References

1. Górski, B., Barthelemy, A.-L., Douglas, J. J., Juliá, F. & Leonori, D. Copper-catalysed amination of alkyl iodides enabled by halogen-atom transfer. *Nat. Catal.* **4**, 623–630 (2021).
2. Guo, P. *et al.* Dynamic Kinetic Cross-Electrophile Arylation of Benzyl Alcohols by Nickel Catalysis. *J. Am. Chem. Soc.* **143**, 513–523 (2021).
3. Volkov, A. *et al.* Mild Deoxygenation of Aromatic Ketones and Aldehydes over Pd/C Using Polymethylhydrosiloxane as the Reducing Agent. *Angew. Chem. Int. Ed.* **54**, 5122–5126 (2015).
4. Kumar, G., Bhattacharya, D., Mistry, P. & Chatterjee, I. In-Catalyzed Transfer Hydrogenation and Regioselective Hydrogen–Deuterium Addition to the Olefins. *J. Org. Chem.* **88**, 6987–6994 (2023).
5. Jain, S., Anmol, Sharma, R., Karmakar, T. & Yadav, M. R. Cu(I)/N,N-Imine Ligand Catalyzed C(sp3)–C(sp) Coupling of Alkyl Bromides with Alkynes: Scope and Mechanistic Investigation. *Org. Lett.* **25**, 5437–5442 (2023).
6. Chang, A. S. *et al.* Alkene Isomerization Using a Heterogeneous Nickel-Hydride Catalyst. *J. Am. Chem. Soc.* **146**, 15596–15608 (2024).
7. Yasui, M., Ota, R., Tsukano, C. & Takemoto, Y. Synthesis of cis-/All-cis-Substituted Cyclopropanes through Stereocontrolled Metalation and Pd-Catalyzed Negishi Coupling. *Org. Lett.* **20**, 7656–7660 (2018).
8. Li, J.-L. *et al.* Redox-Neutral Carboxylation of Benzylic Tertiary C−H Bonds with Carbon Dioxide. *Angew. Chem. Int. Ed.* **64**, e202420852 (2025).
9. Wei, X.-J. *et al.* Visible-Light-Promoted Iron-Catalyzed C(sp2)–C(sp3) Kumada Cross-Coupling in Flow. *Angew. Chem. Int. Ed.* **58**, 13030–13034 (2019).
10. Polterauer, D. *et al.* Deoxyfluorination of Ketones with Sulfur Tetrafluoride (SF_4) and Dialkylamines in Continuous Flow Mode. *Org. Process Res. Dev.* **28**, 2919–2927 (2024).

11. Xie, F. *et al.* Electroreductive Arylcarboxylation of Styrenes with CO2 and Aryl Halides via a Radical–Polar Crossover Mechanism. *Org. Lett.* **26**, 4427–4432 (2024).
12. Mao, J. *et al.* Cobalt–Bisoxazoline-Catalyzed Asymmetric Kumada Cross-Coupling of Racemic α-Bromo Esters with Aryl Grignard Reagents. *J. Am. Chem. Soc.* **136**, 17662–17668 (2014).
13. Zhang, T. *et al.* Transition-metal-free and base promoted C–C bond formation via C–N bond cleavage of organoammonium salts. *Org. Biomol. Chem.* **19**, 8237–8240 (2021).
14. Zheng, Y. *et al.* Green Esterification of Carboxylic Acids Promoted by tert-Butyl Nitrite. *Eur. J. Org. Chem.* **2021**, 2713–2718 (2021).
15. Yang, C., Gao, Y., Bai, S., Jiang, C. & Qi, X. Chemoselective Cross-Coupling of gem-Borazirconocene Alkanes with Aryl Halides. *J. Am. Chem. Soc.* **142**, 11506–11513 (2020).
16. Junaid, M., Happy, S. & Yadagiri, D. Light-induced arylation (alkylation) of N-sulfonylhydrazones with boronic acids. *Chem. Commun.* **60**, 2796–2799 (2024).
17. Fu, W. *et al.* Copper-Catalyzed Site-Selective Electrophilic Aromatic Alkylation of Monosubstituted Simple Arenes. *Org. Lett.* **26**, 2546–2551 (2024).
18. Chen, Y.-G. *et al.* Nickel-catalyzed Enantioselective Hydroarylation and Hydroalkenylation of Styrenes. *J. Am. Chem. Soc.* **141**, 3395–3399 (2019).
19. Nayak, M. K., Mohanty, A. & Roy, S. NHC as a Ligand in Heterobimetallic Catalysis: An Insight into a Catalytic Friedel–Crafts-like Reaction. *Organometallics* **42**, 1927–1933 (2023).
20. Peng, P.-K., Donald, C. P., Dong, Z. & May, J. A. Photoactivation of Hydrazones for the Synthesis of Diarylalkanes and Trialkylmethylboronates: The Key Role Played by Soluble Base. *Org. Lett.* **26**, 3397–3400 (2024).

21. Greb, A. *et al.* A Versatile Route to Unstable Diazo Compounds via Oxadiazolines and their Use in Aryl–Alkyl Cross-Coupling Reactions. *Angew. Chem. Int. Ed.* **56**, 16602–16605 (2017).

22. Papaplioura, E., Templ, J., Wildhack, N. & Schnürch, M. Efficient Synthesis of 2-Arylpropionitriles Via Selective Monomethylation of Aryl Acetonitriles Using an Easy to Handle Methylation Agent. *Eur. J. Org. Chem.* **27**, e202400693 (2024).

23. Franceschi, P. *et al.* A Proton-Coupled Electron Transfer Strategy to the Redox-Neutral Photocatalytic CO2 Fixation. *J. Org. Chem.* **88**, 6454–6464 (2023).

24. Joshi-Pangu, A., Ganesh, M. & Biscoe, M. R. Nickel-Catalyzed Negishi Cross-Coupling Reactions of Secondary Alkylzinc Halides and Aryl Iodides. *Org. Lett.* **13**, 1218–1221 (2011).

25. Li, C. *et al.* Catalyst-free benzylic C(sp3)–H cross-coupling with organotrifluoroborates enabled by electrochemistry. *Green Chem.* **24**, 7883–7888 (2022).

26. Xu, W., Liu, Y., Kato, T. & Maruoka, K. The Formation of C–C or C–N Bonds via the Copper-Catalyzed Coupling of Alkylsilyl Peroxides and Organosilicon Compounds: A Route to Perfluoroalkylation. *Org. Lett.* **23**, 1809–1813 (2021).

27. Okoromoba, O. E. *et al.* Copper-Catalyzed C(sp3)−H α-Acetylation: Generation of Quaternary Centers. *Angew. Chem. Int. Ed.* **64**, e202418692 (2025).

28. Umeda, R., Takahashi, Y. & Nishiyama, Y. Rhenium complex-catalyzed coupling reaction of enol acetates with alcohols. *Tetrahedron Lett.* **55**, 6113–6116 (2014).

29. Nishimoto, Y., Yasuda, M. & Baba, A. Coupling Reaction of Alkyl Chlorides with Silyl Enolates Catalyzed by Indium Trihalide. *Org. Lett.* **9**, 4931–4934 (2007).

30. Ledwith, P. R. *et al.* A Strategy for the Formal C−N Cross-Coupling of Tertiary Amines. *Angew. Chem. Int. Ed.* **63**, e202411555 (2024).

31. Niu, L. *et al.* Manganese-Catalyzed Oxidative Azidation of C(sp3)–H Bonds under Electrophotocatalytic Conditions. *J. Am. Chem. Soc.* **142**, 17693–17702 (2020).

32. Zhang, S. *et al.* Electrochemical Benzylic C(sp3)–H Isothiocyanation. *Org. Lett.* **24**, 1742–1746 (2022).

33. Adamek, J., Mazurkiewicz, R., Węgrzyk, A. & Erfurt, K. 1-Imidoalkylphosphonium salts with modulated Cα–P+ bond strength: synthesis and application as new active α-imidoalkylating agents. *Beilstein J. Org. Chem.* **13**, 1446–1455 (2017).

34. Sang, R. & Gestwicki, J. E. Radical Strategy to the Boron-to-Copper Transmetalation Problem: *N* -Alkylation with Alkylboronic Esters. *J. Am. Chem. Soc.* **147**, 23259–23269 (2025).

35. Howard, E.-L., Guzzardi, N., Tsanova, V. G., Stika, A. & Patel, B. Highly Efficient Copper-Catalyzed Amidation of Benzylic Hydrocarbons Under Neutral Conditions. *Eur. J. Org. Chem.* **2018**, 794–797 (2018).

36. Chen, X., Lian, Z. & Kramer, S. Enantioselective Intermolecular Radical Amidation and Amination of Benzylic C−H Bonds via Dual Copper and Photocatalysis. *Angew. Chem. Int. Ed.* **62**, e202217638 (2023).

37. Liang, G. *et al.* Photocatalytic Generation of Carbocation from Thiols and Application to Cross-Nucleophile Coupling. *Org. Lett.* **26**, 4286–4291 (2024).

38. Vasilopoulos, A., Krska, S. W. & Stahl, S. S. C(sp3)–H methylation enabled by peroxide photosensitization and Ni-mediated radical coupling. *Science* **372**, 398–403 (2021).

39. Dai, C. *et al.* One-Pot Synthesis of α-Branched N-Acylamines via Titanium-Mediated Condensation of Amides, Aldehydes, and Organometallics. *Org. Lett.* **19**, 1064–1067 (2017).

40. Vargová, D., Mudráková, B., Némethová, I. & Šebesta, R. Reductions of Imines Using Zirconocene Chloride Hydride. *Eur. J. Org. Chem.* **2019**, 7606–7612 (2019).

41. Koepler, O. *et al.* Towards a Total Synthesis of Quinocarcin: Diastereoselective Synthesis of Functionalized Azepino[1,2-b]isoquinolines. *Eur. J. Org. Chem.* **2004**, 3611–3622 (2004).

42. Procter, G., Nally, J. & Ordsmith, N. H. R. β-Lactams from tetrahydro-1,2-oxazine-3,6-diones, and a labelling study of the product stereochemistry. *Tetrahedron* **51**, 12837–12842 (1995).

43. Lee, J. M., Park, E. J., Cho, S. H. & Chang, S. Cu-Facilitated C−O Bond Formation Using N-Hydroxyphthalimide: Efficient and Selective Functionalization of Benzyl and Allylic C−H Bonds. *J. Am. Chem. Soc.* **130**, 7824–7825 (2008).

44. Subaramanian, M., Ramar, P. M., Rana, J., Gupta, V. K. & Balaraman, E. Catalytic conversion of ketones to esters via C(O)–C bond cleavage under transition-metal free conditions. *Chem. Commun.* **56**, 8143–8146 (2020).

45. Zhao, C., Sojdak, C. A., Myint, W. & Seidel, D. Reductive Etherification via Anion-Binding Catalysis. *J. Am. Chem. Soc.* **139**, 10224–10227 (2017).

46. Atkins, A. P., Rowett, A. C., Heard, D. M., Tate, J. A. & Lennox, A. J. J. Electrochemical Benzylic C(sp3)–H Acyloxylation. *Org. Lett.* **24**, 5105–5108 (2022).

47. Chênevert, R., Pelchat, N. & Morin, P. Lipase-mediated enantioselective acylation of alcohols with functionalized vinyl esters: acyl donor tolerance and applications. *Tetrahedron Asymmetry* **20**, 1191–1196 (2009).

48. Sun, K. *et al.* Iron-catalyzed benzylic C–H thiolation via photoinduced ligand-to-metal charge-transfer. *Chem. Commun.* **60**, 5755–5758 (2024).

49. Savolainen, M. A. & Wu, J. Markovnikov-Selective Hydrothiolation of Styrenes: Application to the Synthesis of Stereodefined Trisubstituted Olefins. *Org. Lett.* **15**, 3802–3804 (2013).

50. Zachmann, A. K. Z., Drappeau, J. A., Liu, S. & Alexanian, E. J. C(sp3)−H (N-Phenyltetrazole)thiolation as an Enabling Tool for Molecular Diversification. *Angew. Chem. Int. Ed.* **63**, e202404879 (2024).

51. Ortalli, S., Ford, J., Trabanco, A. A., Tredwell, M. & Gouverneur, V. Photoredox Nucleophilic (Radio)fluorination of Alkoxyamines. *J. Am. Chem. Soc.* **146**, 11599–11604 (2024).

52. Zhang, Z., Poletti, L. & Leonori, D. A Radical Strategy for the Alkylation of Amides with Alkyl Halides by Merging Boryl Radical-Mediated Halogen-Atom Transfer and Copper Catalysis. *J. Am. Chem. Soc.* **146**, 22424–22430 (2024).

53. Thiemsorn, W., Keowkamnerd, K., Suwannathada, P., Hessenkemper, H. & Phanichaphant, S. Redox ratio and optical absorption of polyvalent ions in industrial glasses. *Bull. Mater. Sci.* **30**, 487–495 (2007).

54. Young, T. A., Silcock, J. J., Sterling, A. J. & Duarte, F. autodE: Automated Calculation of Reaction Energy Profiles— Application to Organic and Organometallic Reactions. *Angew. Chem.* **133**, 4312–4320 (2021).

55. Adamo, C. & Barone, V. Toward reliable density functional methods without adjustable parameters: The PBE0 model. *J. Chem. Phys.* **110**, 6158–6170 (1999).

56. Weigend, F. & Ahlrichs, R. Balanced basis sets of split valence, triple zeta valence and quadruple zeta valence quality for H to Rn: Design and assessment of accuracy. *Phys. Chem. Chem. Phys.* **7**, 3297–3305 (2005).

57. Becke, A. D. Density-functional exchange-energy approximation with correct asymptotic behavior. *Phys. Rev. A* **38**, 3098–3100 (1988).

58. Density Functional Theory of Electronic Structure | The Journal of Physical Chemistry. https://pubs.acs.org/doi/10.1021/jp960669l.

59. Neese, F. The ORCA program system. *WIREs Comput. Mol. Sci.* **2**, 73–78 (2012).

60. Long-Range Corrected Hybrid Density Functionals with Improved Dispersion Corrections | Journal of Chemical Theory and Computation. https://pubs.acs.org/doi/10.1021/ct300715s.

61. A consistent and accurate ab initio parametrization of density functional dispersion correction (DFT-D) for the 94 elements H-Pu | The Journal of Chemical Physics | AIP Publishing. https://pubs.aip.org/aip/jcp/article/132/15/154104/926936/A-consistent-and-accurate-ab-initio.

62. Weigend, F. Accurate Coulomb-fitting basis sets for H to Rn. *Phys. Chem. Chem. Phys.* **8**, 1057–1065 (2006).

63. Ishida, K., Morokuma, K. & Komornicki, A. The intrinsic reaction coordinate. An ab initio calculation for HNC→HCN and H−+CH4→CH4+H−. *J. Chem. Phys.* **66**, 2153–2156 (1977).

64. Marenich, A. V., Cramer, C. J. & Truhlar, D. G. Universal Solvation Model Based on Solute Electron Density and on a Continuum Model of the Solvent Defined by the Bulk Dielectric Constant and Atomic Surface Tensions. *J. Phys. Chem. B* **113**, 6378–6396 (2009).

65. Young, T. duartegroup/otherm: Major symmetry improvements. Zenodo: 2020. DOI: 10.5281/zenodo.4005686.

66. Johnson, E. R. *et al.* Revealing Noncovalent Interactions. *J. Am. Chem. Soc.* **132**, 6498–6506 (2010).

67. Lu, T. & Chen, F. Multiwfn: A multifunctional wavefunction analyzer. *J. Comput. Chem.* **33**, 580–592 (2012).

68. Lu, T. A comprehensive electron wavefunction analysis toolbox for chemists, Multiwfn. *J. Chem. Phys.* **161**, 082503 (2024).

69. Humphrey, W., Dalke, A. & Schulten, K. VMD: Visual molecular dynamics. *J. Mol. Graph.* **14**, 33–38 (1996).

70. Williams, T. & Kelley, C. Gnuplot 4.6: an interactive plotting program (2019).

71. Steam, A. E., Irish, E. M. & Eyring, H. A Theory of Diffusion in Liquids. *J. Phys. Chem.* **44**, 981–995 (1940).

72. Yanai, T., Tew, D. P. & Handy, N. C. A new hybrid exchange–correlation functional using the Coulomb-attenuating method (CAM-B3LYP). *Chem. Phys. Lett.* **393**, 51–57 (2004).

73. Zhao, Y. & Truhlar, D. G. The M06 suite of density functionals for main group thermochemistry, thermochemical kinetics, noncovalent interactions, excited states, and transition elements: two new functionals and systematic testing of four M06-class functionals and 12 other functionals. *Theor. Chem. Acc.* **120**, 215–241 (2008).

74. Mardirossian, N. & Head-Gordon, M. ωB97M-V: A combinatorially optimized, range-separated hybrid, meta-GGA density functional with VV10 nonlocal correlation. *J. Chem. Phys.* **144**, 214110 (2016).

75. Becke, A. D. Density-functional thermochemistry. III. The role of exact exchange. *J. Chem. Phys.* **98**, 5648–5652 (1993).

76. Grimme, S., Ehrlich, S. & Goerigk, L. Effect of the damping function in dispersion corrected density functional theory. *J. Comput. Chem.* **32**, 1456–1465 (2011).

77. Zhang, Z., Tilby, M. J. & Leonori, D. Boryl radical-mediated halogen-atom transfer enables arylation of alkyl halides with electrophilic and nucleophilic coupling partners. *Nat. Synth.* **3**, 1221–1230 (2024).

78. Zhang, L. & Cao, Z. Formation and reactivity of NHC-boryl radicals: insight into substituent effect from theoretical calculations. *Phys. Chem. Chem. Phys.* **25**, 12072–12080 (2023).

79. Walton, J. C., Dai, W. & Curran, D. P. EPR Studies on the Addition of Ligated Boryl Radicals to Carbonyl Compounds. *J. Org. Chem.* **85**, 4248–4255 (2020).
80. Kuehn, L. *et al.* NHC induced radical formation via homolytic cleavage of B–B bonds and its role in organic reactions. *Chem. Sci.* **13**, 8321–8333 (2022).
81. A pyridine-boryl radical mediated cascade reaction towards the synthesis of indolizines: a computational mechanistic analysis - Organic Chemistry Frontiers (RSC Publishing). https://pubs.rsc.org/en/content/articlelanding/2024/qo/d4qo00558a.
82. Formation of N-Heterocyclic Carbene–Boryl Radicals through Electrochemical and Photochemical Cleavage of the B–S bond in N-Heterocyclic Carbene–Boryl Sulfides | Journal of the American Chemical Society. https://pubs.acs.org/doi/10.1021/ja4066267.
83. Klyukin, I. N., Kolbunova, A. V., Novikov, A. S., Zhizhin, K. Y. & Kuznetsov, N. T. Theoretical Investigation of Bond Dissociation Energies of exo-Polyhedral B–H and B–F Bonds of closo-Borate Anions [BnHn−1X]2− (n = 6, 10, 12; X = H, F). *Computation* **13**, 28 (2025).
84. Hohenberg, P. & Kohn, W. Inhomogeneous Electron Gas. *Phys. Rev.* **136**, B864–B871 (1964).
85. Becke, A. D. Exchange?correlation approximations in density-functional theory. in *Modern Electronic Structure Theory* 1022–1046 (World Scientific Publishing Company, 1995). doi:10.1142/9789812832115_0004.
86. Cohen, A. J., Mori-Sánchez, P. & Yang, W. Insights into Current Limitations of Density Functional Theory. *Science* **321**, 792–794 (2008).
87. Zupan, A., Burke, K., Ernzerhof, M. & Perdew, J. P. Distributions and averages of electron density parameters: Explaining the effects of gradient corrections. *J. Chem. Phys.* **106**, 10184–10193 (1997).

88. Lu, T. Visualization Analysis of Covalent and Noncovalent Interactions in Real Space. *Angew. Chem. Int. Ed.* **64**, e202504895 (2025).

89. Lu, T. & Chen, Q. Visualization Analysis of Weak Interactions in Chemical Systems. in *Comprehensive Computational Chemistry (First Edition)* (eds. Yáñez, M. & Boyd, R. J.) 240–264 (Elsevier, Oxford, 2024). doi:10.1016/B978-0-12-821978-2.00076-3.

90. Kitaigorodsky, A. I. Non-bonded interactions of atoms in organic crystals and molecules. *Chem. Soc. Rev.* **7**, 133–163 (1978).
